# FERN – a Java framework for stochastic simulation and evaluation of reaction networks

**DOI:** 10.1186/1471-2105-9-356

**Published:** 2008-08-29

**Authors:** Florian Erhard, Caroline C Friedel, Ralf Zimmer

**Affiliations:** 1 LFE Bioinformatik, Institut für Informatik, Ludwig-Maximilians-Universität München, Amalienstraße 17, 80333 München, Germany

## Abstract

**Background:**

Stochastic simulation can be used to illustrate the development of biological systems over time and the stochastic nature of these processes. Currently available programs for stochastic simulation, however, are limited in that they either a) do not provide the most efficient simulation algorithms and are difficult to extend, b) cannot be easily integrated into other applications or c) do not allow to monitor and intervene during the simulation process in an easy and intuitive way. Thus, in order to use stochastic simulation in innovative high-level modeling and analysis approaches more flexible tools are necessary.

**Results:**

In this article, we present FERN (Framework for Evaluation of Reaction Networks), a Java framework for the efficient simulation of chemical reaction networks. FERN is subdivided into three layers for network representation, simulation and visualization of the simulation results each of which can be easily extended. It provides efficient and accurate state-of-the-art stochastic simulation algorithms for well-mixed chemical systems and a powerful observer system, which makes it possible to track and control the simulation progress on every level. To illustrate how FERN can be easily integrated into other systems biology applications, plugins to Cytoscape and CellDesigner are included. These plugins make it possible to run simulations and to observe the simulation progress in a reaction network in real-time from within the Cytoscape or CellDesigner environment.

**Conclusion:**

FERN addresses shortcomings of currently available stochastic simulation programs in several ways. First, it provides a broad range of efficient and accurate algorithms both for exact and approximate stochastic simulation and a simple interface for extending to new algorithms. FERN's implementations are considerably faster than the C implementations of gillespie2 or the Java implementations of ISBJava. Second, it can be used in a straightforward way both as a stand-alone program and within new systems biology applications. Finally, complex scenarios requiring intervention during the simulation progress can be modelled easily with FERN.

## Background

Traditionally, wet-lab experiments were focused on describing the function of individual genes or proteins. With the advent of high-throughput technologies, system-level approaches have become common which make it possible to identify the interactions between the individual elements of the cell. Here, mathematical models are crucial in understanding these biological systems. In particular the dynamic simulation of these models can illustrate and predict quantitative aspects of the system such as gene expression in regulatory networks or signal amplification in signal transduction networks [1].

The most common approach to modeling dynamics is via ordinary differential equations (ODEs) which describe deterministically how the system evolves with time (see e.g. [2-4]). Since the simulation of ODEs is deterministic, successive simulations starting from the same initial conditions lead to the same results. Biological systems, however, are not deterministic which can lead to quite different outcomes for the same initial conditions.

**Figure 1:**
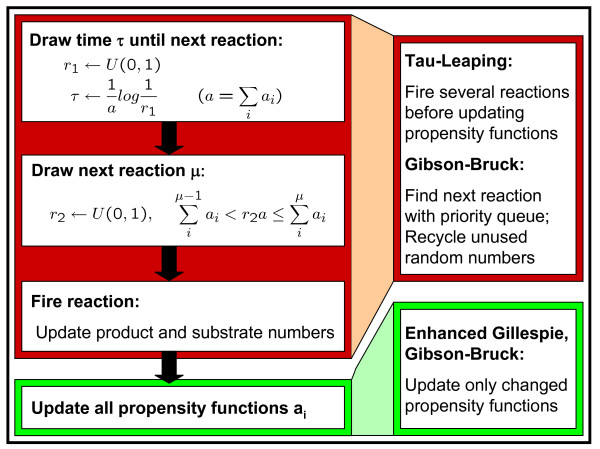
**Stochastic simulation**. This figure shows the flow of one simulation step. On the left-hand side the flow for the original Gillespie algorithm can be seen. On the right-hand side, we illustrate how the different steps are modified by the Gibson-Bruck, enhanced Gillespie and tau-leaping algorithms. Here, *U*(0, 1) denotes the uniform distribution on the range of 0 to 1 and *a*_*μ *_the reaction propensity for reaction *μ*.

The stochastic nature of biological systems can be simulated using numerical simulation algorithms such as the stochastic simulation algorithm (SSA) of Gillespie [5]. The Gillespie algorithm simulates the system reaction by reaction. A reaction step in this case consists of two parts (see Figure 1). First, the time interval *τ *until the next reaction is drawn from the exponential distribution *P*(*τ*) = *a *exp(-*aτ*) using the inversion method. Here, *a *is the sum over all reaction propensities *a*_*μ*_. Second, the reaction *μ *which is to occur in this time interval is drawn with propability *P*(*μ*|*τ*) = *a*_*μ*_/*a*. At the end of each step, molecule numbers and reaction propensities are updated. Both simulations via ODEs and SSAs assume a well-mixed system with a homogeneous distribution of molecules.

The original Gillespie algorithm (also called the direct method) has been modified in several ways to improve runtime. Here, the most commonly used modification is the next-reaction method by Gibson and Bruck [6]. This algorithm improves on a less efficient variant of the direct method which generates *τ*_*k *_for each reaction *R*_*k *_and then fires the reaction with the minimum *τ*_*k*_. The minimum *τ*_*k *_and the corresponding reaction are obtained from a priority queue and time intervals are updated without drawing new random numbers only for those reactions whose propensity was changed by the firing of the reaction. Reactions with changed propensities are identified with a dependency graph which contains an edge from reaction *R*_*i *_to reaction *R*_*j *_if *R*_*i *_changes the molecule number of at least one reactant of *R*_*j*_. It has been been suggested that the next-reaction method is actually less efficient than improved versions of the direct method [7]. Indeed, we could show in our implementation that a variant of the direct method which uses the dependency graph technique to update propensities is significantly faster than the Gibson and Bruck algorithm.

The direct and next-reaction methods are exact methods. This means reaction propensities are updated after each reaction. Recently, Gillespie [8] proposed an approximative method, *tau-leaping*, which performs all reactions in a certain interval *τ *before updating the propensity functions. The interval size *τ *is chosen such that the propensity functions remain almost constant in this interval and reactions may fire multiple times. This, however, can sometimes lead to negative populations and as a consequence, this method has been improved later by Cao et al. [9,10] to avoid this problem. The modified tau-leaping algorithm automatically switches to the exact SSA for a few steps if the choice of *τ *becomes too small.

Both exact and tau-leaping models cannot be used to efficiently simulate models with multiple scales in molecule concentrations or reaction rates. Exact methods are too inefficient to simulate many fast reactions and high molecule concentrations. On the other hand, the presence of low molecule concentrations and slow reactions in the systems will effectively lead to small *τ *values for the tau-leaping methods and thus make them behave as the exact methods. To circumvent these problems, hybrid methods have been developed which partition the system into fast and slow reactions [11-26]. The slow reactions are then generally simulated using the exact SSA. The fast reactions are solved either deterministically or with the Langevin equation [11-18] or simulated with tau-leaping methods [18,19]. Alternatively, the model is simplified such that the effect of the fast reactions is incorporated in the simulation of the slow reactions, e.g. using quasi-steady-state assumptions, without actually firing the fast reactions [20-26].

Several implementations of stochastic simulation algorithms are already available, e.g. COPASI [27], Dizzy [28] using the SSA implementations of the ISBJava library, gillespie2 [29], STOCKS [30], StochKit [31], and BioNetS [32]. In general, these programs were designed as stand-alone programs and as a consequence the user is limited to the functionalities of the user interface. This makes it difficult to use the implementations of the SSAs within other programs. Furthermore, most of these programs provide only one implementation of an exact SSA which is not always fast enough for practical systems biology applications. However, faster SSAs such as e.g. the approximative tau-leaping procedure or new hybrid algorithms cannot be added to the programs easily by the users.

The StochKit software and ISBJava library provide these faster tau-leaping algorithms and the latter was also designed to be used within other systems biology programs. The output of the corresponding SSA implementations, however, is limited to the molecule concentrations. More flexible implementations are necessary to simulate complex high-level models and integrate stochastic simulation algorithms in new and innovative analysis and modeling tools. Two examples which illustrate the need for more flexible tools (see also results) are the visualization of the simulation progress directly in a network and the simulation of cell growth and division. With current simulation tools, it is not possible to implement both examples without having to change the code of the actual simulation algorithms considerably.

**Figure 2:**
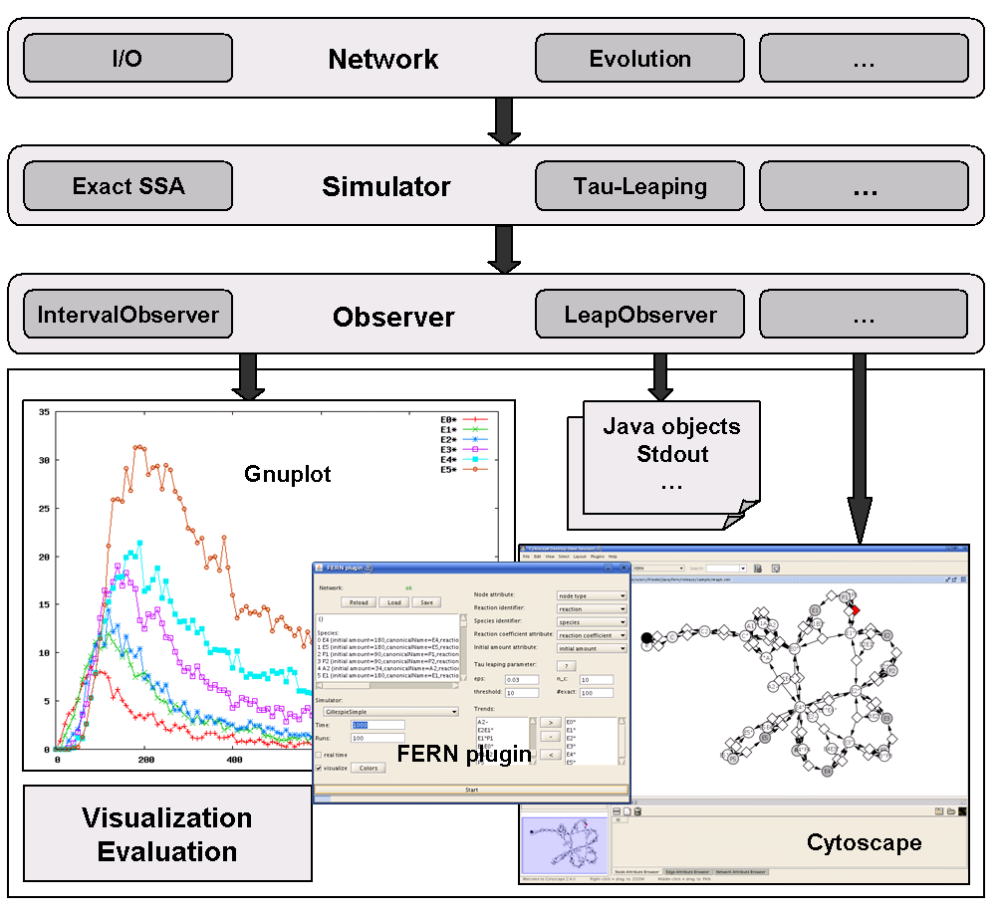
**FERN design**. The figure illustrates the overall design of FERN into three layers. Each layer is represented by one interface or abstract class: Interface *Network *and abstract classes *Simulator *and *Observer*.

In this article, we present FERN, a Java framework for modeling and simulating biological systems which provides accurate and state-of-the-art simulation algorithms (exact, approximate and hybrid) and has been designed to be easily extendable to new ones (see Figure 2). With the help of observers, the simulation progress can be monitored on every level and modifications to the systems can be introduced during simulations in an intuitive way. Even with these additional functionalities, the implemented algorithms are faster than the ISBJava implementations. Results can be visualized easily and networks can be loaded from different sources. Contrary to ISBJava, FERN supports the most current version of SBML and allows arbitrary rate law definitions. FERN can be used as an integral part of other Java applications or as a standalone program in the form of a command-line tool and plugins to Cytoscape and CellDesigner.

## Implementation

FERN is an object oriented library implemented in Java (see Additional file 1). Although it consists of more than 100 classes and interfaces, most classes are just implementations of one of three major interfaces and abstract classes (see Figure 2):

1. The interface *Network *provides the network structure of the model.

2. The abstract class *Simulator *performs simulations on a *Network*. It additionally calls the registered observers during the simulation run.

3. The abstract class *Observer *traces the simulation progress and creates the simulation output.

**Figure 3:**
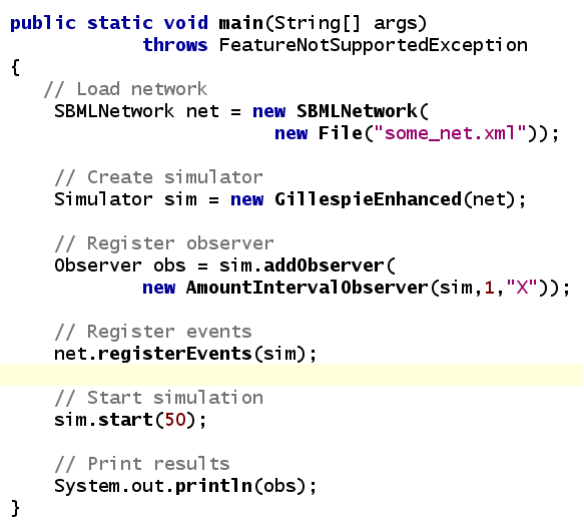
**Example program**. This figure shows a small example on how to use FERN for running a simulation on a network. First, a network is loaded from an SBML file and then a simulator is created. In the next step, an observer is created and registered with the simulator. In this example, the observer records the current amount of molecule *X *every second of simulated time. The SBML events are registered with the simulator and the simulation is started to run for 50 seconds. Finally, the recorded results for *X *are printed.

A simple simulation can be performed in only five lines of code, one line for each of: loading a network file, creating a simulator, creating and registering an observer, running the simulations and printing the results (see Figure 3). More complex examples for using FERN can be found in the FERN distribution. In the following the three layers of FERN are described in more detail.

### Networks

**Figure 4:**
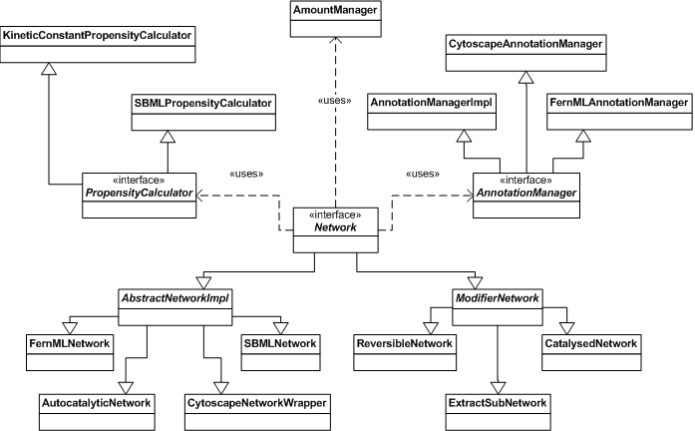
**UML diagram of Network related classes**. This figure shows an UML diagram for the *Network *interface and related interfaces and classes.

The interface *Network *describes the network's structure, i.e. the reactions and species in the networks. For this purpose, each reaction and each species is described by an integer value. Furthermore, the network stores basic information like species names and their initial molecule numbers. For the simulation more information is necessary which is stored in three additional classes (see Figure 4):

• The *AmountManager *controls the amount of each molecular species during the course of a simulation.

• The *AnnotationManager *can store additional annotations for the network, its species and reactions.

• The *PropensityCalculator *calculates the propensities for the reactions by the specified kinetic laws.

There are three types of implementations of the *Network *interface:

• *Readers *which can read network data from files (e.g. FernMLNetwork, SBMLNetwork)

• *Wrappers *which redirect method calls to existing network classes (e.g. CytoscapeNetworkWrapper)

• *Evolution algorithms *which create networks from scratch by certain rules (e.g. AutocatalyticNetwork)

For each network, stochastic simulations can be performed with all implemented simulation algorithms.

#### Import and export of networks

FERN supports two formats for loading and exporting networks: the SBML format [33] as well as the simpler but also XML based FernML format. For reading and writing the SBML format, FERN uses the Java bindings of the C library (libSBML) available at . Thus, it can be easily adapted to new developments of the SBML format. Currently, SBML version 2 levels 1–3 are supported.

From the model loaded by libSBML from the SBML file, a FERN *SBMLNetwork *is created using the list of compartments, species, reactions, parameters and events in the model. Events have to be registered with a simulator by the *SBMLNetwork *if they are to be triggered during the simulation (see Figure 3 for an example). Triggering of events is handled by specific observers.

Currently, the *SBMLNetwork *class uses only the features of SBML necessary for the simulation of the network. It supports MathML to define complex reaction mechanisms but not rules, constraints or function definitions. If these features are required they can be incorporated easily by extending the SBMLNetwork class and loading these features from the SBML model created by libSBML. Since many systems biology applications support SBML (e.g. CellDesigner [34]), the SBML format can be used as an interchange format between FERN and these other applications.

SBML is a powerful format which can provide lots of information about a model. In contrast, FernML stores only the topology of the reaction network, optional annotations and the simulation parameters (see Additional file 2 and Additional file 3). This results in a much more simplified input format. More complex aspects, such as volume change due to cell growth and division, can then be modeled in Java using the FERN library in a straightforward way (see Results for an example). As a consequence, arbitrarily complex models can be designed.

**Figure 5:**
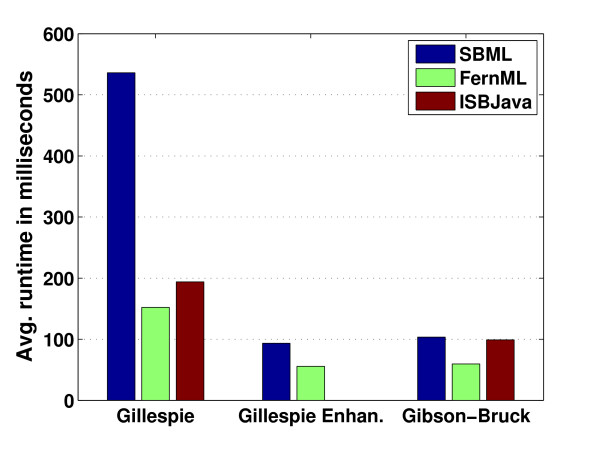
**Runtime Comparisons**. The EGF signaling pathway described by Lee et al. [38] was simulated with the three exact methods provided by FERN (original and enhanced Gillespie algorithm and the next reaction method by Gibson and Bruck) for a simulated time of 800 seconds both with an SBML network using expression trees to represent MathML expressions and a FernML network. For each combination of network type and stochastic simulation algorithm, 1,000 simulations were performed and the average runtime in milliseconds was calculated. The same simulations were performed with the Gillespie and Gibson-Bruck algorithms of ISBJava. All results were obtained on one processor of an Intel Core2Duo with 2.4 GHz. Standard errors in all cases were < 1.5 milliseconds.

Since FernML supports only the reaction rate equations used by Gillespie [5], the propensities can be recalculated at each step efficiently by a few arithmetic operations. SBML uses MathML to store the kinetics of a reaction. This allows for more complex reaction mechanisms and is particularly useful if the model cannot be formulated exclusively with first or higher order rate equations. To evaluate MathML expressions, FERN creates expression trees from them which have to be evaluated every time a propensity is calculated. Since this is one of the essential steps of SSAs, the simulation of an SBML network in FERN can be significantly slower than the simulation of the same network as a FernML network (see Figure 5). Thus, if only simple reaction rate equations are used, an SBML network should be converted to a FernML network using the provided conversion methods before performing the simulation.

FERN is not restricted to the input formats currently available. Any new input format can be easily included by implementing the *Network *interface or extending the *AbstractNetworkImpl *class.

### Simulation algorithms

FERN provides implementations for three exact stochastic simulation algorithms, three state-of-the-art tau leaping procedures (see [8,10]) and a hybrid method combining SSA and tau-leaping [19]. The exact SSAs implemented include the original direct method of Gillespie [5], the next reaction method of Gibson and Bruck [6] and an enhanced version of the direct method. This enhanced method uses the dependency graph technique of the next reaction method to only update the propensity functions which are affected by the firing of a reaction. Apart from this improvement, it is identical to the direct method.

The tau-leaping algorithms are all based on the modified tau-leaping procedure proposed by Cao et al. [9] which avoids the problem of negative populations observed for the original tau-leaping procedure. This method switches to an exact SSA (in our implementations the enhanced Gillespie) for a few steps if the selected *τ *becomes too small. The three implementations differ only in the way the error is bounded (see [10] for details). The error is bounded either by the sum of all propensity functions (*TauLeapingAbsoluteBoundSimulator*), the relative changes in the individual propensity functions (*TauLeapingRelativeBoundSimulator*) or the relative changes in the molecular populations (*TauLeapingSpeciesPopulationBoundSimulator*).

Furthermore, FERN implements the hybrid method by Puchalka and Kierzek [19] which partitions the system during the simulation into slow reactions which involve only small molecule numbers and fast reactions which involve large molecule numbers. The slow reactions are then simulated using an exact SSA while the fast reactions are simulated with tau-leaping. This algorithm was chosen over other hybrid methods for two reasons. First it uses only stochastic simulation algorithms, i.e. exact SSA and tau-leaping, and no further assumptions such as quasi-steady state. Second, the partitioning of the system is performed dynamically according to the state of the system and updated after each reaction step. Our implementation of the hybrid method uses our more efficient enhanced Gillespie algorithm (see Figure 5) instead of the Gibson and Bruck algorithm used by Puchalka and Kierzek. On the model of LacZ and LacY gene expression by Kierzek [30], the hybrid method speeds up the runtime by a factor of 98 compared to the enhanced Gillespie algorithm.

Each simulation algorithm is implemented by extending the abstract class *Simulator *or one of its subclasses. A simulation consists of the following steps (see also Additional file 3). First, the data structures are initialized and the simulation is started by passing a simulation controller implementing the *SimulationController *interface. The simulation controller decides after each step if the simulation can continue. The most basic one is the *DefaultController *which lets the simulation run until a given simulated time is reached.

In each step, the behavior of the simulator depends on the simulation algorithm implemented (see Figure 1). The direct and enhanced Gillespie algorithms draw the time interval *τ *till the next reaction from an exponential distribution. The reaction to be fired is then drawn with a probability proportional to its reaction propensity. For this purpose, a random variable *r*_2 _is first drawn from the uniform distribution between 0 and 1. The corresponding reaction *μ *is then identified via a linear search such that $\sum_{i}^{\mu -1}a_{i}<r_{2}\sum_{i}a_{i}\leq \sum_{i}^{\mu }a_{i}$. The Gibson-Bruck algorithm generates *τ*_*k *_for each reaction *R*_*k *_and at each step fires the reaction with the minimum *τ*_*k *_obtained from a priority queue. Tau-leaping methods also choose a time interval *τ *but in this case several reactions can be fired during this interval (for more details see [9]).

After each step, the molecule numbers for reactants and products and the propensity functions for the reactions are recalculated. Here, reactants and products of a reaction can be identified efficiently from the adjacency list for this reaction stored in the network structure. Propensities are updated efficiently for all simulation algorithms apart from the original Gillespie algorithm using a dependency graph which stores for each reaction all reactions whose propensity is changed by the firing of this reaction.

Future developments of the algorithms can easily be included into FERN by extending one of the SSA implementations or the original *Simulator *class. In the same way ODE solvers or simulators for spatial models which are not provided by FERN can be integrated.

### Observer system

FERN uses observers to trace the simulation progress and react to events. For this purpose, each observer has to implement functions which describe its response at specific time points of the simulations. Such responses may occur either at the beginning or the end of a simulation, before each step, after a reaction is fired or when a certain time is reached. In order to be notified of these events, observers have to be registered with the simulator.

Observer implementations are provided for tracing the molecule numbers for some species in arbitrary intervals, recording the firings of reactions, computing distributions of molecule numbers at a certain time over many simulation runs as well as many others. Several observers can be registered for a simulation at the same time and most of them can also handle repeated simulation runs, e.g. to create average curves or curves containing all trajectories for the individual simulation runs.

### Visualization

In general, the observers use gnuplot to present their results. Once gnuplot is installed on a system and accessible e.g. via the path variable, the *Gnuplot *class makes it possible to easily create plots and retrieve them as *Image *objects, save them as files or present them online in a window. Plots can be customized using appropriate gnuplot commands.

Furthermore, FERN was used to implement Cytoscape [35] and CellDesigner [34] plugins for running and visualizing the simulations from within the Cytoscape or CellDesigner environments (for more details see Results).

### Stochastics

An important feature of FERN is that random number generation is handled by the singleton class *Stochastics*. Accordingly, only one instance of this class is instantiated during a FERN run and all calls for random numbers are referred to this instance. This has several advantages. First, the underlying random number generator can be easily replaced if faster and better random number generators are developed. Currently, the Mersenne Twister implementation of the Colt Project is used . Second, by setting the seed value for the random number generator explicitly, the simulation can be made deterministic and e.g. interesting trajectories can be reproduced. Third, it is possible to count the number of random number generations necessary for different implementations of SSAs.

## Results

### Cytoscape plugin for stochastic simulation

Cytoscape [35] is a software platform for visualizing and integrating networks with an emphasis on biological data. It provides a flexible plugin architecture which can be used to enrich the platform with additional methods. We used this functionality to create a plugin which uses FERN to simulate networks loaded into Cytoscape (see Additional file 3). This plugin makes it possible to track the simulation progress directly on the network. Furthermore, it shows how FERN can be easily integrated into other applications and how the observer system can be used to visualize more than just the changes in molecule numbers. Each network readable by Cytoscape can be used for simulation by the plugin if it consists of two distinct types of nodes, namely reactions and molecular species. Furthermore, the initial amount of each molecular species and the reaction rate coefficient for each reaction have to be given. These parameters and the node type (species or reaction) can be read from arbitrary node attributes specified in Cytoscape. Additionally, the plugin provides access to FernML files in both directions. Thus every Cytoscape network can be saved as FernML, and every FernML file can be loaded into Cytoscape.

Simulations can be performed with every stochastic simulation algorithm provided by FERN and the simulation progress can be visualized directly on the network. Reaction nodes flash up whenever the corresponding reaction is fired and the species nodes are colored according to their molecule numbers. Furthermore, simulations can be run in real-time, which causes the algorithms to pause between two reaction events according to the simulated time. Trend curves of molecular species can also be created using gnuplot.

The implementation of the Cytoscape plugin is straightforward. A central plugin class integrates FERN into the Cytoscape platform by creating a menu item to start the plugin and to load the user interface. Apart from the classes defining the user interface, only a few additional classes are necessary. The most important ones are a wrapper class implementing the *Network *interface to map the Cytoscape network structure to FERN and an *Observer *class to make the visualization possible. Additionally, FERN provides its own *Visual Style *(which defines how nodes and edges are colored and shaped) to guarantee a proper display of the network and to handle the flashing and recoloring of reaction and species nodes, respectively. The Cytoscape plugin was also adapted as a plugin to CellDesigner [34] which now offers a plugin functionality with the recent version 4.0 beta.

### Simulation of cell growth and division using observers

The Cytoscape plugin is one example how observers can be used to track the simulation progress on various levels. Another example which illustrates the potential of the observer system is the simulation of the LacZ model described by Kierzek et al. [30,36] and based on experimental results by Kennell and Riezman [37]. This model requires the simulation of cell division. After each cell division, the stochastic simulation is continued with one promotor molecule and all other molecule numbers divided by 2. RNA polymerase and ribosome molecules are assumed to remain approximately constant with natural variations. For this purpose, the number of these molecules has to be adjusted after each simulation step by drawing from normal distributions. Furthermore, cell growth leads to a linear volume change.

With existing stochastic simulation programs, this model can, in general, only be simulated by changing the code of the actual simulation algorithms. Contrary to that, the model can be easily simulated with FERN by simply defining a cell growth observer. Before each simulation step, the observer checks if a generation has been completed. If this is the case, all molecule numbers are adjusted as described before. In any case, the volume size is adjusted to account for either cell division or cell growth, and the RNA polymerase and ribosome molecule numbers are drawn randomly.

**Figure 6:**
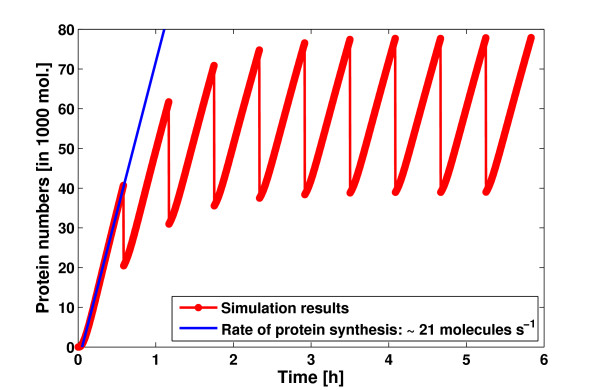
**Results for the LacZ model**. Average results of 1,000 simulations are shown for the LacZ protein over ten bacterial generations (red). After each generation (2100 s) the number molecules for each species was divided by 2 to simulate cell division. The blue line shows a linear fit to the increasing LacZ concentration during the first generation. This yields a rate of protein synthesis of 21*s*^-1^.

This approximation was also used by Kierzek et al. and assumes that cell volume does not change during a simulation step. To perform an exact simulation of volume change, propensity functions would have to be defined which handle the cell volume as a function of time. However, since the volume change during one reaction is extremely small, the differences between the approximate and exact results should be negligible. Using the cell growth observer, we simulated the LacZ model with the enhanced Gillespie algorithm. Our results for the concentration of the LacZ protein (see Figure 6) show clearly the periodic oscillation in the protein numbers due to cell growth and division. From these results, we can estimate the rate of LacZ protein synthesis by a linear fit to the increasing LacZ concentrations during the first generation. Here, we obtained a rate of protein synthesis of 21*s*^-1 ^which is close to the 22*s*^-1 ^obtained by Kierzek et al. [30] and the 20*s*^-1 ^reported by Kennell and Riezman [37].

The LacZ model both in FernML and SBML format and the code for running the simulation is included with the FERN distribution along with several other models such as the model of the EGF signaling pathway by Lee et al. [38].

### Accuracy of stochastic simulation algorithms

To test the accuracy of the implemented stochastic simulation algorithms we used the Discrete Stochastic Models Test Suite (DSMTS) [39]. This test suite provides 36 stochastic models in the SBML format which have been solved either analytically or numerically. To test the implementation of a stochastic simulation algorithm, simulations have to be performed a large number of times (in general 10,000 times) for each individual model. The test is failed for a model if the distribution of the results is statistically significantly different from the known underlying distribution.

All three exact stochastic simulation algorithms in FERN were tested with the DSMTS test suite. Of the 36 models only the test 3.4 is failed. Models 1.10 and 1.11 are rejected because hasOnlySubstanceUnits is not declared to be true. If the rejection is overridden, Model 1.11 is failed, too. According to the DSMTS user guide, failure is expected for this model due to the inappropriate definition of the SBML model. In model 3.4, molecule numbers are reset whenever one molecule exceeds a certain number. This may lead to larger variations than accounted for by the thresholds used in the tests.

To asses our results we also evaluated gillespie2, the stochastic simulation program by some of the authors of the test suite [29]. Since the version of gillespie2 available online does not support level 2, version 3 of SBML, only 33 of the 36 models could be evaluated. We found that tests for model 1.11 and 3.4 are also failed, as well as tests for models 1.3, 1.14, 3.6 and 3.7. Two other models (1.17 and 1.18) could not be simulated as the rate law definition was not accepted by the program. Furthermore, we compared the runtime of FERN and gillespie2 on the DSMTS models which could be run by both programs and found that the runtime of FERN was significantly less than the runtime of gillespie2 (see Additional file 3).

### Runtime performance

Runtime performance of the exact SSA implementations of FERN was compared against the performance of the Gillespie and Gibson-Bruck algorithms of ISBJava and the Gillespie algorithm of gillespie2. For this purpose, simulations were performed for the EGF signaling pathway by Lee et al. [38] which contains 39 molecular species and 19 reversible and 12 irreversible reactions. For each implementation, 1,000 simulations were performed for a simulated time of 800 seconds and results were obtained for the activated enzymes of the signaling cascade (see Figure 5 and Additional file 3).

Our results show that the implementations of the original Gillespie and Gibson-Bruck algorithm of FERN are always more efficient for the FernML network than the implementations provided by ISBJava. For the SBML network, the performance is similar for the Gibson-Bruck simulator but significantly worse for the original Gillespie algorithm. This is due to the evaluation of the MathML expressions required at each step of the simulation for each molecular species. However, this allows for more complex definitions of kinetic laws than possible in ISBJava which supports SBML only up to level 1, version 2 without MathML. If we compare FERN's implementation of the Gillespie algorithm to gillespie2 which also supports MathML, we observe that FERN is more than three times faster than gillespie2 on the SBML network.

Furthermore, the enhanced implementation of the Gillespie algorithm provided by FERN is more efficient both for FernML and SBML than any of the exact methods provided by ISBJava. This shows that the powerful observer system of FERN does not come at the cost of a reduced runtime performance. Accordingly, FERN is a useful library for stochastic simulation even if the observer tools are not used.

## Discussion

In this article, we presented FERN, a Java framework for modeling and simulating biological reaction networks. FERN is subdivided into three layers which are represented by either one interface or abstract class. The functionalities of the package are then provided by implementations of these classes. Accordingly, FERN can be easily extended. For instance, any network class can use the algorithms of FERN by implementing the *Network *interface. New simulation algorithms can be implemented easily by overriding only a few methods of the abstract *Simulator *class and filling them with the new functionality. In this way, arbitrary FERN-readable networks can be simulated in different ways and the presentation system can be exploited.

It is possible to do reasonable simulations with FERN in just five lines of Java code. Each of the five steps can be expanded to cover more complex scenarios and simulations can be controlled at different levels. For instance, to simulate cell growth, an observer can be modified to change the volume of the simulation space. Alternatively, an interesting subnetwork can be selected on which simulations can then be run. FERN can be easily integrated into other applications making its functionalities available within different environments. We have illustrated this by implementing FERN plugins to Cytoscape and CellDesigner. With only few additional classes, the Cytoscape plugin enables the users to follow the simulation progress directly on the network. This was made possible by the powerful observer system provided by FERN which is one of its major advantages compared to other available simulation programs.

The accuracy of our SSA implementations was analyzed by applying them to the Discrete Stochastic Models Test Suite. All three of the exact simulation algorithms passed 94.4% of the DSMTS models which is significantly better than the performance of gillespie2 which passes only 80.6% of the tests. This shows that the SSAs provided by FERN are highly accurate as well as fast. Even though FERN is implemented in Java which is often claimed to be less efficient than C, FERN's original gillespie algorithm is significantly faster than the C implementation of gillespie2 (see Additional file 3).

Compared with the ISBJava library, FERN has several advantages. First, FERN is more flexible than ISBJava and offers more functions for tracking and interacting with simulations. Second, it implements both a hybrid algorithm as well as the most current tau-leaping methods which resolve the problem of negative concentrations. Furthermore, its stochastic simulation algorithms are significantly faster than the ISBJava implementations. Finally, it supports the current version of SBML and allows arbitrary rate laws.

## Conclusion

FERN is an easy-to-use framework for modeling and simulating reaction networks and can be easily integrated into other systems biology applications implemented in Java. It provides state-of-the-art stochastic simulation algorithms, efficient representations of networks with several input and output options and various ways of tracing and visualizing simulation data. Although some available stochastic simulation programs offer a few specialized features not yet supported by FERN such as e.g. time-delayed dynamics, none of them offer such a wide range of features and can be extended to new features as easily as FERN. Thus, FERN is a useful tool for biochemical network analysis or the development of new analysis methods or applications.

## Availability and requirements

• Project name: FERN

• Project home page: 

• Operating system(s): Platform independent

• Programming language: Java

• Other requirements: Java 1.5 or higher; Colt package ; JDOM 

• Optional: libSBML  for SBML version 2 level 1–3 support; Cytoscape 2.4.0 or higher  to use the Cytoscape plugin; CellDesigner 4.0 beta or higher  to use the CellDesigner plugin; gnuplot .

• License: FERN is freely available under the GNU Lesser General Public License (LGPL).

## Authors' contributions

EF designed and implemented the framework. CCF helped with the design and coordinated the project. EF and CCF drafted the manuscript. RZ provided advice and guidance for the project and helped to revise the manuscript. All authors read and approved the final manuscript.

## Supplementary Material

Additional file 1 FERN distribution, Version 1.3. This archive contains the FERN source code and binaries as well as documentation and example models in FernML and SBML. Click here for file

Additional file 2 FERN user guide. The user guide provides instructions on installing and using FERN, as well as a description of the software architecture and a specification of FernML and supported features of SBML. Click here for file

Additional file 3 Supplementary Figures. This file contains Supplementary Figures on FernML, the simulation cycle, the Cytoscape plugin and runtime comparisons between FERN and gillespie2. Click here for file
