# Supplementary material for: FERN – a Java framework for stochastic simulation and evaluation of reaction networks
Source: BMC Bioinformatics. 2008 Aug 29;9:356. doi: 10.1186/1471-2105-9-356 (PMC2553347; doi:10.1186/1471-2105-9-356)
Supplement: Additional file 1 — FERN distribution, Version 1.3. This archive contains the FERN source code and binaries as well as documentation and example models in FernML and SBML. [file 1471-2105-9-356-S1.zip › fern/doc/cytoscape.pdf]

FERN

Framework for Evaluation of chemical Reaction Networks

Cytoscape plugin

written in java

by Florian Erhard, Institut für Informatik, Ludwig-Maximilians-Universität München,
Amalienstraße 17, 80333 München, Germany

Contents

1 Cytoscape **4**

2 Requirements **4**

3 Example session **5**

4 User interface - features **11**

4.1 Main window 11

4.2 Color window 12

4.3 Plot window 12

5 Plotting trend curves **12**

1 Cytoscape

(from www.cytoscape.org):
Cytoscape is a bioinformatics software platform for visualizing molecular interaction networks and integrating these interactions with gene expression profiles and other state data. Additional features are available as plugins. Plugins are available for network and molecular profiling analyses, new layouts, additional file format support and connection with databases. Plugins may be developed using the Cytoscape open Java software architecture by anyone and plugin community development is encouraged.

An additional feature could be visualizing the simulation of a reaction network. This is provided by the FERN cytoscape plugin (for more information about FERN please refer to the FERN documentation available at http://www.bio.ifi.lmu.de/fern).

2 Requirements

There are only two basic requirements for *running* the plugin:

- An actual version of Cytoscape (we tested it with version 2.4.0)
- The fern.jar file in the *cytoscape/plugin* folder

However, if you plan to *use* the plugin with a certain network, the network must satisfy following restrictions:

- Reaction and species nodes must be distinguishable: Since a reaction network is basically a petrinet with species as places and reactions as transitions, there must be an attribute for each node to distinguish reactions and species.
- Edges are only allowed between reaction nodes and species nodes (so the network is a bipartite graph).
- The direction of edges (is the species a reactant or a product of the connected reaction?) must be identifiable (Either the edge identifier contains *reactant* or *product*, as it is given by the cytoscape sbml plugin or the edges are directed from reactant to reaction and from reaction to product).
- For each reaction node there must be an attribute containing the reaction coefficient, for each species one containing the initial amount.

These attributes can be specified in the plugin's main window. If you use the cytoscape sbml reader, the plugin should automatically determine the attributes (and of course, if you load a fernml network from within the plugin, this will work automatically as well).

Additionally, if you want to produce trend curves, make sure you have gnuplot installed and it is accessible (e.g. the binary's path is within you PATH environment variable).

3 Example session

Let us start with an example of using the fern plugin. You will see how to load a fernml network, how to simulate it and visualize the progress and how to produce trend curves of some species.

Start cytoscape and activate the plugin.

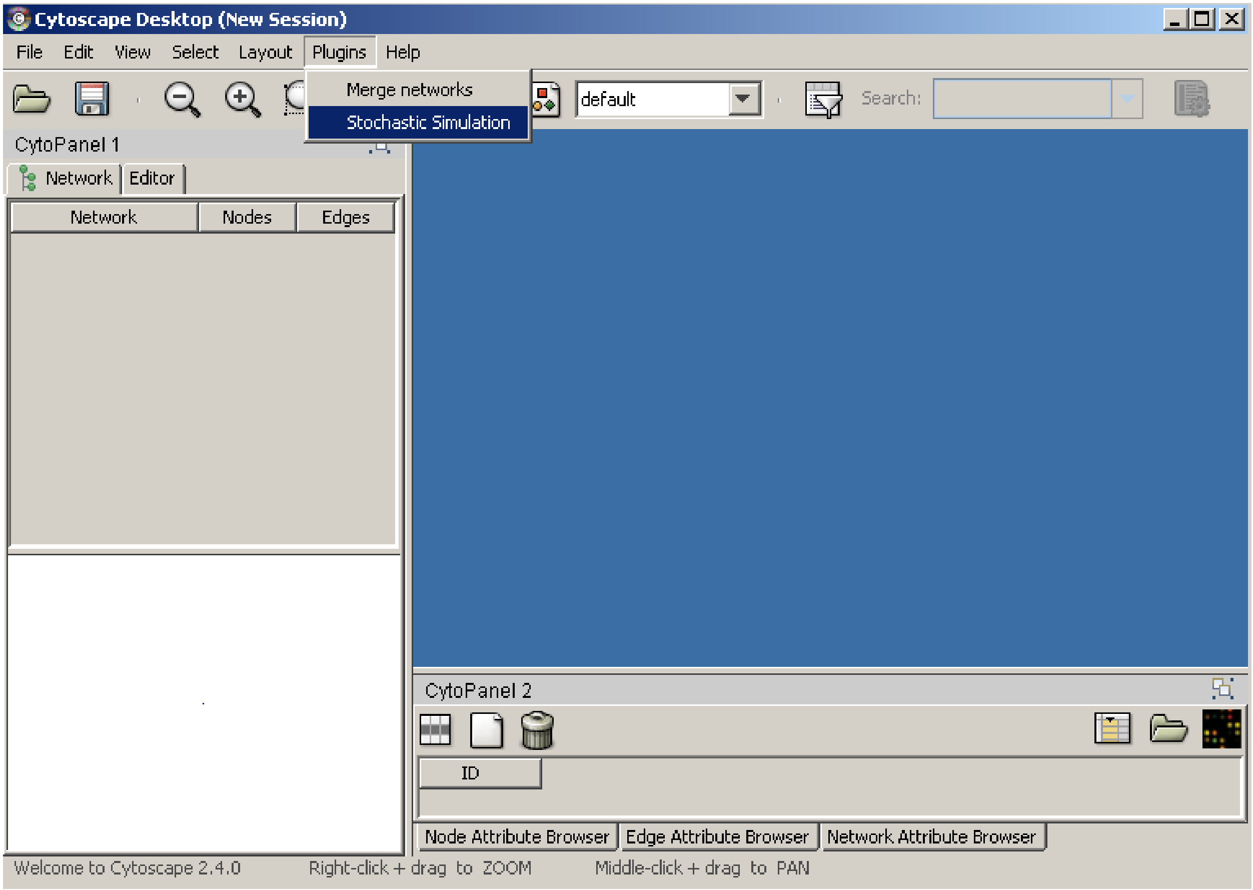

Figure 1: Cytoscape plugin example - step 1

Click the *load* button in the upper left corner of the plugin window and choose a fernml file from your filesystem (there are some examples included in the fern package - let us use *mapk.xml*).

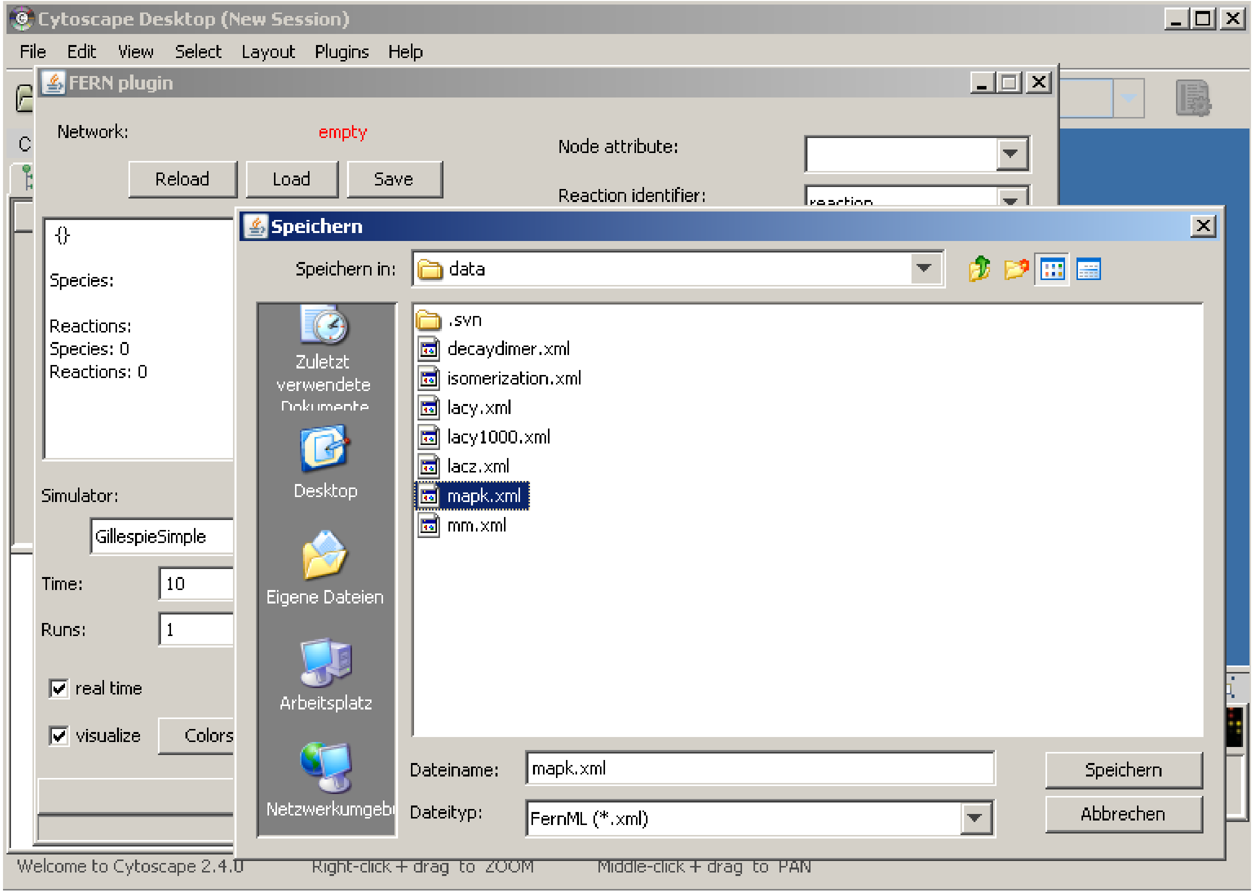

Figure 2: Cytoscape plugin example - step 2

Now we can see that the network is loaded correctly, but since fernml does not specify a graph layout, let us beautify the view and pick an automatic layout (e.g. the organic one from the yFiles plugin).

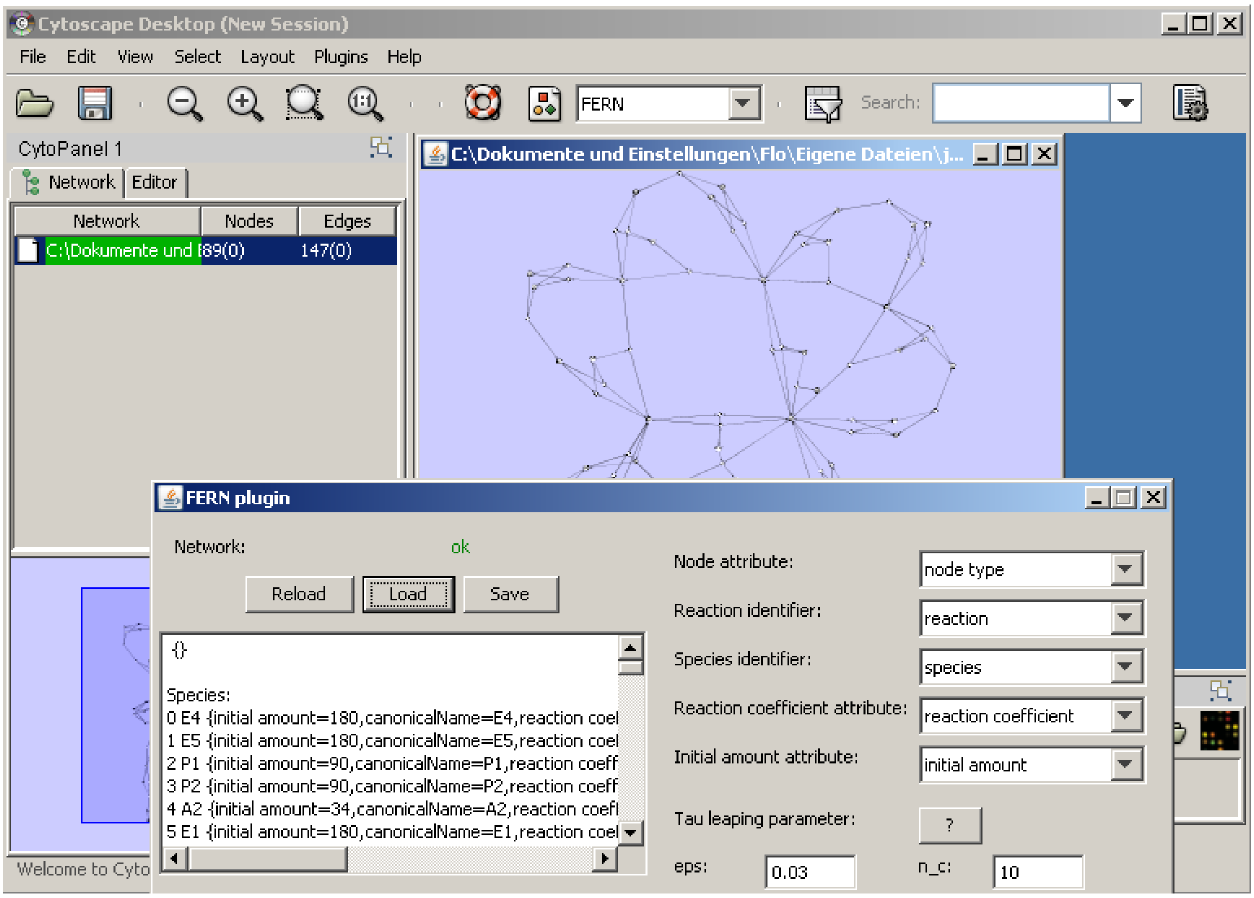

Figure 3: Cytoscape plugin example - step 3

Since the interesting dynamics of this network emerge during a time period of 1000 units [1], we change this value. Now we can start the simulation with everything else set to default (don't forget to look at the cytoscape main window!).

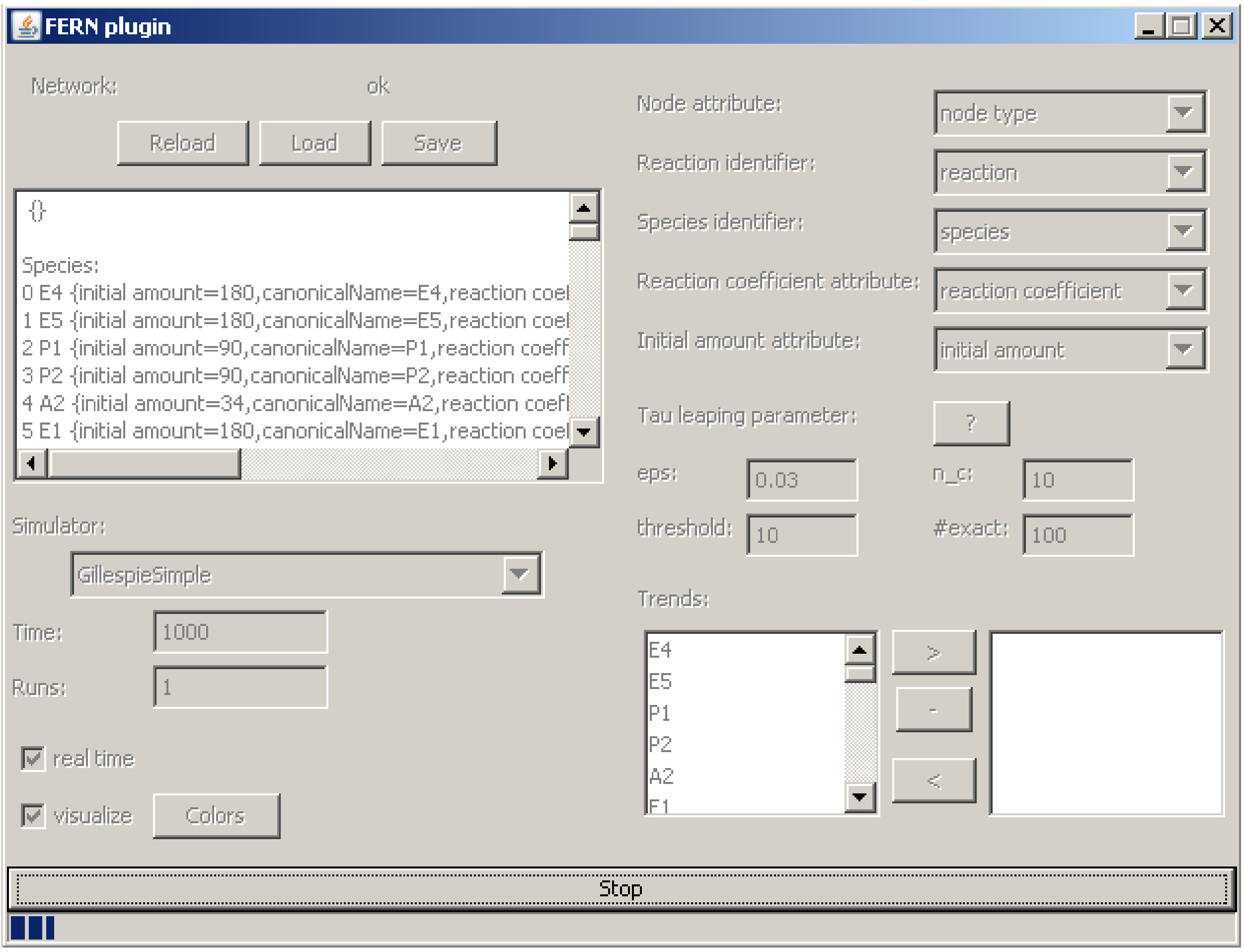

Figure 4: Cytoscape plugin example - step 4

Because we had *real time* checked, the simulation will last at least 1000 seconds. Because we don't want to wait so long, we stop the simulation. Now let us produce some nice trend curves. Uncheck *real time* and *visualize* and move the *E0** to *E5** to the right of the two lists in the lower right corner. We want an average trend of 100 runs, so we fill in that value. Now we can restart the simulation.

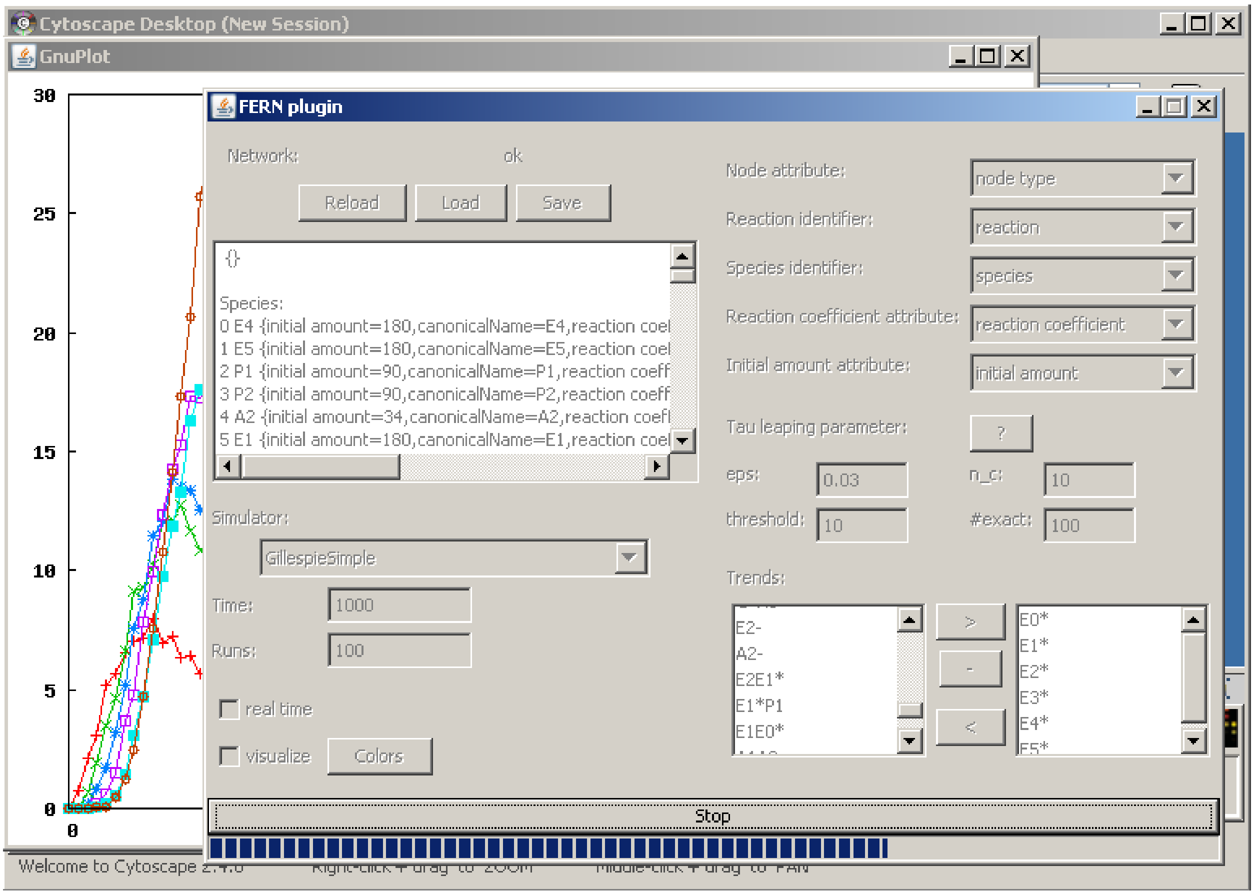

Figure 5: Cytoscape plugin example - step 5

If you want to save the plot, you simply have to click onto it.

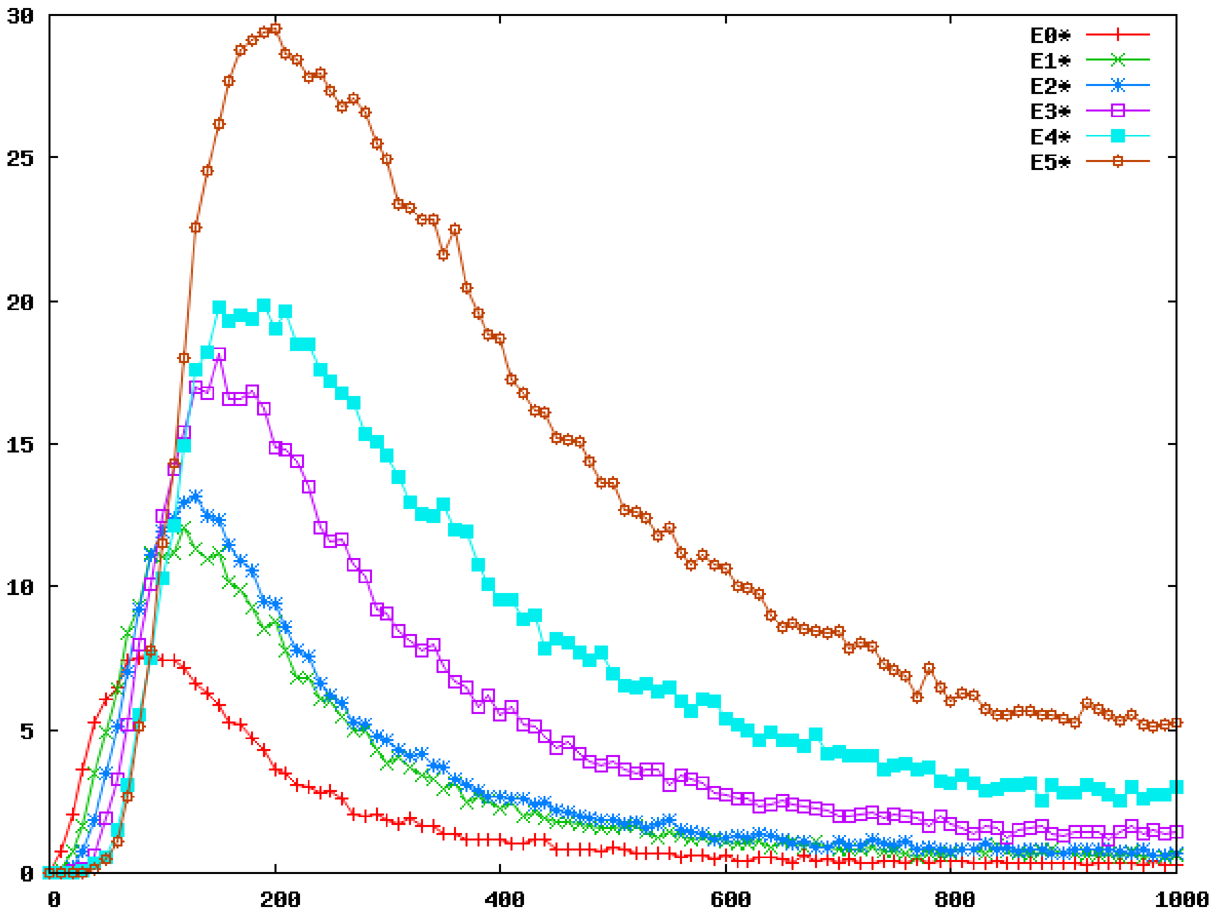

Figure 6: Cytoscape plugin example - step 6

4 User interface - features

4.1 Main window

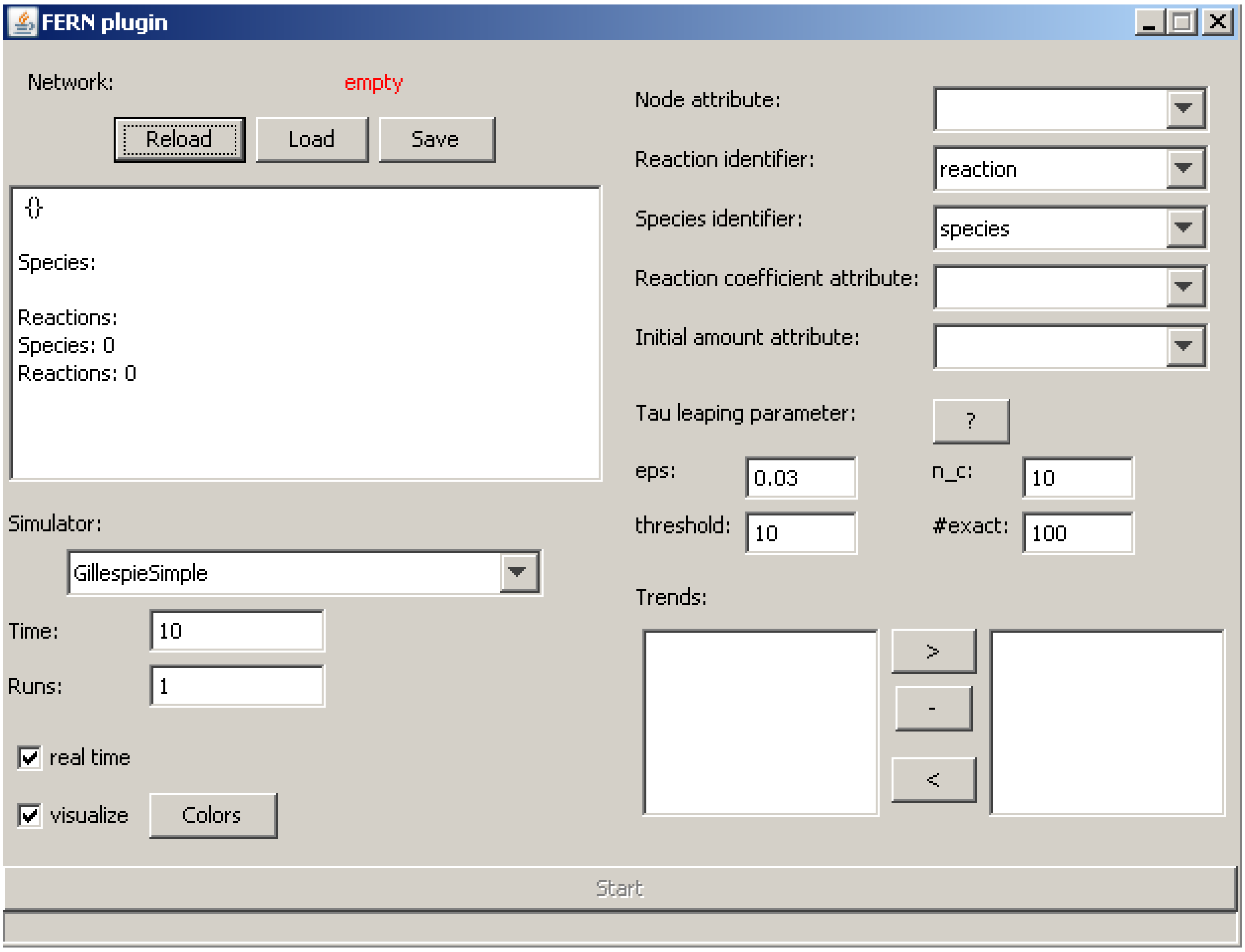

Figure 7: Cytoscape plugin main window

In figure 7 the main window of the cytoscape plugin is shown. It is composed of several elements (from top to bottom and left to right):

- Network: Indicates wheter the actual loaded network can be used for simulation (if not, some error message is displayed in the textbox).
  - Reload: Try to load the network actually displayed in cytoscape into the plugin.
  - Load; Load a fernml network from the filesystem.

  - Save: Save the actual loaded network as fernml file.

- Simulator: Choose one of the built-in simulation algorithms. For more information refer to the fern documentation or to [2, 3, 4, 5, 6].
- Time: Define the timespan to simulate (depends on your reaction network).
- Runs: If you want to automatically repeat the simulation, you can specify here the number of repeats (a non positive value leads to infite repeats).
- real time: If you check this, the algorithm will pause between to reaction firings the simulated amount of time. This means that the simulation will take at least as much time as you defined in *time* and it would take exactly the time you defined if your computer was infinetly fast (and hence can run the algorithm and draw the network without any extra time).
- visualize: If you check this, the simulation progress is shown directly in the cytoscape main window. Each firing of a reaction is indicated by a blinking of the corresponding reaction node and the amounts of each molecular species is indicated by coloring the corresponding species node.
- Attributes: As mentioned above, the network must fulfill some requirements by setting some attributes. The names of these attributes have to be specified here.
- Tau leaping parameters: For the three tau leaping algorithms you can specify some parameters controlling the behaviour / performance (for more information refer to [6]:
  - eps: error bounding
  - n_c: threshold for critical reactions
  - threshold: for temporarily abandoning tau leaping
  - #exact: how many exact SSA steps when abandoning tau leaping
- Trends: In the left listbox the names of each molecular species of the loaded network is shown. You can move them to the right box (and of course back) by selecting them and clicking the appropriate button. For each species in the left box a trend will be created when you simulate the network. If you want several plots, you can separate species by using the - button.

4.2 Color window

If you want to visualize the simulation progress in the cytoscape main window, you can specify the colors by clicking the colors button in the main window which yields to a window shown in figure 8.

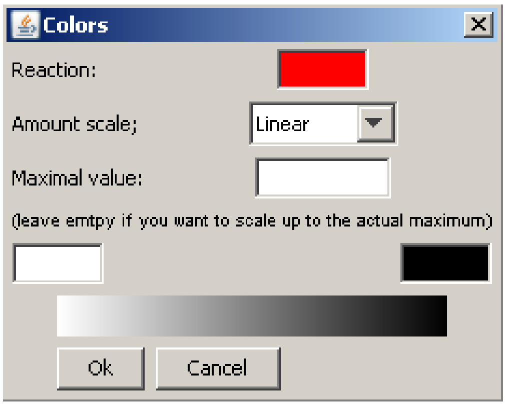

Figure 8: Cytoscape plugin - color selection window

As mentioned above, the reaction nodes will blink in the specified color and the reaction nodes will have a color corresponding to their actual amount.

4.3 Plot window

If you create plots, a window as shown in figure 9 pops up.

If you want to save this image, just click onto it or press F3 or CTRL-S. Supported formats are ps and png.

5 Plotting trend curves

As described in previous sections, it is fairy easy to create trend curves of some molecular species. There are some issues to consider:

- It will only work if gnuplot is installed. Additionally make sure it is accessible from the Cytoscape startup path (e.g. puth it into the path variable or create a link named gnuplot).
- For each species and each simulation run, exactly 100 reading points are recorded (e.g. if you simulated up to a time of 1000, every 10 time units the amount of eachs species is recorded).
- Every time you click on Start, a new plot is created.

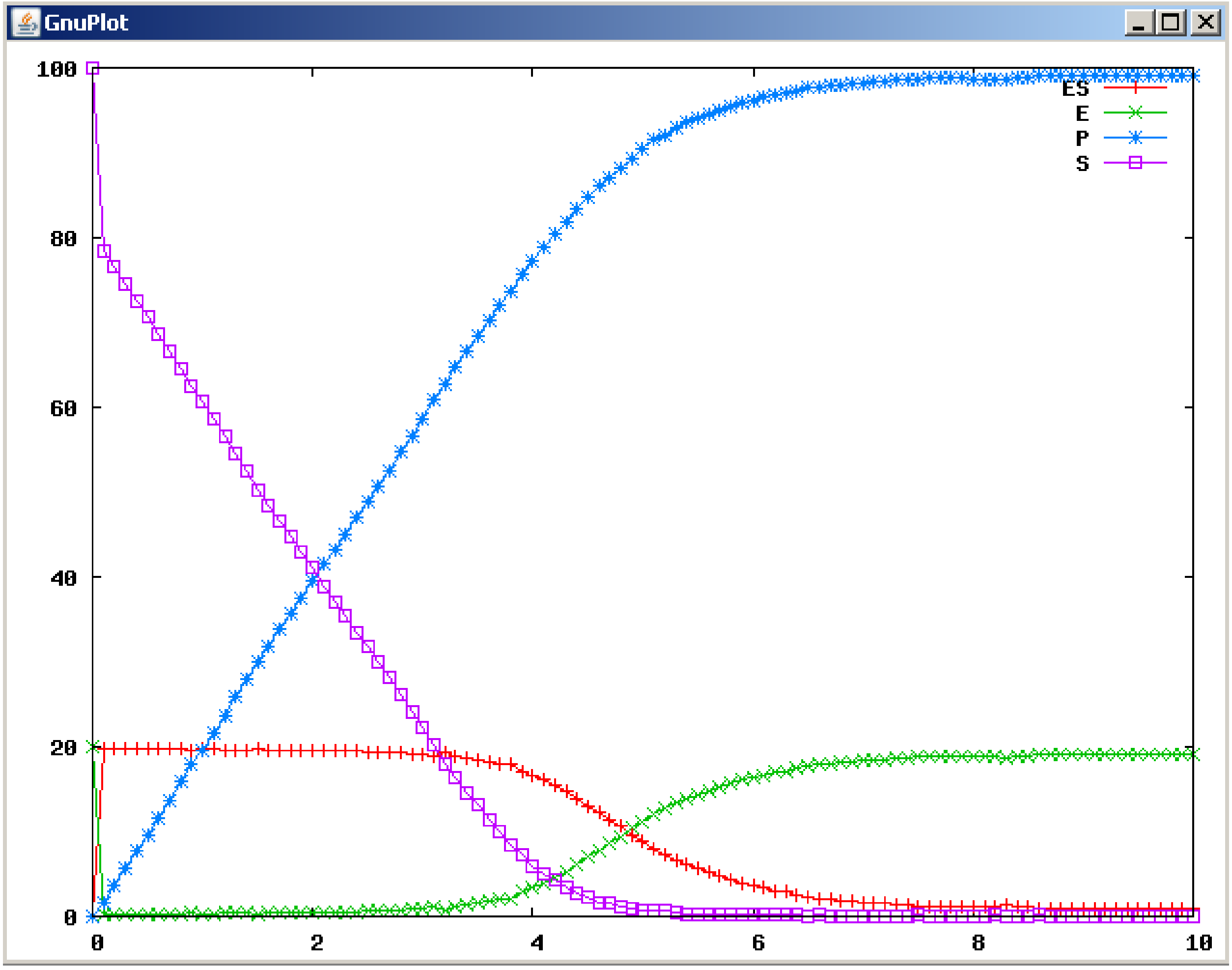

Figure 9: Cytoscape plugin - gnuplot window

- If you specify more than one run, after each run the actual average of each trend is calculated and shown.
- If you perform a real time simulation, the progress is shown online and the plot is created everytime the simulation crosses a reading point.

References

[1] Lee et al., Colored petri net modeling and simulation of signal transduction pathways.

[2] Gillespie D.T., J. Comput. Phys. 22, 403 (1976)

[3] Gibson M.A. and Bruck J., J. Phys. Chem. 104, 1876 (2000)

[4] Gillespie D.T., J. Phys. Chem. 115, 1716 (2001)

[5] Gillespie D.T. and Petzold L., J. Chem. Phys. 119, 8229 (2003)

[6] Cao Y., J. Chem. Phys. 124, 044109 (2006)
