# Supplementary material for: FERN – a Java framework for stochastic simulation and evaluation of reaction networks
Source: BMC Bioinformatics. 2008 Aug 29;9:356. doi: 10.1186/1471-2105-9-356 (PMC2553347; doi:10.1186/1471-2105-9-356)
Supplement: Additional file 1 — FERN distribution, Version 1.3. This archive contains the FERN source code and binaries as well as documentation and example models in FernML and SBML. [file 1471-2105-9-356-S1.zip › fern/doc/guide.pdf]

FERN

a Framework for the Evaluation of chemical Reaction Networks

written in java

by Florian Erhard, Institut für Informatik, Ludwig-Maximilians-Universität München,
Amalienstraße 17, 80333 München, Germany

Contents

1 Overview **4**

2 Installation and first steps **4**

2.1 Windows users 5

2.2 Eclipse or other IDEs 5

3 File formats **6**

3.1 FernML 6

3.2 SBML 7

4 Framework structure **8**

4.1 Network 9

4.2 Simulator 11

4.3 Observer 13

5 Gnuplot **16**

6 Analysis **16**

7 Stochastics **17**

8 Examples **17**

8.1 MichaelisMentenKinetic 18

8.2 DecayingDimerizing 18

8.3 IrreversibleIzomerization 18

8.4 LacZ/LacY 19

8.5 MapkBenchmark 19

8.6 AutocatalyticNetworkExample 19

8.7 Dsmts 19
8.8 start.sh 20

A FernML example 21

B FernML specifications 22

C SBML support 23

1 Overview

FERN is a fast, extensible and comprehensive framework for the stochastic simulation and analysis of chemical reaction networks. A chemical reaction network is composed of several molecular species coupled by reaction channels. The goal of stochastic simulation algorithms (SSAs) is to simulate a trajectory of the species' populations by a Monte Carlo Markov Chain. Starting from a vector of initial amounts for each species, the SSA updates these values by firing reaction channels accordingly to a distribution given by their propensities. Additionally it calculates the time between two firings and hence provides a time scale for the events. For a broader view about the theory of SSAs please refer to [2, 4, 8]. This guide is intended to be a complete description of the basic FERN package. The documentation for the Cytoscape plugin is separately available at http://www.bio.ifi.lmu.de/FERN. The Cytoscape plugin was also adapted as a plugin to CellDesigner [13] which offers a plug-in functionality with the recent version 4.0 beta.

2 Installation and first steps

If you do not already have Java installed on your computer (or you do not have a current version - try to type *java -version* into a console), you will have to download the JDK from http://java.sun.com/javase/downloads/index.jsp. Then you just have to follow the installation instructions.

The installation process of FERN itself is also quite easy: Just download either the zip or the tar.gz file from our website and unpack it to some directory. Now the basic FERN package is ready to use. You can try it by typing either

```
./start.sh examples/mm.xml 5 1,
./start_cygwin.sh examples/mm.xml 5 1 or
start.bat examples\mm.xml 5 1
```

into your console. Depending on your environment, choose one of start.sh, start.bat or start_cygwin.sh for linux/unix/mac, windows or windows with cygwin, respectively. You should get 5 lines characterizing a trajectory of a simple Michaelis-Menten kinetics network.

If you have access to gnuplot (i.e. it starts if you type gnuplot into your console), the following line should give you a neat impression of FERN's abilities:

```
./start.sh examples/mm.xml 7 0.1 -n 25 -i,
./start_cygwin.sh examples/mm.xml 7 0.1 -n 25 -i or
start.bat examples\mm.xml 7 0.1 -n 25 -i
```

If you want to read SBML files, you have to get the newest libsbml. There you will also find comprehensive documentation about compiling and installing libsbml. Running windows, you simply have to download the precompiled package and copy the files of the subdirectory bindings/java to your FERN directory. Under linux/unix, you have to compile from source which is well documented at the libsbml website. Do not forget to set the –with-java flag for configure and to set the LD_LIBRARY_PATH variable. Then you just have to copy the sbmlj.jar to your FERN directory. Now the line

```
./start.sh examples/mm_sbml.xml 7 0.1 -n 25 -i,
./start_cygwin.sh examples/mm_sbml.xml 7 0.1 -n 25 -i or
start.bat examples\mm_sbml.xml 7 0.1 -n 25 -i
```

is supposed to produce the same results as the line above.

2.1 Windows users

Since windows users are usually not familiar with a command line console, here some first steps:

To start the console, use Start - Run (or just the search field under vista) to type

cmd

and press Enter. Now you can navigate to you FERN installation by typing

cd "C:\Program Files\fern"

if you unpacked FERN into that folder. Once you are within this folder, you can start FERN by using the start.bat as described above.

2.2 Eclipse or other IDEs

If you want to use FERN with Eclipse or another IDE of your choice, you only have to include the external jars colt, concurrent, jdom and fern in your project. Try to create a class Test with a main method and insert following code:

```java
Network net = new FernMLNetwork(new File("some_net.xml"));
NetworkTools.dumpNetwork(net);
```

You should get information about the network in some_net.xml. If you have problems running this simple test, check your java runtime environment and if necessary update it to the newest version.

Another possibility is to import the src directory of the FERN package into your project. Make sure you import the correct folder structure by checking if you have the item *fern* directly within your project in the Eclipse Package Explorer. If you just included colt, concurrent and jdom to your project, you will get compile errors in the packages *fern.network.sbml*, *fern.cytoscape* and *fern.cellDesigner*. In Eclipse, these will not affect other parts of FERN, so you can either ignore them or alternatively include sbmlj.jar, cytoscape.jar (available with the Cytoscape distribution, version 2.4.0. or higher) and CellDesigner.jar (get it from the newest version of CellDesigner - at least 4.0beta) into your project. Note that for some IDEs, compiling might fail completely if you don't delete the affected packages or include the jars.

3 File formats

FERN introduces its own file format, FernML. The advantage to existing file formats like SBML [11] is its simplicity. While in SBML you can model complex biological systems, FernML is supposed to describe just the reaction network of several molecular species. Since FERN uses the reaction rate equations described in [2], the propensities can calculated efficiently by a few arithmetic operations. SBML however uses MathML to store the kinetics of a reaction and hence it is neccessary to evaluate an expression tree every time a propensity is calculated (which is one of the essential steps of SSAs, together with the drawing of random numbers). This is significantly slower than direct multiplication, as shown in figure 1. These two formats can easily converted into each other by FERN:

```
Network net = new FernMLNetwork(new File("some_net.xml"));
SBMLNetwork sbml = new SBMLNetwork(net);
sbml.saveToFile(new File("some_net.sbml"'));
```

For converting a SBML network into a FernML network, the same holds, only Fern has to be exchanged with SB and vice versa. But note that in this case, only FernML specific features are saved, so a double conversion from SBML to FernML to SBML will yield a different SBML file.

3.1 FernML

A FernML document is a XML conform file and consists of

- a list of species definitions
- and a list of reaction definitions

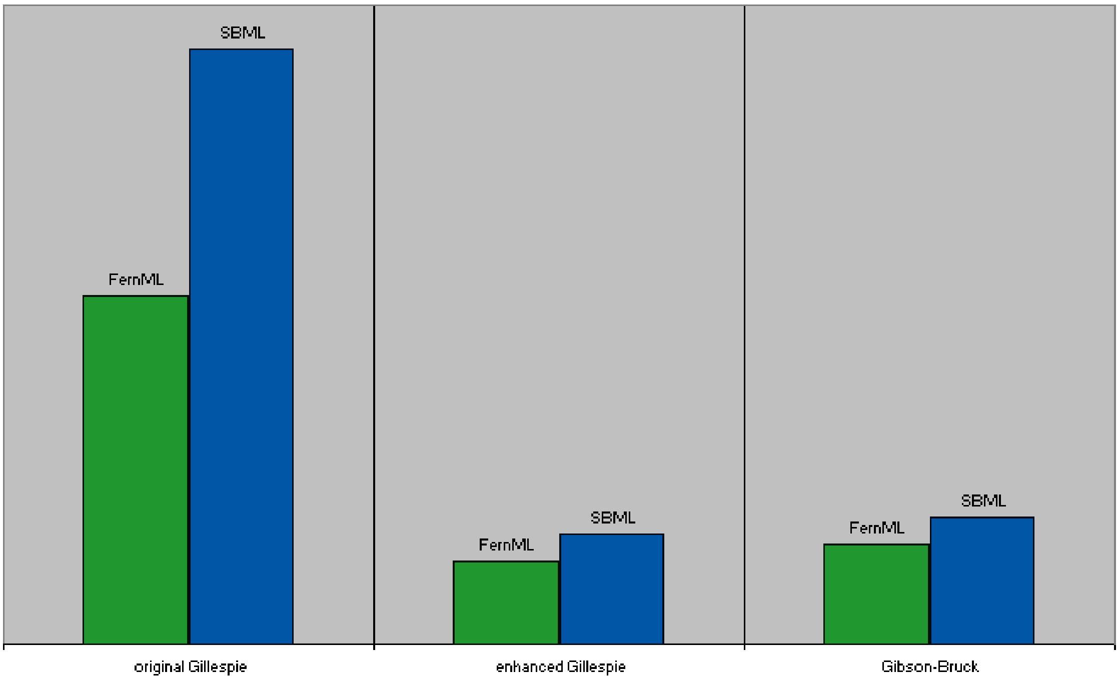

Figure 1: Relative time for simulating the MAPK example using FernML / SBML; the millseconds after 1000 runs are plotted for the three exact SSAs. As expected, the difference for the original Gillespie algorithm is the largest since it needs more propensity calculations than the other algorithms.

Each species definition has two attributes: its name and its initial amount. Each reaction definition has the mandatory attribute kineticConstant and the optional attribute kineticConstantReversible if the reaction is reversible. Additionally, each reaction element has to contain a <listOfReactants> and a <listOfProducts>, both consisting of species references. A ¡speciesReference> tag only contains the name of a previously defined species. For documentation purposes annotation elements can be attached to the root element and to species and reaction branches.

For further information see the appendix.

3.2 SBML

For information about SBML see [11] or at the website. Not each aspect of the current version (version 2 level 3) is supported, for a complete list see the appendix.

4 Framework structure

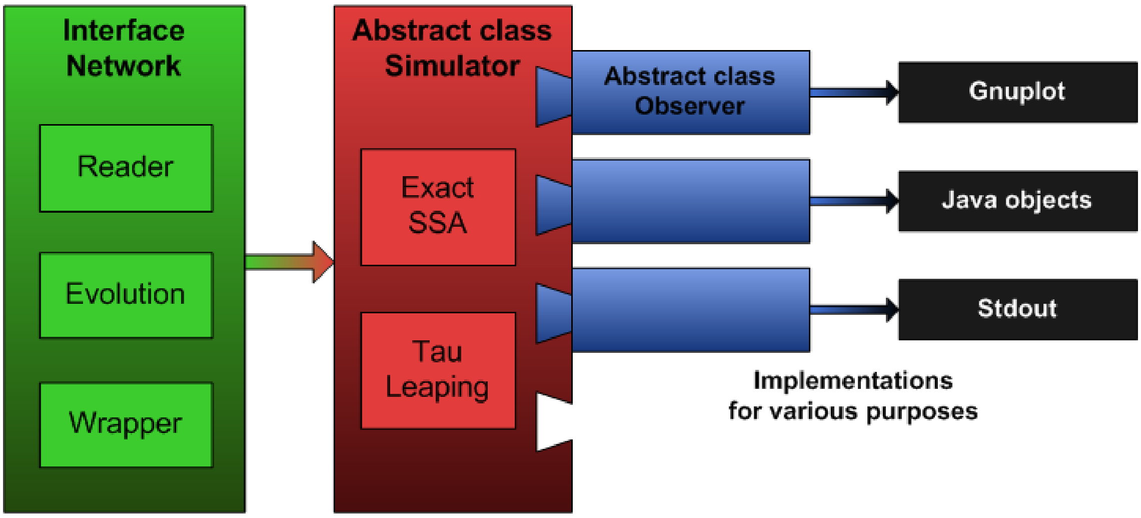

Figure 2: Structure of FERN: An application using FERN will always create Network objects, which can be instanciated by various implementations like file readers, evolution algorithms or wrappers of existing network network object. Usually these are passed to a Simulator, either one of the exact SSAs or a tau leaping procedure. A fundamental feature of FERN is the easy way of getting information out of simulations by registering Observer objects at the Simulator and the efficient processing of collected data.

For the simulation part of the framework there are three important interfaces / classes:

- Network: basic structure of a chemical reaction network
- Simulator: takes a network and performs a stochastic simulation
- Observer: observes certain aspects of a simulation and makes its results available

Network is an interface which can be implemented by network file readers (like FernMLNetwork, SBMLNetwork,), wrappers (like CytoscapeNetworkWrapper) or evolution algorithms (like AutocatalyticNetwork).

Simulator is an abstract class which provides certain functionalities for its extending classes (which contains just the simulation algorithms - like GillespieSimple, GibsonBruckSimulator) especially the observer system: You can register one to many Observer objects at the simulator. During a simulation run, specific methods of the observer are invoked at each step of the algorithm.

The **Observer** is an abstract class which provides several useful methods for extending classes and the abstract callback methods which are invoked by the Simulator. By different implementations of these, Observers are able to analyze several aspects of the simulation (e.g. changes of amounts of certain molecule species, firings of reactions). Several implementations are already available within the FERN distribution. These provide comprehensive functionalities like handling repeated simulation runs by calculating average data whenever it makes sense or introducing methods to pass data directly to gnuplot (please refer to the javadoc).

So, a very elementary example of using FERN could be:

```
1 Network net = new SBMLNetwork(new File("some_net.xml"));
2 Simulator sim = new GillespieEnhanced(net);
3 net.registerEvents(sim);
4 Observer obs = sim.addObserver(new
     AmountIntervalObserver(sim,1,"X"));
5 sim.start(50);
6 System.out.println(obs);
```

In (1) a network is loaded from a SBML file and in (2) a simulator is created. To ensure a proper SBML event handling, the events are registered (3) at the simulator (this is only neccessary, if events are defined within the SBML network - if you are not sure, just register them, it won't hurt - if there are no events to register, no events will be registered). In (4) an AmountIntervalObserver (which observes the molecule X repeatedly after an interval 1) is created and registered at the simulator. (5) starts the simulation algorithm and tells it to stop after 50 seconds of simulated time. Then (6) will print out the amount of each molecule in the array X for the timepoints 0, 1, 2, 3,...,50.

4.1 Network

The interface Network only stores the network's structure (together with some basic information like species names and their initial amounts). However each network object has to know more than that and has attached therefore three important classes as field variables:

- AmountManager: controls the amount of each molecular species during a simulation
- AnnotationManager: can store annotations for the network, its species and reactions
- PropensityCalculator: calculates propensities for reactions by some kinetic law

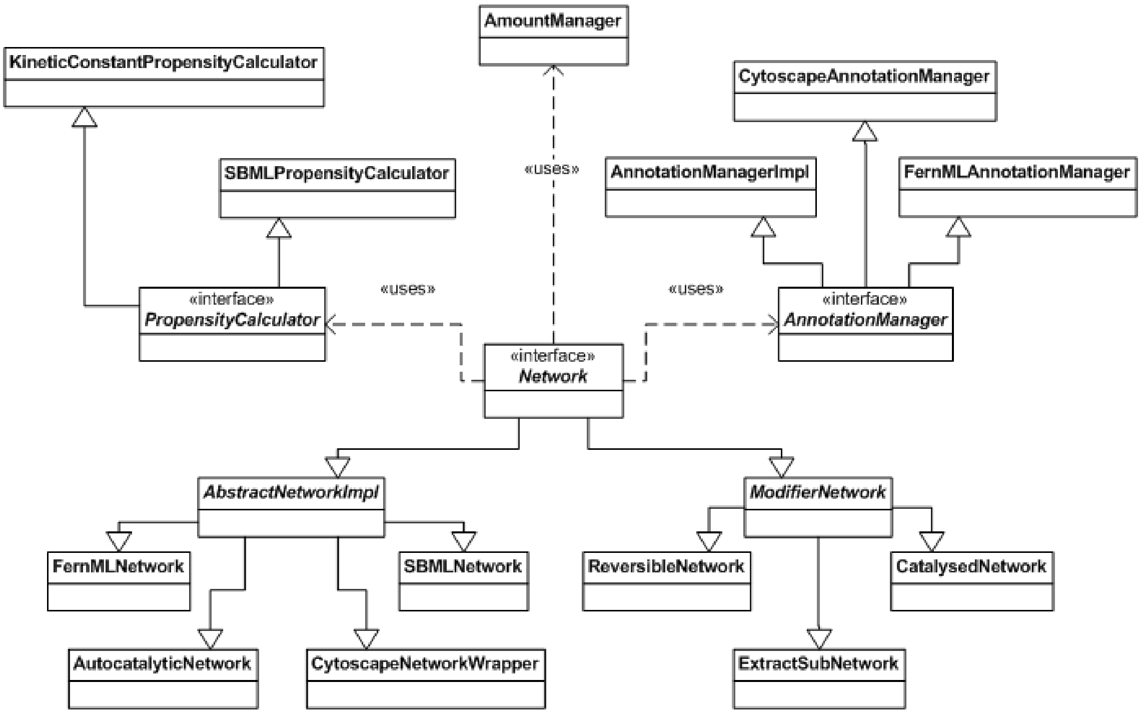

Figure 3: UML of Network related classes

In a network, each reaction and each species is basically a number between 0 and the number of species and reactions, respectively. So if you look for a class named Species or Reaction you won't find any. Each piece of information about species / reactions is stored in separate arrays and not in one object for each species / reaction.

Currently, there are various implementations of the network interface (refer to table 1). Once you have instantiated one of these Networks, you can:

- pass it to a Simulator object.
- pass it to an analysis algorithm.
- save it as FernML-file (by first creating a FernMLNetwork from it)
- save it as SBML-file (by first creating a SBMLNetwork from it)
- modify it.

In the package fern.network.modification are some implementations of the interface Network which take an existing Network and yield a modified version of it. For example the ReversibleNetwork virtually creates a reverse reaction to each reaction of the original network.

FernMLNetwork	Loads Networks in FernML. Supports only simple reaction rate kinetics.
SBMLNetwork	Loads Networks in SBML. Supports arbitrary rate laws in MathML. Does not support rules, constraints or function definitions
CytoscapeNetworkWrapper	Used by the Cytoscape plugin to adapt the cytoscape data structures to FERN and redirect function calls
CellDesignerNetworkWrapper	Used by the CellDesigner plugin to adapt the CellDesigner data structures to FERN and redirect function calls
AutocatalyticNetwork	Evolves a network form some starting monomers by aggregation / disaggregation reactions leading to polymers of customizable length

Table 1: Implementations of the network interface

"'Virtually creates"' means that the ModifierNetwork will not copy the network structure but rather redirect calls (for more information see the javadoc).

4.2 Simulator

Each built-in simulator follows the same scheme (see also figure 4):

- Initialize data structures
- As long as the passed SimulationController allows to go on:
  - Perform one step

The SimulationController is an interface which can tell the simulator whether to go on or not. Usually you will use the DefaultController which lets the simulation run until a given simulated time is crossed. During one step the algorithm will fire one or more reactions.

Afterwards, new amounts for the reactants and products are calculated by the network's AmountManager and new propensities for some reactions (which depends on the algorithm) by the PropensityCalculator (which itself needs the AmountManager to calculate these).

GillespieSimple	The direct method by Gillespie [2]
GibsonBruckSimulator	The next-reaction method by Gibson and Bruck [3]
GillespieEnhanced	Uses dependency graphs to improve the runtime of the original Gillespie algorithm
TauLeaping AbsoluteBoundSimulator	Tau-leaping algorithm. The error is bounded by the sum of all propensity functions [8]
TauLeaping RelativeBoundSimulator	Tau-leaping algorithm. The error is bounded by the relative change in the individual propensity functions [8]
TauLeaping SpeciesPopulationBoundSimulator	Tau-leaping algorithm. The error is bounded by the relative changes in the molecular populations [8]
HybridMaximalTimeStep	Maximal time step method by Puchalka [12]. Automatic paritioning into slow and fast reactions, which are fired according to an exact and tau leaping method, respectively.

Table 2: Included simulation algorithms

In FERN, current state-of-the-art algorithms are included (look at table 2: Three of them are exact algorithms (which means that they create the Markov trajectory reaction by reaction); the threww TauLeaping procedure are approximate algorithms which should be faster (but not for every type of network) but the produced results are not exact (if you can call the results of Monte Carlo algorithms exact - more accurate would be "do not exactly match the correct distributions").

The tau-leaping algorithms are all based on the modified tau-leaping procedure proposed by Cao et al. [8] which avoids the problem of negative populations observed for the original tau-leaping procedure. Basically, the three implementations differ only in the way the

error is bound (see [9] for details). The error is bounded either by the sum of all propensitiy functions (*TauLeapingAbsoluteBoundSimulator*), the relative change in the individual propensity functions (*TauLeapingRelativeBoundSimulator*) or the relative changes in the molecular populations (*TauLeapingSpeciesPopulationBoundSimulator*)

The hybrid method by Puchalka and Kierzek [12] dynamically partitions the system during the simulation into "slow" reactions which involve only small molecule numbers and "fast" reactions which involve large molecule numbers. The slow reactions are then simulated using an exact SSA while the fast reactions are simulated with approximate tau-leaping. Our implementation of the hybrid method uses the more efficient enhanced Gillespie algorithm instead of the Gibson and Bruck algorithm proposed by Puchalka and Kierzek.

You can register a PrintWriter object to a simulator. Once you do this, the string representation of each observer is printed when a simulation has finished.

If you have to simulate reactions networks with triggers (e.g. a cell division divides each species population by 2 at some moment, cell growth...) you can implement your own observer and invoke setAmount(species) or setVolume(volume) of the Simulator object. In this case, you should consider that each propensity has to be recalculated (which is done automatically by each implemented simulator). GillespieEnhanced and even more GibsonBruck will be slower than GillespieSimple whenever you invoke one of these methods!

4.3 Observer

As mentioned before, the framework uses an observer system to reflect the progress of the simulation. Once a observer is registered at a simulator and the simulator is started, it will invoke several methods of the observer during a simulation (see table 3 and figure 4).

The methods start, finished, step and activateReaction are handled by the simulator base class, theta has to be invoked by the implementing algorithms, since its invocation depends heavily on the algorithm (if you want to implement your own, keep that in mind). If an Observer wants to called at a specific timepoint during a simulation (e.g. in order to record amounts of molecular species), it can register this timepoint θ at the simulator. If θ is crossed, the method *thetaEvent* is invoked by the Simulator and the corresponding observer can react (e.g. record the amount of some species). This task could also be handled by the method *step* and checking for the timepoint. We suggest to prefer the theta method because the step method does not guarantee a complete record when the simulation algorithm leaps across several reading points.

There are several different types of observers, some can handle repeated simulations and present average results, some cannot, some produce gnuplot data some do not. For more information refer to to table 4 and the *fern.simulaton.observer* package in the javadoc.

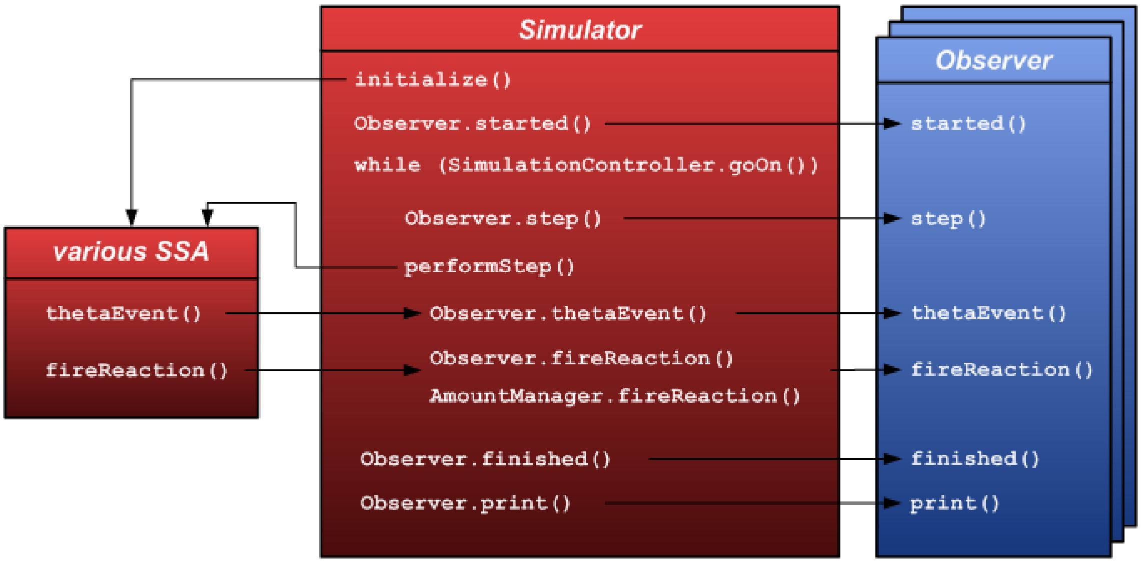

Figure 4: Simulation cycle and when Observer methods are invoked

start	invoked after initialization of the algorithm to give the observer the chance to (re)initialize itself
finished	invoked right after the SimulationController stops the simulation. Observers can clean up resources, for instance.
step	invoked every time after the SimulationController tells to go on and before the simulation algorithm fires reaction(s)
activateReaction	invoked when a reaction channel fires (but before the amounts are changed)
theta	invoked when a certain timepoint is crossed; the specific timepoint is defined by calling *Simulator.registerTheta*

Table 3: Observer methods

AmountIntervalObserver	The probably most prominent observer, tracks the amount of molecules repeatedly after intervals. Can handle repeated simulations and will then return averaged trends.
MultiAmountIntervalObserver	Similarly as the AmountIntervalObserver, but instead of recording the amount of the species individually, it records the sum of the specified molecules. This is useful if you have to split a logical entity such as mRNA into e.g. mRnaFree and mRnaDuringTranslation, but you want to record the total amount of mRna.
ReactionIntervalObserver	Similarly as the AmountIntervalObserver, but instead of recording the amount of molecular species, it records firings of reactions.
AmountAtMomentObserver	Record the amount of some molecule species at one certain moment. When simulations are repeated, it calculates histograms for that moment and for each species.
RandomNumberGeneratorCallObs	Records, what kind of random numbers are drawn during simulation. Since efficient random number generation is important for stochastic simulation, this observer can be useful for performance measurements.
FireTypeObserver	Track the types of firings (exact, tau leaping), which can be interesting when using the tau leaping procedures (when they avoid negative amounts, they use some exact steps [8], this can be a performance bottleneck for these algorithms) or of course the hybrid algorithm.
InstantOutputObserver	Prints out every event that the simulatr reports to the observer.

Table 4: Built-in observers

5 Gnuplot

As mentioned above, some observers produce gnuplot data. You can use this data directly from java via the GnuPlot class. The observers provide a method toGnuplot, which adds the current data (either average data or recent data) to a gnuplot object. With a gnuplot object, you can:

- retrieve the gnuplot data as String or save it to a file
- get the plot command
- plot the data by calling gnuplot from java
- retrieve the plot as image object or save it to a png file
- show the plot in a JFrame

It is possible to add custom gnuplot commands to change the look of the plot as well as to pass additional styles for the plot command (for more details refer to the javadoc and the gnuplot documentation).

The data is managed by Axes objects. Each gnuplot object has a list of Axes objects, each Axes object can have a list of additional Axes objects and each Axes objects has one to many columns. Thus data can be combined in many different ways:

- add it to different gnuplot objects: different data files and different plots are created (e.g. one observer records the amount of some species, another one the step sizes of the leaps, and you want different plots)
- add it to the same gnuplot object as separate Axes objects: different data files are created but only one plot (if you want the amount trend and the step sizes in one plot)
- add one Axes object to another one: only one data file and hence one plot is created (this is only possible, if the number of rows is equal; it is reasonable, if you have already a Axes object containing a x axis and some value axis and you want to add just another value axis)

6 Analysis

If you want to take a look at your networks analytically (e.g. perform algorithms based on a breadth/depth first search), you should use the AnalysisBase class. Since the network

class only knows the neighbourhood of reactions and not of species (which is not necessary for simulations), it creates the corresponding index structures for you and provides generic implementations of breadth/depth first searches (for customization refer to the javadoc).

Two analysis algorithms are included in the framework:

- Shortest paths: By using the bfs the shortest paths starting from some source species to each other species is computed.
- AutocatalyticNetworkDetection: By using two modified bfs the autocatalytic subset of a network is detected [1].

7 Stochastics

An important feature of FERN is the use of one central class for handling random numbers (which is fundamental for stochastic algorithms). This has several advantages:

- If a new, faster random number generator has been developed, it can easily be used for the whole framework.
- By setting the seed value for the random number generator explicitly, you are able to make the simulation deterministic and e.g. reproduce some interesting trajectory of the Marcov Chain
- It is possible (and implemented) to count the number of random number generation of different distributions (which have a significant influence on the performance)

8 Examples

If you plan to use or extend FERN, the first step should be to look at the examples. Some of them emphasize technical aspects (gnuplot system - MichaelisMentenKinetic,MapkBenchmark; observer - LacYComplete, AutocatalyticNetworkExample), some of them show differences of the algotithms (DecayingDimerizingInteractive, DecayingDimerizing, IrreversibleIzomerization) and some of them are supposed to reproduce earlier published results of other implementations (LacZ, LacYComplete, DecayingDimerizingHistogramDistances, LacYHistogramDistances). **Do not forget to match the paths within the code to your example directory!** You can find the examples in the package *fern.example* (just look into the code, they are commented in detail):

8.1 MichaelisMentenKinetic

The most basic example uses the famous enzyme kinetics equation by Michaelis and Menten

$$S + E \rightleftharpoons ES \rightarrow P$$

to introduce fundamental loading and repeated simulation of reaction networks. Furthermore, some advanced usage of the Gnuplot class is presented: Two plots are created from the data of one Observer, one showing the average trend curve over all trajectories, the other showing each trajectory individually. Both plots are updated after each simulation run.

Network file: mm.xml
Point of interest: Basic loading and simulation, creating average and individual trend plots, customize plots

8.2 DecayingDimerizing

Demonstration of performance and accuracy differences of the different simulation algorithms. The reaction network proposed in [4] are used to calculate histograms and the histogram distances [9] of the amount of some molecular species at a special time point in order to compare results of different algorithms. The *Interactive* example reproduces Fig. 3 of [6] and shows the gain of accuracy when using a species bounded tau leaping procedure without loss of performance. The *HistogramDistances* reproduces the values of Fig. 10 of [8]. Running it the first time will cost some time, the results are saved and then loaded in further runs. The same holds for *Plots*, which produces the same plots as *Interactive*.

Network file: decaydimer.xml
Point of interest: accuracy of tau leaping, performance differences

8.3 IrreversibleIzomerization

Uses the irreversible-isomerization model proposed in [4] to show effects of different choices for epsilon. The number of leaps is slightly greater than proposed in the paper because in the paper tau wasn't bound by sigma yet (yielding sometimes to lower tau and hence more leaps).

Network file: isomerization.xml
Point of interest: influence of the choice of tau on the accuracy of tau leaping

8.4 LacZ/LacY

The LacZ/LacY model of procaryotic gene expression proposed by [5, 7] is simulated in different ways: In *LacYComplete*, the full model (containing LacZ and LacY regulation) is simulated for one full cell cycle. Since it is very time consuming, the simulation progress is shown online in the console. In *LacZ*, Fig. 1A of [5] is reproduced, which shows the amount of LacZ over several cell cycles. *LacYHistogramDistances* reproduces the values of [8] Fig. 4, which represent histogram distances of the different procedures regarding the simulation of time 1000 to 1001 of the cell cycle.

Network file: lacz.xml, lacy.xml, lacy1000.xml
Point of interest: real life example, cell growth / division realized by an Observer

8.5 MapkBenchmark

Use the signal transduction pathway network of the epidermal growth factor proposed by [10] to introduce the benchmark system. For more information please refer to the javadoc.

Network file: mapk.xml
Point of interest: Benchmark system

8.6 AutocatalyticNetworkExample

Here, the evolution of a reaction network as proposed by [1] is performed. Then, the autocatalytic subset [1] is determined and extracted. This subnet is then simulated to examine the dynamic behaviour of autocatalytic reaction networks. For more information about the evolution and the detection algorithm, please refer to the javadoc.

Point of interest: Network evolution, extraction of subnets

8.7 Dsmts

Perform a series of tests (refer to http://www.calibayes.ncl.ac.uk/Resources/dsmts). You have to specify the path to the unpacked dsmts archive. The method test produces one line of text containing the test results for each species in the model. If specified, it also produces 4 plots:

- average trend curve of the simulated trajectories and the analytical determined

- stddev trend curve of the simulated trajectories and the analytical determined
- deviation of the simulated averages to the real ones (the z values described in the dsmts user guide)
- deviation of the simulated stddevs to the real ones (the y values described in the dsmts user guide)

Point of interest: correctness of the algorithms and proper sbml loading

8.8 start.sh

Create species population trends by command line. For command line options, start it without parameters to get a usage message.

A FernML example

```
<fernml version="1.0">
 <listOfSpecies>
  <species name="S" initialAmount="100">
   <listOfAnnotations>
    <annotation name="Description">substrate</annotation>
   </listOfAnnotations>
  </species>
  <species initialAmount="20" name="E" />
  <species initialAmount="0" name="P" />
  <species initialAmount="0" name="ES" />
 </listOfSpecies>
 <listOfReactions>
  <reaction kineticConstant="5.0">
   <listOfReactants>
    <speciesReference name="S" />
    <speciesReference name="E" />
   </listOfReactants>
   <listOfProducts><speciesReference name="ES" /></listOfProducts>
   </reaction>
   <reaction kineticConstant="4.0">
 <listOfReactants><speciesReference name="ES" /></listOfReactants>
    <listOfProducts>
     <speciesReference name="S" />
     <speciesReference name="E" />
    </listOfProducts>
   </reaction>
   <reaction kineticConstant="1.0">
 <listOfReactants><speciesReference name="ES" /></listOfReactants>
    <listOfProducts>
     <speciesReference name="P" />
     <speciesReference name="E" />
    </listOfProducts>
   </reaction>
 </listOfReactions>
</fernml>
```

B FernML specifications

If you want a formal description of FernML, the XML Schema file FernMLSchema.xsd is everything you need. But since XML schema files are not dedicated for human readability, you find here a summary about FernML.

The classes of FernML are

- Fernml: root of the XML document tree containing a <listOfSpecies> and a <listOfReactions>
- Species: with two mandatory attributes
  - initialAmount of the default XML type float
  - name of the default XML type string
- Reaction: with two lists of speciesReferences and two attributes
  - kineticConstant (flaot, mandatory)
  - kineticConstantReverisble (float, optional)
- SpeciesReference: with the mandatory attribute *name*; a Species object with the same name has to exist within the <listOfSpecies>, which is checked automatically by the XML schema
- FernBase: base class for the Fernml, Species and Reaction to allow the optional <listOfAnnotations>
- Annotation: with the mandatory attribute name (string) and the annotation as inner text

FernML was designed to represent a chemical reaction network as simple as possible. First, you have to define the molecular species as Species objects within the <listOfSpecies>. Second, you have to give the reactions' stoichiometry by the <listOfProducts> and a <listOfReactants> within a Reaction object. Look at the example above and consider the reaction

$$S + E \rightarrow ES$$

The reactants are defined as speciesReference S and E, the product as speciesReference ES. SpeciesReferences only reference previously (in the <listOfSpecies>) defined Species objects, so no other attributes than the name make sense.

In order to make annotations possible for the various network entities, the classes FernMl, Reaction and Species derive from a class FernBase which allows an optional <listOfAnnotations> child tag for these classes.

C SBML support

At the moment, SBML version 2 levels 1 - 3 are supported, but only follwing branches of an SBML file are read:

- listOfCompartments: for using the volume as a parameter in formulas
- listOfSpecies:
  - the flag hasOnlySubstanceUnits has to be set for each species
  - initialAmount instead of initialConcentration has to be used
  - the flag BoundaryCondition is supported
- listOfParameters: are read and can be overridden by local parameters within a reaction branch
- listOfReactions: fully supported
- listOfEvents: fully supported, but if events are supposed be triggered during simulations, you have to invoke the method registerEvents of your SBMLNetwork. This has to be done because event handling is achieved by observers which have to be registered at the simulator.

Other tags like initialAssignments, Rules, Constraints are not supported at the moment and an exception is thrown if you try to load a network containing one of these. If you want to do the simulation anyway, you can use the ignoreExceptions flag of the second constructor of SBMLNetwork.

References

[1] Kauffmann S.A, The Origins of Order: Self-Organization and Selection in Evolution. New York: Oxford University Press, (1993)

[2] Gillespie D.T., J. Comput. Phys. 22, 403 (1976)

[3] Gibson M.A. and Bruck J., J. Phys. Chem. 104, 1876 (2000)

[4] Gillespie D.T., J. Phys. Chem. 115, 1716 (2001)

[5] Kierzek A.M., Bioinformatics 18, 670 (2002)

[6] Gillespie D.T. and Petzold L., J. Chem. Phys. 119, 8229 (2003)

[7] Tian T. and Burrage K., J. Chem. Phys. 121, 10356 (2004)

[8] Cao Y., J. Chem. Phys. 124, 044109 (2006)

[9] Cao Y. and Petzold L., J. Comp. Phys. 212, 624 (2006)

[10] Lee D.-Y., Metabolic Engineering 8, 112-122 (2006)

[11] A. Finney and M. Hucka., Biochem. Soc. Trans. 31, 1472-1473. (2003)

[12] Puchalka J. and Kierzek A.M., BIOPHYSICAL, 86, 1357 (2004)

[13] Funahashi A. Biosilico, 1, 159-162 (2003)
