# Supplementary material for: FERN – a Java framework for stochastic simulation and evaluation of reaction networks
Source: BMC Bioinformatics. 2008 Aug 29;9:356. doi: 10.1186/1471-2105-9-356 (PMC2553347; doi:10.1186/1471-2105-9-356)
Supplement: Additional file 1 — FERN distribution, Version 1.3. This archive contains the FERN source code and binaries as well as documentation and example models in FernML and SBML. [file 1471-2105-9-356-S1.zip › fern/doc/javadoc/allclasses-frame.html]

All Classes


**All Classes**
  

|  |
| --- |
| AbstractBaseTauLeaping   AbstractKineticConstantPropensityCalculator   AbstractNetworkImpl   AbstractTauLeapingPropensityBoundSimulator   AmountAtMomentObserver   AmountIntervalObserver   AmountManager   AnalysisBase   AndController   *AnnotationManager*   AnnotationManagerImpl   ArrayKineticConstantPropensityCalculator   ArrayMatrixAxes   AutocatalyticNetwork   AutocatalyticNetworkDetection   AutocatalyticNetworkExample   Axes   Benchmark   CatalysedNetwork   *CatalystIterator*   CellDesignerNetworkWrapper   CellDesignerPropensityCalculator   CellGrowthObserver   CollectionAxes   ColorCalculator   ColorCalculator.Scale   ColorPicker   ColorSpectrum   *ComplexDependenciesPropensityCalculator*   ConfigReader   CytoscapeAnnotationManager   CytoscapeColorChangeObserver   CytoscapeColorChangeObserver.ColorChangingNodeAppeareanceCalculator   CytoscapeNetworkWrapper   CytoscapeVisualizer   DecayingDimerizingHistogramDistances   DecayingDimerizingInteractive   DecayingDimerizingPlots   DefaultController   DependencyGraph   Dsmts   ExamplePath   ExtendedPane   ExtendedPane   ExtractSubNetwork   FeatureNotSupportedException   FernCellDesignerPlugin   FernMLAnnotationManager   FernMLNetwork   FernVisualStyle   FireTypeObserver   GibsonBruckSimulator   GillespieEnhanced   GillespieSimple   GnuPlot   *GnuPlotObserver*   HistogramDistanceTestSet   HybridMaximalTimeStep   IndexedPriorityQueue   InstantOutputObserver   IntervalObserver   IntQueue   *IntSearchStructure*   IntStack   IrreversibleIsomerization   LacYComplete   LacYHistogramDistances   LacZ   LeapObserver   MainFrame   MainFrame   MapkBenchmark   MathTree   MathTree.ConstLeaf   MathTree.GlobalLeaf   MathTree.InnerNode   MathTree.Node   MathTree.VarLeaf   MichaelisMentenKinetic   ModifierNetwork   MultiAmountIntervalObserver   *Network*   NetworkChecker   *NetworkChecker.EdgeClassifier*   *NetworkChecker.NodeClassifier*   NetworkLoader   *NetworkSearchAction*   NetworkSearchAction.NeighborType   NetworkTools   *NodeChecker*   NodeCheckerByAnnotation   NumberTools   Observer   OrController   OverviewPane   OverviewPane   *Probability*   Probability.Constant   Probability.ReactionProbability   *PropensityCalculator*   RandomNumber   RandomNumberGeneratorCallObserver   ReactionIntervalObserver   ReversibleNetwork   SBMLEventHandlerObserver   SBMLMathTreeTest   SBMLNetwork   SBMLPropensityCalculator   ShortestPath   *SimulationController*   Simulator   Simulator.FireType   SimulatorCorrectness   SimulatorFireTypes   SimulatorPerformance   SimulatorRandomNumbers   SimulatorTime   Start   Stochastics   TauLeapingAbsoluteBoundSimulator   TauLeapingRelativeBoundSimulator   TauLeapingSpeciesPopulationBoundSimulator   TransposedArrayMatrixAxes   TriggerObserver |
