# Supplementary material for: FERN – a Java framework for stochastic simulation and evaluation of reaction networks
Source: BMC Bioinformatics. 2008 Aug 29;9:356. doi: 10.1186/1471-2105-9-356 (PMC2553347; doi:10.1186/1471-2105-9-356)
Supplement: Additional file 1 — FERN distribution, Version 1.3. This archive contains the FERN source code and binaries as well as documentation and example models in FernML and SBML. [file 1471-2105-9-356-S1.zip › fern/doc/javadoc/constant-values.html]

Constant Field Values


---


|  |  |  |  |  |  |  |  |  |  |  |
| --- | --- | --- | --- | --- | --- | --- | --- | --- | --- | --- |
| |  |  |  |  |  |  |  |  | | --- | --- | --- | --- | --- | --- | --- | --- | | **Overview** | Package | Class | Use | **Tree** | **Deprecated** | **Index** | **Help** | | |  |
| PREV   NEXT | **FRAMES**    **NO FRAMES**     **All Classes** |


---


# Constant Field Values


---

**Contents**

- fern.cellDesigner.\*- fern.cytoscape.\*- fern.network.\*

| fern.cellDesigner.\* |
| --- |

| fern.cellDesigner.ui.ExtendedPane | | |
| --- | --- | --- |
| `public static final String` | `sep` | `"________"` |

| fern.cytoscape.\* |
| --- |

| fern.cytoscape.ui.ExtendedPane | | |
| --- | --- | --- |
| `public static final String` | `sep` | `"________"` |

| fern.network.\* |
| --- |

| fern.network.creation.AutocatalyticNetwork | | |
| --- | --- | --- |
| `public static final String` | `CATALYSTS_FIELD` | `"Catalysts"` |
| `public static final String` | `CATALYSTS_FIELD_REVERSIBLE` | `"CatalystsReversible"` |

| fern.network.modification.ReversibleNetwork | | |
| --- | --- | --- |
| `public static final String` | `REVERSIBLE_SUFFIX` | `"Reversible"` |

---


|  |  |  |  |  |  |  |  |  |  |  |
| --- | --- | --- | --- | --- | --- | --- | --- | --- | --- | --- |
| |  |  |  |  |  |  |  |  | | --- | --- | --- | --- | --- | --- | --- | --- | | **Overview** | Package | Class | Use | **Tree** | **Deprecated** | **Index** | **Help** | | |  |
| PREV   NEXT | **FRAMES**    **NO FRAMES**     **All Classes** |


---
