# Supplementary material for: FERN – a Java framework for stochastic simulation and evaluation of reaction networks
Source: BMC Bioinformatics. 2008 Aug 29;9:356. doi: 10.1186/1471-2105-9-356 (PMC2553347; doi:10.1186/1471-2105-9-356)
Supplement: Additional file 1 — FERN distribution, Version 1.3. This archive contains the FERN source code and binaries as well as documentation and example models in FernML and SBML. [file 1471-2105-9-356-S1.zip › fern/doc/javadoc/fern/analysis/AnalysisBase.html]

AnalysisBase


---


|  |  |  |  |  |  |  |  |  |  |  |
| --- | --- | --- | --- | --- | --- | --- | --- | --- | --- | --- |
| |  |  |  |  |  |  |  |  | | --- | --- | --- | --- | --- | --- | --- | --- | | **Overview** | **Package** | **Class** | **Use** | **Tree** | **Deprecated** | **Index** | **Help** | | |  |
| PREV CLASS   **NEXT CLASS** | **FRAMES**    **NO FRAMES**     **All Classes** |
| SUMMARY: NESTED | FIELD | CONSTR | METHOD | DETAIL: FIELD | CONSTR | METHOD |


---


## fern.analysis Class AnalysisBase

```
java.lang.Object
  fern.analysis.AnalysisBase
```

**Direct Known Subclasses:**: AutocatalyticNetworkDetection, ShortestPath

---

``` public class AnalysisBase extends Object ```

This class can be used as a base class for many analysis algorithms (like AutocatalticNetworkDetection,
ShortestPath, ...). It creates adjacency lists for the molecule species (which are
usually not present in `Network` implementing classes) and presents methods for a
general breath first search and depth first search, which can be controlled by a `NetworkSearchAction`

**Author:**
:   Florian Erhard

**See Also:**: `NetworkSearchAction`

---

| **Field Summary** | |
| --- | --- |
| `protected  int[][]` | `adjListAsPro`             Contains the adjacency list for molecule species towards reactions, where the species is a product. |
| `protected  int[][]` | `adjListAsRea`             Contains the adjacency list for molecule species towards reactions, where the species is a reactant. |
| `protected  Network` | `network`             Contains the network. |
| `protected  Network` | `originalNetwork`             Contains the original network if `network` is a `ModifierNetwork`. |


| **Constructor Summary** | |
| --- | --- |
| `AnalysisBase(Network network)`             Creates an analysis instance. |


| **Method Summary** | |
| --- | --- |
| `int` | `bfs(int[] speciesSource, int[] reactionSource, NetworkSearchAction action)`             Performs a breath first search starting at the given sources (which means the contents of `speciesSource` and `reactionSource` are the initial content of the queue. |
| `protected  void` | `createSpeciesAdjacencyLists()`             Creates the adjacency lists for the molecule species. |
| `int` | `dfs(int[] speciesSource, int[] reactionSource, NetworkSearchAction action)`             Performs a depth first search starting at the given sources (which means the contents of `speciesSource` and `reactionSource` are the initial content of the stack. |
| `int` | `search(IntSearchStructure str, int[] speciesSource, int[] reactionSource, NetworkSearchAction action)`             Performs a search starting at the given sources (which means the contents of `speciesSource` and `reactionSource` are the initial content of the search structure `IntSearchStructure`. |

| **Methods inherited from class java.lang.Object** |
| --- |
| `clone, equals, finalize, getClass, hashCode, notify, notifyAll, toString, wait, wait, wait` |

| **Field Detail** |
| --- |

### network

```
protected Network network
```

:   Contains the network.

---


### originalNetwork

```
protected Network originalNetwork
```

:   Contains the original network if `network` is a `ModifierNetwork`.
    Otherwise it contains also net `network`.

---


### adjListAsRea

```
protected int[][] adjListAsRea
```

:   Contains the adjacency list for molecule species towards reactions, where the species
    is a reactant.

---


### adjListAsPro

```
protected int[][] adjListAsPro
```

:   Contains the adjacency list for molecule species towards reactions, where the species
    is a product.


| **Constructor Detail** |
| --- |

### AnalysisBase

```
public AnalysisBase(Network network)
```

:   Creates an analysis instance. In order to do that, a `Network` is required.
    If the network is a `ModifierNetwork`, the original network is also
    discovered and stored.

    **Parameters:**: `network` - the network for analysis


| **Method Detail** |
| --- |

### createSpeciesAdjacencyLists

```
protected void createSpeciesAdjacencyLists()
```

:   Creates the adjacency lists for the molecule species. A subclass has to invoke this
    before it can use the protected fields adjListAsRea and adjListAsPro.

---


### bfs

```
public int bfs(int[] speciesSource,
               int[] reactionSource,
               NetworkSearchAction action)
```

:   Performs a breath first search starting at the given sources (which means the contents of `speciesSource`
    and `reactionSource` are the initial content of the queue. The search is controlled by an `NetworkSearchAction`.

    :   **Parameters:**: `speciesSource` - indices of the species to start with: `reactionSource` - indices of the reactions to start with: `action` - controls what species/reactions have to be visited and what to do after discovering/finishing a species/reaction **Returns:**: number of visited species/reactions

---


### dfs

```
public int dfs(int[] speciesSource,
               int[] reactionSource,
               NetworkSearchAction action)
```

:   Performs a depth first search starting at the given sources (which means the contents of `speciesSource`
    and `reactionSource` are the initial content of the stack. The search is controlled by an `NetworkSearchAction`.

    :   **Parameters:**: `speciesSource` - indices of the species to start with: `reactionSource` - indices of the reactions to start with: `action` - controls what species/reactions have to be visited and what to do after discovering/finishing a species/reaction **Returns:**: number of visited species/reactions

---


### search

```
public int search(IntSearchStructure str,
                  int[] speciesSource,
                  int[] reactionSource,
                  NetworkSearchAction action)
```

:   Performs a search starting at the given sources (which means the contents of `speciesSource`
    and `reactionSource` are the initial content of the search structure `IntSearchStructure`.
    The search is controlled by an `NetworkSearchAction`.

    :   **Parameters:**: `str` - the search structure (fifo/lifo): `speciesSource` - indices of the species to start with: `reactionSource` - indices of the reactions to start with: `action` - controls what species/reactions have to be visited and what to do after discovering/finishing a species/reaction **Returns:**: number of visited species/reactions **See Also:**: `bfs(int[], int[], NetworkSearchAction)`, `dfs(int[], int[], NetworkSearchAction)`


---


|  |  |  |  |  |  |  |  |  |  |  |
| --- | --- | --- | --- | --- | --- | --- | --- | --- | --- | --- |
| |  |  |  |  |  |  |  |  | | --- | --- | --- | --- | --- | --- | --- | --- | | **Overview** | **Package** | **Class** | **Use** | **Tree** | **Deprecated** | **Index** | **Help** | | |  |
| PREV CLASS   **NEXT CLASS** | **FRAMES**    **NO FRAMES**     **All Classes** |
| SUMMARY: NESTED | FIELD | CONSTR | METHOD | DETAIL: FIELD | CONSTR | METHOD |


---
