# Supplementary material for: FERN – a Java framework for stochastic simulation and evaluation of reaction networks
Source: BMC Bioinformatics. 2008 Aug 29;9:356. doi: 10.1186/1471-2105-9-356 (PMC2553347; doi:10.1186/1471-2105-9-356)
Supplement: Additional file 1 — FERN distribution, Version 1.3. This archive contains the FERN source code and binaries as well as documentation and example models in FernML and SBML. [file 1471-2105-9-356-S1.zip › fern/doc/javadoc/fern/analysis/AutocatalyticNetworkDetection.html]

AutocatalyticNetworkDetection


---


|  |  |  |  |  |  |  |  |  |  |  |
| --- | --- | --- | --- | --- | --- | --- | --- | --- | --- | --- |
| |  |  |  |  |  |  |  |  | | --- | --- | --- | --- | --- | --- | --- | --- | | **Overview** | **Package** | **Class** | **Use** | **Tree** | **Deprecated** | **Index** | **Help** | | |  |
| **PREV CLASS**   **NEXT CLASS** | **FRAMES**    **NO FRAMES**     **All Classes** |
| SUMMARY: NESTED | FIELD | CONSTR | METHOD | DETAIL: FIELD | CONSTR | METHOD |


---


## fern.analysis Class AutocatalyticNetworkDetection

```
java.lang.Object
  fern.analysis.AnalysisBase
      fern.analysis.AutocatalyticNetworkDetection
```

---

``` public class AutocatalyticNetworkDetection extends AnalysisBase ```

Detects the autocatalytic set of the given network if there is any. An autocatalytic
set is defined as a set of species that are produced by a path of reactions, starting
at some food molecules and fully catalyzed by members of the autocatalytic set.

The algorithm iterates over two modified breath first searches until none of them can
exclude species / reactions any more. The first bfs removes reactions that are not
catalyzed in the remaining network (and molecule species that are only produced by that
reactions). The second bfs removes species, that do not have a path from each necessary
food molecule.

**Author:**
:   Florian Erhard

---

| **Field Summary** | |
| --- | --- |

| **Fields inherited from class fern.analysis.AnalysisBase** |
| --- |
| `adjListAsPro, adjListAsRea, network, originalNetwork` |


| **Constructor Summary** | |
| --- | --- |
| `AutocatalyticNetworkDetection(Network network)`             Creates the AutocatalyticDetection by using the in the network built in `CatalystIterator`. |
| `AutocatalyticNetworkDetection(Network network, CatalystIterator cataIt)`             Creates the AutocatalyticDetection by using the second argument as `CatalystIterator`. |


| **Method Summary** | |
| --- | --- |
| `void` | `annotate(String field, String value)`             Adds annotations to each autocatalytic reaction / species. |
| `int` | `detect()`             Performs the detection algorithm. |
| `BitVector` | `getAutocatalyticReactions()`             Gets the autocatalytic reactions as `BitVector`. |
| `BitVector` | `getAutocatalyticSpecies()`             Gets the autocatalytic species as `BitVector`. |
| `protected  int[]` | `getFoodSpecies()`             Gets the food molecules of the network. |
| `boolean` | `isAutocatalyticReaction(int reaction)`             Returns true if the given reaction is autocatalytic. |
| `boolean` | `isAutocatalyticSpecies(int species)`             Returns true if the given species is autocatalytic. |

| **Methods inherited from class fern.analysis.AnalysisBase** |
| --- |
| `bfs, createSpeciesAdjacencyLists, dfs, search` |

| **Methods inherited from class java.lang.Object** |
| --- |
| `clone, equals, finalize, getClass, hashCode, notify, notifyAll, toString, wait, wait, wait` |

| **Constructor Detail** |
| --- |

### AutocatalyticNetworkDetection

```
public AutocatalyticNetworkDetection(Network network)
```

:   Creates the AutocatalyticDetection by using the in the network built in `CatalystIterator`.
    The network has to implement `CatalystIterator`, otherwise an `IllegalArgumentException` is thrown.

    **Parameters:**: `network` - the network to detect the autocatalytic set in

---


### AutocatalyticNetworkDetection

```
public AutocatalyticNetworkDetection(Network network,
                                     CatalystIterator cataIt)
```

:   Creates the AutocatalyticDetection by using the second argument as `CatalystIterator`.

    **Parameters:**: `network` - network the network to detect the autocatalytic set in: `cataIt` - a `CatalystIterator` for the network


| **Method Detail** |
| --- |

### detect

```
public int detect()
```

:   Performs the detection algorithm. The results can be retrieved by the methods
    `getAutocatalyticReactions`,
    `getAutocatalyticSpecies`,
    `isAutocatalyticReaction`,
    `isAutocatalyticSpecies`,
    `annotate`

    :   **Returns:**: number of iterations

---


### getAutocatalyticReactions

```
public BitVector getAutocatalyticReactions()
```

:   Gets the autocatalytic reactions as `BitVector`. Throws a `RuntimeException`
    if the detection algorithms has not been called.

    :   **Returns:**: autocatalytic reactions

---


### getAutocatalyticSpecies

```
public BitVector getAutocatalyticSpecies()
```

:   Gets the autocatalytic species as `BitVector`. Throws a `RuntimeException`
    if the detection algorithms has not been called.

    :   **Returns:**: autocatalytic species

---


### isAutocatalyticReaction

```
public boolean isAutocatalyticReaction(int reaction)
```

:   Returns true if the given reaction is autocatalytic. Throws a `RuntimeException`
    if the detection algorithms has not been called.

    :   **Parameters:**: `reaction` - the reaction index **Returns:**: if the reaction is autocatalytic

---


### isAutocatalyticSpecies

```
public boolean isAutocatalyticSpecies(int species)
```

:   Returns true if the given species is autocatalytic. Throws a `RuntimeException`
    if the detection algorithms has not been called.

    :   **Parameters:**: `species` - the species index **Returns:**: if the species is autocatalytic

---


### annotate

```
public void annotate(String field,
                     String value)
```

:   Adds annotations to each autocatalytic reaction / species.

    :   **Parameters:**: `field` - name of the annotation: `value` - value of the annotation

---


### getFoodSpecies

```
protected int[] getFoodSpecies()
```

:   Gets the food molecules of the network. As default, monomers are the food molecules.

    :   **Returns:**: indices of the food molecules


---


|  |  |  |  |  |  |  |  |  |  |  |
| --- | --- | --- | --- | --- | --- | --- | --- | --- | --- | --- |
| |  |  |  |  |  |  |  |  | | --- | --- | --- | --- | --- | --- | --- | --- | | **Overview** | **Package** | **Class** | **Use** | **Tree** | **Deprecated** | **Index** | **Help** | | |  |
| **PREV CLASS**   **NEXT CLASS** | **FRAMES**    **NO FRAMES**     **All Classes** |
| SUMMARY: NESTED | FIELD | CONSTR | METHOD | DETAIL: FIELD | CONSTR | METHOD |


---
