# Supplementary material for: FERN – a Java framework for stochastic simulation and evaluation of reaction networks
Source: BMC Bioinformatics. 2008 Aug 29;9:356. doi: 10.1186/1471-2105-9-356 (PMC2553347; doi:10.1186/1471-2105-9-356)
Supplement: Additional file 1 — FERN distribution, Version 1.3. This archive contains the FERN source code and binaries as well as documentation and example models in FernML and SBML. [file 1471-2105-9-356-S1.zip › fern/doc/javadoc/fern/analysis/class-use/NetworkSearchAction.html]

Uses of Interface fern.analysis.NetworkSearchAction


---


|  |  |  |  |  |  |  |  |  |  |  |
| --- | --- | --- | --- | --- | --- | --- | --- | --- | --- | --- |
| |  |  |  |  |  |  |  |  | | --- | --- | --- | --- | --- | --- | --- | --- | | **Overview** | **Package** | **Class** | **Use** | **Tree** | **Deprecated** | **Index** | **Help** | | |  |
| PREV   NEXT | **FRAMES**    **NO FRAMES**     **All Classes** |


---


## **Uses of Interface fern.analysis.NetworkSearchAction**

| Packages that use NetworkSearchAction | |
| --- | --- |
| **fern.analysis** | Provides classes and algorithms for analysing networks like ShortestPath, AutocatalyticDetection. |

| Uses of NetworkSearchAction in fern.analysis | |
| --- | --- |

| Methods in fern.analysis with parameters of type NetworkSearchAction | |
| --- | --- |
| `int` | `AnalysisBase.bfs(int[] speciesSource, int[] reactionSource, NetworkSearchAction action)`             Performs a breath first search starting at the given sources (which means the contents of `speciesSource` and `reactionSource` are the initial content of the queue. |
| `int` | `AnalysisBase.dfs(int[] speciesSource, int[] reactionSource, NetworkSearchAction action)`             Performs a depth first search starting at the given sources (which means the contents of `speciesSource` and `reactionSource` are the initial content of the stack. |
| `int` | `AnalysisBase.search(IntSearchStructure str, int[] speciesSource, int[] reactionSource, NetworkSearchAction action)`             Performs a search starting at the given sources (which means the contents of `speciesSource` and `reactionSource` are the initial content of the search structure `IntSearchStructure`. |

---


|  |  |  |  |  |  |  |  |  |  |  |
| --- | --- | --- | --- | --- | --- | --- | --- | --- | --- | --- |
| |  |  |  |  |  |  |  |  | | --- | --- | --- | --- | --- | --- | --- | --- | | **Overview** | **Package** | **Class** | **Use** | **Tree** | **Deprecated** | **Index** | **Help** | | |  |
| PREV   NEXT | **FRAMES**    **NO FRAMES**     **All Classes** |


---
