# Supplementary material for: FERN – a Java framework for stochastic simulation and evaluation of reaction networks
Source: BMC Bioinformatics. 2008 Aug 29;9:356. doi: 10.1186/1471-2105-9-356 (PMC2553347; doi:10.1186/1471-2105-9-356)
Supplement: Additional file 1 — FERN distribution, Version 1.3. This archive contains the FERN source code and binaries as well as documentation and example models in FernML and SBML. [file 1471-2105-9-356-S1.zip › fern/doc/javadoc/fern/analysis/class-use/NodeChecker.html]

Uses of Interface fern.analysis.NodeChecker


---


|  |  |  |  |  |  |  |  |  |  |  |
| --- | --- | --- | --- | --- | --- | --- | --- | --- | --- | --- |
| |  |  |  |  |  |  |  |  | | --- | --- | --- | --- | --- | --- | --- | --- | | **Overview** | **Package** | **Class** | **Use** | **Tree** | **Deprecated** | **Index** | **Help** | | |  |
| PREV   NEXT | **FRAMES**    **NO FRAMES**     **All Classes** |


---


## **Uses of Interface fern.analysis.NodeChecker**

| Packages that use NodeChecker | |
| --- | --- |
| **fern.analysis** | Provides classes and algorithms for analysing networks like ShortestPath, AutocatalyticDetection. |

| Uses of NodeChecker in fern.analysis | |
| --- | --- |

| Classes in fern.analysis that implement NodeChecker | |
| --- | --- |
| `class` | `NodeCheckerByAnnotation`             An instance of `NodeCheckerByAnnotation` can be used to control a search in `AnalysisBase` by a `NetworkSearchAction`. |

| Methods in fern.analysis with parameters of type NodeChecker | |
| --- | --- |
| `ShortestPath.Path` | `ShortestPath.computePath(NodeChecker checker, String toSpecies, String... species)`             Compute the shortest paths from some source species to one species by only using parts of the network specified by the `NodeChecker` `checker`. |
| `ShortestPath.Path[]` | `ShortestPath.computePaths(NodeChecker checker, String... species)`             Compute all shortest paths from some source species by only using parts of the network specified by the `NodeChecker` `checker`. |

---


|  |  |  |  |  |  |  |  |  |  |  |
| --- | --- | --- | --- | --- | --- | --- | --- | --- | --- | --- |
| |  |  |  |  |  |  |  |  | | --- | --- | --- | --- | --- | --- | --- | --- | | **Overview** | **Package** | **Class** | **Use** | **Tree** | **Deprecated** | **Index** | **Help** | | |  |
| PREV   NEXT | **FRAMES**    **NO FRAMES**     **All Classes** |


---
