# Supplementary material for: FERN – a Java framework for stochastic simulation and evaluation of reaction networks
Source: BMC Bioinformatics. 2008 Aug 29;9:356. doi: 10.1186/1471-2105-9-356 (PMC2553347; doi:10.1186/1471-2105-9-356)
Supplement: Additional file 1 — FERN distribution, Version 1.3. This archive contains the FERN source code and binaries as well as documentation and example models in FernML and SBML. [file 1471-2105-9-356-S1.zip › fern/doc/javadoc/fern/analysis/class-use/ShortestPath.Path.html]

Uses of Class fern.analysis.ShortestPath.Path


---


|  |  |  |  |  |  |  |  |  |  |  |
| --- | --- | --- | --- | --- | --- | --- | --- | --- | --- | --- |
| |  |  |  |  |  |  |  |  | | --- | --- | --- | --- | --- | --- | --- | --- | | **Overview** | **Package** | **Class** | **Use** | **Tree** | **Deprecated** | **Index** | **Help** | | |  |
| PREV   NEXT | **FRAMES**    **NO FRAMES**     **All Classes** |


---


## **Uses of Class fern.analysis.ShortestPath.Path**

| Packages that use ShortestPath.Path | |
| --- | --- |
| **fern.analysis** | Provides classes and algorithms for analysing networks like ShortestPath, AutocatalyticDetection. |

| Uses of ShortestPath.Path in fern.analysis | |
| --- | --- |

| Methods in fern.analysis that return ShortestPath.Path | |
| --- | --- |
| `ShortestPath.Path` | `ShortestPath.computePath(NodeChecker checker, String toSpecies, String... species)`             Compute the shortest paths from some source species to one species by only using parts of the network specified by the `NodeChecker` `checker`. |
| `ShortestPath.Path` | `ShortestPath.computePath(String toSpecies, String... species)`             Compute the shortest paths from some source species to one species. |
| `ShortestPath.Path[]` | `ShortestPath.computePaths(NodeChecker checker, String... species)`             Compute all shortest paths from some source species by only using parts of the network specified by the `NodeChecker` `checker`. |
| `ShortestPath.Path[]` | `ShortestPath.computePaths(String... species)`             Compute all shortest paths from some source species. |

---


|  |  |  |  |  |  |  |  |  |  |  |
| --- | --- | --- | --- | --- | --- | --- | --- | --- | --- | --- |
| |  |  |  |  |  |  |  |  | | --- | --- | --- | --- | --- | --- | --- | --- | | **Overview** | **Package** | **Class** | **Use** | **Tree** | **Deprecated** | **Index** | **Help** | | |  |
| PREV   NEXT | **FRAMES**    **NO FRAMES**     **All Classes** |


---
