# Supplementary material for: FERN – a Java framework for stochastic simulation and evaluation of reaction networks
Source: BMC Bioinformatics. 2008 Aug 29;9:356. doi: 10.1186/1471-2105-9-356 (PMC2553347; doi:10.1186/1471-2105-9-356)
Supplement: Additional file 1 — FERN distribution, Version 1.3. This archive contains the FERN source code and binaries as well as documentation and example models in FernML and SBML. [file 1471-2105-9-356-S1.zip › fern/doc/javadoc/fern/analysis/IntStack.html]

IntStack


---


|  |  |  |  |  |  |  |  |  |  |  |
| --- | --- | --- | --- | --- | --- | --- | --- | --- | --- | --- |
| |  |  |  |  |  |  |  |  | | --- | --- | --- | --- | --- | --- | --- | --- | | **Overview** | **Package** | **Class** | **Use** | **Tree** | **Deprecated** | **Index** | **Help** | | |  |
| **PREV CLASS**   **NEXT CLASS** | **FRAMES**    **NO FRAMES**     **All Classes** |
| SUMMARY: NESTED | FIELD | CONSTR | METHOD | DETAIL: FIELD | CONSTR | METHOD |


---


## fern.analysis Class IntStack

```
java.lang.Object
  cern.colt.PersistentObject
      cern.colt.list.AbstractCollection
          cern.colt.list.AbstractList
              cern.colt.list.AbstractIntList
                  cern.colt.list.IntArrayList
                      fern.analysis.IntStack
```

**All Implemented Interfaces:**: IntBufferConsumer, IntSearchStructure, Serializable, Cloneable

---

``` public class IntStack extends IntArrayList implements IntSearchStructure ```

IntStack is an search structure for `AnalysisBase` representing an lifo stack for
a depth first search.

**Author:**
:   Florian Erhard

**See Also:**: Serialized Form

---

| **Field Summary** | |
| --- | --- |

| **Fields inherited from class cern.colt.list.IntArrayList** |
| --- |
| `elements` |

| **Fields inherited from class cern.colt.list.AbstractIntList** |
| --- |
| `size` |


| **Constructor Summary** | |
| --- | --- |
| `IntStack()` |
| `IntStack(int initialSize)` |


| **Method Summary** | |
| --- | --- |
| `int` | `get()` |

| **Methods inherited from class cern.colt.list.IntArrayList** |
| --- |
| `add, beforeInsert, binarySearchFromTo, clone, copy, countSortFromTo, elements, elements, ensureCapacity, equals, forEach, get, getQuick, indexOfFromTo, lastIndexOfFromTo, partFromTo, removeAll, replaceFromToWithFrom, retainAll, reverse, set, setQuick, shuffleFromTo, sortFromTo, trimToSize` |

| **Methods inherited from class cern.colt.list.AbstractIntList** |
| --- |
| `addAllOf, addAllOfFromTo, beforeInsertAllOfFromTo, beforeInsertDummies, binarySearch, contains, delete, fillFromToWith, indexOf, lastIndexOf, mergeSortFromTo, mergeSortFromTo, quickSortFromTo, quickSortFromTo, removeFromTo, replaceFromToWithFromTo, replaceFromWith, setSizeRaw, size, times, toList, toString` |

| **Methods inherited from class cern.colt.list.AbstractList** |
| --- |
| `addAllOf, beforeInsertAllOf, checkRange, checkRangeFromTo, clear, mergeSort, quickSort, remove, setSize, shuffle, sort` |

| **Methods inherited from class cern.colt.list.AbstractCollection** |
| --- |
| `isEmpty` |

| **Methods inherited from class java.lang.Object** |
| --- |
| `finalize, getClass, hashCode, notify, notifyAll, wait, wait, wait` |

| **Methods inherited from interface fern.analysis.IntSearchStructure** |
| --- |
| `add, isEmpty` |

| **Constructor Detail** |
| --- |

### IntStack

```
public IntStack()
```

---


### IntStack

```
public IntStack(int initialSize)
```


| **Method Detail** |
| --- |

### get

```
public int get()
```

:   **Specified by:**: `get` in interface `IntSearchStructure`


---


|  |  |  |  |  |  |  |  |  |  |  |
| --- | --- | --- | --- | --- | --- | --- | --- | --- | --- | --- |
| |  |  |  |  |  |  |  |  | | --- | --- | --- | --- | --- | --- | --- | --- | | **Overview** | **Package** | **Class** | **Use** | **Tree** | **Deprecated** | **Index** | **Help** | | |  |
| **PREV CLASS**   **NEXT CLASS** | **FRAMES**    **NO FRAMES**     **All Classes** |
| SUMMARY: NESTED | FIELD | CONSTR | METHOD | DETAIL: FIELD | CONSTR | METHOD |


---
