# Supplementary material for: FERN – a Java framework for stochastic simulation and evaluation of reaction networks
Source: BMC Bioinformatics. 2008 Aug 29;9:356. doi: 10.1186/1471-2105-9-356 (PMC2553347; doi:10.1186/1471-2105-9-356)
Supplement: Additional file 1 — FERN distribution, Version 1.3. This archive contains the FERN source code and binaries as well as documentation and example models in FernML and SBML. [file 1471-2105-9-356-S1.zip › fern/doc/javadoc/fern/analysis/NetworkSearchAction.html]

NetworkSearchAction


---


|  |  |  |  |  |  |  |  |  |  |  |
| --- | --- | --- | --- | --- | --- | --- | --- | --- | --- | --- |
| |  |  |  |  |  |  |  |  | | --- | --- | --- | --- | --- | --- | --- | --- | | **Overview** | **Package** | **Class** | **Use** | **Tree** | **Deprecated** | **Index** | **Help** | | |  |
| **PREV CLASS**   **NEXT CLASS** | **FRAMES**    **NO FRAMES**     **All Classes** |
| SUMMARY: NESTED | FIELD | CONSTR | METHOD | DETAIL: FIELD | CONSTR | METHOD |


---


## fern.analysis Interface NetworkSearchAction

---

``` public interface NetworkSearchAction ```

Implementing classes of `NetworkSearchAction` are able to control/watch searches in
`AnalysisBase`. On the one hand they control by using `checkReaction`, `checkSpecies` (control
if a network node should be visited) and `getAdditionalSpeciesNeighbors`, `getAdditionalSpeciesNeighbors`
(if there are other neighbors to visit e.g. catalysts of reactions).
On the other hand they can watch the searches by implementing `reactionDiscovered`, `reactionFinished` and
`speciesDiscovered`, `speciesFinished`.

**Author:**
:   Florian Erhard

---

| **Nested Class Summary** | |
| --- | --- |
| `static class` | `NetworkSearchAction.NeighborType`             Defines different types of neighborhoods in a `Network`. |


| **Method Summary** | |
| --- | --- |
| `boolean` | `checkReaction(int reaction, NetworkSearchAction.NeighborType neighborType)`             Gets called, before the reaction is inserted into the search structure. |
| `boolean` | `checkSpecies(int species, NetworkSearchAction.NeighborType neighborType)`             Gets called, before the species is inserted into the search structure. |
| `void` | `finished()`             Gets called, when the search is done. |
| `Iterable<Integer>` | `getAdditionalReactionNeighbors(int index)`             Returns an iterator for additional neighbors of this species |
| `Iterable<Integer>` | `getAdditionalSpeciesNeighbors(int index)`             Returns an iterator for additional neighbors of this reaction |
| `void` | `initialize(Network net)`             Gets called before anything is inserted into the search structure. |
| `void` | `reactionDiscovered(int reaction)`             Gets called when a reaction is inserted into the search structure. |
| `void` | `reactionFinished(int reaction)`             Gets called when a reaction gets out of the search structure. |
| `void` | `speciesDiscovered(int species)`             Gets called when a species is inserted into the search structure. |
| `void` | `speciesFinished(int species)`             Gets called when a species gets out of the search structure. |

| **Method Detail** |
| --- |

### reactionDiscovered

```
void reactionDiscovered(int reaction)
```

:   Gets called when a reaction is inserted into the search structure.

    :   **Parameters:**: `reaction` - index of the inserted reaction

---


### speciesDiscovered

```
void speciesDiscovered(int species)
```

:   Gets called when a species is inserted into the search structure.

    :   **Parameters:**: `species` - index of the inserted species

---


### reactionFinished

```
void reactionFinished(int reaction)
```

:   Gets called when a reaction gets out of the search structure.

    :   **Parameters:**: `reaction` - index of the reactions

---


### speciesFinished

```
void speciesFinished(int species)
```

:   Gets called when a species gets out of the search structure.

    :   **Parameters:**: `species` - index of the species

---


### checkSpecies

```
boolean checkSpecies(int species,
                     NetworkSearchAction.NeighborType neighborType)
```

:   Gets called, before the species is inserted into the search structure.
    If the implementing instance returns false, the species is not inserted.

    :   **Parameters:**: `species` - species index: `neighborType` - one of the NeighborTypes **Returns:**: true if the species should be inserted

---


### checkReaction

```
boolean checkReaction(int reaction,
                      NetworkSearchAction.NeighborType neighborType)
```

:   Gets called, before the reaction is inserted into the search structure.
    If the implementing instance returns false, the reaction is not inserted.

    :   **Parameters:**: `reaction` - reaction index: `neighborType` - one of the NeighborTypes **Returns:**: true if the reaction should be inserted

---


### initialize

```
void initialize(Network net)
```

:   Gets called before anything is inserted into the search structure.

    :   **Parameters:**: `net` - the network where the search is going to be performed

---


### finished

```
void finished()
```

:   Gets called, when the search is done.

---


### getAdditionalSpeciesNeighbors

```
Iterable<Integer> getAdditionalSpeciesNeighbors(int index)
```

:   Returns an iterator for additional neighbors of this reaction

    :   **Parameters:**: `index` - reaction index **Returns:**: iterator of additional neighbors

---


### getAdditionalReactionNeighbors

```
Iterable<Integer> getAdditionalReactionNeighbors(int index)
```

:   Returns an iterator for additional neighbors of this species

    :   **Parameters:**: `index` - species index **Returns:**: iterator of additional neighbors


---


|  |  |  |  |  |  |  |  |  |  |  |
| --- | --- | --- | --- | --- | --- | --- | --- | --- | --- | --- |
| |  |  |  |  |  |  |  |  | | --- | --- | --- | --- | --- | --- | --- | --- | | **Overview** | **Package** | **Class** | **Use** | **Tree** | **Deprecated** | **Index** | **Help** | | |  |
| **PREV CLASS**   **NEXT CLASS** | **FRAMES**    **NO FRAMES**     **All Classes** |
| SUMMARY: NESTED | FIELD | CONSTR | METHOD | DETAIL: FIELD | CONSTR | METHOD |


---
