# Supplementary material for: FERN – a Java framework for stochastic simulation and evaluation of reaction networks
Source: BMC Bioinformatics. 2008 Aug 29;9:356. doi: 10.1186/1471-2105-9-356 (PMC2553347; doi:10.1186/1471-2105-9-356)
Supplement: Additional file 1 — FERN distribution, Version 1.3. This archive contains the FERN source code and binaries as well as documentation and example models in FernML and SBML. [file 1471-2105-9-356-S1.zip › fern/doc/javadoc/fern/analysis/NetworkSearchAction.NeighborType.html]

NetworkSearchAction.NeighborType


---


|  |  |  |  |  |  |  |  |  |  |  |
| --- | --- | --- | --- | --- | --- | --- | --- | --- | --- | --- |
| |  |  |  |  |  |  |  |  | | --- | --- | --- | --- | --- | --- | --- | --- | | **Overview** | **Package** | **Class** | **Use** | **Tree** | **Deprecated** | **Index** | **Help** | | |  |
| **PREV CLASS**   **NEXT CLASS** | **FRAMES**    **NO FRAMES**     **All Classes** |
| SUMMARY: NESTED | ENUM CONSTANTS | FIELD | METHOD | DETAIL: ENUM CONSTANTS | FIELD | METHOD |


---


## fern.analysis Enum NetworkSearchAction.NeighborType

```
java.lang.Object
  java.lang.Enum<NetworkSearchAction.NeighborType>
      fern.analysis.NetworkSearchAction.NeighborType
```

**All Implemented Interfaces:**: Serializable, Comparable<NetworkSearchAction.NeighborType>

**Enclosing interface:**: NetworkSearchAction

---

``` public static enum NetworkSearchAction.NeighborType extends Enum<NetworkSearchAction.NeighborType> ```

Defines different types of neighborhoods in a `Network`.

**Author:**
:   Florian Erhard

---

| **Enum Constant Summary** | |
| --- | --- |
| `Additional` |
| `Product` |
| `Reactant` |


| **Method Summary** | |
| --- | --- |
| `static NetworkSearchAction.NeighborType` | `valueOf(String name)`             Returns the enum constant of this type with the specified name. |
| `static NetworkSearchAction.NeighborType[]` | `values()`             Returns an array containing the constants of this enum type, in the order they are declared. |

| **Methods inherited from class java.lang.Enum** |
| --- |
| `clone, compareTo, equals, finalize, getDeclaringClass, hashCode, name, ordinal, toString, valueOf` |

| **Methods inherited from class java.lang.Object** |
| --- |
| `getClass, notify, notifyAll, wait, wait, wait` |

| **Enum Constant Detail** |
| --- |

### Reactant

```
public static final NetworkSearchAction.NeighborType Reactant
```

---


### Product

```
public static final NetworkSearchAction.NeighborType Product
```

---


### Additional

```
public static final NetworkSearchAction.NeighborType Additional
```


| **Method Detail** |
| --- |

### values

```
public static NetworkSearchAction.NeighborType[] values()
```

:   Returns an array containing the constants of this enum type, in
    the order they are declared. This method may be used to iterate
    over the constants as follows:

    ```
    for (NetworkSearchAction.NeighborType c : NetworkSearchAction.NeighborType.values())
        System.out.println(c);
    ```

    :   **Returns:**: an array containing the constants of this enum type, in the order they are declared

---


### valueOf

```
public static NetworkSearchAction.NeighborType valueOf(String name)
```

:   Returns the enum constant of this type with the specified name.
    The string must match *exactly* an identifier used to declare an
    enum constant in this type. (Extraneous whitespace characters are
    not permitted.)

    :   **Parameters:**: `name` - the name of the enum constant to be returned. **Returns:**: the enum constant with the specified name **Throws:**: `IllegalArgumentException` - if this enum type has no constant with the specified name: `NullPointerException` - if the argument is null


---


|  |  |  |  |  |  |  |  |  |  |  |
| --- | --- | --- | --- | --- | --- | --- | --- | --- | --- | --- |
| |  |  |  |  |  |  |  |  | | --- | --- | --- | --- | --- | --- | --- | --- | | **Overview** | **Package** | **Class** | **Use** | **Tree** | **Deprecated** | **Index** | **Help** | | |  |
| **PREV CLASS**   **NEXT CLASS** | **FRAMES**    **NO FRAMES**     **All Classes** |
| SUMMARY: NESTED | ENUM CONSTANTS | FIELD | METHOD | DETAIL: ENUM CONSTANTS | FIELD | METHOD |


---
