# Supplementary material for: FERN – a Java framework for stochastic simulation and evaluation of reaction networks
Source: BMC Bioinformatics. 2008 Aug 29;9:356. doi: 10.1186/1471-2105-9-356 (PMC2553347; doi:10.1186/1471-2105-9-356)
Supplement: Additional file 1 — FERN distribution, Version 1.3. This archive contains the FERN source code and binaries as well as documentation and example models in FernML and SBML. [file 1471-2105-9-356-S1.zip › fern/doc/javadoc/fern/analysis/NodeChecker.html]

NodeChecker


---


|  |  |  |  |  |  |  |  |  |  |  |
| --- | --- | --- | --- | --- | --- | --- | --- | --- | --- | --- |
| |  |  |  |  |  |  |  |  | | --- | --- | --- | --- | --- | --- | --- | --- | | **Overview** | **Package** | **Class** | **Use** | **Tree** | **Deprecated** | **Index** | **Help** | | |  |
| **PREV CLASS**   **NEXT CLASS** | **FRAMES**    **NO FRAMES**     **All Classes** |
| SUMMARY: NESTED | FIELD | CONSTR | METHOD | DETAIL: FIELD | CONSTR | METHOD |


---


## fern.analysis Interface NodeChecker

**All Known Implementing Classes:**: NodeCheckerByAnnotation

---

``` public interface NodeChecker ```

Implementing classes can be used for a `NetworkSearchAction`s `checkReaction`,
`checkSpecies`, if the information whether or not to visit the nodes is not
accessible for the `NetworkSearchAction`.

**Author:**
:   Florian Erhard

---

| **Method Summary** | |
| --- | --- |
| `boolean` | `checkReactionNode(Network network, int reaction)` |
| `boolean` | `checkSpeciesNode(Network network, int species)` |

| **Method Detail** |
| --- |

### checkReactionNode

```
boolean checkReactionNode(Network network,
                          int reaction)
```

---


### checkSpeciesNode

```
boolean checkSpeciesNode(Network network,
                         int species)
```


---


|  |  |  |  |  |  |  |  |  |  |  |
| --- | --- | --- | --- | --- | --- | --- | --- | --- | --- | --- |
| |  |  |  |  |  |  |  |  | | --- | --- | --- | --- | --- | --- | --- | --- | | **Overview** | **Package** | **Class** | **Use** | **Tree** | **Deprecated** | **Index** | **Help** | | |  |
| **PREV CLASS**   **NEXT CLASS** | **FRAMES**    **NO FRAMES**     **All Classes** |
| SUMMARY: NESTED | FIELD | CONSTR | METHOD | DETAIL: FIELD | CONSTR | METHOD |


---
