# Supplementary material for: FERN – a Java framework for stochastic simulation and evaluation of reaction networks
Source: BMC Bioinformatics. 2008 Aug 29;9:356. doi: 10.1186/1471-2105-9-356 (PMC2553347; doi:10.1186/1471-2105-9-356)
Supplement: Additional file 1 — FERN distribution, Version 1.3. This archive contains the FERN source code and binaries as well as documentation and example models in FernML and SBML. [file 1471-2105-9-356-S1.zip › fern/doc/javadoc/fern/analysis/NodeCheckerByAnnotation.html]

NodeCheckerByAnnotation


---


|  |  |  |  |  |  |  |  |  |  |  |
| --- | --- | --- | --- | --- | --- | --- | --- | --- | --- | --- |
| |  |  |  |  |  |  |  |  | | --- | --- | --- | --- | --- | --- | --- | --- | | **Overview** | **Package** | **Class** | **Use** | **Tree** | **Deprecated** | **Index** | **Help** | | |  |
| **PREV CLASS**   **NEXT CLASS** | **FRAMES**    **NO FRAMES**     **All Classes** |
| SUMMARY: NESTED | FIELD | CONSTR | METHOD | DETAIL: FIELD | CONSTR | METHOD |


---


## fern.analysis Class NodeCheckerByAnnotation

```
java.lang.Object
  fern.analysis.NodeCheckerByAnnotation
```

**All Implemented Interfaces:**: NodeChecker

---

``` public class NodeCheckerByAnnotation extends Object implements NodeChecker ```

An instance of `NodeCheckerByAnnotation` can be used to control a search in
`AnalysisBase` by a `NetworkSearchAction`. Then the reactions / species
in the network are only visited, if they have the specified annotation.

**Author:**
:   Florian Erhard

---

| **Constructor Summary** | |
| --- | --- |
| `NodeCheckerByAnnotation(String field, String value)`             Creates the NodeChecker with the specified annotation field and value. |


| **Method Summary** | |
| --- | --- |
| `boolean` | `checkReactionNode(Network network, int reaction)` |
| `boolean` | `checkSpeciesNode(Network network, int species)` |

| **Methods inherited from class java.lang.Object** |
| --- |
| `clone, equals, finalize, getClass, hashCode, notify, notifyAll, toString, wait, wait, wait` |

| **Constructor Detail** |
| --- |

### NodeCheckerByAnnotation

```
public NodeCheckerByAnnotation(String field,
                               String value)
```

:   Creates the NodeChecker with the specified annotation field and value. If
    value is `null` then the reaction / species is valid if an annotation
    named `field` exists.

    **Parameters:**: `field` - the annotation name: `value` - the annotation value


| **Method Detail** |
| --- |

### checkReactionNode

```
public boolean checkReactionNode(Network network,
                                 int reaction)
```

:   **Specified by:**: `checkReactionNode` in interface `NodeChecker`

---


### checkSpeciesNode

```
public boolean checkSpeciesNode(Network network,
                                int species)
```

:   **Specified by:**: `checkSpeciesNode` in interface `NodeChecker`


---


|  |  |  |  |  |  |  |  |  |  |  |
| --- | --- | --- | --- | --- | --- | --- | --- | --- | --- | --- |
| |  |  |  |  |  |  |  |  | | --- | --- | --- | --- | --- | --- | --- | --- | | **Overview** | **Package** | **Class** | **Use** | **Tree** | **Deprecated** | **Index** | **Help** | | |  |
| **PREV CLASS**   **NEXT CLASS** | **FRAMES**    **NO FRAMES**     **All Classes** |
| SUMMARY: NESTED | FIELD | CONSTR | METHOD | DETAIL: FIELD | CONSTR | METHOD |


---
