# Supplementary material for: FERN – a Java framework for stochastic simulation and evaluation of reaction networks
Source: BMC Bioinformatics. 2008 Aug 29;9:356. doi: 10.1186/1471-2105-9-356 (PMC2553347; doi:10.1186/1471-2105-9-356)
Supplement: Additional file 1 — FERN distribution, Version 1.3. This archive contains the FERN source code and binaries as well as documentation and example models in FernML and SBML. [file 1471-2105-9-356-S1.zip › fern/doc/javadoc/fern/analysis/package-summary.html]

fern.analysis


---


|  |  |  |  |  |  |  |  |  |  |  |
| --- | --- | --- | --- | --- | --- | --- | --- | --- | --- | --- |
| |  |  |  |  |  |  |  |  | | --- | --- | --- | --- | --- | --- | --- | --- | | **Overview** | **Package** | Class | **Use** | **Tree** | **Deprecated** | **Index** | **Help** | | |  |
| **PREV PACKAGE**   **NEXT PACKAGE** | **FRAMES**    **NO FRAMES**     **All Classes** |


---

## Package fern.analysis

Provides classes and algorithms for analysing networks like ShortestPath, AutocatalyticDetection.

**See:**
  
          **Description**

| **Interface Summary** | |
| --- | --- |
| **IntSearchStructure** | Implementing class can be used as search structure for searches in `AnalysisBase`. |
| **NetworkSearchAction** | Implementing classes of `NetworkSearchAction` are able to control/watch searches in `AnalysisBase`. |
| **NodeChecker** | Implementing classes can be used for a `NetworkSearchAction`s `checkReaction`, `checkSpecies`, if the information whether or not to visit the nodes is not accessible for the `NetworkSearchAction`. |

| **Class Summary** | |
| --- | --- |
| **AnalysisBase** | This class can be used as a base class for many analysis algorithms (like AutocatalticNetworkDetection, ShortestPath, ...). |
| **AutocatalyticNetworkDetection** | Detects the autocatalytic set of the given network if there is any. |
| **IntQueue** | IntQueue is an search structure for `AnalysisBase` representing an fifo queue for a breadth first search. |
| **IntStack** | IntStack is an search structure for `AnalysisBase` representing an lifo stack for a depth first search. |
| **NodeCheckerByAnnotation** | An instance of `NodeCheckerByAnnotation` can be used to control a search in `AnalysisBase` by a `NetworkSearchAction`. |
| **ShortestPath** | Computes shortest paths in the network by a bfs. |

| **Enum Summary** | |
| --- | --- |
| **NetworkSearchAction.NeighborType** | Defines different types of neighborhoods in a `Network`. |

## Package fern.analysis Description

Provides classes and algorithms for analysing networks like ShortestPath, AutocatalyticDetection.

---


|  |  |  |  |  |  |  |  |  |  |  |
| --- | --- | --- | --- | --- | --- | --- | --- | --- | --- | --- |
| |  |  |  |  |  |  |  |  | | --- | --- | --- | --- | --- | --- | --- | --- | | **Overview** | **Package** | Class | **Use** | **Tree** | **Deprecated** | **Index** | **Help** | | |  |
| **PREV PACKAGE**   **NEXT PACKAGE** | **FRAMES**    **NO FRAMES**     **All Classes** |


---
