# Supplementary material for: FERN – a Java framework for stochastic simulation and evaluation of reaction networks
Source: BMC Bioinformatics. 2008 Aug 29;9:356. doi: 10.1186/1471-2105-9-356 (PMC2553347; doi:10.1186/1471-2105-9-356)
Supplement: Additional file 1 — FERN distribution, Version 1.3. This archive contains the FERN source code and binaries as well as documentation and example models in FernML and SBML. [file 1471-2105-9-356-S1.zip › fern/doc/javadoc/fern/analysis/package-tree.html]

fern.analysis Class Hierarchy


---


|  |  |  |  |  |  |  |  |  |  |  |
| --- | --- | --- | --- | --- | --- | --- | --- | --- | --- | --- |
| |  |  |  |  |  |  |  |  | | --- | --- | --- | --- | --- | --- | --- | --- | | **Overview** | **Package** | Class | Use | **Tree** | **Deprecated** | **Index** | **Help** | | |  |
| **PREV**   **NEXT** | **FRAMES**    **NO FRAMES**     **All Classes** |


---


## Hierarchy For Package fern.analysis

**Package Hierarchies:**: All Packages

---

## Class Hierarchy

- java.lang.**Object**
  - fern.analysis.**AnalysisBase**
    - fern.analysis.**AutocatalyticNetworkDetection**- fern.analysis.**ShortestPath**- fern.analysis.**NodeCheckerByAnnotation** (implements fern.analysis.NodeChecker)- cern.colt.**PersistentObject** (implements java.lang.Cloneable, java.io.Serializable)
        - cern.colt.list.**AbstractCollection**
          - cern.colt.list.**AbstractList**
            - cern.colt.list.**AbstractIntList** (implements cern.colt.buffer.IntBufferConsumer)
              - cern.colt.list.**IntArrayList**
                - fern.analysis.**IntQueue** (implements fern.analysis.IntSearchStructure)- fern.analysis.**IntStack** (implements fern.analysis.IntSearchStructure)- fern.analysis.**ShortestPath.Path**

## Interface Hierarchy

- fern.analysis.**IntSearchStructure**- fern.analysis.**NetworkSearchAction**- fern.analysis.**NodeChecker**

## Enum Hierarchy

- java.lang.**Object**
  - java.lang.**Enum**<E> (implements java.lang.Comparable<T>, java.io.Serializable)
    - fern.analysis.**NetworkSearchAction.NeighborType**

---


|  |  |  |  |  |  |  |  |  |  |  |
| --- | --- | --- | --- | --- | --- | --- | --- | --- | --- | --- |
| |  |  |  |  |  |  |  |  | | --- | --- | --- | --- | --- | --- | --- | --- | | **Overview** | **Package** | Class | Use | **Tree** | **Deprecated** | **Index** | **Help** | | |  |
| **PREV**   **NEXT** | **FRAMES**    **NO FRAMES**     **All Classes** |


---
