# Supplementary material for: FERN – a Java framework for stochastic simulation and evaluation of reaction networks
Source: BMC Bioinformatics. 2008 Aug 29;9:356. doi: 10.1186/1471-2105-9-356 (PMC2553347; doi:10.1186/1471-2105-9-356)
Supplement: Additional file 1 — FERN distribution, Version 1.3. This archive contains the FERN source code and binaries as well as documentation and example models in FernML and SBML. [file 1471-2105-9-356-S1.zip › fern/doc/javadoc/fern/analysis/package-use.html]

Uses of Package fern.analysis


---


|  |  |  |  |  |  |  |  |  |  |  |
| --- | --- | --- | --- | --- | --- | --- | --- | --- | --- | --- |
| |  |  |  |  |  |  |  |  | | --- | --- | --- | --- | --- | --- | --- | --- | | **Overview** | **Package** | Class | **Use** | **Tree** | **Deprecated** | **Index** | **Help** | | |  |
| PREV   NEXT | **FRAMES**    **NO FRAMES**     **All Classes** |


---


## **Uses of Package fern.analysis**

| Packages that use fern.analysis | |
| --- | --- |
| **fern.analysis** | Provides classes and algorithms for analysing networks like ShortestPath, AutocatalyticDetection. |

| Classes in fern.analysis used by fern.analysis | |
| --- | --- |
| ****AnalysisBase****             This class can be used as a base class for many analysis algorithms (like AutocatalticNetworkDetection, ShortestPath, ...). |
| ****IntSearchStructure****             Implementing class can be used as search structure for searches in `AnalysisBase`. |
| ****NetworkSearchAction****             Implementing classes of `NetworkSearchAction` are able to control/watch searches in `AnalysisBase`. |
| ****NetworkSearchAction.NeighborType****             Defines different types of neighborhoods in a `Network`. |
| ****NodeChecker****             Implementing classes can be used for a `NetworkSearchAction`s `checkReaction`, `checkSpecies`, if the information whether or not to visit the nodes is not accessible for the `NetworkSearchAction`. |
| ****ShortestPath.Path****             Encapsulates a path from one species to another. |

---


|  |  |  |  |  |  |  |  |  |  |  |
| --- | --- | --- | --- | --- | --- | --- | --- | --- | --- | --- |
| |  |  |  |  |  |  |  |  | | --- | --- | --- | --- | --- | --- | --- | --- | | **Overview** | **Package** | Class | **Use** | **Tree** | **Deprecated** | **Index** | **Help** | | |  |
| PREV   NEXT | **FRAMES**    **NO FRAMES**     **All Classes** |


---
