# Supplementary material for: FERN – a Java framework for stochastic simulation and evaluation of reaction networks
Source: BMC Bioinformatics. 2008 Aug 29;9:356. doi: 10.1186/1471-2105-9-356 (PMC2553347; doi:10.1186/1471-2105-9-356)
Supplement: Additional file 1 — FERN distribution, Version 1.3. This archive contains the FERN source code and binaries as well as documentation and example models in FernML and SBML. [file 1471-2105-9-356-S1.zip › fern/doc/javadoc/fern/analysis/ShortestPath.html]

ShortestPath


---


|  |  |  |  |  |  |  |  |  |  |  |
| --- | --- | --- | --- | --- | --- | --- | --- | --- | --- | --- |
| |  |  |  |  |  |  |  |  | | --- | --- | --- | --- | --- | --- | --- | --- | | **Overview** | **Package** | **Class** | **Use** | **Tree** | **Deprecated** | **Index** | **Help** | | |  |
| **PREV CLASS**   **NEXT CLASS** | **FRAMES**    **NO FRAMES**     **All Classes** |
| SUMMARY: NESTED | FIELD | CONSTR | METHOD | DETAIL: FIELD | CONSTR | METHOD |


---


## fern.analysis Class ShortestPath

```
java.lang.Object
  fern.analysis.AnalysisBase
      fern.analysis.ShortestPath
```

---

``` public class ShortestPath extends AnalysisBase ```

Computes shortest paths in the network by a bfs. Either the path from some
source species to only one species can be calculated (by using one of the
`computePath` methods) or paths to all species (by `computePaths`).
A `NodeChecker` can optionally be given for each method.

**Author:**
:   Florian Erhard

---

| **Nested Class Summary** | |
| --- | --- |
| `class` | `ShortestPath.Path`             Encapsulates a path from one species to another. |


| **Field Summary** | |
| --- | --- |

| **Fields inherited from class fern.analysis.AnalysisBase** |
| --- |
| `adjListAsPro, adjListAsRea, network, originalNetwork` |


| **Constructor Summary** | |
| --- | --- |
| `ShortestPath(Network network)`             Creates the class with the specified network. |


| **Method Summary** | |
| --- | --- |
| `ShortestPath.Path` | `computePath(NodeChecker checker, String toSpecies, String... species)`             Compute the shortest paths from some source species to one species by only using parts of the network specified by the `NodeChecker` `checker`. |
| `ShortestPath.Path` | `computePath(String toSpecies, String... species)`             Compute the shortest paths from some source species to one species. |
| `ShortestPath.Path[]` | `computePaths(NodeChecker checker, String... species)`             Compute all shortest paths from some source species by only using parts of the network specified by the `NodeChecker` `checker`. |
| `ShortestPath.Path[]` | `computePaths(String... species)`             Compute all shortest paths from some source species. |

| **Methods inherited from class fern.analysis.AnalysisBase** |
| --- |
| `bfs, createSpeciesAdjacencyLists, dfs, search` |

| **Methods inherited from class java.lang.Object** |
| --- |
| `clone, equals, finalize, getClass, hashCode, notify, notifyAll, toString, wait, wait, wait` |

| **Constructor Detail** |
| --- |

### ShortestPath

```
public ShortestPath(Network network)
```

:   Creates the class with the specified network.

    **Parameters:**: `network` - the network where shortest paths shall be computed


| **Method Detail** |
| --- |

### computePaths

```
public ShortestPath.Path[] computePaths(String... species)
```

:   Compute all shortest paths from some source species.

    :   **Parameters:**: `species` - the names of the source species **Returns:**: an array of paths **See Also:**: `ShortestPath.Path`

---


### computePaths

```
public ShortestPath.Path[] computePaths(NodeChecker checker,
                                        String... species)
```

:   Compute all shortest paths from some source species by only using parts of the
    network specified by the `NodeChecker` `checker`.

    :   **Parameters:**: `species` - the names of the source species: `checker` - a NodeChecker for the search **Returns:**: an array of paths **See Also:**: `ShortestPath.Path`

---


### computePath

```
public ShortestPath.Path computePath(String toSpecies,
                                     String... species)
```

:   Compute the shortest paths from some source species to one species.

    :   **Parameters:**: `toSpecies` - the name of the species where the shortest path should be computed to: `species` - the names of the source species **Returns:**: the shortest path **See Also:**: `ShortestPath.Path`

---


### computePath

```
public ShortestPath.Path computePath(NodeChecker checker,
                                     String toSpecies,
                                     String... species)
```

:   Compute the shortest paths from some source species to one species by only using parts of the
    network specified by the `NodeChecker` `checker`.

    :   **Parameters:**: `checker` - a NodeChecker for the search: `toSpecies` - the name of the species where the shortest path should be computed to: `species` - the names of the source species **Returns:**: the shortest path **See Also:**: `ShortestPath.Path`


---


|  |  |  |  |  |  |  |  |  |  |  |
| --- | --- | --- | --- | --- | --- | --- | --- | --- | --- | --- |
| |  |  |  |  |  |  |  |  | | --- | --- | --- | --- | --- | --- | --- | --- | | **Overview** | **Package** | **Class** | **Use** | **Tree** | **Deprecated** | **Index** | **Help** | | |  |
| **PREV CLASS**   **NEXT CLASS** | **FRAMES**    **NO FRAMES**     **All Classes** |
| SUMMARY: NESTED | FIELD | CONSTR | METHOD | DETAIL: FIELD | CONSTR | METHOD |


---
