# Supplementary material for: FERN – a Java framework for stochastic simulation and evaluation of reaction networks
Source: BMC Bioinformatics. 2008 Aug 29;9:356. doi: 10.1186/1471-2105-9-356 (PMC2553347; doi:10.1186/1471-2105-9-356)
Supplement: Additional file 1 — FERN distribution, Version 1.3. This archive contains the FERN source code and binaries as well as documentation and example models in FernML and SBML. [file 1471-2105-9-356-S1.zip › fern/doc/javadoc/fern/analysis/ShortestPath.Path.html]

ShortestPath.Path


---


|  |  |  |  |  |  |  |  |  |  |  |
| --- | --- | --- | --- | --- | --- | --- | --- | --- | --- | --- |
| |  |  |  |  |  |  |  |  | | --- | --- | --- | --- | --- | --- | --- | --- | | **Overview** | **Package** | **Class** | **Use** | **Tree** | **Deprecated** | **Index** | **Help** | | |  |
| **PREV CLASS**   NEXT CLASS | **FRAMES**    **NO FRAMES**     **All Classes** |
| SUMMARY: NESTED | FIELD | CONSTR | METHOD | DETAIL: FIELD | CONSTR | METHOD |


---


## fern.analysis Class ShortestPath.Path

```
java.lang.Object
  fern.analysis.ShortestPath.Path
```

**Enclosing class:**: ShortestPath

---

``` public class ShortestPath.Path extends Object ```

Encapsulates a path from one species to another.

**Author:**
:   Florian Erhard

---

| **Constructor Summary** | |
| --- | --- |
| `ShortestPath.Path(int[] path)`             Creates a path from an array. |


| **Method Summary** | |
| --- | --- |
| `int[]` | `getRawData()`             Returns the raw path: Is ends with a species index and is composed of alternating indices of species and reactions. |
| `int[]` | `getReactions()`             Returns all the reactions on this path. |
| `int[]` | `getSpecies()`             Returns all the species on this path. |
| `String` | `toString()` |

| **Methods inherited from class java.lang.Object** |
| --- |
| `clone, equals, finalize, getClass, hashCode, notify, notifyAll, wait, wait, wait` |

| **Constructor Detail** |
| --- |

### ShortestPath.Path

```
public ShortestPath.Path(int[] path)
```

:   Creates a path from an array. The array has to end with a species and has
    to be composed of alternating indices of species and reactions.

    **Parameters:**: `path` - Arrays containing the network indices of the path's components


| **Method Detail** |
| --- |

### getRawData

```
public int[] getRawData()
```

:   Returns the raw path: Is ends with a species index and is composed of alternating
    indices of species and reactions.

    :   **Returns:**: Raw path of alternating indices of species and reactions ending at a species index.

---


### getSpecies

```
public int[] getSpecies()
```

:   Returns all the species on this path.

    :   **Returns:**: Indices of the species.

---


### getReactions

```
public int[] getReactions()
```

:   Returns all the reactions on this path.

    :   **Returns:**: Indices of the reactions.

---


### toString

```
public String toString()
```

:   **Overrides:**: `toString` in class `Object`


---


|  |  |  |  |  |  |  |  |  |  |  |
| --- | --- | --- | --- | --- | --- | --- | --- | --- | --- | --- |
| |  |  |  |  |  |  |  |  | | --- | --- | --- | --- | --- | --- | --- | --- | | **Overview** | **Package** | **Class** | **Use** | **Tree** | **Deprecated** | **Index** | **Help** | | |  |
| **PREV CLASS**   NEXT CLASS | **FRAMES**    **NO FRAMES**     **All Classes** |
| SUMMARY: NESTED | FIELD | CONSTR | METHOD | DETAIL: FIELD | CONSTR | METHOD |


---
