# Supplementary material for: FERN – a Java framework for stochastic simulation and evaluation of reaction networks
Source: BMC Bioinformatics. 2008 Aug 29;9:356. doi: 10.1186/1471-2105-9-356 (PMC2553347; doi:10.1186/1471-2105-9-356)
Supplement: Additional file 1 — FERN distribution, Version 1.3. This archive contains the FERN source code and binaries as well as documentation and example models in FernML and SBML. [file 1471-2105-9-356-S1.zip › fern/doc/javadoc/fern/benchmark/Benchmark.html]

Benchmark


---


|  |  |  |  |  |  |  |  |  |  |  |
| --- | --- | --- | --- | --- | --- | --- | --- | --- | --- | --- |
| |  |  |  |  |  |  |  |  | | --- | --- | --- | --- | --- | --- | --- | --- | | **Overview** | **Package** | **Class** | **Use** | **Tree** | **Deprecated** | **Index** | **Help** | | |  |
| PREV CLASS   **NEXT CLASS** | **FRAMES**    **NO FRAMES**     **All Classes** |
| SUMMARY: NESTED | FIELD | CONSTR | METHOD | DETAIL: FIELD | CONSTR | METHOD |


---


## fern.benchmark Class Benchmark

```
java.lang.Object
  fern.benchmark.Benchmark
```

**Direct Known Subclasses:**: RandomNumber, SimulatorPerformance

---

``` public abstract class Benchmark extends Object ```

Base class for all benchmark classes. Gives methods for measuring elapsed time
as well as methods for creating test sets and benchmark data handling.

**Author:**
:   Florian Erhard

---

| **Constructor Summary** | |
| --- | --- |
| `Benchmark()` |


| **Method Summary** | |
| --- | --- |
| `void` | `addData(double[] d)`             Adds benchmark data to the data pool. |
| `void` | `clearData()`             Clears all collected benchmark data. |
| `double[]` | `createRandomDoubleArray(int size, AbstractDistribution dist)`             Creates a test set containing `size` random numbers of the distribution `dist`. |
| `long` | `end()`             Gets the elapsed time since the last call of `start` in nanoseconds |
| `int` | `getNumBins()`             Gets the number of bins that are used for creating gnuplot histograms. |
| `void` | `setNumBins(int numBins)`             Sets the number of bins that are used for creating gnuplot histograms. |
| `void` | `start()`             sets a start time for the benchmark system |
| `GnuPlot` | `toGnuplot(GnuPlot gnuplot, String[] dataLabels, String[] styles)`             Adds the benchmark data without conversion to a given `GnuPlot` object. |
| `GnuPlot` | `toGnuplot(String[] dataLabels, String[] styles)`             Adds the benchmark data without conversion to a new `GnuPlot` object. |
| `GnuPlot` | `toGnuPlotAsHistogram(GnuPlot gnuplot, String[] dataLabels, String[] styles)`             Adds the benchmark data as histogram to a given `GnuPlot` object. |
| `GnuPlot` | `toGnuPlotAsHistogram(String[] dataLabels, String[] styles)`             Adds the benchmark data as histogram to a new `GnuPlot` object. |

| **Methods inherited from class java.lang.Object** |
| --- |
| `clone, equals, finalize, getClass, hashCode, notify, notifyAll, toString, wait, wait, wait` |

| **Constructor Detail** |
| --- |

### Benchmark

```
public Benchmark()
```


| **Method Detail** |
| --- |

### getNumBins

```
public int getNumBins()
```

:   Gets the number of bins that are used for creating gnuplot histograms.
    Default is 100.

    :   **Returns:**: number of bins

---


### setNumBins

```
public void setNumBins(int numBins)
```

:   Sets the number of bins that are used for creating gnuplot histograms.
    Default is 100.

    :   **Parameters:**: `numBins` - number of bins

---


### addData

```
public void addData(double[] d)
```

:   Adds benchmark data to the data pool.

    :   **Parameters:**: `d` - benchmark data

---


### clearData

```
public void clearData()
```

:   Clears all collected benchmark data.

---


### toGnuplot

```
public GnuPlot toGnuplot(String[] dataLabels,
                         String[] styles)
```

:   Adds the benchmark data without conversion to a new `GnuPlot` object.

    :   **Parameters:**: `dataLabels` - labels for the benchmark data: `styles` - styles for the benchmark data **Returns:**: a `GnuPlot` object containing the benchmark data **See Also:**: `GnuPlot`

---


### toGnuplot

```
public GnuPlot toGnuplot(GnuPlot gnuplot,
                         String[] dataLabels,
                         String[] styles)
```

:   Adds the benchmark data without conversion to a given `GnuPlot` object.

    :   **Parameters:**: `dataLabels` - labels for the benchmark data: `styles` - styles for the benchmark data **Returns:**: a `GnuPlot` object containing the benchmark data **See Also:**: `GnuPlot`

---


### toGnuPlotAsHistogram

```
public GnuPlot toGnuPlotAsHistogram(String[] dataLabels,
                                    String[] styles)
```

:   Adds the benchmark data as histogram to a new `GnuPlot` object.

    :   **Parameters:**: `dataLabels` - labels for the benchmark data: `styles` - styles for the benchmark data **Returns:**: a `GnuPlot` object containing the benchmark data **See Also:**: `GnuPlot`

---


### toGnuPlotAsHistogram

```
public GnuPlot toGnuPlotAsHistogram(GnuPlot gnuplot,
                                    String[] dataLabels,
                                    String[] styles)
```

:   Adds the benchmark data as histogram to a given `GnuPlot` object.

    :   **Parameters:**: `dataLabels` - labels for the benchmark data: `styles` - styles for the benchmark data **Returns:**: a `GnuPlot` object containing the benchmark data **See Also:**: `GnuPlot`

---


### start

```
public void start()
```

:   sets a start time for the benchmark system

---


### end

```
public long end()
```

:   Gets the elapsed time since the last call of `start` in nanoseconds

    :   **Returns:**: elapsed time in ns

---


### createRandomDoubleArray

```
public double[] createRandomDoubleArray(int size,
                                        AbstractDistribution dist)
```

:   Creates a test set containing `size` random numbers of the
    distribution `dist`.

    :   **Parameters:**: `size` - the size of the test set: `dist` - the probability distribution **Returns:**: test set


---


|  |  |  |  |  |  |  |  |  |  |  |
| --- | --- | --- | --- | --- | --- | --- | --- | --- | --- | --- |
| |  |  |  |  |  |  |  |  | | --- | --- | --- | --- | --- | --- | --- | --- | | **Overview** | **Package** | **Class** | **Use** | **Tree** | **Deprecated** | **Index** | **Help** | | |  |
| PREV CLASS   **NEXT CLASS** | **FRAMES**    **NO FRAMES**     **All Classes** |
| SUMMARY: NESTED | FIELD | CONSTR | METHOD | DETAIL: FIELD | CONSTR | METHOD |


---
