# Supplementary material for: FERN – a Java framework for stochastic simulation and evaluation of reaction networks
Source: BMC Bioinformatics. 2008 Aug 29;9:356. doi: 10.1186/1471-2105-9-356 (PMC2553347; doi:10.1186/1471-2105-9-356)
Supplement: Additional file 1 — FERN distribution, Version 1.3. This archive contains the FERN source code and binaries as well as documentation and example models in FernML and SBML. [file 1471-2105-9-356-S1.zip › fern/doc/javadoc/fern/benchmark/class-use/SimulatorPerformance.html]

Uses of Class fern.benchmark.SimulatorPerformance


---


|  |  |  |  |  |  |  |  |  |  |  |
| --- | --- | --- | --- | --- | --- | --- | --- | --- | --- | --- |
| |  |  |  |  |  |  |  |  | | --- | --- | --- | --- | --- | --- | --- | --- | | **Overview** | **Package** | **Class** | **Use** | **Tree** | **Deprecated** | **Index** | **Help** | | |  |
| PREV   NEXT | **FRAMES**    **NO FRAMES**     **All Classes** |


---


## **Uses of Class fern.benchmark.SimulatorPerformance**

| Packages that use SimulatorPerformance | |
| --- | --- |
| **fern.benchmark** | Provides classes for benchmarking simulations (time benchmarks, histogram distance calculation). |

| Uses of SimulatorPerformance in fern.benchmark | |
| --- | --- |

| Subclasses of SimulatorPerformance in fern.benchmark | |
| --- | --- |
| `class` | `SimulatorCorrectness`             Benchmark the correctness of the built-in simulators for a given net. |
| `class` | `SimulatorFireTypes`             Benchmark the `Simulator.FireType`s for a given net. |
| `class` | `SimulatorRandomNumbers`             Check the number of random number creations of different distributions for a given net. |
| `class` | `SimulatorTime` |

---


|  |  |  |  |  |  |  |  |  |  |  |
| --- | --- | --- | --- | --- | --- | --- | --- | --- | --- | --- |
| |  |  |  |  |  |  |  |  | | --- | --- | --- | --- | --- | --- | --- | --- | | **Overview** | **Package** | **Class** | **Use** | **Tree** | **Deprecated** | **Index** | **Help** | | |  |
| PREV   NEXT | **FRAMES**    **NO FRAMES**     **All Classes** |


---
