# Supplementary material for: FERN – a Java framework for stochastic simulation and evaluation of reaction networks
Source: BMC Bioinformatics. 2008 Aug 29;9:356. doi: 10.1186/1471-2105-9-356 (PMC2553347; doi:10.1186/1471-2105-9-356)
Supplement: Additional file 1 — FERN distribution, Version 1.3. This archive contains the FERN source code and binaries as well as documentation and example models in FernML and SBML. [file 1471-2105-9-356-S1.zip › fern/doc/javadoc/fern/benchmark/package-summary.html]

fern.benchmark


---


|  |  |  |  |  |  |  |  |  |  |  |
| --- | --- | --- | --- | --- | --- | --- | --- | --- | --- | --- |
| |  |  |  |  |  |  |  |  | | --- | --- | --- | --- | --- | --- | --- | --- | | **Overview** | **Package** | Class | **Use** | **Tree** | **Deprecated** | **Index** | **Help** | | |  |
| **PREV PACKAGE**   **NEXT PACKAGE** | **FRAMES**    **NO FRAMES**     **All Classes** |


---

## Package fern.benchmark

Provides classes for benchmarking simulations (time benchmarks, histogram distance calculation).

**See:**
  
          **Description**

| **Class Summary** | |
| --- | --- |
| **Benchmark** | Base class for all benchmark classes. |
| **RandomNumber** | Benchmarking of the time needed for different random number generations. |
| **SimulatorCorrectness** | Benchmark the correctness of the built-in simulators for a given net. |
| **SimulatorFireTypes** | Benchmark the `Simulator.FireType`s for a given net. |
| **SimulatorPerformance** | Measures the performance of the different simulation algorithms for a given network. |
| **SimulatorRandomNumbers** | Check the number of random number creations of different distributions for a given net. |
| **SimulatorTime** |  |

## Package fern.benchmark Description

Provides classes for benchmarking simulations (time benchmarks, histogram distance calculation).

---


|  |  |  |  |  |  |  |  |  |  |  |
| --- | --- | --- | --- | --- | --- | --- | --- | --- | --- | --- |
| |  |  |  |  |  |  |  |  | | --- | --- | --- | --- | --- | --- | --- | --- | | **Overview** | **Package** | Class | **Use** | **Tree** | **Deprecated** | **Index** | **Help** | | |  |
| **PREV PACKAGE**   **NEXT PACKAGE** | **FRAMES**    **NO FRAMES**     **All Classes** |


---
