# Supplementary material for: FERN – a Java framework for stochastic simulation and evaluation of reaction networks
Source: BMC Bioinformatics. 2008 Aug 29;9:356. doi: 10.1186/1471-2105-9-356 (PMC2553347; doi:10.1186/1471-2105-9-356)
Supplement: Additional file 1 — FERN distribution, Version 1.3. This archive contains the FERN source code and binaries as well as documentation and example models in FernML and SBML. [file 1471-2105-9-356-S1.zip › fern/doc/javadoc/fern/benchmark/RandomNumber.html]

RandomNumber


---


|  |  |  |  |  |  |  |  |  |  |  |
| --- | --- | --- | --- | --- | --- | --- | --- | --- | --- | --- |
| |  |  |  |  |  |  |  |  | | --- | --- | --- | --- | --- | --- | --- | --- | | **Overview** | **Package** | **Class** | **Use** | **Tree** | **Deprecated** | **Index** | **Help** | | |  |
| **PREV CLASS**   **NEXT CLASS** | **FRAMES**    **NO FRAMES**     **All Classes** |
| SUMMARY: NESTED | FIELD | CONSTR | METHOD | DETAIL: FIELD | CONSTR | METHOD |


---


## fern.benchmark Class RandomNumber

```
java.lang.Object
  fern.benchmark.Benchmark
      fern.benchmark.RandomNumber
```

---

``` public class RandomNumber extends Benchmark ```

Benchmarking of the time needed for different random number generations.

**Author:**
:   Florian Erhard

---

| **Constructor Summary** | |
| --- | --- |
| `RandomNumber()` |


| **Method Summary** | |
| --- | --- |
| `void` | `benchmarkLangevinAgainstTauLeapNumberGeneration(double[] at)`             Benchmark for the different computation of random numbers in the Langevin (normal distributed) and tau leap (poisson distributed) method for double values at |
| `static void` | `main(String[] args)` |

| **Methods inherited from class fern.benchmark.Benchmark** |
| --- |
| `addData, clearData, createRandomDoubleArray, end, getNumBins, setNumBins, start, toGnuplot, toGnuplot, toGnuPlotAsHistogram, toGnuPlotAsHistogram` |

| **Methods inherited from class java.lang.Object** |
| --- |
| `clone, equals, finalize, getClass, hashCode, notify, notifyAll, toString, wait, wait, wait` |

| **Constructor Detail** |
| --- |

### RandomNumber

```
public RandomNumber()
```


| **Method Detail** |
| --- |

### benchmarkLangevinAgainstTauLeapNumberGeneration

```
public void benchmarkLangevinAgainstTauLeapNumberGeneration(double[] at)
```

:   Benchmark for the different computation of random numbers in the
    Langevin (normal distributed) and tau leap (poisson distributed) method
    for double values at

    :   **Parameters:**: `at` - the products of arbitraty propensites a with arbitrary taus

---


### main

```
public static void main(String[] args)
```

:   **Parameters:**: `args` -


---


|  |  |  |  |  |  |  |  |  |  |  |
| --- | --- | --- | --- | --- | --- | --- | --- | --- | --- | --- |
| |  |  |  |  |  |  |  |  | | --- | --- | --- | --- | --- | --- | --- | --- | | **Overview** | **Package** | **Class** | **Use** | **Tree** | **Deprecated** | **Index** | **Help** | | |  |
| **PREV CLASS**   **NEXT CLASS** | **FRAMES**    **NO FRAMES**     **All Classes** |
| SUMMARY: NESTED | FIELD | CONSTR | METHOD | DETAIL: FIELD | CONSTR | METHOD |


---
