# Supplementary material for: FERN – a Java framework for stochastic simulation and evaluation of reaction networks
Source: BMC Bioinformatics. 2008 Aug 29;9:356. doi: 10.1186/1471-2105-9-356 (PMC2553347; doi:10.1186/1471-2105-9-356)
Supplement: Additional file 1 — FERN distribution, Version 1.3. This archive contains the FERN source code and binaries as well as documentation and example models in FernML and SBML. [file 1471-2105-9-356-S1.zip › fern/doc/javadoc/fern/benchmark/SimulatorCorrectness.html]

SimulatorCorrectness


---


|  |  |  |  |  |  |  |  |  |  |  |
| --- | --- | --- | --- | --- | --- | --- | --- | --- | --- | --- |
| |  |  |  |  |  |  |  |  | | --- | --- | --- | --- | --- | --- | --- | --- | | **Overview** | **Package** | **Class** | **Use** | **Tree** | **Deprecated** | **Index** | **Help** | | |  |
| **PREV CLASS**   **NEXT CLASS** | **FRAMES**    **NO FRAMES**     **All Classes** |
| SUMMARY: NESTED | FIELD | CONSTR | METHOD | DETAIL: FIELD | CONSTR | METHOD |


---


## fern.benchmark Class SimulatorCorrectness

```
java.lang.Object
  fern.benchmark.Benchmark
      fern.benchmark.SimulatorPerformance
          fern.benchmark.SimulatorCorrectness
```

---

``` public class SimulatorCorrectness extends SimulatorPerformance ```

Benchmark the correctness of the built-in simulators for a given net. For this net and a
specified moment in time, the average histogram distance is calculated for given species of the network.
Additionally the histograms are plotted. The `benchmark` method can be invoked
repeatedly to calculate the average over many simulations.

For references, see Yang Cao, Linda Petzold, Accuracy limitations and the measurement of errors in
the stochastic simulation of chemically reacting systems, Journal of Computational Physics 212 (2006) 6�24.

**Author:**
:   Florian Erhard

---

| **Field Summary** | |
| --- | --- |

| **Fields inherited from class fern.benchmark.SimulatorPerformance** |
| --- |
| `count, simulatorNames, simulators` |


| **Constructor Summary** | |
| --- | --- |
| `SimulatorCorrectness(Network net, double moment, String... speciesNames)`             Creates the benchmark instance with given network, moment in time and species. |


| **Method Summary** | |
| --- | --- |
| `protected  SimulationController` | `getController(int i)`             Returns the `SimulationController` for the base class. |
| `static void` | `main(String[] args)` |
| `void` | `present()`             Gets called after some iterations of the method `benchmark` in the base class. |

| **Methods inherited from class fern.benchmark.SimulatorPerformance** |
| --- |
| `benchmark, getShowSteps, getSimulators, setShowSteps` |

| **Methods inherited from class fern.benchmark.Benchmark** |
| --- |
| `addData, clearData, createRandomDoubleArray, end, getNumBins, setNumBins, start, toGnuplot, toGnuplot, toGnuPlotAsHistogram, toGnuPlotAsHistogram` |

| **Methods inherited from class java.lang.Object** |
| --- |
| `clone, equals, finalize, getClass, hashCode, notify, notifyAll, toString, wait, wait, wait` |

| **Constructor Detail** |
| --- |

### SimulatorCorrectness

```
public SimulatorCorrectness(Network net,
                            double moment,
                            String... speciesNames)
```

:   Creates the benchmark instance with given network, moment in time and species.

    **Parameters:**: `net` - the network to benchmark: `moment` - the moment in time at which the amounts are to be measured: `speciesNames` - the names of the species of which the amounts are to be measured


| **Method Detail** |
| --- |

### getController

```
protected SimulationController getController(int i)
```

:   Returns the `SimulationController` for the base class.

    :   **Specified by:**: `getController` in class `SimulatorPerformance`
    :   **Parameters:**: `i` - index of the `Simulator` **Returns:**: a `SimulationController` for the ith `Simulator`

---


### present

```
public void present()
```

:   Gets called after some iterations of the method `benchmark` in the base class.
    A gnuplot is created containing histograms for each species and simulator and the
    histogram distances for the simulators is printed to stdout

    :   **Specified by:**: `present` in class `SimulatorPerformance`
    :   **See Also:**: `SimulatorPerformance.setShowSteps(int)`

---


### main

```
public static void main(String[] args)
                 throws IOException,
                        JDOMException
```

:   **Throws:**: `IOException`: `JDOMException`


---


|  |  |  |  |  |  |  |  |  |  |  |
| --- | --- | --- | --- | --- | --- | --- | --- | --- | --- | --- |
| |  |  |  |  |  |  |  |  | | --- | --- | --- | --- | --- | --- | --- | --- | | **Overview** | **Package** | **Class** | **Use** | **Tree** | **Deprecated** | **Index** | **Help** | | |  |
| **PREV CLASS**   **NEXT CLASS** | **FRAMES**    **NO FRAMES**     **All Classes** |
| SUMMARY: NESTED | FIELD | CONSTR | METHOD | DETAIL: FIELD | CONSTR | METHOD |


---
