# Supplementary material for: FERN – a Java framework for stochastic simulation and evaluation of reaction networks
Source: BMC Bioinformatics. 2008 Aug 29;9:356. doi: 10.1186/1471-2105-9-356 (PMC2553347; doi:10.1186/1471-2105-9-356)
Supplement: Additional file 1 — FERN distribution, Version 1.3. This archive contains the FERN source code and binaries as well as documentation and example models in FernML and SBML. [file 1471-2105-9-356-S1.zip › fern/doc/javadoc/fern/benchmark/SimulatorPerformance.html]

SimulatorPerformance


---


|  |  |  |  |  |  |  |  |  |  |  |
| --- | --- | --- | --- | --- | --- | --- | --- | --- | --- | --- |
| |  |  |  |  |  |  |  |  | | --- | --- | --- | --- | --- | --- | --- | --- | | **Overview** | **Package** | **Class** | **Use** | **Tree** | **Deprecated** | **Index** | **Help** | | |  |
| **PREV CLASS**   **NEXT CLASS** | **FRAMES**    **NO FRAMES**     **All Classes** |
| SUMMARY: NESTED | FIELD | CONSTR | METHOD | DETAIL: FIELD | CONSTR | METHOD |


---


## fern.benchmark Class SimulatorPerformance

```
java.lang.Object
  fern.benchmark.Benchmark
      fern.benchmark.SimulatorPerformance
```

**Direct Known Subclasses:**: SimulatorCorrectness, SimulatorTime

---

``` public abstract class SimulatorPerformance extends Benchmark ```

Measures the performance of the different simulation algorithms for a given network.
This is the base class for different benchmarks and manages the simulators, the iteration
of the simulators and when results have to be presented. You just have to implement
`present` and `getController` and add some `Observer`s to the
`simulators`.

**Author:**
:   Florian Erhard

---

| **Field Summary** | |
| --- | --- |
| `protected  int` | `count`             Contains the number of iterations done for using in the `present`-method of extending classes. |
| `protected  String[]` | `simulatorNames`             Contains the names of the simulators for using in the `present`-method of extending classes. |
| `protected  Simulator[]` | `simulators`             Contains the `Simulator`s - use this field to attach `Observer`s. |


| **Constructor Summary** | |
| --- | --- |
| `SimulatorPerformance(Network net)`             Registers the six built-in simulators for the performance benchmarks. |


| **Method Summary** | |
| --- | --- |
| `void` | `benchmark()`             Performs one benchmark for each of the six simulators. |
| `protected abstract  SimulationController` | `getController(int i)`             Extending classes have to determine the `SimulationController` of each `Simulator` here. |
| `int` | `getShowSteps()`             Gets the number of iterations between two `present`-calls. |
| `Simulator[]` | `getSimulators()`             Gets the simulators used by this benchmark. |
| `protected abstract  void` | `present()`             Is called after `getShowSteps` iterations. |
| `void` | `setShowSteps(int showSteps)`             Sets the number of iterations between two `present`-calls. |

| **Methods inherited from class fern.benchmark.Benchmark** |
| --- |
| `addData, clearData, createRandomDoubleArray, end, getNumBins, setNumBins, start, toGnuplot, toGnuplot, toGnuPlotAsHistogram, toGnuPlotAsHistogram` |

| **Methods inherited from class java.lang.Object** |
| --- |
| `clone, equals, finalize, getClass, hashCode, notify, notifyAll, toString, wait, wait, wait` |

| **Field Detail** |
| --- |

### simulators

```
protected Simulator[] simulators
```

:   Contains the `Simulator`s - use this field to attach `Observer`s.

---


### simulatorNames

```
protected String[] simulatorNames
```

:   Contains the names of the simulators for using in the `present`-method of extending classes.

---


### count

```
protected int count
```

:   Contains the number of iterations done for using in the `present`-method of extending classes.


| **Constructor Detail** |
| --- |

### SimulatorPerformance

```
public SimulatorPerformance(Network net)
```

:   Registers the six built-in simulators for the performance benchmarks.

    **Parameters:**: `net` - the network to benchmark **See Also:**: `GillespieSimple`, `GillespieEnhanced`, `GibsonBruckSimulator`, `TauLeapingAbsoluteBoundSimulator`, `TauLeapingRelativeBoundSimulator`, `TauLeapingSpeciesPopulationBoundSimulator`


| **Method Detail** |
| --- |

### benchmark

```
public void benchmark()
```

:   Performs one benchmark for each of the six simulators. To minimize effects that could
    compromise time benchmarks like caching, the order of the simulators is randomized
    each time. The time needed for each algorithm is recorded and added to `Benchmark`'s
    data pool. After each `getShowSteps()` iterations, `present`
    is called.

    :   **See Also:**: `Benchmark.addData(double[])`

---


### getShowSteps

```
public int getShowSteps()
```

:   Gets the number of iterations between two `present`-calls.

    :   **Returns:**: the showSteps

---


### setShowSteps

```
public void setShowSteps(int showSteps)
```

:   Sets the number of iterations between two `present`-calls.

    :   **Parameters:**: `showSteps` - the showSteps to set

---


### getSimulators

```
public Simulator[] getSimulators()
```

:   Gets the simulators used by this benchmark.

    :   **Returns:**: simulators.

---


### getController

```
protected abstract SimulationController getController(int i)
```

:   Extending classes have to determine the `SimulationController` of each
    `Simulator` here.

    :   **Parameters:**: `i` - index of the simulator **Returns:**: a `SimulationController` for the ith simulator

---


### present

```
protected abstract void present()
```

:   Is called after `getShowSteps` iterations. Implement your benchmark
    presentation here.


---


|  |  |  |  |  |  |  |  |  |  |  |
| --- | --- | --- | --- | --- | --- | --- | --- | --- | --- | --- |
| |  |  |  |  |  |  |  |  | | --- | --- | --- | --- | --- | --- | --- | --- | | **Overview** | **Package** | **Class** | **Use** | **Tree** | **Deprecated** | **Index** | **Help** | | |  |
| **PREV CLASS**   **NEXT CLASS** | **FRAMES**    **NO FRAMES**     **All Classes** |
| SUMMARY: NESTED | FIELD | CONSTR | METHOD | DETAIL: FIELD | CONSTR | METHOD |


---
