# Supplementary material for: FERN – a Java framework for stochastic simulation and evaluation of reaction networks
Source: BMC Bioinformatics. 2008 Aug 29;9:356. doi: 10.1186/1471-2105-9-356 (PMC2553347; doi:10.1186/1471-2105-9-356)
Supplement: Additional file 1 — FERN distribution, Version 1.3. This archive contains the FERN source code and binaries as well as documentation and example models in FernML and SBML. [file 1471-2105-9-356-S1.zip › fern/doc/javadoc/fern/benchmark/SimulatorRandomNumbers.html]

SimulatorRandomNumbers


---


|  |  |  |  |  |  |  |  |  |  |  |
| --- | --- | --- | --- | --- | --- | --- | --- | --- | --- | --- |
| |  |  |  |  |  |  |  |  | | --- | --- | --- | --- | --- | --- | --- | --- | | **Overview** | **Package** | **Class** | **Use** | **Tree** | **Deprecated** | **Index** | **Help** | | |  |
| **PREV CLASS**   **NEXT CLASS** | **FRAMES**    **NO FRAMES**     **All Classes** |
| SUMMARY: NESTED | FIELD | CONSTR | METHOD | DETAIL: FIELD | CONSTR | METHOD |


---


## fern.benchmark Class SimulatorRandomNumbers

```
java.lang.Object
  fern.benchmark.Benchmark
      fern.benchmark.SimulatorPerformance
          fern.benchmark.SimulatorTime
              fern.benchmark.SimulatorRandomNumbers
```

---

``` public class SimulatorRandomNumbers extends SimulatorTime ```

Check the number of random number creations of different distributions for a given net.
The random number creations are essential for the performance of the simulation algorithms.
The `benchmark` method can be invoked
repeatedly to calculate the average over many simulations.

**Author:**
:   Florian Erhard

---

| **Field Summary** | |
| --- | --- |

| **Fields inherited from class fern.benchmark.SimulatorPerformance** |
| --- |
| `count, simulatorNames, simulators` |


| **Constructor Summary** | |
| --- | --- |
| `SimulatorRandomNumbers(Network net, double time)`             Create the benchmark and defines the time each simulator has to run in one iteration. |


| **Method Summary** | |
| --- | --- |
| `protected  SimulationController` | `getController(int i)`             Returns the `SimulationController` for the base class. |
| `void` | `present()`             Present results of this benchmark is gnuplot and text to stdout. |

| **Methods inherited from class fern.benchmark.SimulatorPerformance** |
| --- |
| `benchmark, getShowSteps, getSimulators, setShowSteps` |

| **Methods inherited from class fern.benchmark.Benchmark** |
| --- |
| `addData, clearData, createRandomDoubleArray, end, getNumBins, setNumBins, start, toGnuplot, toGnuplot, toGnuPlotAsHistogram, toGnuPlotAsHistogram` |

| **Methods inherited from class java.lang.Object** |
| --- |
| `clone, equals, finalize, getClass, hashCode, notify, notifyAll, toString, wait, wait, wait` |

| **Constructor Detail** |
| --- |

### SimulatorRandomNumbers

```
public SimulatorRandomNumbers(Network net,
                              double time)
```

:   Create the benchmark and defines the time each simulator has to run in one iteration.

    **Parameters:**: `net` - the network to benchmark: `time` - running time for the simulators


| **Method Detail** |
| --- |

### getController

```
protected SimulationController getController(int i)
```

:   Returns the `SimulationController` for the base class.

    :   **Overrides:**: `getController` in class `SimulatorTime`
    :   **Parameters:**: `i` - index of the `Simulator` **Returns:**: a `SimulationController` for the ith `Simulator`

---


### present

```
public void present()
```

:   Present results of this benchmark is gnuplot and text to stdout.

    :   **Overrides:**: `present` in class `SimulatorTime`


---


|  |  |  |  |  |  |  |  |  |  |  |
| --- | --- | --- | --- | --- | --- | --- | --- | --- | --- | --- |
| |  |  |  |  |  |  |  |  | | --- | --- | --- | --- | --- | --- | --- | --- | | **Overview** | **Package** | **Class** | **Use** | **Tree** | **Deprecated** | **Index** | **Help** | | |  |
| **PREV CLASS**   **NEXT CLASS** | **FRAMES**    **NO FRAMES**     **All Classes** |
| SUMMARY: NESTED | FIELD | CONSTR | METHOD | DETAIL: FIELD | CONSTR | METHOD |


---
