# Supplementary material for: FERN – a Java framework for stochastic simulation and evaluation of reaction networks
Source: BMC Bioinformatics. 2008 Aug 29;9:356. doi: 10.1186/1471-2105-9-356 (PMC2553347; doi:10.1186/1471-2105-9-356)
Supplement: Additional file 1 — FERN distribution, Version 1.3. This archive contains the FERN source code and binaries as well as documentation and example models in FernML and SBML. [file 1471-2105-9-356-S1.zip › fern/doc/javadoc/fern/cellDesigner/CellDesignerNetworkWrapper.html]

CellDesignerNetworkWrapper


---


|  |  |  |  |  |  |  |  |  |  |  |
| --- | --- | --- | --- | --- | --- | --- | --- | --- | --- | --- |
| |  |  |  |  |  |  |  |  | | --- | --- | --- | --- | --- | --- | --- | --- | | **Overview** | **Package** | **Class** | **Use** | **Tree** | **Deprecated** | **Index** | **Help** | | |  |
| PREV CLASS   **NEXT CLASS** | **FRAMES**    **NO FRAMES**     **All Classes** |
| SUMMARY: NESTED | FIELD | CONSTR | METHOD | DETAIL: FIELD | CONSTR | METHOD |


---


## fern.cellDesigner Class CellDesignerNetworkWrapper

```
java.lang.Object
  fern.network.AbstractNetworkImpl
      fern.cellDesigner.CellDesignerNetworkWrapper
```

**All Implemented Interfaces:**: Network

---

``` public class CellDesignerNetworkWrapper extends AbstractNetworkImpl ```

---

| **Field Summary** | |
| --- | --- |

| **Fields inherited from class fern.network.AbstractNetworkImpl** |
| --- |
| `adjListPro, adjListRea, amountManager, annotationManager, indexToSpeciesId, name, propensitiyCalculator, speciesIdToIndex` |


| **Constructor Summary** | |
| --- | --- |
| `CellDesignerNetworkWrapper(PluginModel model)` |


| **Method Summary** | |
| --- | --- |
| `protected  void` | `createAdjacencyLists()`             Reminds extending class to fill `AbstractNetworkImpl.adjListPro` and `AbstractNetworkImpl.adjListRea`. |
| `protected  void` | `createAmountManager()`             Reminds extending class to fill `AbstractNetworkImpl.amountManager`. |
| `protected  void` | `createAnnotationManager()`             Reminds extending class to fill `AbstractNetworkImpl.annotationManager`. |
| `protected  void` | `createPropensityCalulator()`             Reminds extending class to fill `AbstractNetworkImpl.propensitiyCalculator`. |
| `protected  void` | `createSpeciesMapping()`             Reminds extending class to fill `AbstractNetworkImpl.speciesIdToIndex` and `AbstractNetworkImpl.indexToSpeciesId`. |
| `long` | `getInitialAmount(int species)`             Gets the initial amount of the specified molecule species. |
| `void` | `setInitialAmount(int species, long value)`             Sets the initial amount of the specified molecule species. |

| **Methods inherited from class fern.network.AbstractNetworkImpl** |
| --- |
| `getAmountManager, getAnnotationManager, getName, getNumReactions, getNumSpecies, getProducts, getPropensityCalculator, getReactants, getReactionName, getSpeciesByName, getSpeciesMapping, getSpeciesName` |

| **Methods inherited from class java.lang.Object** |
| --- |
| `clone, equals, finalize, getClass, hashCode, notify, notifyAll, toString, wait, wait, wait` |

| **Constructor Detail** |
| --- |

### CellDesignerNetworkWrapper

```
public CellDesignerNetworkWrapper(PluginModel model)
```


| **Method Detail** |
| --- |

### createAdjacencyLists

```
protected void createAdjacencyLists()
```

:   **Description copied from class: `AbstractNetworkImpl`**
:   Reminds extending class to fill `AbstractNetworkImpl.adjListPro` and `AbstractNetworkImpl.adjListRea`.

    :   **Specified by:**: `createAdjacencyLists` in class `AbstractNetworkImpl`

---


### createAmountManager

```
protected void createAmountManager()
```

:   **Description copied from class: `AbstractNetworkImpl`**
:   Reminds extending class to fill `AbstractNetworkImpl.amountManager`.

    :   **Specified by:**: `createAmountManager` in class `AbstractNetworkImpl`

---


### createAnnotationManager

```
protected void createAnnotationManager()
```

:   **Description copied from class: `AbstractNetworkImpl`**
:   Reminds extending class to fill `AbstractNetworkImpl.annotationManager`.

    :   **Specified by:**: `createAnnotationManager` in class `AbstractNetworkImpl`

---


### createPropensityCalulator

```
protected void createPropensityCalulator()
```

:   **Description copied from class: `AbstractNetworkImpl`**
:   Reminds extending class to fill `AbstractNetworkImpl.propensitiyCalculator`.

    :   **Specified by:**: `createPropensityCalulator` in class `AbstractNetworkImpl`

---


### createSpeciesMapping

```
protected void createSpeciesMapping()
```

:   **Description copied from class: `AbstractNetworkImpl`**
:   Reminds extending class to fill `AbstractNetworkImpl.speciesIdToIndex` and `AbstractNetworkImpl.indexToSpeciesId`.

    :   **Specified by:**: `createSpeciesMapping` in class `AbstractNetworkImpl`

---


### getInitialAmount

```
public long getInitialAmount(int species)
```

:   **Description copied from interface: `Network`**
:   Gets the initial amount of the specified molecule species.

    :   **Parameters:**: `species` - index of the species **Returns:**: initial amount of the species

---


### setInitialAmount

```
public void setInitialAmount(int species,
                             long value)
```

:   **Description copied from interface: `Network`**
:   Sets the initial amount of the specified molecule species.

    :   **Parameters:**: `species` - index of the species: `value` - initial amount of the species


---


|  |  |  |  |  |  |  |  |  |  |  |
| --- | --- | --- | --- | --- | --- | --- | --- | --- | --- | --- |
| |  |  |  |  |  |  |  |  | | --- | --- | --- | --- | --- | --- | --- | --- | | **Overview** | **Package** | **Class** | **Use** | **Tree** | **Deprecated** | **Index** | **Help** | | |  |
| PREV CLASS   **NEXT CLASS** | **FRAMES**    **NO FRAMES**     **All Classes** |
| SUMMARY: NESTED | FIELD | CONSTR | METHOD | DETAIL: FIELD | CONSTR | METHOD |


---
