# Supplementary material for: FERN – a Java framework for stochastic simulation and evaluation of reaction networks
Source: BMC Bioinformatics. 2008 Aug 29;9:356. doi: 10.1186/1471-2105-9-356 (PMC2553347; doi:10.1186/1471-2105-9-356)
Supplement: Additional file 1 — FERN distribution, Version 1.3. This archive contains the FERN source code and binaries as well as documentation and example models in FernML and SBML. [file 1471-2105-9-356-S1.zip › fern/doc/javadoc/fern/cellDesigner/CellDesignerPropensityCalculator.html]

CellDesignerPropensityCalculator


---


|  |  |  |  |  |  |  |  |  |  |  |
| --- | --- | --- | --- | --- | --- | --- | --- | --- | --- | --- |
| |  |  |  |  |  |  |  |  | | --- | --- | --- | --- | --- | --- | --- | --- | | **Overview** | **Package** | **Class** | **Use** | **Tree** | **Deprecated** | **Index** | **Help** | | |  |
| **PREV CLASS**   **NEXT CLASS** | **FRAMES**    **NO FRAMES**     **All Classes** |
| SUMMARY: NESTED | FIELD | CONSTR | METHOD | DETAIL: FIELD | CONSTR | METHOD |


---


## fern.cellDesigner Class CellDesignerPropensityCalculator

```
java.lang.Object
  fern.network.sbml.SBMLPropensityCalculator
      fern.cellDesigner.CellDesignerPropensityCalculator
```

**All Implemented Interfaces:**: ComplexDependenciesPropensityCalculator, PropensityCalculator

---

``` public class CellDesignerPropensityCalculator extends SBMLPropensityCalculator ```

Propensity calculator which is used for `SBMLNetwork`s. The propensities are
calculated by using a `MathTree` derived by the MathML representation of the
kinetic law for each reaction.

**Author:**
:   Florian Erhard

---

| **Constructor Summary** | |
| --- | --- |
| `CellDesignerPropensityCalculator(PluginModel model, CellDesignerNetworkWrapper net)`             Creates the `MathTree`s and parses the parameters. |


| **Method Summary** | |
| --- | --- |
| `double` | `calculatePropensity(int reaction, AmountManager amount, Simulator sim)`             Calculates the propensity for a reaction given the amounts of the `AmountManager`. |
| `Map<String,Double>` | `getGlobalParameters()`             Gets the global parameters. |
| `List<Integer>` | `getKineticLawSpecies(int reaction)`             Gets the indices of the species that are included in the calculation of the given reaction. |

| **Methods inherited from class fern.network.sbml.SBMLPropensityCalculator** |
| --- |
| `getMathTree` |

| **Methods inherited from class java.lang.Object** |
| --- |
| `clone, equals, finalize, getClass, hashCode, notify, notifyAll, toString, wait, wait, wait` |

| **Constructor Detail** |
| --- |

### CellDesignerPropensityCalculator

```
public CellDesignerPropensityCalculator(PluginModel model,
                                        CellDesignerNetworkWrapper net)
```

:   Creates the `MathTree`s and parses the parameters.

    **Parameters:**: `net` - sbml netowrk


| **Method Detail** |
| --- |

### getGlobalParameters

```
public Map<String,Double> getGlobalParameters()
```

:   Gets the global parameters.

    :   **Overrides:**: `getGlobalParameters` in class `SBMLPropensityCalculator`
    :   **Returns:**: global parameters

---


### calculatePropensity

```
public double calculatePropensity(int reaction,
                                  AmountManager amount,
                                  Simulator sim)
```

:   **Description copied from interface: `PropensityCalculator`**
:   Calculates the propensity for a reaction given the amounts of the `AmountManager`.
    If a positive
    value for volume is given, it is assumed that the constants are deterministic rate
    constants and are hence to be transformed to specific reaction rate constants.

    :   **Specified by:**: `calculatePropensity` in interface `PropensityCalculator` **Overrides:**: `calculatePropensity` in class `SBMLPropensityCalculator`
    :   **Parameters:**: `reaction` - index of the reaction: `amount` - AmountManager: `sim` - Simulator **Returns:**: actual propensity of the reaction

---


### getKineticLawSpecies

```
public List<Integer> getKineticLawSpecies(int reaction)
```

:   **Description copied from interface: `ComplexDependenciesPropensityCalculator`**
:   Gets the indices of the species that are included in the calculation of
    the given reaction.

    :   **Specified by:**: `getKineticLawSpecies` in interface `ComplexDependenciesPropensityCalculator` **Overrides:**: `getKineticLawSpecies` in class `SBMLPropensityCalculator`
    :   **Parameters:**: `reaction` - index of the reaction **Returns:**: indices of the species included in the reaction's kinetic law


---


|  |  |  |  |  |  |  |  |  |  |  |
| --- | --- | --- | --- | --- | --- | --- | --- | --- | --- | --- |
| |  |  |  |  |  |  |  |  | | --- | --- | --- | --- | --- | --- | --- | --- | | **Overview** | **Package** | **Class** | **Use** | **Tree** | **Deprecated** | **Index** | **Help** | | |  |
| **PREV CLASS**   **NEXT CLASS** | **FRAMES**    **NO FRAMES**     **All Classes** |
| SUMMARY: NESTED | FIELD | CONSTR | METHOD | DETAIL: FIELD | CONSTR | METHOD |


---
