# Supplementary material for: FERN – a Java framework for stochastic simulation and evaluation of reaction networks
Source: BMC Bioinformatics. 2008 Aug 29;9:356. doi: 10.1186/1471-2105-9-356 (PMC2553347; doi:10.1186/1471-2105-9-356)
Supplement: Additional file 1 — FERN distribution, Version 1.3. This archive contains the FERN source code and binaries as well as documentation and example models in FernML and SBML. [file 1471-2105-9-356-S1.zip › fern/doc/javadoc/fern/cellDesigner/class-use/CellDesignerNetworkWrapper.html]

Uses of Class fern.cellDesigner.CellDesignerNetworkWrapper


---


|  |  |  |  |  |  |  |  |  |  |  |
| --- | --- | --- | --- | --- | --- | --- | --- | --- | --- | --- |
| |  |  |  |  |  |  |  |  | | --- | --- | --- | --- | --- | --- | --- | --- | | **Overview** | **Package** | **Class** | **Use** | **Tree** | **Deprecated** | **Index** | **Help** | | |  |
| PREV   NEXT | **FRAMES**    **NO FRAMES**     **All Classes** |


---


## **Uses of Class fern.cellDesigner.CellDesignerNetworkWrapper**

| Packages that use CellDesignerNetworkWrapper | |
| --- | --- |
| **fern.cellDesigner** |  |
| **fern.cellDesigner.ui** |  |

| Uses of CellDesignerNetworkWrapper in fern.cellDesigner | |
| --- | --- |

| Constructors in fern.cellDesigner with parameters of type CellDesignerNetworkWrapper | |
| --- | --- |
| `CellDesignerPropensityCalculator(PluginModel model, CellDesignerNetworkWrapper net)`             Creates the `MathTree`s and parses the parameters. |

| Uses of CellDesignerNetworkWrapper in fern.cellDesigner.ui | |
| --- | --- |

| Methods in fern.cellDesigner.ui that return CellDesignerNetworkWrapper | |
| --- | --- |
| `CellDesignerNetworkWrapper` | `MainFrame.getNetwork()` |

---


|  |  |  |  |  |  |  |  |  |  |  |
| --- | --- | --- | --- | --- | --- | --- | --- | --- | --- | --- |
| |  |  |  |  |  |  |  |  | | --- | --- | --- | --- | --- | --- | --- | --- | | **Overview** | **Package** | **Class** | **Use** | **Tree** | **Deprecated** | **Index** | **Help** | | |  |
| PREV   NEXT | **FRAMES**    **NO FRAMES**     **All Classes** |


---
