# Supplementary material for: FERN – a Java framework for stochastic simulation and evaluation of reaction networks
Source: BMC Bioinformatics. 2008 Aug 29;9:356. doi: 10.1186/1471-2105-9-356 (PMC2553347; doi:10.1186/1471-2105-9-356)
Supplement: Additional file 1 — FERN distribution, Version 1.3. This archive contains the FERN source code and binaries as well as documentation and example models in FernML and SBML. [file 1471-2105-9-356-S1.zip › fern/doc/javadoc/fern/cellDesigner/FernCellDesignerPlugin.html]

FernCellDesignerPlugin


---


|  |  |  |  |  |  |  |  |  |  |  |
| --- | --- | --- | --- | --- | --- | --- | --- | --- | --- | --- |
| |  |  |  |  |  |  |  |  | | --- | --- | --- | --- | --- | --- | --- | --- | | **Overview** | **Package** | **Class** | **Use** | **Tree** | **Deprecated** | **Index** | **Help** | | |  |
| **PREV CLASS**   NEXT CLASS | **FRAMES**    **NO FRAMES**     **All Classes** |
| SUMMARY: NESTED | FIELD | CONSTR | METHOD | DETAIL: FIELD | CONSTR | METHOD |


---


## fern.cellDesigner Class FernCellDesignerPlugin

```
java.lang.Object
  CellDesignerPlugin
      fern.cellDesigner.FernCellDesignerPlugin
```

---

``` public class FernCellDesignerPlugin extends CellDesignerPlugin ```

---

| **Constructor Summary** | |
| --- | --- |
| `FernCellDesignerPlugin()` |


| **Method Summary** | |
| --- | --- |
| `void` | `addPluginMenu()` |
| `void` | `modelClosed(PluginSBase arg0)` |
| `void` | `modelOpened(PluginSBase arg0)` |
| `void` | `modelSelectChanged(PluginSBase model)` |
| `void` | `SBaseAdded(PluginSBase arg0)` |
| `void` | `SBaseChanged(PluginSBase arg0)` |
| `void` | `SBaseDeleted(PluginSBase arg0)` |

| **Methods inherited from class java.lang.Object** |
| --- |
| `clone, equals, finalize, getClass, hashCode, notify, notifyAll, toString, wait, wait, wait` |

| **Constructor Detail** |
| --- |

### FernCellDesignerPlugin

```
public FernCellDesignerPlugin()
```


| **Method Detail** |
| --- |

### SBaseAdded

```
public void SBaseAdded(PluginSBase arg0)
```

---


### SBaseChanged

```
public void SBaseChanged(PluginSBase arg0)
```

---


### SBaseDeleted

```
public void SBaseDeleted(PluginSBase arg0)
```

---


### addPluginMenu

```
public void addPluginMenu()
```

---


### modelClosed

```
public void modelClosed(PluginSBase arg0)
```

---


### modelOpened

```
public void modelOpened(PluginSBase arg0)
```

---


### modelSelectChanged

```
public void modelSelectChanged(PluginSBase model)
```


---


|  |  |  |  |  |  |  |  |  |  |  |
| --- | --- | --- | --- | --- | --- | --- | --- | --- | --- | --- |
| |  |  |  |  |  |  |  |  | | --- | --- | --- | --- | --- | --- | --- | --- | | **Overview** | **Package** | **Class** | **Use** | **Tree** | **Deprecated** | **Index** | **Help** | | |  |
| **PREV CLASS**   NEXT CLASS | **FRAMES**    **NO FRAMES**     **All Classes** |
| SUMMARY: NESTED | FIELD | CONSTR | METHOD | DETAIL: FIELD | CONSTR | METHOD |


---
