# Supplementary material for: FERN – a Java framework for stochastic simulation and evaluation of reaction networks
Source: BMC Bioinformatics. 2008 Aug 29;9:356. doi: 10.1186/1471-2105-9-356 (PMC2553347; doi:10.1186/1471-2105-9-356)
Supplement: Additional file 1 — FERN distribution, Version 1.3. This archive contains the FERN source code and binaries as well as documentation and example models in FernML and SBML. [file 1471-2105-9-356-S1.zip › fern/doc/javadoc/fern/cytoscape/class-use/ColorCalculator.Scale.html]

Uses of Class fern.cytoscape.ColorCalculator.Scale


---


|  |  |  |  |  |  |  |  |  |  |  |
| --- | --- | --- | --- | --- | --- | --- | --- | --- | --- | --- |
| |  |  |  |  |  |  |  |  | | --- | --- | --- | --- | --- | --- | --- | --- | | **Overview** | **Package** | **Class** | **Use** | **Tree** | **Deprecated** | **Index** | **Help** | | |  |
| PREV   NEXT | **FRAMES**    **NO FRAMES**     **All Classes** |


---


## **Uses of Class fern.cytoscape.ColorCalculator.Scale**

| Packages that use ColorCalculator.Scale | |
| --- | --- |
| **fern.cytoscape** | Provides the classes for the cytoscape plugin. |

| Uses of ColorCalculator.Scale in fern.cytoscape | |
| --- | --- |

| Methods in fern.cytoscape that return ColorCalculator.Scale | |
| --- | --- |
| `ColorCalculator.Scale` | `ColorCalculator.getScale()` |
| `static ColorCalculator.Scale` | `ColorCalculator.Scale.valueOf(String name)`             Returns the enum constant of this type with the specified name. |
| `static ColorCalculator.Scale[]` | `ColorCalculator.Scale.values()`             Returns an array containing the constants of this enum type, in the order they are declared. |

| Methods in fern.cytoscape with parameters of type ColorCalculator.Scale | |
| --- | --- |
| `void` | `ColorCalculator.setScale(ColorCalculator.Scale scale)` |

---


|  |  |  |  |  |  |  |  |  |  |  |
| --- | --- | --- | --- | --- | --- | --- | --- | --- | --- | --- |
| |  |  |  |  |  |  |  |  | | --- | --- | --- | --- | --- | --- | --- | --- | | **Overview** | **Package** | **Class** | **Use** | **Tree** | **Deprecated** | **Index** | **Help** | | |  |
| PREV   NEXT | **FRAMES**    **NO FRAMES**     **All Classes** |


---
