# Supplementary material for: FERN – a Java framework for stochastic simulation and evaluation of reaction networks
Source: BMC Bioinformatics. 2008 Aug 29;9:356. doi: 10.1186/1471-2105-9-356 (PMC2553347; doi:10.1186/1471-2105-9-356)
Supplement: Additional file 1 — FERN distribution, Version 1.3. This archive contains the FERN source code and binaries as well as documentation and example models in FernML and SBML. [file 1471-2105-9-356-S1.zip › fern/doc/javadoc/fern/cytoscape/class-use/FernVisualStyle.html]

Uses of Class fern.cytoscape.FernVisualStyle


---


|  |  |  |  |  |  |  |  |  |  |  |
| --- | --- | --- | --- | --- | --- | --- | --- | --- | --- | --- |
| |  |  |  |  |  |  |  |  | | --- | --- | --- | --- | --- | --- | --- | --- | | **Overview** | **Package** | **Class** | **Use** | **Tree** | **Deprecated** | **Index** | **Help** | | |  |
| PREV   NEXT | **FRAMES**    **NO FRAMES**     **All Classes** |


---


## **Uses of Class fern.cytoscape.FernVisualStyle**

| Packages that use FernVisualStyle | |
| --- | --- |
| **fern.cytoscape** | Provides the classes for the cytoscape plugin. |
| **fern.cytoscape.ui** |  |

| Uses of FernVisualStyle in fern.cytoscape | |
| --- | --- |

| Constructors in fern.cytoscape with parameters of type FernVisualStyle | |
| --- | --- |
| `CytoscapeColorChangeObserver(boolean visualize, boolean showTrendSteps, Simulator sim, CytoscapeNetworkWrapper net, FernVisualStyle style, String[] trendSpecies, double time)` |

| Uses of FernVisualStyle in fern.cytoscape.ui | |
| --- | --- |

| Constructors in fern.cytoscape.ui with parameters of type FernVisualStyle | |
| --- | --- |
| `MainFrame(FernVisualStyle style)` |

---


|  |  |  |  |  |  |  |  |  |  |  |
| --- | --- | --- | --- | --- | --- | --- | --- | --- | --- | --- |
| |  |  |  |  |  |  |  |  | | --- | --- | --- | --- | --- | --- | --- | --- | | **Overview** | **Package** | **Class** | **Use** | **Tree** | **Deprecated** | **Index** | **Help** | | |  |
| PREV   NEXT | **FRAMES**    **NO FRAMES**     **All Classes** |


---
