# Supplementary material for: FERN – a Java framework for stochastic simulation and evaluation of reaction networks
Source: BMC Bioinformatics. 2008 Aug 29;9:356. doi: 10.1186/1471-2105-9-356 (PMC2553347; doi:10.1186/1471-2105-9-356)
Supplement: Additional file 1 — FERN distribution, Version 1.3. This archive contains the FERN source code and binaries as well as documentation and example models in FernML and SBML. [file 1471-2105-9-356-S1.zip › fern/doc/javadoc/fern/cytoscape/class-use/NetworkChecker.NodeClassifier.html]

Uses of Interface fern.cytoscape.NetworkChecker.NodeClassifier


---


|  |  |  |  |  |  |  |  |  |  |  |
| --- | --- | --- | --- | --- | --- | --- | --- | --- | --- | --- |
| |  |  |  |  |  |  |  |  | | --- | --- | --- | --- | --- | --- | --- | --- | | **Overview** | **Package** | **Class** | **Use** | **Tree** | **Deprecated** | **Index** | **Help** | | |  |
| PREV   NEXT | **FRAMES**    **NO FRAMES**     **All Classes** |


---


## **Uses of Interface fern.cytoscape.NetworkChecker.NodeClassifier**

| Packages that use NetworkChecker.NodeClassifier | |
| --- | --- |
| **fern.cytoscape** | Provides the classes for the cytoscape plugin. |

| Uses of NetworkChecker.NodeClassifier in fern.cytoscape | |
| --- | --- |

| Classes in fern.cytoscape that implement NetworkChecker.NodeClassifier | |
| --- | --- |
| `class` | `NetworkChecker.NodeClassifierByAnnotation<T>` |

| Methods in fern.cytoscape that return NetworkChecker.NodeClassifier | |
| --- | --- |
| `NetworkChecker.NodeClassifier` | `NetworkChecker.getNodeClassifier()` |

---


|  |  |  |  |  |  |  |  |  |  |  |
| --- | --- | --- | --- | --- | --- | --- | --- | --- | --- | --- |
| |  |  |  |  |  |  |  |  | | --- | --- | --- | --- | --- | --- | --- | --- | | **Overview** | **Package** | **Class** | **Use** | **Tree** | **Deprecated** | **Index** | **Help** | | |  |
| PREV   NEXT | **FRAMES**    **NO FRAMES**     **All Classes** |


---
