# Supplementary material for: FERN – a Java framework for stochastic simulation and evaluation of reaction networks
Source: BMC Bioinformatics. 2008 Aug 29;9:356. doi: 10.1186/1471-2105-9-356 (PMC2553347; doi:10.1186/1471-2105-9-356)
Supplement: Additional file 1 — FERN distribution, Version 1.3. This archive contains the FERN source code and binaries as well as documentation and example models in FernML and SBML. [file 1471-2105-9-356-S1.zip › fern/doc/javadoc/fern/cytoscape/ColorCalculator.html]

ColorCalculator


---


|  |  |  |  |  |  |  |  |  |  |  |
| --- | --- | --- | --- | --- | --- | --- | --- | --- | --- | --- |
| |  |  |  |  |  |  |  |  | | --- | --- | --- | --- | --- | --- | --- | --- | | **Overview** | **Package** | **Class** | **Use** | **Tree** | **Deprecated** | **Index** | **Help** | | |  |
| PREV CLASS   **NEXT CLASS** | **FRAMES**    **NO FRAMES**     **All Classes** |
| SUMMARY: NESTED | FIELD | CONSTR | METHOD | DETAIL: FIELD | CONSTR | METHOD |


---


## fern.cytoscape Class ColorCalculator

```
java.lang.Object
  fern.cytoscape.ColorCalculator
```

**All Implemented Interfaces:**: Cloneable

---

``` public class ColorCalculator extends Object implements Cloneable ```

---

| **Nested Class Summary** | |
| --- | --- |
| `static class` | `ColorCalculator.Scale` |


| **Constructor Summary** | |
| --- | --- |
| `ColorCalculator()` |


| **Method Summary** | |
| --- | --- |
| `Object` | `clone()` |
| `Color` | `getAmountBottomColor()` |
| `Color` | `getAmountTopColor()` |
| `Color` | `getColor(double d, double max)` |
| `Color` | `getReactionColor()` |
| `ColorCalculator.Scale` | `getScale()` |
| `double` | `getScaleMax()` |
| `void` | `setAmountBottomColor(Color amountBottomColor)` |
| `void` | `setAmountTopColor(Color amountTopColor)` |
| `void` | `setReactionColor(Color reactionColor)` |
| `void` | `setScale(ColorCalculator.Scale scale)` |
| `void` | `setScaleMax(double scaleMax)` |

| **Methods inherited from class java.lang.Object** |
| --- |
| `equals, finalize, getClass, hashCode, notify, notifyAll, toString, wait, wait, wait` |

| **Constructor Detail** |
| --- |

### ColorCalculator

```
public ColorCalculator()
```


| **Method Detail** |
| --- |

### clone

```
public Object clone()
             throws CloneNotSupportedException
```

:   **Overrides:**: `clone` in class `Object`
:   **Throws:**: `CloneNotSupportedException`

---


### getReactionColor

```
public Color getReactionColor()
```

---


### setReactionColor

```
public void setReactionColor(Color reactionColor)
```

---


### getAmountBottomColor

```
public Color getAmountBottomColor()
```

---


### setAmountBottomColor

```
public void setAmountBottomColor(Color amountBottomColor)
```

---


### getAmountTopColor

```
public Color getAmountTopColor()
```

---


### setAmountTopColor

```
public void setAmountTopColor(Color amountTopColor)
```

---


### getScale

```
public ColorCalculator.Scale getScale()
```

---


### setScale

```
public void setScale(ColorCalculator.Scale scale)
```

---


### getScaleMax

```
public double getScaleMax()
```

---


### setScaleMax

```
public void setScaleMax(double scaleMax)
```

---


### getColor

```
public Color getColor(double d,
                      double max)
```


---


|  |  |  |  |  |  |  |  |  |  |  |
| --- | --- | --- | --- | --- | --- | --- | --- | --- | --- | --- |
| |  |  |  |  |  |  |  |  | | --- | --- | --- | --- | --- | --- | --- | --- | | **Overview** | **Package** | **Class** | **Use** | **Tree** | **Deprecated** | **Index** | **Help** | | |  |
| PREV CLASS   **NEXT CLASS** | **FRAMES**    **NO FRAMES**     **All Classes** |
| SUMMARY: NESTED | FIELD | CONSTR | METHOD | DETAIL: FIELD | CONSTR | METHOD |


---
