# Supplementary material for: FERN – a Java framework for stochastic simulation and evaluation of reaction networks
Source: BMC Bioinformatics. 2008 Aug 29;9:356. doi: 10.1186/1471-2105-9-356 (PMC2553347; doi:10.1186/1471-2105-9-356)
Supplement: Additional file 1 — FERN distribution, Version 1.3. This archive contains the FERN source code and binaries as well as documentation and example models in FernML and SBML. [file 1471-2105-9-356-S1.zip › fern/doc/javadoc/fern/cytoscape/CytoscapeColorChangeObserver.ColorChangingNodeAppeareanceCalculator.html]

CytoscapeColorChangeObserver.ColorChangingNodeAppeareanceCalculator


---


|  |  |  |  |  |  |  |  |  |  |  |
| --- | --- | --- | --- | --- | --- | --- | --- | --- | --- | --- |
| |  |  |  |  |  |  |  |  | | --- | --- | --- | --- | --- | --- | --- | --- | | **Overview** | **Package** | **Class** | **Use** | **Tree** | **Deprecated** | **Index** | **Help** | | |  |
| **PREV CLASS**   **NEXT CLASS** | **FRAMES**    **NO FRAMES**     **All Classes** |
| SUMMARY: NESTED | FIELD | CONSTR | METHOD | DETAIL: FIELD | CONSTR | METHOD |


---


## fern.cytoscape Class CytoscapeColorChangeObserver.ColorChangingNodeAppeareanceCalculator

```
java.lang.Object
  cytoscape.visual.NodeAppearanceCalculator
      fern.cytoscape.CytoscapeColorChangeObserver.ColorChangingNodeAppeareanceCalculator
```

**All Implemented Interfaces:**: Cloneable

**Enclosing class:**: CytoscapeColorChangeObserver

---

``` protected static class CytoscapeColorChangeObserver.ColorChangingNodeAppeareanceCalculator extends cytoscape.visual.NodeAppearanceCalculator ```

---

| **Field Summary** | |
| --- | --- |
| `protected  cytoscape.visual.Appearance` | `tmpDefaultAppearance` |

| **Fields inherited from class cytoscape.visual.NodeAppearanceCalculator** |
| --- |
| `nodeBorderColorBypass, nodeFillColorBypass, nodeFontBypass, nodeHeightBypass, nodeLabelBypass, nodeLabelColorBypass, nodeLineTypeBypass, nodeShapeBypass, nodeToolTipBypass, nodeWidthBypass` |


| **Constructor Summary** | |
| --- | --- |
| `CytoscapeColorChangeObserver.ColorChangingNodeAppeareanceCalculator(cytoscape.visual.NodeAppearanceCalculator parent)` |


| **Method Summary** | |
| --- | --- |
| `protected  void` | `applyProperties(cytoscape.visual.Appearance arg0, String arg1, Properties arg2, String arg3, cytoscape.visual.CalculatorCatalog arg4)` |
| `void` | `calculateNodeAppearance(cytoscape.visual.NodeAppearance app, giny.model.Node node, cytoscape.CyNetwork net)` |
| `Object` | `clone()` |
| `cytoscape.visual.calculators.Calculator` | `getCalculator(byte arg0)` |
| `List<cytoscape.visual.calculators.Calculator>` | `getCalculators()` |
| `protected  String` | `getDescription(String arg0, cytoscape.visual.Appearance arg1)` |
| `protected  Properties` | `getProperties(cytoscape.visual.Appearance arg0, String arg1)` |
| `void` | `removeCalculator(byte arg0)` |
| `void` | `setCalculator(cytoscape.visual.calculators.Calculator arg0)` |
| `void` | `setColor(giny.model.Node n, Color c)` |
| `void` | `unsetColor(giny.model.Node n)` |

| **Methods inherited from class cytoscape.visual.NodeAppearanceCalculator** |
| --- |
| `applyProperties, calculateNodeAppearance, calculateNodeBorderColor, calculateNodeFillColor, calculateNodeFont, calculateNodeHeight, calculateNodeLabel, calculateNodeLabelColor, calculateNodeLineType, calculateNodeShape, calculateNodeToolTip, calculateNodeWidth, copyDefaultAppearance, getDefaultAppearance, getDefaultNodeBorderColor, getDefaultNodeFillColor, getDefaultNodeFont, getDefaultNodeFontFace, getDefaultNodeFontSize, getDefaultNodeHeight, getDefaultNodeLabel, getDefaultNodeLabelColor, getDefaultNodeLineType, getDefaultNodeShape, getDefaultNodeToolTip, getDefaultNodeWidth, getDescription, getNodeBorderColorCalculator, getNodeFillColorCalculator, getNodeFontFaceCalculator, getNodeFontSizeCalculator, getNodeHeightCalculator, getNodeLabelCalculator, getNodeLabelColorCalculator, getNodeLineTypeCalculator, getNodeShapeCalculator, getNodeSizeLocked, getNodeToolTipCalculator, getNodeWidthCalculator, getProperties, isValidCalculator, setDefaultAppearance, setDefaultNodeBorderColor, setDefaultNodeFillColor, setDefaultNodeFont, setDefaultNodeFontFace, setDefaultNodeFontSize, setDefaultNodeHeight, setDefaultNodeLabel, setDefaultNodeLabelColor, setDefaultNodeLineType, setDefaultNodeShape, setDefaultNodeToolTip, setDefaultNodeWidth, setNodeBorderColorCalculator, setNodeFillColorCalculator, setNodeFontFaceCalculator, setNodeFontSizeCalculator, setNodeHeightCalculator, setNodeLabelCalculator, setNodeLabelColorCalculator, setNodeLineTypeCalculator, setNodeShapeCalculator, setNodeSizeLocked, setNodeToolTipCalculator, setNodeWidthCalculator` |

| **Methods inherited from class java.lang.Object** |
| --- |
| `equals, finalize, getClass, hashCode, notify, notifyAll, toString, wait, wait, wait` |

| **Field Detail** |
| --- |

### tmpDefaultAppearance

```
protected cytoscape.visual.Appearance tmpDefaultAppearance
```


| **Constructor Detail** |
| --- |

### CytoscapeColorChangeObserver.ColorChangingNodeAppeareanceCalculator

```
public CytoscapeColorChangeObserver.ColorChangingNodeAppeareanceCalculator(cytoscape.visual.NodeAppearanceCalculator parent)
```


| **Method Detail** |
| --- |

### calculateNodeAppearance

```
public void calculateNodeAppearance(cytoscape.visual.NodeAppearance app,
                                    giny.model.Node node,
                                    cytoscape.CyNetwork net)
```

:   **Overrides:**: `calculateNodeAppearance` in class `cytoscape.visual.NodeAppearanceCalculator`

---


### setColor

```
public void setColor(giny.model.Node n,
                     Color c)
```

---


### unsetColor

```
public void unsetColor(giny.model.Node n)
```

---


### clone

```
public Object clone()
```

:   **Overrides:**: `clone` in class `Object`

---


### getCalculator

```
public cytoscape.visual.calculators.Calculator getCalculator(byte arg0)
```

---


### getCalculators

```
public List<cytoscape.visual.calculators.Calculator> getCalculators()
```

---


### removeCalculator

```
public void removeCalculator(byte arg0)
```

---


### setCalculator

```
public void setCalculator(cytoscape.visual.calculators.Calculator arg0)
```

---


### getDescription

```
protected String getDescription(String arg0,
                                cytoscape.visual.Appearance arg1)
```

---


### applyProperties

```
protected void applyProperties(cytoscape.visual.Appearance arg0,
                               String arg1,
                               Properties arg2,
                               String arg3,
                               cytoscape.visual.CalculatorCatalog arg4)
```

---


### getProperties

```
protected Properties getProperties(cytoscape.visual.Appearance arg0,
                                   String arg1)
```


---


|  |  |  |  |  |  |  |  |  |  |  |
| --- | --- | --- | --- | --- | --- | --- | --- | --- | --- | --- |
| |  |  |  |  |  |  |  |  | | --- | --- | --- | --- | --- | --- | --- | --- | | **Overview** | **Package** | **Class** | **Use** | **Tree** | **Deprecated** | **Index** | **Help** | | |  |
| **PREV CLASS**   **NEXT CLASS** | **FRAMES**    **NO FRAMES**     **All Classes** |
| SUMMARY: NESTED | FIELD | CONSTR | METHOD | DETAIL: FIELD | CONSTR | METHOD |


---
