# Supplementary material for: FERN – a Java framework for stochastic simulation and evaluation of reaction networks
Source: BMC Bioinformatics. 2008 Aug 29;9:356. doi: 10.1186/1471-2105-9-356 (PMC2553347; doi:10.1186/1471-2105-9-356)
Supplement: Additional file 1 — FERN distribution, Version 1.3. This archive contains the FERN source code and binaries as well as documentation and example models in FernML and SBML. [file 1471-2105-9-356-S1.zip › fern/doc/javadoc/fern/cytoscape/CytoscapeColorChangeObserver.html]

CytoscapeColorChangeObserver


---


|  |  |  |  |  |  |  |  |  |  |  |
| --- | --- | --- | --- | --- | --- | --- | --- | --- | --- | --- |
| |  |  |  |  |  |  |  |  | | --- | --- | --- | --- | --- | --- | --- | --- | | **Overview** | **Package** | **Class** | **Use** | **Tree** | **Deprecated** | **Index** | **Help** | | |  |
| **PREV CLASS**   **NEXT CLASS** | **FRAMES**    **NO FRAMES**     **All Classes** |
| SUMMARY: NESTED | FIELD | CONSTR | METHOD | DETAIL: FIELD | CONSTR | METHOD |


---


## fern.cytoscape Class CytoscapeColorChangeObserver

```
java.lang.Object
  fern.simulation.observer.Observer
      fern.cytoscape.CytoscapeColorChangeObserver
```

---

``` public class CytoscapeColorChangeObserver extends Observer ```

---

| **Nested Class Summary** | |
| --- | --- |
| `protected static class` | `CytoscapeColorChangeObserver.ColorChangingNodeAppeareanceCalculator` |


| **Constructor Summary** | |
| --- | --- |
| `CytoscapeColorChangeObserver(boolean visualize, boolean showTrendSteps, Simulator sim, CytoscapeNetworkWrapper net, FernVisualStyle style, String[] trendSpecies, double time)` |


| **Method Summary** | |
| --- | --- |
| `void` | `activateReaction(int mu, double tau, Simulator.FireType fireType, int times)`             Gets called before a reaction fires. |
| `void` | `finished()`             Gets called when a simulation has finished, directly after the termination check. |
| `void` | `started()`             Gets called when the simulation has started after the initialization and before the termination condition is checked the first time. |
| `void` | `step()`             Gets called after each termination check and before `Simulator.performStep(fern.simulation.controller.SimulationController)` is called. |
| `void` | `theta(double theta)`             Gets called by simulators when a certain moment in time is reached. |

| **Methods inherited from class fern.simulation.observer.Observer** |
| --- |
| `applyLabelFormat, getLabel, getLabelFormat, getNumSimulations, getPrintWriter, getSimulator, getTheta, getTitlesCommand, print, setLabelFormat, setPrintWriter, setSimulator, setTheta` |

| **Methods inherited from class java.lang.Object** |
| --- |
| `clone, equals, finalize, getClass, hashCode, notify, notifyAll, toString, wait, wait, wait` |

| **Constructor Detail** |
| --- |

### CytoscapeColorChangeObserver

```
public CytoscapeColorChangeObserver(boolean visualize,
                                    boolean showTrendSteps,
                                    Simulator sim,
                                    CytoscapeNetworkWrapper net,
                                    FernVisualStyle style,
                                    String[] trendSpecies,
                                    double time)
```


| **Method Detail** |
| --- |

### finished

```
public void finished()
```

:   **Description copied from class: `Observer`**
:   Gets called when a simulation has finished, directly after the termination check.

    :   **Specified by:**: `finished` in class `Observer`

---


### started

```
public void started()
```

:   **Description copied from class: `Observer`**
:   Gets called when the simulation has started after the initialization and before
    the termination condition is checked the first time.

    :   **Specified by:**: `started` in class `Observer`

---


### step

```
public void step()
```

:   **Description copied from class: `Observer`**
:   Gets called after each termination check and before `Simulator.performStep(fern.simulation.controller.SimulationController)`
    is called.

    :   **Specified by:**: `step` in class `Observer`

---


### activateReaction

```
public void activateReaction(int mu,
                             double tau,
                             Simulator.FireType fireType,
                             int times)
```

:   **Description copied from class: `Observer`**
:   Gets called before a reaction fires.

    :   **Specified by:**: `activateReaction` in class `Observer`
    :   **Parameters:**: `mu` - the reaction which is supposed to fire: `tau` - the time the reaction fires (at this time `Simulator.getTime()` does not necessarily yield the firing time): `fireType` - the type of the firing: `times` - TODO

---


### theta

```
public void theta(double theta)
```

:   **Description copied from class: `Observer`**
:   Gets called by simulators when a certain moment in time is reached. This moment in
    time has to be registered by `Observer.getTheta()`

    :   **Specified by:**: `theta` in class `Observer`
    :   **Parameters:**: `theta` - moment in time


---


|  |  |  |  |  |  |  |  |  |  |  |
| --- | --- | --- | --- | --- | --- | --- | --- | --- | --- | --- |
| |  |  |  |  |  |  |  |  | | --- | --- | --- | --- | --- | --- | --- | --- | | **Overview** | **Package** | **Class** | **Use** | **Tree** | **Deprecated** | **Index** | **Help** | | |  |
| **PREV CLASS**   **NEXT CLASS** | **FRAMES**    **NO FRAMES**     **All Classes** |
| SUMMARY: NESTED | FIELD | CONSTR | METHOD | DETAIL: FIELD | CONSTR | METHOD |


---
