# Supplementary material for: FERN – a Java framework for stochastic simulation and evaluation of reaction networks
Source: BMC Bioinformatics. 2008 Aug 29;9:356. doi: 10.1186/1471-2105-9-356 (PMC2553347; doi:10.1186/1471-2105-9-356)
Supplement: Additional file 1 — FERN distribution, Version 1.3. This archive contains the FERN source code and binaries as well as documentation and example models in FernML and SBML. [file 1471-2105-9-356-S1.zip › fern/doc/javadoc/fern/cytoscape/CytoscapeVisualizer.html]

CytoscapeVisualizer


---


|  |  |  |  |  |  |  |  |  |  |  |
| --- | --- | --- | --- | --- | --- | --- | --- | --- | --- | --- |
| |  |  |  |  |  |  |  |  | | --- | --- | --- | --- | --- | --- | --- | --- | | **Overview** | **Package** | **Class** | **Use** | **Tree** | **Deprecated** | **Index** | **Help** | | |  |
| **PREV CLASS**   **NEXT CLASS** | **FRAMES**    **NO FRAMES**     **All Classes** |
| SUMMARY: NESTED | FIELD | CONSTR | METHOD | DETAIL: FIELD | CONSTR | METHOD |


---


## fern.cytoscape Class CytoscapeVisualizer

```
java.lang.Object
  cytoscape.plugin.CytoscapePlugin
      fern.cytoscape.CytoscapeVisualizer
```

**All Implemented Interfaces:**: PropertyChangeListener, EventListener

---

``` public class CytoscapeVisualizer extends cytoscape.plugin.CytoscapePlugin ```

---

| **Nested Class Summary** | |
| --- | --- |
| `class` | `CytoscapeVisualizer.ShowMainFrameAction` |
| `class` | `CytoscapeVisualizer.SimulationAction` |


| **Constructor Summary** | |
| --- | --- |
| `CytoscapeVisualizer()` |


| **Method Summary** | |
| --- | --- |
| `String` | `describe()` |

| **Methods inherited from class cytoscape.plugin.CytoscapePlugin** |
| --- |
| `activate, deactivate, getScriptName, interpretScript, isScriptable, loadPlugin, propertyChange, restoreSessionState, saveSessionStateFiles` |

| **Methods inherited from class java.lang.Object** |
| --- |
| `clone, equals, finalize, getClass, hashCode, notify, notifyAll, toString, wait, wait, wait` |

| **Constructor Detail** |
| --- |

### CytoscapeVisualizer

```
public CytoscapeVisualizer()
```


| **Method Detail** |
| --- |

### describe

```
public String describe()
```

:   **Overrides:**: `describe` in class `cytoscape.plugin.CytoscapePlugin`


---


|  |  |  |  |  |  |  |  |  |  |  |
| --- | --- | --- | --- | --- | --- | --- | --- | --- | --- | --- |
| |  |  |  |  |  |  |  |  | | --- | --- | --- | --- | --- | --- | --- | --- | | **Overview** | **Package** | **Class** | **Use** | **Tree** | **Deprecated** | **Index** | **Help** | | |  |
| **PREV CLASS**   **NEXT CLASS** | **FRAMES**    **NO FRAMES**     **All Classes** |
| SUMMARY: NESTED | FIELD | CONSTR | METHOD | DETAIL: FIELD | CONSTR | METHOD |


---
