# Supplementary material for: FERN – a Java framework for stochastic simulation and evaluation of reaction networks
Source: BMC Bioinformatics. 2008 Aug 29;9:356. doi: 10.1186/1471-2105-9-356 (PMC2553347; doi:10.1186/1471-2105-9-356)
Supplement: Additional file 1 — FERN distribution, Version 1.3. This archive contains the FERN source code and binaries as well as documentation and example models in FernML and SBML. [file 1471-2105-9-356-S1.zip › fern/doc/javadoc/fern/cytoscape/CytoscapeVisualizer.ShowMainFrameAction.html]

CytoscapeVisualizer.ShowMainFrameAction


---


|  |  |  |  |  |  |  |  |  |  |  |
| --- | --- | --- | --- | --- | --- | --- | --- | --- | --- | --- |
| |  |  |  |  |  |  |  |  | | --- | --- | --- | --- | --- | --- | --- | --- | | **Overview** | **Package** | **Class** | **Use** | **Tree** | **Deprecated** | **Index** | **Help** | | |  |
| **PREV CLASS**   **NEXT CLASS** | **FRAMES**    **NO FRAMES**     **All Classes** |
| SUMMARY: NESTED | FIELD | CONSTR | METHOD | DETAIL: FIELD | CONSTR | METHOD |


---


## fern.cytoscape Class CytoscapeVisualizer.ShowMainFrameAction

```
java.lang.Object
  javax.swing.AbstractAction
      cytoscape.util.CytoscapeAction
          fern.cytoscape.CytoscapeVisualizer.ShowMainFrameAction
```

**All Implemented Interfaces:**: ActionListener, Serializable, Cloneable, EventListener, Action

**Enclosing class:**: CytoscapeVisualizer

---

``` public class CytoscapeVisualizer.ShowMainFrameAction extends cytoscape.util.CytoscapeAction ```

**See Also:**: Serialized Form

---

| **Field Summary** | |
| --- | --- |

| **Fields inherited from class cytoscape.util.CytoscapeAction** |
| --- |
| `acceleratorSet, consoleName, keyCode, keyModifiers, menuIndex, preferredButtonGroup, preferredMenu` |

| **Fields inherited from class javax.swing.AbstractAction** |
| --- |
| `changeSupport, enabled` |

| **Fields inherited from interface javax.swing.Action** |
| --- |
| `ACCELERATOR_KEY, ACTION_COMMAND_KEY, DEFAULT, DISPLAYED_MNEMONIC_INDEX_KEY, LARGE_ICON_KEY, LONG_DESCRIPTION, MNEMONIC_KEY, NAME, SELECTED_KEY, SHORT_DESCRIPTION, SMALL_ICON` |


| **Constructor Summary** | |
| --- | --- |
| `CytoscapeVisualizer.ShowMainFrameAction()` |


| **Method Summary** | |
| --- | --- |
| `void` | `actionPerformed(ActionEvent arg0)` |

| **Methods inherited from class cytoscape.util.CytoscapeAction** |
| --- |
| `actionHelp, clone, completions, getActionList, getKeyCode, getKeyModifiers, getName, getPreferredButtonGroup, getPreferredMenu, getPrefferedIndex, initialize, isAccelerated, isInMenuBar, isInToolBar, setAcceleratorCombo, setName, setPreferredButtonGroup, setPreferredIndex, setPreferredMenu, takeArgs` |

| **Methods inherited from class javax.swing.AbstractAction** |
| --- |
| `addPropertyChangeListener, firePropertyChange, getKeys, getPropertyChangeListeners, getValue, isEnabled, putValue, removePropertyChangeListener, setEnabled` |

| **Methods inherited from class java.lang.Object** |
| --- |
| `equals, finalize, getClass, hashCode, notify, notifyAll, toString, wait, wait, wait` |

| **Constructor Detail** |
| --- |

### CytoscapeVisualizer.ShowMainFrameAction

```
public CytoscapeVisualizer.ShowMainFrameAction()
```


| **Method Detail** |
| --- |

### actionPerformed

```
public void actionPerformed(ActionEvent arg0)
```

:   **Specified by:**: `actionPerformed` in interface `ActionListener` **Specified by:**: `actionPerformed` in class `cytoscape.util.CytoscapeAction`


---


|  |  |  |  |  |  |  |  |  |  |  |
| --- | --- | --- | --- | --- | --- | --- | --- | --- | --- | --- |
| |  |  |  |  |  |  |  |  | | --- | --- | --- | --- | --- | --- | --- | --- | | **Overview** | **Package** | **Class** | **Use** | **Tree** | **Deprecated** | **Index** | **Help** | | |  |
| **PREV CLASS**   **NEXT CLASS** | **FRAMES**    **NO FRAMES**     **All Classes** |
| SUMMARY: NESTED | FIELD | CONSTR | METHOD | DETAIL: FIELD | CONSTR | METHOD |


---
