# Supplementary material for: FERN – a Java framework for stochastic simulation and evaluation of reaction networks
Source: BMC Bioinformatics. 2008 Aug 29;9:356. doi: 10.1186/1471-2105-9-356 (PMC2553347; doi:10.1186/1471-2105-9-356)
Supplement: Additional file 1 — FERN distribution, Version 1.3. This archive contains the FERN source code and binaries as well as documentation and example models in FernML and SBML. [file 1471-2105-9-356-S1.zip › fern/doc/javadoc/fern/cytoscape/FernVisualStyle.html]

FernVisualStyle


---


|  |  |  |  |  |  |  |  |  |  |  |
| --- | --- | --- | --- | --- | --- | --- | --- | --- | --- | --- |
| |  |  |  |  |  |  |  |  | | --- | --- | --- | --- | --- | --- | --- | --- | | **Overview** | **Package** | **Class** | **Use** | **Tree** | **Deprecated** | **Index** | **Help** | | |  |
| **PREV CLASS**   **NEXT CLASS** | **FRAMES**    **NO FRAMES**     **All Classes** |
| SUMMARY: NESTED | FIELD | CONSTR | METHOD | DETAIL: FIELD | CONSTR | METHOD |


---


## fern.cytoscape Class FernVisualStyle

```
java.lang.Object
  cytoscape.visual.VisualStyle
      fern.cytoscape.FernVisualStyle
```

**All Implemented Interfaces:**: Cloneable

---

``` public class FernVisualStyle extends cytoscape.visual.VisualStyle ```

---

| **Field Summary** | |
| --- | --- |
| `static ColorCalculator` | `colorCalculator` |

| **Fields inherited from class cytoscape.visual.VisualStyle** |
| --- |
| `dupeCount` |


| **Constructor Summary** | |
| --- | --- |
| `FernVisualStyle()` |


| **Method Summary** | |
| --- | --- |
| `void` | `resetColors()` |
| `void` | `setNetworkChecker(NetworkChecker networkChecker)` |
| `void` | `setReactionFire(giny.model.Node node)` |
| `void` | `setReactionUnFire(giny.model.Node node)` |
| `void` | `setValue(giny.model.Node node, double val, double max)` |

| **Methods inherited from class cytoscape.visual.VisualStyle** |
| --- |
| `checkConflictingCalculator, clone, getDupeCount, getEdgeAppearanceCalculator, getGlobalAppearanceCalculator, getName, getNodeAppearanceCalculator, setEdgeAppearanceCalculator, setGlobalAppearanceCalculator, setName, setNodeAppearanceCalculator, toString` |

| **Methods inherited from class java.lang.Object** |
| --- |
| `equals, finalize, getClass, hashCode, notify, notifyAll, wait, wait, wait` |

| **Field Detail** |
| --- |

### colorCalculator

```
public static ColorCalculator colorCalculator
```


| **Constructor Detail** |
| --- |

### FernVisualStyle

```
public FernVisualStyle()
```


| **Method Detail** |
| --- |

### setNetworkChecker

```
public void setNetworkChecker(NetworkChecker networkChecker)
```

---


### setValue

```
public void setValue(giny.model.Node node,
                     double val,
                     double max)
```

---


### setReactionFire

```
public void setReactionFire(giny.model.Node node)
```

---


### setReactionUnFire

```
public void setReactionUnFire(giny.model.Node node)
```

---


### resetColors

```
public void resetColors()
```


---


|  |  |  |  |  |  |  |  |  |  |  |
| --- | --- | --- | --- | --- | --- | --- | --- | --- | --- | --- |
| |  |  |  |  |  |  |  |  | | --- | --- | --- | --- | --- | --- | --- | --- | | **Overview** | **Package** | **Class** | **Use** | **Tree** | **Deprecated** | **Index** | **Help** | | |  |
| **PREV CLASS**   **NEXT CLASS** | **FRAMES**    **NO FRAMES**     **All Classes** |
| SUMMARY: NESTED | FIELD | CONSTR | METHOD | DETAIL: FIELD | CONSTR | METHOD |


---
