# Supplementary material for: FERN – a Java framework for stochastic simulation and evaluation of reaction networks
Source: BMC Bioinformatics. 2008 Aug 29;9:356. doi: 10.1186/1471-2105-9-356 (PMC2553347; doi:10.1186/1471-2105-9-356)
Supplement: Additional file 1 — FERN distribution, Version 1.3. This archive contains the FERN source code and binaries as well as documentation and example models in FernML and SBML. [file 1471-2105-9-356-S1.zip › fern/doc/javadoc/fern/cytoscape/NetworkChecker.EdgeClassifier.html]

NetworkChecker.EdgeClassifier


---


|  |  |  |  |  |  |  |  |  |  |  |
| --- | --- | --- | --- | --- | --- | --- | --- | --- | --- | --- |
| |  |  |  |  |  |  |  |  | | --- | --- | --- | --- | --- | --- | --- | --- | | **Overview** | **Package** | **Class** | **Use** | **Tree** | **Deprecated** | **Index** | **Help** | | |  |
| **PREV CLASS**   **NEXT CLASS** | **FRAMES**    **NO FRAMES**     **All Classes** |
| SUMMARY: NESTED | FIELD | CONSTR | METHOD | DETAIL: FIELD | CONSTR | METHOD |


---


## fern.cytoscape Interface NetworkChecker.EdgeClassifier

**All Known Implementing Classes:**: NetworkChecker.EdgeClassifierByDirection, NetworkChecker.EdgeClassifierByIdentifier

**Enclosing class:**: NetworkChecker

---

``` public static interface NetworkChecker.EdgeClassifier ```

---

| **Method Summary** | |
| --- | --- |
| `boolean` | `isReactionToProductEdge(giny.model.Edge e)` |
| `boolean` | `isReactionToReactantEdge(giny.model.Edge e)` |
| `boolean` | `isUsable()` |

| **Method Detail** |
| --- |

### isReactionToProductEdge

```
boolean isReactionToProductEdge(giny.model.Edge e)
```

---


### isReactionToReactantEdge

```
boolean isReactionToReactantEdge(giny.model.Edge e)
```

---


### isUsable

```
boolean isUsable()
```


---


|  |  |  |  |  |  |  |  |  |  |  |
| --- | --- | --- | --- | --- | --- | --- | --- | --- | --- | --- |
| |  |  |  |  |  |  |  |  | | --- | --- | --- | --- | --- | --- | --- | --- | | **Overview** | **Package** | **Class** | **Use** | **Tree** | **Deprecated** | **Index** | **Help** | | |  |
| **PREV CLASS**   **NEXT CLASS** | **FRAMES**    **NO FRAMES**     **All Classes** |
| SUMMARY: NESTED | FIELD | CONSTR | METHOD | DETAIL: FIELD | CONSTR | METHOD |


---
