# Supplementary material for: FERN – a Java framework for stochastic simulation and evaluation of reaction networks
Source: BMC Bioinformatics. 2008 Aug 29;9:356. doi: 10.1186/1471-2105-9-356 (PMC2553347; doi:10.1186/1471-2105-9-356)
Supplement: Additional file 1 — FERN distribution, Version 1.3. This archive contains the FERN source code and binaries as well as documentation and example models in FernML and SBML. [file 1471-2105-9-356-S1.zip › fern/doc/javadoc/fern/cytoscape/NetworkChecker.EdgeClassifierByDirection.html]

NetworkChecker.EdgeClassifierByDirection


---


|  |  |  |  |  |  |  |  |  |  |  |
| --- | --- | --- | --- | --- | --- | --- | --- | --- | --- | --- |
| |  |  |  |  |  |  |  |  | | --- | --- | --- | --- | --- | --- | --- | --- | | **Overview** | **Package** | **Class** | **Use** | **Tree** | **Deprecated** | **Index** | **Help** | | |  |
| **PREV CLASS**   **NEXT CLASS** | **FRAMES**    **NO FRAMES**     **All Classes** |
| SUMMARY: NESTED | FIELD | CONSTR | METHOD | DETAIL: FIELD | CONSTR | METHOD |


---


## fern.cytoscape Class NetworkChecker.EdgeClassifierByDirection

```
java.lang.Object
  fern.cytoscape.NetworkChecker.EdgeClassifierByDirection
```

**All Implemented Interfaces:**: NetworkChecker.EdgeClassifier

**Enclosing class:**: NetworkChecker

---

``` public class NetworkChecker.EdgeClassifierByDirection extends Object implements NetworkChecker.EdgeClassifier ```

---

| **Constructor Summary** | |
| --- | --- |
| `NetworkChecker.EdgeClassifierByDirection()` |


| **Method Summary** | |
| --- | --- |
| `boolean` | `isReactionToProductEdge(giny.model.Edge e)` |
| `boolean` | `isReactionToReactantEdge(giny.model.Edge e)` |
| `boolean` | `isUsable()` |

| **Methods inherited from class java.lang.Object** |
| --- |
| `clone, equals, finalize, getClass, hashCode, notify, notifyAll, toString, wait, wait, wait` |

| **Constructor Detail** |
| --- |

### NetworkChecker.EdgeClassifierByDirection

```
public NetworkChecker.EdgeClassifierByDirection()
```


| **Method Detail** |
| --- |

### isReactionToProductEdge

```
public boolean isReactionToProductEdge(giny.model.Edge e)
```

:   **Specified by:**: `isReactionToProductEdge` in interface `NetworkChecker.EdgeClassifier`

---


### isReactionToReactantEdge

```
public boolean isReactionToReactantEdge(giny.model.Edge e)
```

:   **Specified by:**: `isReactionToReactantEdge` in interface `NetworkChecker.EdgeClassifier`

---


### isUsable

```
public boolean isUsable()
```

:   **Specified by:**: `isUsable` in interface `NetworkChecker.EdgeClassifier`


---


|  |  |  |  |  |  |  |  |  |  |  |
| --- | --- | --- | --- | --- | --- | --- | --- | --- | --- | --- |
| |  |  |  |  |  |  |  |  | | --- | --- | --- | --- | --- | --- | --- | --- | | **Overview** | **Package** | **Class** | **Use** | **Tree** | **Deprecated** | **Index** | **Help** | | |  |
| **PREV CLASS**   **NEXT CLASS** | **FRAMES**    **NO FRAMES**     **All Classes** |
| SUMMARY: NESTED | FIELD | CONSTR | METHOD | DETAIL: FIELD | CONSTR | METHOD |


---
