# Supplementary material for: FERN – a Java framework for stochastic simulation and evaluation of reaction networks
Source: BMC Bioinformatics. 2008 Aug 29;9:356. doi: 10.1186/1471-2105-9-356 (PMC2553347; doi:10.1186/1471-2105-9-356)
Supplement: Additional file 1 — FERN distribution, Version 1.3. This archive contains the FERN source code and binaries as well as documentation and example models in FernML and SBML. [file 1471-2105-9-356-S1.zip › fern/doc/javadoc/fern/cytoscape/NetworkChecker.html]

NetworkChecker


---


|  |  |  |  |  |  |  |  |  |  |  |
| --- | --- | --- | --- | --- | --- | --- | --- | --- | --- | --- |
| |  |  |  |  |  |  |  |  | | --- | --- | --- | --- | --- | --- | --- | --- | | **Overview** | **Package** | **Class** | **Use** | **Tree** | **Deprecated** | **Index** | **Help** | | |  |
| **PREV CLASS**   **NEXT CLASS** | **FRAMES**    **NO FRAMES**     **All Classes** |
| SUMMARY: NESTED | FIELD | CONSTR | METHOD | DETAIL: FIELD | CONSTR | METHOD |


---


## fern.cytoscape Class NetworkChecker

```
java.lang.Object
  fern.cytoscape.NetworkChecker
```

---

``` public class NetworkChecker extends Object ```

---

| **Nested Class Summary** | |
| --- | --- |
| `static interface` | `NetworkChecker.EdgeClassifier` |
| `class` | `NetworkChecker.EdgeClassifierByDirection` |
| `class` | `NetworkChecker.EdgeClassifierByIdentifier` |
| `static interface` | `NetworkChecker.NodeClassifier` |
| `class` | `NetworkChecker.NodeClassifierByAnnotation<T>` |
| `class` | `NetworkChecker.NodeParameter` |


| **Field Summary** | |
| --- | --- |
| `String` | `coefficient` |
| `String` | `initialAmount` |
| `String` | `nodeType` |
| `Object` | `nodeTypeReaction` |
| `Object` | `nodeTypeSpecies` |


| **Constructor Summary** | |
| --- | --- |
| `NetworkChecker()` |
| `NetworkChecker(String nodeType, Object nodeTypeReaction, Object nodeTypeSpecies, String coefficient, String initialAmount)` |


| **Method Summary** | |
| --- | --- |
| `void` | `check()` |
| `NetworkChecker.EdgeClassifier` | `getEdgeClassifier()` |
| `NetworkChecker.NodeClassifier` | `getNodeClassifier()` |
| `NetworkChecker.NodeParameter` | `getNodeParameter()` |
| `boolean` | `isValid()` |

| **Methods inherited from class java.lang.Object** |
| --- |
| `clone, equals, finalize, getClass, hashCode, notify, notifyAll, toString, wait, wait, wait` |

| **Field Detail** |
| --- |

### nodeType

```
public String nodeType
```

---


### nodeTypeReaction

```
public Object nodeTypeReaction
```

---


### nodeTypeSpecies

```
public Object nodeTypeSpecies
```

---


### coefficient

```
public String coefficient
```

---


### initialAmount

```
public String initialAmount
```


| **Constructor Detail** |
| --- |

### NetworkChecker

```
public NetworkChecker()
```

---


### NetworkChecker

```
public NetworkChecker(String nodeType,
                      Object nodeTypeReaction,
                      Object nodeTypeSpecies,
                      String coefficient,
                      String initialAmount)
```


| **Method Detail** |
| --- |

### isValid

```
public boolean isValid()
```

---


### check

```
public void check()
```

---


### getNodeClassifier

```
public NetworkChecker.NodeClassifier getNodeClassifier()
```

---


### getEdgeClassifier

```
public NetworkChecker.EdgeClassifier getEdgeClassifier()
```

---


### getNodeParameter

```
public NetworkChecker.NodeParameter getNodeParameter()
```


---


|  |  |  |  |  |  |  |  |  |  |  |
| --- | --- | --- | --- | --- | --- | --- | --- | --- | --- | --- |
| |  |  |  |  |  |  |  |  | | --- | --- | --- | --- | --- | --- | --- | --- | | **Overview** | **Package** | **Class** | **Use** | **Tree** | **Deprecated** | **Index** | **Help** | | |  |
| **PREV CLASS**   **NEXT CLASS** | **FRAMES**    **NO FRAMES**     **All Classes** |
| SUMMARY: NESTED | FIELD | CONSTR | METHOD | DETAIL: FIELD | CONSTR | METHOD |


---
