# Supplementary material for: FERN – a Java framework for stochastic simulation and evaluation of reaction networks
Source: BMC Bioinformatics. 2008 Aug 29;9:356. doi: 10.1186/1471-2105-9-356 (PMC2553347; doi:10.1186/1471-2105-9-356)
Supplement: Additional file 1 — FERN distribution, Version 1.3. This archive contains the FERN source code and binaries as well as documentation and example models in FernML and SBML. [file 1471-2105-9-356-S1.zip › fern/doc/javadoc/fern/cytoscape/NetworkChecker.NodeClassifierByAnnotation.html]

NetworkChecker.NodeClassifierByAnnotation


---


|  |  |  |  |  |  |  |  |  |  |  |
| --- | --- | --- | --- | --- | --- | --- | --- | --- | --- | --- |
| |  |  |  |  |  |  |  |  | | --- | --- | --- | --- | --- | --- | --- | --- | | **Overview** | **Package** | **Class** | **Use** | **Tree** | **Deprecated** | **Index** | **Help** | | |  |
| **PREV CLASS**   **NEXT CLASS** | **FRAMES**    **NO FRAMES**     **All Classes** |
| SUMMARY: NESTED | FIELD | CONSTR | METHOD | DETAIL: FIELD | CONSTR | METHOD |


---


## fern.cytoscape Class NetworkChecker.NodeClassifierByAnnotation<T>

```
java.lang.Object
  fern.cytoscape.NetworkChecker.NodeClassifierByAnnotation<T>
```

**All Implemented Interfaces:**: NetworkChecker.NodeClassifier

**Enclosing class:**: NetworkChecker

---

``` public class NetworkChecker.NodeClassifierByAnnotation<T> extends Object implements NetworkChecker.NodeClassifier ```

---

| **Constructor Summary** | |
| --- | --- |
| `NetworkChecker.NodeClassifierByAnnotation(String typeIdentifier, T reactionType, T speciesType)` |


| **Method Summary** | |
| --- | --- |
| `boolean` | `isReactionNode(giny.model.Node n)` |
| `boolean` | `isSpeciesNode(giny.model.Node n)` |
| `boolean` | `isUsable()` |

| **Methods inherited from class java.lang.Object** |
| --- |
| `clone, equals, finalize, getClass, hashCode, notify, notifyAll, toString, wait, wait, wait` |

| **Constructor Detail** |
| --- |

### NetworkChecker.NodeClassifierByAnnotation

```
public NetworkChecker.NodeClassifierByAnnotation(String typeIdentifier,
                                                 T reactionType,
                                                 T speciesType)
```


| **Method Detail** |
| --- |

### isReactionNode

```
public boolean isReactionNode(giny.model.Node n)
```

:   **Specified by:**: `isReactionNode` in interface `NetworkChecker.NodeClassifier`

---


### isSpeciesNode

```
public boolean isSpeciesNode(giny.model.Node n)
```

:   **Specified by:**: `isSpeciesNode` in interface `NetworkChecker.NodeClassifier`

---


### isUsable

```
public boolean isUsable()
```

:   **Specified by:**: `isUsable` in interface `NetworkChecker.NodeClassifier`


---


|  |  |  |  |  |  |  |  |  |  |  |
| --- | --- | --- | --- | --- | --- | --- | --- | --- | --- | --- |
| |  |  |  |  |  |  |  |  | | --- | --- | --- | --- | --- | --- | --- | --- | | **Overview** | **Package** | **Class** | **Use** | **Tree** | **Deprecated** | **Index** | **Help** | | |  |
| **PREV CLASS**   **NEXT CLASS** | **FRAMES**    **NO FRAMES**     **All Classes** |
| SUMMARY: NESTED | FIELD | CONSTR | METHOD | DETAIL: FIELD | CONSTR | METHOD |


---
