# Supplementary material for: FERN – a Java framework for stochastic simulation and evaluation of reaction networks
Source: BMC Bioinformatics. 2008 Aug 29;9:356. doi: 10.1186/1471-2105-9-356 (PMC2553347; doi:10.1186/1471-2105-9-356)
Supplement: Additional file 1 — FERN distribution, Version 1.3. This archive contains the FERN source code and binaries as well as documentation and example models in FernML and SBML. [file 1471-2105-9-356-S1.zip › fern/doc/javadoc/fern/cytoscape/NetworkChecker.NodeParameter.html]

NetworkChecker.NodeParameter


---


|  |  |  |  |  |  |  |  |  |  |  |
| --- | --- | --- | --- | --- | --- | --- | --- | --- | --- | --- |
| |  |  |  |  |  |  |  |  | | --- | --- | --- | --- | --- | --- | --- | --- | | **Overview** | **Package** | **Class** | **Use** | **Tree** | **Deprecated** | **Index** | **Help** | | |  |
| **PREV CLASS**   NEXT CLASS | **FRAMES**    **NO FRAMES**     **All Classes** |
| SUMMARY: NESTED | FIELD | CONSTR | METHOD | DETAIL: FIELD | CONSTR | METHOD |


---


## fern.cytoscape Class NetworkChecker.NodeParameter

```
java.lang.Object
  fern.cytoscape.NetworkChecker.NodeParameter
```

**Enclosing class:**: NetworkChecker

---

``` public class NetworkChecker.NodeParameter extends Object ```

---

| **Constructor Summary** | |
| --- | --- |
| `NetworkChecker.NodeParameter(String coeff, String initAm)` |


| **Method Summary** | |
| --- | --- |
| `double` | `getReactionCoefficient(giny.model.Node n)` |
| `long` | `getSpeciesInitialAmount(giny.model.Node n)` |
| `boolean` | `isUsable()` |
| `void` | `setSpeciesInitialAmount(giny.model.Node n, long value)` |

| **Methods inherited from class java.lang.Object** |
| --- |
| `clone, equals, finalize, getClass, hashCode, notify, notifyAll, toString, wait, wait, wait` |

| **Constructor Detail** |
| --- |

### NetworkChecker.NodeParameter

```
public NetworkChecker.NodeParameter(String coeff,
                                    String initAm)
```


| **Method Detail** |
| --- |

### getReactionCoefficient

```
public double getReactionCoefficient(giny.model.Node n)
```

---


### getSpeciesInitialAmount

```
public long getSpeciesInitialAmount(giny.model.Node n)
```

---


### setSpeciesInitialAmount

```
public void setSpeciesInitialAmount(giny.model.Node n,
                                    long value)
```

---


### isUsable

```
public boolean isUsable()
```


---


|  |  |  |  |  |  |  |  |  |  |  |
| --- | --- | --- | --- | --- | --- | --- | --- | --- | --- | --- |
| |  |  |  |  |  |  |  |  | | --- | --- | --- | --- | --- | --- | --- | --- | | **Overview** | **Package** | **Class** | **Use** | **Tree** | **Deprecated** | **Index** | **Help** | | |  |
| **PREV CLASS**   NEXT CLASS | **FRAMES**    **NO FRAMES**     **All Classes** |
| SUMMARY: NESTED | FIELD | CONSTR | METHOD | DETAIL: FIELD | CONSTR | METHOD |


---
