# Supplementary material for: FERN – a Java framework for stochastic simulation and evaluation of reaction networks
Source: BMC Bioinformatics. 2008 Aug 29;9:356. doi: 10.1186/1471-2105-9-356 (PMC2553347; doi:10.1186/1471-2105-9-356)
Supplement: Additional file 1 — FERN distribution, Version 1.3. This archive contains the FERN source code and binaries as well as documentation and example models in FernML and SBML. [file 1471-2105-9-356-S1.zip › fern/doc/javadoc/fern/cytoscape/package-summary.html]

fern.cytoscape


---


|  |  |  |  |  |  |  |  |  |  |  |
| --- | --- | --- | --- | --- | --- | --- | --- | --- | --- | --- |
| |  |  |  |  |  |  |  |  | | --- | --- | --- | --- | --- | --- | --- | --- | | **Overview** | **Package** | Class | **Use** | **Tree** | **Deprecated** | **Index** | **Help** | | |  |
| **PREV PACKAGE**   **NEXT PACKAGE** | **FRAMES**    **NO FRAMES**     **All Classes** |


---

## Package fern.cytoscape

Provides the classes for the cytoscape plugin.

**See:**
  
          **Description**

| **Interface Summary** | |
| --- | --- |
| **NetworkChecker.EdgeClassifier** |  |
| **NetworkChecker.NodeClassifier** |  |

| **Class Summary** | |
| --- | --- |
| **ColorCalculator** |  |
| **CytoscapeAnnotationManager** |  |
| **CytoscapeColorChangeObserver** |  |
| **CytoscapeColorChangeObserver.ColorChangingNodeAppeareanceCalculator** |  |
| **CytoscapeNetworkWrapper** |  |
| **CytoscapeVisualizer** |  |
| **FernVisualStyle** |  |
| **NetworkChecker** |  |

| **Enum Summary** | |
| --- | --- |
| **ColorCalculator.Scale** |  |

## Package fern.cytoscape Description

Provides the classes for the cytoscape plugin.

---


|  |  |  |  |  |  |  |  |  |  |  |
| --- | --- | --- | --- | --- | --- | --- | --- | --- | --- | --- |
| |  |  |  |  |  |  |  |  | | --- | --- | --- | --- | --- | --- | --- | --- | | **Overview** | **Package** | Class | **Use** | **Tree** | **Deprecated** | **Index** | **Help** | | |  |
| **PREV PACKAGE**   **NEXT PACKAGE** | **FRAMES**    **NO FRAMES**     **All Classes** |


---
