# Supplementary material for: FERN – a Java framework for stochastic simulation and evaluation of reaction networks
Source: BMC Bioinformatics. 2008 Aug 29;9:356. doi: 10.1186/1471-2105-9-356 (PMC2553347; doi:10.1186/1471-2105-9-356)
Supplement: Additional file 1 — FERN distribution, Version 1.3. This archive contains the FERN source code and binaries as well as documentation and example models in FernML and SBML. [file 1471-2105-9-356-S1.zip › fern/doc/javadoc/fern/cytoscape/package-tree.html]

fern.cytoscape Class Hierarchy


---


|  |  |  |  |  |  |  |  |  |  |  |
| --- | --- | --- | --- | --- | --- | --- | --- | --- | --- | --- |
| |  |  |  |  |  |  |  |  | | --- | --- | --- | --- | --- | --- | --- | --- | | **Overview** | **Package** | Class | Use | **Tree** | **Deprecated** | **Index** | **Help** | | |  |
| **PREV**   **NEXT** | **FRAMES**    **NO FRAMES**     **All Classes** |


---


## Hierarchy For Package fern.cytoscape

**Package Hierarchies:**: All Packages

---

## Class Hierarchy

- java.lang.**Object**
  - javax.swing.**AbstractAction** (implements javax.swing.Action, java.lang.Cloneable, java.io.Serializable)
    - cytoscape.util.CytoscapeAction
      - fern.cytoscape.**CytoscapeVisualizer.ShowMainFrameAction**- fern.cytoscape.**CytoscapeVisualizer.SimulationAction**- fern.network.**AbstractNetworkImpl** (implements fern.network.Network)
      - fern.cytoscape.**CytoscapeNetworkWrapper**- fern.cytoscape.**ColorCalculator** (implements java.lang.Cloneable)- fern.cytoscape.**CytoscapeAnnotationManager** (implements fern.network.AnnotationManager)- cytoscape.plugin.CytoscapePlugin (implements java.beans.PropertyChangeListener)
            - fern.cytoscape.**CytoscapeVisualizer**- fern.cytoscape.**NetworkChecker**- fern.cytoscape.**NetworkChecker.EdgeClassifierByDirection** (implements fern.cytoscape.NetworkChecker.EdgeClassifier)- fern.cytoscape.**NetworkChecker.EdgeClassifierByIdentifier** (implements fern.cytoscape.NetworkChecker.EdgeClassifier)- fern.cytoscape.**NetworkChecker.NodeClassifierByAnnotation**<T> (implements fern.cytoscape.NetworkChecker.NodeClassifier)- fern.cytoscape.**NetworkChecker.NodeParameter**- cytoscape.visual.NodeAppearanceCalculator
                        - fern.cytoscape.**CytoscapeColorChangeObserver.ColorChangingNodeAppeareanceCalculator**- fern.simulation.observer.**Observer**
                          - fern.cytoscape.**CytoscapeColorChangeObserver**- cytoscape.visual.VisualStyle (implements java.lang.Cloneable)
                            - fern.cytoscape.**FernVisualStyle**

## Interface Hierarchy

- fern.cytoscape.**NetworkChecker.EdgeClassifier**- fern.cytoscape.**NetworkChecker.NodeClassifier**

## Enum Hierarchy

- java.lang.**Object**
  - java.lang.**Enum**<E> (implements java.lang.Comparable<T>, java.io.Serializable)
    - fern.cytoscape.**ColorCalculator.Scale**

---


|  |  |  |  |  |  |  |  |  |  |  |
| --- | --- | --- | --- | --- | --- | --- | --- | --- | --- | --- |
| |  |  |  |  |  |  |  |  | | --- | --- | --- | --- | --- | --- | --- | --- | | **Overview** | **Package** | Class | Use | **Tree** | **Deprecated** | **Index** | **Help** | | |  |
| **PREV**   **NEXT** | **FRAMES**    **NO FRAMES**     **All Classes** |


---
