# Supplementary material for: FERN – a Java framework for stochastic simulation and evaluation of reaction networks
Source: BMC Bioinformatics. 2008 Aug 29;9:356. doi: 10.1186/1471-2105-9-356 (PMC2553347; doi:10.1186/1471-2105-9-356)
Supplement: Additional file 1 — FERN distribution, Version 1.3. This archive contains the FERN source code and binaries as well as documentation and example models in FernML and SBML. [file 1471-2105-9-356-S1.zip › fern/doc/javadoc/fern/cytoscape/package-use.html]

Uses of Package fern.cytoscape


---


|  |  |  |  |  |  |  |  |  |  |  |
| --- | --- | --- | --- | --- | --- | --- | --- | --- | --- | --- |
| |  |  |  |  |  |  |  |  | | --- | --- | --- | --- | --- | --- | --- | --- | | **Overview** | **Package** | Class | **Use** | **Tree** | **Deprecated** | **Index** | **Help** | | |  |
| PREV   NEXT | **FRAMES**    **NO FRAMES**     **All Classes** |


---


## **Uses of Package fern.cytoscape**

| Packages that use fern.cytoscape | |
| --- | --- |
| **fern.cytoscape** | Provides the classes for the cytoscape plugin. |
| **fern.cytoscape.ui** |  |

| Classes in fern.cytoscape used by fern.cytoscape | |
| --- | --- |
| ****ColorCalculator**** |
| ****ColorCalculator.Scale**** |
| ****CytoscapeNetworkWrapper**** |
| ****FernVisualStyle**** |
| ****NetworkChecker**** |
| ****NetworkChecker.EdgeClassifier**** |
| ****NetworkChecker.NodeClassifier**** |
| ****NetworkChecker.NodeParameter**** |

| Classes in fern.cytoscape used by fern.cytoscape.ui | |
| --- | --- |
| ****CytoscapeNetworkWrapper**** |
| ****FernVisualStyle**** |
| ****NetworkChecker**** |

---


|  |  |  |  |  |  |  |  |  |  |  |
| --- | --- | --- | --- | --- | --- | --- | --- | --- | --- | --- |
| |  |  |  |  |  |  |  |  | | --- | --- | --- | --- | --- | --- | --- | --- | | **Overview** | **Package** | Class | **Use** | **Tree** | **Deprecated** | **Index** | **Help** | | |  |
| PREV   NEXT | **FRAMES**    **NO FRAMES**     **All Classes** |


---
