# Supplementary material for: FERN – a Java framework for stochastic simulation and evaluation of reaction networks
Source: BMC Bioinformatics. 2008 Aug 29;9:356. doi: 10.1186/1471-2105-9-356 (PMC2553347; doi:10.1186/1471-2105-9-356)
Supplement: Additional file 1 — FERN distribution, Version 1.3. This archive contains the FERN source code and binaries as well as documentation and example models in FernML and SBML. [file 1471-2105-9-356-S1.zip › fern/doc/javadoc/fern/cytoscape/ui/package-tree.html]

fern.cytoscape.ui Class Hierarchy


---


|  |  |  |  |  |  |  |  |  |  |  |
| --- | --- | --- | --- | --- | --- | --- | --- | --- | --- | --- |
| |  |  |  |  |  |  |  |  | | --- | --- | --- | --- | --- | --- | --- | --- | | **Overview** | **Package** | Class | Use | **Tree** | **Deprecated** | **Index** | **Help** | | |  |
| **PREV**   **NEXT** | **FRAMES**    **NO FRAMES**     **All Classes** |


---


## Hierarchy For Package fern.cytoscape.ui

**Package Hierarchies:**: All Packages

---

## Class Hierarchy

- java.lang.**Object**
  - java.awt.**Component** (implements java.awt.image.ImageObserver, java.awt.MenuContainer, java.io.Serializable)
    - java.awt.**Container**
      - javax.swing.**JComponent** (implements java.io.Serializable)
        - javax.swing.**JPanel** (implements javax.accessibility.Accessible)
          - fern.cytoscape.ui.**ExtendedPane**- fern.cytoscape.ui.**OverviewPane**- java.awt.**Window** (implements javax.accessibility.Accessible)
          - java.awt.**Dialog**
            - javax.swing.**JDialog** (implements javax.accessibility.Accessible, javax.swing.RootPaneContainer, javax.swing.WindowConstants)
              - fern.cytoscape.ui.**ColorPicker**- java.awt.**Frame** (implements java.awt.MenuContainer)
              - javax.swing.**JFrame** (implements javax.accessibility.Accessible, javax.swing.RootPaneContainer, javax.swing.WindowConstants)
                - fern.cytoscape.ui.**MainFrame**

---


|  |  |  |  |  |  |  |  |  |  |  |
| --- | --- | --- | --- | --- | --- | --- | --- | --- | --- | --- |
| |  |  |  |  |  |  |  |  | | --- | --- | --- | --- | --- | --- | --- | --- | | **Overview** | **Package** | Class | Use | **Tree** | **Deprecated** | **Index** | **Help** | | |  |
| **PREV**   **NEXT** | **FRAMES**    **NO FRAMES**     **All Classes** |


---
