# Supplementary material for: FERN – a Java framework for stochastic simulation and evaluation of reaction networks
Source: BMC Bioinformatics. 2008 Aug 29;9:356. doi: 10.1186/1471-2105-9-356 (PMC2553347; doi:10.1186/1471-2105-9-356)
Supplement: Additional file 1 — FERN distribution, Version 1.3. This archive contains the FERN source code and binaries as well as documentation and example models in FernML and SBML. [file 1471-2105-9-356-S1.zip › fern/doc/javadoc/fern/example/AutocatalyticNetworkExample.html]

AutocatalyticNetworkExample


---


|  |  |  |  |  |  |  |  |  |  |  |
| --- | --- | --- | --- | --- | --- | --- | --- | --- | --- | --- |
| |  |  |  |  |  |  |  |  | | --- | --- | --- | --- | --- | --- | --- | --- | | **Overview** | **Package** | **Class** | **Use** | **Tree** | **Deprecated** | **Index** | **Help** | | |  |
| PREV CLASS   **NEXT CLASS** | **FRAMES**    **NO FRAMES**     **All Classes** |
| SUMMARY: NESTED | FIELD | CONSTR | METHOD | DETAIL: FIELD | CONSTR | METHOD |


---


## fern.example Class AutocatalyticNetworkExample

```
java.lang.Object
  fern.example.AutocatalyticNetworkExample
```

---

``` public class AutocatalyticNetworkExample extends Object ```

Here, the evolution of a reaction network as proposed by [1]
is performed. Then, the autocatalytic subset [1] is determined and extracted.
This subnet is then simulated to examine the dynamic behaviour of autocatalytic
reaction networks. For more information about the evolution and the detection algorithm,
please refer `AutocatalyticNetwork` and `AutocatalyticNetworkDetection`.

References:
[1] Kauffmann S.A, The Origins of Order: Self-Organization and Selection in Evolution. New York: Oxford University Press, (1993)

**Author:**
:   Florian Erhard

---

| **Constructor Summary** | |
| --- | --- |
| `AutocatalyticNetworkExample()` |


| **Method Summary** | |
| --- | --- |
| `static void` | `main(String[] args)` |

| **Methods inherited from class java.lang.Object** |
| --- |
| `clone, equals, finalize, getClass, hashCode, notify, notifyAll, toString, wait, wait, wait` |

| **Constructor Detail** |
| --- |

### AutocatalyticNetworkExample

```
public AutocatalyticNetworkExample()
```


| **Method Detail** |
| --- |

### main

```
public static void main(String[] args)
                 throws IOException
```

:   **Throws:**: `IOException`


---


|  |  |  |  |  |  |  |  |  |  |  |
| --- | --- | --- | --- | --- | --- | --- | --- | --- | --- | --- |
| |  |  |  |  |  |  |  |  | | --- | --- | --- | --- | --- | --- | --- | --- | | **Overview** | **Package** | **Class** | **Use** | **Tree** | **Deprecated** | **Index** | **Help** | | |  |
| PREV CLASS   **NEXT CLASS** | **FRAMES**    **NO FRAMES**     **All Classes** |
| SUMMARY: NESTED | FIELD | CONSTR | METHOD | DETAIL: FIELD | CONSTR | METHOD |


---
