# Supplementary material for: FERN – a Java framework for stochastic simulation and evaluation of reaction networks
Source: BMC Bioinformatics. 2008 Aug 29;9:356. doi: 10.1186/1471-2105-9-356 (PMC2553347; doi:10.1186/1471-2105-9-356)
Supplement: Additional file 1 — FERN distribution, Version 1.3. This archive contains the FERN source code and binaries as well as documentation and example models in FernML and SBML. [file 1471-2105-9-356-S1.zip › fern/doc/javadoc/fern/example/CellGrowthObserver.html]

CellGrowthObserver


---


|  |  |  |  |  |  |  |  |  |  |  |
| --- | --- | --- | --- | --- | --- | --- | --- | --- | --- | --- |
| |  |  |  |  |  |  |  |  | | --- | --- | --- | --- | --- | --- | --- | --- | | **Overview** | **Package** | **Class** | **Use** | **Tree** | **Deprecated** | **Index** | **Help** | | |  |
| **PREV CLASS**   **NEXT CLASS** | **FRAMES**    **NO FRAMES**     **All Classes** |
| SUMMARY: NESTED | FIELD | CONSTR | METHOD | DETAIL: FIELD | CONSTR | METHOD |


---


## fern.example Class CellGrowthObserver

```
java.lang.Object
  fern.simulation.observer.Observer
      fern.example.CellGrowthObserver
```

---

``` public class CellGrowthObserver extends Observer ```

Does not observe anything, but it controls the reaction networks cell. At the beginning
the volume is set to 1 and then linearly increased to 2 until the generation time is reached.
Then a cell division is simulated by dividing each population by 2 and restart the volume
at 1.

This class is used for the LacZ examples, so at a cell division the amount of PLac (the
promoter) is set to 1.

**Author:**
:   Florian Erhard

---

| **Constructor Summary** | |
| --- | --- |
| `CellGrowthObserver(Simulator sim, double generationTime, double timeOffset)`             Creates the observer for given simulator, generation time and a time offset (because the simulator starts at time 0 and for one example it actually starts at time 1000) |


| **Method Summary** | |
| --- | --- |
| `void` | `activateReaction(int mu, double tau, Simulator.FireType fireType, int times)`             Gets called before a reaction fires. |
| `void` | `finished()`             Gets called when a simulation has finished, directly after the termination check. |
| `long` | `getNumSteps()`             Gets the number of steps taken in one simulation |
| `void` | `started()`             Gets called when the simulation has started after the initialization and before the termination condition is checked the first time. |
| `void` | `step()`             Gets called after each termination check and before `Simulator.performStep(fern.simulation.controller.SimulationController)` is called. |
| `void` | `theta(double theta)`             Gets called by simulators when a certain moment in time is reached. |

| **Methods inherited from class fern.simulation.observer.Observer** |
| --- |
| `applyLabelFormat, getLabel, getLabelFormat, getNumSimulations, getPrintWriter, getSimulator, getTheta, getTitlesCommand, print, setLabelFormat, setPrintWriter, setSimulator, setTheta` |

| **Methods inherited from class java.lang.Object** |
| --- |
| `clone, equals, finalize, getClass, hashCode, notify, notifyAll, toString, wait, wait, wait` |

| **Constructor Detail** |
| --- |

### CellGrowthObserver

```
public CellGrowthObserver(Simulator sim,
                          double generationTime,
                          double timeOffset)
```

:   Creates the observer for given simulator, generation time and a time offset (because
    the simulator starts at time 0 and for one example it actually starts at time 1000)

    **Parameters:**: `sim` - the simulator: `generationTime` - generation time: `timeOffset` - time offset


| **Method Detail** |
| --- |

### activateReaction

```
public void activateReaction(int mu,
                             double tau,
                             Simulator.FireType fireType,
                             int times)
```

:   **Description copied from class: `Observer`**
:   Gets called before a reaction fires.

    :   **Specified by:**: `activateReaction` in class `Observer`
    :   **Parameters:**: `mu` - the reaction which is supposed to fire: `tau` - the time the reaction fires (at this time `Simulator.getTime()` does not necessarily yield the firing time): `fireType` - the type of the firing: `times` - TODO

---


### finished

```
public void finished()
```

:   **Description copied from class: `Observer`**
:   Gets called when a simulation has finished, directly after the termination check.

    :   **Specified by:**: `finished` in class `Observer`

---


### started

```
public void started()
```

:   **Description copied from class: `Observer`**
:   Gets called when the simulation has started after the initialization and before
    the termination condition is checked the first time.

    :   **Specified by:**: `started` in class `Observer`

---


### step

```
public void step()
```

:   **Description copied from class: `Observer`**
:   Gets called after each termination check and before `Simulator.performStep(fern.simulation.controller.SimulationController)`
    is called.

    :   **Specified by:**: `step` in class `Observer`

---


### theta

```
public void theta(double theta)
```

:   **Description copied from class: `Observer`**
:   Gets called by simulators when a certain moment in time is reached. This moment in
    time has to be registered by `Observer.getTheta()`

    :   **Specified by:**: `theta` in class `Observer`
    :   **Parameters:**: `theta` - moment in time

---


### getNumSteps

```
public long getNumSteps()
```

:   Gets the number of steps taken in one simulation

    :   **Returns:**: number of steps


---


|  |  |  |  |  |  |  |  |  |  |  |
| --- | --- | --- | --- | --- | --- | --- | --- | --- | --- | --- |
| |  |  |  |  |  |  |  |  | | --- | --- | --- | --- | --- | --- | --- | --- | | **Overview** | **Package** | **Class** | **Use** | **Tree** | **Deprecated** | **Index** | **Help** | | |  |
| **PREV CLASS**   **NEXT CLASS** | **FRAMES**    **NO FRAMES**     **All Classes** |
| SUMMARY: NESTED | FIELD | CONSTR | METHOD | DETAIL: FIELD | CONSTR | METHOD |


---
