# Supplementary material for: FERN – a Java framework for stochastic simulation and evaluation of reaction networks
Source: BMC Bioinformatics. 2008 Aug 29;9:356. doi: 10.1186/1471-2105-9-356 (PMC2553347; doi:10.1186/1471-2105-9-356)
Supplement: Additional file 1 — FERN distribution, Version 1.3. This archive contains the FERN source code and binaries as well as documentation and example models in FernML and SBML. [file 1471-2105-9-356-S1.zip › fern/doc/javadoc/fern/example/DecayingDimerizingPlots.html]

DecayingDimerizingPlots


---


|  |  |  |  |  |  |  |  |  |  |  |
| --- | --- | --- | --- | --- | --- | --- | --- | --- | --- | --- |
| |  |  |  |  |  |  |  |  | | --- | --- | --- | --- | --- | --- | --- | --- | | **Overview** | **Package** | **Class** | **Use** | **Tree** | **Deprecated** | **Index** | **Help** | | |  |
| **PREV CLASS**   **NEXT CLASS** | **FRAMES**    **NO FRAMES**     **All Classes** |
| SUMMARY: NESTED | FIELD | CONSTR | METHOD | DETAIL: FIELD | CONSTR | METHOD |


---


## fern.example Class DecayingDimerizingPlots

```
java.lang.Object
  fern.example.DecayingDimerizingPlots
```

---

**Deprecated.**

``` @Deprecated public class DecayingDimerizingPlots extends Object ```

Demonstration of performance and accuracy differences of the different
simulation algorithms. The reaction network proposed in [1]
are used to calculate histograms and the histogram distances [4] of the amount
of some molecular species at a special time point in order to compare results
of different algorithms. Here, Fig. 3 of [3] is reproduced and the gain
of accuracy when using a species bounded tau leaping procedure without
loss of performance is shown.

For references see
[1] Gillespie D.T., J. Comput. Phys. 22, 403 (1976),
[2] D.Gillespie, J.Chem.Phys. 115, 1716 (2001),
[3] D.Gillespie and L.Petzold, J.Chem.Phys. 119, 8229 (2003),
[4] Cao Y. and Petzold L., J. Comp. Phys. 212, 6�24 (2006).

---

| **Constructor Summary** | |
| --- | --- |
| `DecayingDimerizingPlots()`             **Deprecated.** |


| **Method Summary** | |
| --- | --- |
| `static void` | `main(String[] args)`             **Deprecated.** |

| **Methods inherited from class java.lang.Object** |
| --- |
| `clone, equals, finalize, getClass, hashCode, notify, notifyAll, toString, wait, wait, wait` |

| **Constructor Detail** |
| --- |

### DecayingDimerizingPlots

```
public DecayingDimerizingPlots()
```

:   **Deprecated.**


| **Method Detail** |
| --- |

### main

```
public static void main(String[] args)
                 throws IOException,
                        JDOMException
```

:   **Deprecated.**:   **Throws:**: `IOException`: `JDOMException`


---


|  |  |  |  |  |  |  |  |  |  |  |
| --- | --- | --- | --- | --- | --- | --- | --- | --- | --- | --- |
| |  |  |  |  |  |  |  |  | | --- | --- | --- | --- | --- | --- | --- | --- | | **Overview** | **Package** | **Class** | **Use** | **Tree** | **Deprecated** | **Index** | **Help** | | |  |
| **PREV CLASS**   **NEXT CLASS** | **FRAMES**    **NO FRAMES**     **All Classes** |
| SUMMARY: NESTED | FIELD | CONSTR | METHOD | DETAIL: FIELD | CONSTR | METHOD |


---
