# Supplementary material for: FERN – a Java framework for stochastic simulation and evaluation of reaction networks
Source: BMC Bioinformatics. 2008 Aug 29;9:356. doi: 10.1186/1471-2105-9-356 (PMC2553347; doi:10.1186/1471-2105-9-356)
Supplement: Additional file 1 — FERN distribution, Version 1.3. This archive contains the FERN source code and binaries as well as documentation and example models in FernML and SBML. [file 1471-2105-9-356-S1.zip › fern/doc/javadoc/fern/example/Dsmts.html]

Dsmts


---


|  |  |  |  |  |  |  |  |  |  |  |
| --- | --- | --- | --- | --- | --- | --- | --- | --- | --- | --- |
| |  |  |  |  |  |  |  |  | | --- | --- | --- | --- | --- | --- | --- | --- | | **Overview** | **Package** | **Class** | **Use** | **Tree** | **Deprecated** | **Index** | **Help** | | |  |
| **PREV CLASS**   **NEXT CLASS** | **FRAMES**    **NO FRAMES**     **All Classes** |
| SUMMARY: NESTED | FIELD | CONSTR | METHOD | DETAIL: FIELD | CONSTR | METHOD |


---


## fern.example Class Dsmts

```
java.lang.Object
  fern.example.Dsmts
```

---

``` public class Dsmts extends Object ```

Perform a series of tests (refer to http://www.calibayes.ncl.ac.uk/Resources/dsmts).
You have to specify the path to the unpacked dsmts archive. The method test produces
one line of text containing the test results for each species in the model. If specified,
it also produces 4 plots:

- average trend curve of the simulated trajectories and the analytical determined
- stddev trend curve of the simulated trajectories and the analytical determined
- deviation of the simulated averages to the real ones (the z values described in the dsmts user guide)
- deviation of the simulated stddevs to the real ones (the y values described in the dsmts user guide)

It may be wise to leave the producePlot flag set to false in the for loop because for
each test model 4 windows will pop up!

---

| **Constructor Summary** | |
| --- | --- |
| `Dsmts()` |


| **Method Summary** | |
| --- | --- |
| `static void` | `main(String[] args)` |

| **Methods inherited from class java.lang.Object** |
| --- |
| `clone, equals, finalize, getClass, hashCode, notify, notifyAll, toString, wait, wait, wait` |

| **Constructor Detail** |
| --- |

### Dsmts

```
public Dsmts()
```


| **Method Detail** |
| --- |

### main

```
public static void main(String[] args)
                 throws IOException
```

:   **Throws:**: `IOException`


---


|  |  |  |  |  |  |  |  |  |  |  |
| --- | --- | --- | --- | --- | --- | --- | --- | --- | --- | --- |
| |  |  |  |  |  |  |  |  | | --- | --- | --- | --- | --- | --- | --- | --- | | **Overview** | **Package** | **Class** | **Use** | **Tree** | **Deprecated** | **Index** | **Help** | | |  |
| **PREV CLASS**   **NEXT CLASS** | **FRAMES**    **NO FRAMES**     **All Classes** |
| SUMMARY: NESTED | FIELD | CONSTR | METHOD | DETAIL: FIELD | CONSTR | METHOD |


---
