# Supplementary material for: FERN – a Java framework for stochastic simulation and evaluation of reaction networks
Source: BMC Bioinformatics. 2008 Aug 29;9:356. doi: 10.1186/1471-2105-9-356 (PMC2553347; doi:10.1186/1471-2105-9-356)
Supplement: Additional file 1 — FERN distribution, Version 1.3. This archive contains the FERN source code and binaries as well as documentation and example models in FernML and SBML. [file 1471-2105-9-356-S1.zip › fern/doc/javadoc/fern/example/HistogramDistanceTestSet.html]

HistogramDistanceTestSet


---


|  |  |  |  |  |  |  |  |  |  |  |
| --- | --- | --- | --- | --- | --- | --- | --- | --- | --- | --- |
| |  |  |  |  |  |  |  |  | | --- | --- | --- | --- | --- | --- | --- | --- | | **Overview** | **Package** | **Class** | **Use** | **Tree** | **Deprecated** | **Index** | **Help** | | |  |
| **PREV CLASS**   **NEXT CLASS** | **FRAMES**    **NO FRAMES**     **All Classes** |
| SUMMARY: NESTED | FIELD | CONSTR | METHOD | DETAIL: FIELD | CONSTR | METHOD |


---


## fern.example Class HistogramDistanceTestSet

```
java.lang.Object
  fern.example.HistogramDistanceTestSet
```

---

``` public class HistogramDistanceTestSet extends Object ```

Encapsulate test sets for histogram distance calculations.

**Author:**
:   Florian Erhard

---

| **Constructor Summary** | |
| --- | --- |
| `HistogramDistanceTestSet(Simulator sim, double eps, int runs, double time, String species)` |


| **Method Summary** | |
| --- | --- |
| `void` | `createHistogram()` |
| `double` | `getEpsilon()` |
| `File` | `getFile()` |
| `double[][]` | `getHistoAsParallelArray()` |
| `Map<Integer,Integer>` | `getHistogram()` |
| `Simulator` | `getSimulator()` |

| **Methods inherited from class java.lang.Object** |
| --- |
| `clone, equals, finalize, getClass, hashCode, notify, notifyAll, toString, wait, wait, wait` |

| **Constructor Detail** |
| --- |

### HistogramDistanceTestSet

```
public HistogramDistanceTestSet(Simulator sim,
                                double eps,
                                int runs,
                                double time,
                                String species)
```


| **Method Detail** |
| --- |

### createHistogram

```
public void createHistogram()
                     throws IOException
```

:   **Throws:**: `IOException`

---


### getHistoAsParallelArray

```
public double[][] getHistoAsParallelArray()
```

---


### getFile

```
public File getFile()
```

---


### getHistogram

```
public Map<Integer,Integer> getHistogram()
```

---


### getEpsilon

```
public double getEpsilon()
```

---


### getSimulator

```
public Simulator getSimulator()
```


---


|  |  |  |  |  |  |  |  |  |  |  |
| --- | --- | --- | --- | --- | --- | --- | --- | --- | --- | --- |
| |  |  |  |  |  |  |  |  | | --- | --- | --- | --- | --- | --- | --- | --- | | **Overview** | **Package** | **Class** | **Use** | **Tree** | **Deprecated** | **Index** | **Help** | | |  |
| **PREV CLASS**   **NEXT CLASS** | **FRAMES**    **NO FRAMES**     **All Classes** |
| SUMMARY: NESTED | FIELD | CONSTR | METHOD | DETAIL: FIELD | CONSTR | METHOD |


---
