# Supplementary material for: FERN – a Java framework for stochastic simulation and evaluation of reaction networks
Source: BMC Bioinformatics. 2008 Aug 29;9:356. doi: 10.1186/1471-2105-9-356 (PMC2553347; doi:10.1186/1471-2105-9-356)
Supplement: Additional file 1 — FERN distribution, Version 1.3. This archive contains the FERN source code and binaries as well as documentation and example models in FernML and SBML. [file 1471-2105-9-356-S1.zip › fern/doc/javadoc/fern/example/LacYComplete.html]

LacYComplete


---


|  |  |  |  |  |  |  |  |  |  |  |
| --- | --- | --- | --- | --- | --- | --- | --- | --- | --- | --- |
| |  |  |  |  |  |  |  |  | | --- | --- | --- | --- | --- | --- | --- | --- | | **Overview** | **Package** | **Class** | **Use** | **Tree** | **Deprecated** | **Index** | **Help** | | |  |
| **PREV CLASS**   **NEXT CLASS** | **FRAMES**    **NO FRAMES**     **All Classes** |
| SUMMARY: NESTED | FIELD | CONSTR | METHOD | DETAIL: FIELD | CONSTR | METHOD |


---


## fern.example Class LacYComplete

```
java.lang.Object
  fern.example.LacYComplete
```

---

``` public class LacYComplete extends Object ```

The LacZ/LacY model of procaryotic gene expression proposed by [1]
is simulated. Here, the full model (containing LacZ and LacY regulation)
is simulated for one full cell cycle. Since it is very time consuming with an exact method,
the simulation progress is shown online in the console.

For references see [1] Kierzek A.M., Bioinformatics 18, 670 (2002)

**Author:**
:   Florian Erhard

---

| **Constructor Summary** | |
| --- | --- |
| `LacYComplete()` |


| **Method Summary** | |
| --- | --- |
| `static void` | `main(String[] args)` |

| **Methods inherited from class java.lang.Object** |
| --- |
| `clone, equals, finalize, getClass, hashCode, notify, notifyAll, toString, wait, wait, wait` |

| **Constructor Detail** |
| --- |

### LacYComplete

```
public LacYComplete()
```


| **Method Detail** |
| --- |

### main

```
public static void main(String[] args)
                 throws IOException,
                        JDOMException
```

:   **Throws:**: `IOException`: `JDOMException`


---


|  |  |  |  |  |  |  |  |  |  |  |
| --- | --- | --- | --- | --- | --- | --- | --- | --- | --- | --- |
| |  |  |  |  |  |  |  |  | | --- | --- | --- | --- | --- | --- | --- | --- | | **Overview** | **Package** | **Class** | **Use** | **Tree** | **Deprecated** | **Index** | **Help** | | |  |
| **PREV CLASS**   **NEXT CLASS** | **FRAMES**    **NO FRAMES**     **All Classes** |
| SUMMARY: NESTED | FIELD | CONSTR | METHOD | DETAIL: FIELD | CONSTR | METHOD |


---
