# Supplementary material for: FERN – a Java framework for stochastic simulation and evaluation of reaction networks
Source: BMC Bioinformatics. 2008 Aug 29;9:356. doi: 10.1186/1471-2105-9-356 (PMC2553347; doi:10.1186/1471-2105-9-356)
Supplement: Additional file 1 — FERN distribution, Version 1.3. This archive contains the FERN source code and binaries as well as documentation and example models in FernML and SBML. [file 1471-2105-9-356-S1.zip › fern/doc/javadoc/fern/example/MapkBenchmark.html]

MapkBenchmark


---


|  |  |  |  |  |  |  |  |  |  |  |
| --- | --- | --- | --- | --- | --- | --- | --- | --- | --- | --- |
| |  |  |  |  |  |  |  |  | | --- | --- | --- | --- | --- | --- | --- | --- | | **Overview** | **Package** | **Class** | **Use** | **Tree** | **Deprecated** | **Index** | **Help** | | |  |
| **PREV CLASS**   **NEXT CLASS** | **FRAMES**    **NO FRAMES**     **All Classes** |
| SUMMARY: NESTED | FIELD | CONSTR | METHOD | DETAIL: FIELD | CONSTR | METHOD |


---


## fern.example Class MapkBenchmark

```
java.lang.Object
  fern.example.MapkBenchmark
```

---

``` public class MapkBenchmark extends Object ```

Use the signal transduction pathway network of the epidermal growth factor
proposed by [1] to introduce the benchmark system. For more information
please refer to `Benchmark`.

References:
[1] Lee D.-Y., Metabolic Engineering 8, 112-122 (2006)

**Author:**
:   Florian Erhard

---

| **Constructor Summary** | |
| --- | --- |
| `MapkBenchmark()` |


| **Method Summary** | |
| --- | --- |
| `static void` | `main(String[] args)` |

| **Methods inherited from class java.lang.Object** |
| --- |
| `clone, equals, finalize, getClass, hashCode, notify, notifyAll, toString, wait, wait, wait` |

| **Constructor Detail** |
| --- |

### MapkBenchmark

```
public MapkBenchmark()
```


| **Method Detail** |
| --- |

### main

```
public static void main(String[] args)
                 throws IOException,
                        JDOMException
```

:   **Throws:**: `IOException`: `JDOMException`


---


|  |  |  |  |  |  |  |  |  |  |  |
| --- | --- | --- | --- | --- | --- | --- | --- | --- | --- | --- |
| |  |  |  |  |  |  |  |  | | --- | --- | --- | --- | --- | --- | --- | --- | | **Overview** | **Package** | **Class** | **Use** | **Tree** | **Deprecated** | **Index** | **Help** | | |  |
| **PREV CLASS**   **NEXT CLASS** | **FRAMES**    **NO FRAMES**     **All Classes** |
| SUMMARY: NESTED | FIELD | CONSTR | METHOD | DETAIL: FIELD | CONSTR | METHOD |


---
