# Supplementary material for: FERN – a Java framework for stochastic simulation and evaluation of reaction networks
Source: BMC Bioinformatics. 2008 Aug 29;9:356. doi: 10.1186/1471-2105-9-356 (PMC2553347; doi:10.1186/1471-2105-9-356)
Supplement: Additional file 1 — FERN distribution, Version 1.3. This archive contains the FERN source code and binaries as well as documentation and example models in FernML and SBML. [file 1471-2105-9-356-S1.zip › fern/doc/javadoc/fern/example/package-summary.html]

fern.example


---


|  |  |  |  |  |  |  |  |  |  |  |
| --- | --- | --- | --- | --- | --- | --- | --- | --- | --- | --- |
| |  |  |  |  |  |  |  |  | | --- | --- | --- | --- | --- | --- | --- | --- | | **Overview** | **Package** | Class | **Use** | **Tree** | **Deprecated** | **Index** | **Help** | | |  |
| **PREV PACKAGE**   **NEXT PACKAGE** | **FRAMES**    **NO FRAMES**     **All Classes** |


---

## Package fern.example

Contains examples and demonstrations of the framework.

**See:**
  
          **Description**

| **Class Summary** | |
| --- | --- |
| **AutocatalyticNetworkExample** | Here, the evolution of a reaction network as proposed by [1] is performed. |
| **CellGrowthObserver** | Does not observe anything, but it controls the reaction networks cell. |
| **DecayingDimerizingHistogramDistances** | Demonstration of performance and accuracy differences of the different simulation algorithms. |
| **DecayingDimerizingInteractive** | Demonstration of performance and accuracy differences of the different simulation algorithms. |
| **DecayingDimerizingPlots** | **Deprecated.** |
| **Dsmts** | Perform a series of tests (refer to http://www.calibayes.ncl.ac.uk/Resources/dsmts). |
| **ExamplePath** | Determine the path to the example network files. |
| **HistogramDistanceTestSet** | Encapsulate test sets for histogram distance calculations. |
| **IrreversibleIsomerization** | Uses the Irreversible-isomerization model to show effects of different choices for epsilon. |
| **LacYComplete** | The LacZ/LacY model of procaryotic gene expression proposed by [1] is simulated. |
| **LacYHistogramDistances** | The LacZ/LacY model of procaryotic gene expression proposed by [1] is simulated. |
| **LacZ** | The LacZ/LacY model of procaryotic gene expression proposed by [1] is simulated. |
| **MapkBenchmark** | Use the signal transduction pathway network of the epidermal growth factor proposed by [1] to introduce the benchmark system. |
| **MichaelisMentenKinetic** | The most basic example uses the famous enzyme kinetics equation by Michaelis and Menten S + E <-> ES -> P to introduce fundamental loading and repeated simulation of reaction networks. |
| **SBMLMathTreeTest** |  |

## Package fern.example Description

Contains examples and demonstrations of the framework.

---


|  |  |  |  |  |  |  |  |  |  |  |
| --- | --- | --- | --- | --- | --- | --- | --- | --- | --- | --- |
| |  |  |  |  |  |  |  |  | | --- | --- | --- | --- | --- | --- | --- | --- | | **Overview** | **Package** | Class | **Use** | **Tree** | **Deprecated** | **Index** | **Help** | | |  |
| **PREV PACKAGE**   **NEXT PACKAGE** | **FRAMES**    **NO FRAMES**     **All Classes** |


---
