# Supplementary material for: FERN – a Java framework for stochastic simulation and evaluation of reaction networks
Source: BMC Bioinformatics. 2008 Aug 29;9:356. doi: 10.1186/1471-2105-9-356 (PMC2553347; doi:10.1186/1471-2105-9-356)
Supplement: Additional file 1 — FERN distribution, Version 1.3. This archive contains the FERN source code and binaries as well as documentation and example models in FernML and SBML. [file 1471-2105-9-356-S1.zip › fern/doc/javadoc/fern/example/package-tree.html]

fern.example Class Hierarchy


---


|  |  |  |  |  |  |  |  |  |  |  |
| --- | --- | --- | --- | --- | --- | --- | --- | --- | --- | --- |
| |  |  |  |  |  |  |  |  | | --- | --- | --- | --- | --- | --- | --- | --- | | **Overview** | **Package** | Class | Use | **Tree** | **Deprecated** | **Index** | **Help** | | |  |
| **PREV**   **NEXT** | **FRAMES**    **NO FRAMES**     **All Classes** |


---


## Hierarchy For Package fern.example

**Package Hierarchies:**: All Packages

---

## Class Hierarchy

- java.lang.**Object**
  - fern.example.**AutocatalyticNetworkExample**- fern.example.**DecayingDimerizingHistogramDistances**- fern.example.**DecayingDimerizingInteractive**- fern.example.**DecayingDimerizingPlots**- fern.example.**Dsmts**- fern.example.**ExamplePath**- fern.example.**HistogramDistanceTestSet**- fern.example.**IrreversibleIsomerization**- fern.example.**LacYComplete**- fern.example.**LacYHistogramDistances**- fern.example.**LacZ**- fern.example.**MapkBenchmark**- fern.example.**MichaelisMentenKinetic**- fern.simulation.observer.**Observer**
                              - fern.example.**CellGrowthObserver**- fern.example.**SBMLMathTreeTest**

---


|  |  |  |  |  |  |  |  |  |  |  |
| --- | --- | --- | --- | --- | --- | --- | --- | --- | --- | --- |
| |  |  |  |  |  |  |  |  | | --- | --- | --- | --- | --- | --- | --- | --- | | **Overview** | **Package** | Class | Use | **Tree** | **Deprecated** | **Index** | **Help** | | |  |
| **PREV**   **NEXT** | **FRAMES**    **NO FRAMES**     **All Classes** |


---
