# Supplementary material for: FERN – a Java framework for stochastic simulation and evaluation of reaction networks
Source: BMC Bioinformatics. 2008 Aug 29;9:356. doi: 10.1186/1471-2105-9-356 (PMC2553347; doi:10.1186/1471-2105-9-356)
Supplement: Additional file 1 — FERN distribution, Version 1.3. This archive contains the FERN source code and binaries as well as documentation and example models in FernML and SBML. [file 1471-2105-9-356-S1.zip › fern/doc/javadoc/fern/network/AbstractKineticConstantPropensityCalculator.html]

AbstractKineticConstantPropensityCalculator


---


|  |  |  |  |  |  |  |  |  |  |  |
| --- | --- | --- | --- | --- | --- | --- | --- | --- | --- | --- |
| |  |  |  |  |  |  |  |  | | --- | --- | --- | --- | --- | --- | --- | --- | | **Overview** | **Package** | **Class** | **Use** | **Tree** | **Deprecated** | **Index** | **Help** | | |  |
| PREV CLASS   **NEXT CLASS** | **FRAMES**    **NO FRAMES**     **All Classes** |
| SUMMARY: NESTED | FIELD | CONSTR | METHOD | DETAIL: FIELD | CONSTR | METHOD |


---


## fern.network Class AbstractKineticConstantPropensityCalculator

```
java.lang.Object
  fern.network.AbstractKineticConstantPropensityCalculator
```

**All Implemented Interfaces:**: PropensityCalculator

**Direct Known Subclasses:**: ArrayKineticConstantPropensityCalculator

---

``` public abstract class AbstractKineticConstantPropensityCalculator extends Object implements PropensityCalculator ```

Base implementation of a `PropensityCalculator`. The propensity is simply
a product of the populations of its reactant species and a specific reaction
probability rate constant (which is related to the conventional deterministic
rate constant and can be calculated by `getConstantFromDeterministicRateConstant`.

Some of the tau leap procedures need partial derivatives of the propensity function (and
use therefore the method `calculatePartialDerivative`) so the use of this
procedures is only possible when the network's `PropensityCalculator`
is a `AbstractKineticConstantPropensityCalculator`.

**Author:**
:   Florian Erhard

---

| **Constructor Summary** | |
| --- | --- |
| `AbstractKineticConstantPropensityCalculator(int[][] reactants)`             Creates a `AbstractKineticConstantPropensityCalculator` by an array of adjacency arrays for the reaction's reactant species (which are the only one needed for the propensity calculation). |


| **Method Summary** | |
| --- | --- |
| `double` | `calculatePartialDerivative(int reaction, AmountManager amount, int reactantIndex, double volume)`             Calculates partial differentials of the propensity functions for the tau leaping methods. |
| `double` | `calculatePropensity(int reaction, AmountManager amount, Simulator sim)`             Calculates the propensity for `reaction` by the formula h\*c, where c is the kinetic constant for `reaction` and h is the number of distinct molecular reactant combinations for `reaction`. |
| `abstract  double` | `getConstant(int reaction)`             Gets the constant for a reaction |
| `double` | `getConstantFromDeterministicRateConstant(double k, int reaction, double V)`             Calculates the specific reaction probability rate constant c from the conventional deterministic rate constant k in some fixed volume v by the formula c=|reactants| ! |

| **Methods inherited from class java.lang.Object** |
| --- |
| `clone, equals, finalize, getClass, hashCode, notify, notifyAll, toString, wait, wait, wait` |

| **Constructor Detail** |
| --- |

### AbstractKineticConstantPropensityCalculator

```
public AbstractKineticConstantPropensityCalculator(int[][] reactants)
```

:   Creates a `AbstractKineticConstantPropensityCalculator` by an array of
    adjacency arrays for the reaction's reactant species (which are the only one needed
    for the propensity calculation).

    **Parameters:**: `reactants` - array of adjacency arrays


| **Method Detail** |
| --- |

### calculatePropensity

```
public double calculatePropensity(int reaction,
                                  AmountManager amount,
                                  Simulator sim)
```

:   Calculates the propensity for `reaction` by the formula h\*c, where
    c is the kinetic constant for `reaction` and h is the number of
    distinct molecular reactant combinations for `reaction`. If a positive
    value for volume is given, it is assumed that the constants are deterministic rate
    constants and are hence transformed to specific reaction rate constants.

    :   **Specified by:**: `calculatePropensity` in interface `PropensityCalculator`
    :   **Parameters:**: `reaction` - the index of the reaction: `amount` - the `AmountManager`: `sim` - the simulator **Returns:**: the propensity for the reaction

---


### calculatePartialDerivative

```
public double calculatePartialDerivative(int reaction,
                                         AmountManager amount,
                                         int reactantIndex,
                                         double volume)
```

:   Calculates partial differentials of the propensity functions for the tau leaping
    methods. If a positive
    value for volume is given, it is assumed that the constants are deterministic rate
    constants and are hence transformed to specific reaction rate constants.

    :   **Parameters:**: `reaction` - the reaction index: `amount` - the `AmountManager`: `reactantIndex` - the network index of the reactant to calculate the partial differential for: `volume` - the volume of the reaction space **Returns:**: partial differential

---


### getConstant

```
public abstract double getConstant(int reaction)
```

:   Gets the constant for a reaction

    :   **Parameters:**: `reaction` - index of the reaction **Returns:**: constant for the reaction

---


### getConstantFromDeterministicRateConstant

```
public double getConstantFromDeterministicRateConstant(double k,
                                                       int reaction,
                                                       double V)
```

:   Calculates the specific reaction probability rate constant c from the conventional
    deterministic rate constant k in some fixed volume v by the formula
    c=|reactants| ! \* k / V^(|reactants|-1)
      
      
    For references see Daniel T. Gillespie, A General Method for Numerically Simulating
    the Stochastic Time Evolution of Coupled Chemical Reactions, Journal of Computational
    Physics 22, 403-434 (1976)

    :   **Parameters:**: `k` - deterministic rate constant: `reaction` - the index of the constant's reaction: `V` - the fixed volume **Returns:**: the specific reaction probability rate constant


---


|  |  |  |  |  |  |  |  |  |  |  |
| --- | --- | --- | --- | --- | --- | --- | --- | --- | --- | --- |
| |  |  |  |  |  |  |  |  | | --- | --- | --- | --- | --- | --- | --- | --- | | **Overview** | **Package** | **Class** | **Use** | **Tree** | **Deprecated** | **Index** | **Help** | | |  |
| PREV CLASS   **NEXT CLASS** | **FRAMES**    **NO FRAMES**     **All Classes** |
| SUMMARY: NESTED | FIELD | CONSTR | METHOD | DETAIL: FIELD | CONSTR | METHOD |


---
