# Supplementary material for: FERN – a Java framework for stochastic simulation and evaluation of reaction networks
Source: BMC Bioinformatics. 2008 Aug 29;9:356. doi: 10.1186/1471-2105-9-356 (PMC2553347; doi:10.1186/1471-2105-9-356)
Supplement: Additional file 1 — FERN distribution, Version 1.3. This archive contains the FERN source code and binaries as well as documentation and example models in FernML and SBML. [file 1471-2105-9-356-S1.zip › fern/doc/javadoc/fern/network/AbstractNetworkImpl.html]

AbstractNetworkImpl


---


|  |  |  |  |  |  |  |  |  |  |  |
| --- | --- | --- | --- | --- | --- | --- | --- | --- | --- | --- |
| |  |  |  |  |  |  |  |  | | --- | --- | --- | --- | --- | --- | --- | --- | | **Overview** | **Package** | **Class** | **Use** | **Tree** | **Deprecated** | **Index** | **Help** | | |  |
| **PREV CLASS**   **NEXT CLASS** | **FRAMES**    **NO FRAMES**     **All Classes** |
| SUMMARY: NESTED | FIELD | CONSTR | METHOD | DETAIL: FIELD | CONSTR | METHOD |


---


## fern.network Class AbstractNetworkImpl

```
java.lang.Object
  fern.network.AbstractNetworkImpl
```

**All Implemented Interfaces:**: Network

**Direct Known Subclasses:**: AutocatalyticNetwork, CellDesignerNetworkWrapper, CytoscapeNetworkWrapper, FernMLNetwork, SBMLNetwork

---

``` public abstract class AbstractNetworkImpl extends Object implements Network ```

Base implementation for the `Network` interface. Implementing class only have
to create all the protected fields.

**Author:**
:   Florian Erhard

---

| **Field Summary** | |
| --- | --- |
| `protected  int[][]` | `adjListPro`             Stores the adjacency lists of reactions to their products. |
| `protected  int[][]` | `adjListRea`             Stores the adjacency lists of reactions to their reactants. |
| `protected  AmountManager` | `amountManager`             Stores the `AmountManager` of the network. |
| `protected  AnnotationManager` | `annotationManager`             Stores the `AnnotationManager` of the network. |
| `protected  String[]` | `indexToSpeciesId`             Stores a mapping from species indices to their names. |
| `protected  String` | `name`             Stores the network's identifier. |
| `protected  PropensityCalculator` | `propensitiyCalculator`             Stores the `PropensityCalculator` of the network. |
| `protected  Map<String,Integer>` | `speciesIdToIndex`             Stores a mapping from species names to their indices. |


| **Constructor Summary** | |
| --- | --- |
| `AbstractNetworkImpl(String name)`             Create the network and give it an identifier. |


| **Method Summary** | |
| --- | --- |
| `protected abstract  void` | `createAdjacencyLists()`             Reminds extending class to fill `adjListPro` and `adjListRea`. |
| `protected abstract  void` | `createAmountManager()`             Reminds extending class to fill `amountManager`. |
| `protected abstract  void` | `createAnnotationManager()`             Reminds extending class to fill `annotationManager`. |
| `protected abstract  void` | `createPropensityCalulator()`             Reminds extending class to fill `propensitiyCalculator`. |
| `protected abstract  void` | `createSpeciesMapping()`             Reminds extending class to fill `speciesIdToIndex` and `indexToSpeciesId`. |
| `AmountManager` | `getAmountManager()`             Gets the `AmountManager` for this network. |
| `AnnotationManager` | `getAnnotationManager()`             Gets the `AnnotationManager` for this network. |
| `String` | `getName()`             Gets an identifier of the network. |
| `int` | `getNumReactions()`             Gets the number of reaction within the network. |
| `int` | `getNumSpecies()`             Gets the number of species within the network. |
| `int[]` | `getProducts(int reaction)`             Gets the products of the specified reaction. |
| `PropensityCalculator` | `getPropensityCalculator()`             Gets the `PropensityCalculator` for this network. |
| `int[]` | `getReactants(int reaction)`             Gets the reactants of the specified reaction. |
| `String` | `getReactionName(int index)`             Gets a string representation of the reaction. |
| `int` | `getSpeciesByName(String name)`             Gets the species index by name. |
| `Map<String,Integer>` | `getSpeciesMapping()`             Gets the mapping from species names to their indices. |
| `String` | `getSpeciesName(int index)`             Gets the species name by index. |

| **Methods inherited from class java.lang.Object** |
| --- |
| `clone, equals, finalize, getClass, hashCode, notify, notifyAll, toString, wait, wait, wait` |

| **Methods inherited from interface fern.network.Network** |
| --- |
| `getInitialAmount, setInitialAmount` |

| **Field Detail** |
| --- |

### propensitiyCalculator

```
protected PropensityCalculator propensitiyCalculator
```

:   Stores the `PropensityCalculator` of the network.

---


### amountManager

```
protected AmountManager amountManager
```

:   Stores the `AmountManager` of the network.

---


### annotationManager

```
protected AnnotationManager annotationManager
```

:   Stores the `AnnotationManager` of the network.

---


### adjListPro

```
protected int[][] adjListPro
```

:   Stores the adjacency lists of reactions to their products.

---


### adjListRea

```
protected int[][] adjListRea
```

:   Stores the adjacency lists of reactions to their reactants.

---


### speciesIdToIndex

```
protected Map<String,Integer> speciesIdToIndex
```

:   Stores a mapping from species names to their indices.

---


### indexToSpeciesId

```
protected String[] indexToSpeciesId
```

:   Stores a mapping from species indices to their names.

---


### name

```
protected String name
```

:   Stores the network's identifier.


| **Constructor Detail** |
| --- |

### AbstractNetworkImpl

```
public AbstractNetworkImpl(String name)
```

:   Create the network and give it an identifier.

    **Parameters:**: `name` - identifier for the network


| **Method Detail** |
| --- |

### createAnnotationManager

```
protected abstract void createAnnotationManager()
```

:   Reminds extending class to fill `annotationManager`.

---


### createSpeciesMapping

```
protected abstract void createSpeciesMapping()
```

:   Reminds extending class to fill `speciesIdToIndex` and `indexToSpeciesId`.

---


### createAdjacencyLists

```
protected abstract void createAdjacencyLists()
```

:   Reminds extending class to fill `adjListPro` and `adjListRea`.

---


### createAmountManager

```
protected abstract void createAmountManager()
```

:   Reminds extending class to fill `amountManager`.

---


### createPropensityCalulator

```
protected abstract void createPropensityCalulator()
```

:   Reminds extending class to fill `propensitiyCalculator`.

---


### getAmountManager

```
public AmountManager getAmountManager()
```

:   **Description copied from interface: `Network`**
:   Gets the `AmountManager` for this network.

    :   **Specified by:**: `getAmountManager` in interface `Network`
    :   **Returns:**: the `AmountManager`

---


### getPropensityCalculator

```
public PropensityCalculator getPropensityCalculator()
```

:   **Description copied from interface: `Network`**
:   Gets the `PropensityCalculator` for this network.

    :   **Specified by:**: `getPropensityCalculator` in interface `Network`
    :   **Returns:**: the `PropensityCalculator`

---


### getAnnotationManager

```
public AnnotationManager getAnnotationManager()
```

:   **Description copied from interface: `Network`**
:   Gets the `AnnotationManager` for this network.

    :   **Specified by:**: `getAnnotationManager` in interface `Network`
    :   **Returns:**: the `AnnotationManager`

---


### getNumReactions

```
public int getNumReactions()
```

:   **Description copied from interface: `Network`**
:   Gets the number of reaction within the network.

    :   **Specified by:**: `getNumReactions` in interface `Network`
    :   **Returns:**: number of reactions

---


### getNumSpecies

```
public int getNumSpecies()
```

:   **Description copied from interface: `Network`**
:   Gets the number of species within the network.

    :   **Specified by:**: `getNumSpecies` in interface `Network`
    :   **Returns:**: number of species

---


### getProducts

```
public int[] getProducts(int reaction)
```

:   **Description copied from interface: `Network`**
:   Gets the products of the specified reaction.

    :   **Specified by:**: `getProducts` in interface `Network`
    :   **Parameters:**: `reaction` - index of the reaction **Returns:**: indices of the products

---


### getReactants

```
public int[] getReactants(int reaction)
```

:   **Description copied from interface: `Network`**
:   Gets the reactants of the specified reaction.

    :   **Specified by:**: `getReactants` in interface `Network`
    :   **Parameters:**: `reaction` - index of the reaction **Returns:**: indices of the reactants

---


### getSpeciesByName

```
public int getSpeciesByName(String name)
```

:   **Description copied from interface: `Network`**
:   Gets the species index by name. If the argument is no valid species, -1 is returned.

    :   **Specified by:**: `getSpeciesByName` in interface `Network`
    :   **Parameters:**: `name` - name of the species **Returns:**: index of the species

---


### getSpeciesMapping

```
public Map<String,Integer> getSpeciesMapping()
```

:   Gets the mapping from species names to their indices.

    :   **Returns:**: species mapping

---


### getSpeciesName

```
public String getSpeciesName(int index)
```

:   **Description copied from interface: `Network`**
:   Gets the species name by index.

    :   **Specified by:**: `getSpeciesName` in interface `Network`
    :   **Parameters:**: `index` - index of the species **Returns:**: name of the species

---


### getReactionName

```
public String getReactionName(int index)
```

:   **Description copied from interface: `Network`**
:   Gets a string representation of the reaction.

    :   **Specified by:**: `getReactionName` in interface `Network`
    :   **Parameters:**: `index` - reaction index **Returns:**: string representation

---


### getName

```
public String getName()
```

:   **Description copied from interface: `Network`**
:   Gets an identifier of the network.

    :   **Specified by:**: `getName` in interface `Network`
    :   **Returns:**: identifier of the network


---


|  |  |  |  |  |  |  |  |  |  |  |
| --- | --- | --- | --- | --- | --- | --- | --- | --- | --- | --- |
| |  |  |  |  |  |  |  |  | | --- | --- | --- | --- | --- | --- | --- | --- | | **Overview** | **Package** | **Class** | **Use** | **Tree** | **Deprecated** | **Index** | **Help** | | |  |
| **PREV CLASS**   **NEXT CLASS** | **FRAMES**    **NO FRAMES**     **All Classes** |
| SUMMARY: NESTED | FIELD | CONSTR | METHOD | DETAIL: FIELD | CONSTR | METHOD |


---
