# Supplementary material for: FERN – a Java framework for stochastic simulation and evaluation of reaction networks
Source: BMC Bioinformatics. 2008 Aug 29;9:356. doi: 10.1186/1471-2105-9-356 (PMC2553347; doi:10.1186/1471-2105-9-356)
Supplement: Additional file 1 — FERN distribution, Version 1.3. This archive contains the FERN source code and binaries as well as documentation and example models in FernML and SBML. [file 1471-2105-9-356-S1.zip › fern/doc/javadoc/fern/network/AmountManager.html]

AmountManager


---


|  |  |  |  |  |  |  |  |  |  |  |
| --- | --- | --- | --- | --- | --- | --- | --- | --- | --- | --- |
| |  |  |  |  |  |  |  |  | | --- | --- | --- | --- | --- | --- | --- | --- | | **Overview** | **Package** | **Class** | **Use** | **Tree** | **Deprecated** | **Index** | **Help** | | |  |
| **PREV CLASS**   **NEXT CLASS** | **FRAMES**    **NO FRAMES**     **All Classes** |
| SUMMARY: NESTED | FIELD | CONSTR | METHOD | DETAIL: FIELD | CONSTR | METHOD |


---


## fern.network Class AmountManager

```
java.lang.Object
  fern.network.AmountManager
```

---

``` public class AmountManager extends Object ```

The `AmountManager` is one of the most important connections between a
`Network` and a `Simulator`. Each `Simulator` calls the
`performReaction` method when it fires a reaction. The amount manager then
reflects the change of its reactant / product populations. Additionally, the
`PropensityCalculator` uses `getAmount` to calculate the propensity
of a reaction.

It is also possible (and necessary for the tau leaping algorithms) to save the actual
amount of each species and, if some error happened, restore these saved values.

The amounts are stored in an array.

**Author:**
:   Florian Erhard

---

| **Constructor Summary** | |
| --- | --- |
| `AmountManager(Network net)`             Creates an `AmountManager` for a given network |


| **Method Summary** | |
| --- | --- |
| `long` | `getAmount(int species)`             Gets the current amount of a species. |
| `void` | `performReaction(int reaction, int times)`             Reflects a (multiple) firing of a reaction by adjusting the populations of the reactants and the products. |
| `void` | `resetAmount()`             Resets the amount of each species to the initial amount retrieved by the networks `AnnotationManager`. |
| `void` | `rollback()`             Restore the amount array from the recently saved one. |
| `void` | `save()`             Makes a copy of the amount array. |
| `void` | `setAmount(int species, long amount)`             Sets the current amount of a species. |

| **Methods inherited from class java.lang.Object** |
| --- |
| `clone, equals, finalize, getClass, hashCode, notify, notifyAll, toString, wait, wait, wait` |

| **Constructor Detail** |
| --- |

### AmountManager

```
public AmountManager(Network net)
```

:   Creates an `AmountManager` for a given network

    **Parameters:**: `net` - the network


| **Method Detail** |
| --- |

### performReaction

```
public void performReaction(int reaction,
                            int times)
```

:   Reflects a (multiple) firing of a reaction by adjusting the populations of the
    reactants and the products. If a population becomes negative, a `RuntimeException` is thrown.

    :   **Parameters:**: `reaction` - the index of the reaction fired: `times` - the number of firings

---


### getAmount

```
public long getAmount(int species)
```

:   Gets the current amount of a species.

    :   **Parameters:**: `species` - index of the species **Returns:**: actual amount of the species

---


### setAmount

```
public void setAmount(int species,
                      long amount)
```

:   Sets the current amount of a species.

    :   **Parameters:**: `species` - index of the species

---


### resetAmount

```
public void resetAmount()
```

:   Resets the amount of each species to the initial amount retrieved by the networks
    `AnnotationManager`. This is called whenever a `Simulator` is started.

---


### save

```
public void save()
```

:   Makes a copy of the amount array.

---


### rollback

```
public void rollback()
```

:   Restore the amount array from the recently saved one.


---


|  |  |  |  |  |  |  |  |  |  |  |
| --- | --- | --- | --- | --- | --- | --- | --- | --- | --- | --- |
| |  |  |  |  |  |  |  |  | | --- | --- | --- | --- | --- | --- | --- | --- | | **Overview** | **Package** | **Class** | **Use** | **Tree** | **Deprecated** | **Index** | **Help** | | |  |
| **PREV CLASS**   **NEXT CLASS** | **FRAMES**    **NO FRAMES**     **All Classes** |
| SUMMARY: NESTED | FIELD | CONSTR | METHOD | DETAIL: FIELD | CONSTR | METHOD |


---
