# Supplementary material for: FERN – a Java framework for stochastic simulation and evaluation of reaction networks
Source: BMC Bioinformatics. 2008 Aug 29;9:356. doi: 10.1186/1471-2105-9-356 (PMC2553347; doi:10.1186/1471-2105-9-356)
Supplement: Additional file 1 — FERN distribution, Version 1.3. This archive contains the FERN source code and binaries as well as documentation and example models in FernML and SBML. [file 1471-2105-9-356-S1.zip › fern/doc/javadoc/fern/network/AnnotationManager.html]

AnnotationManager


---


|  |  |  |  |  |  |  |  |  |  |  |
| --- | --- | --- | --- | --- | --- | --- | --- | --- | --- | --- |
| |  |  |  |  |  |  |  |  | | --- | --- | --- | --- | --- | --- | --- | --- | | **Overview** | **Package** | **Class** | **Use** | **Tree** | **Deprecated** | **Index** | **Help** | | |  |
| **PREV CLASS**   **NEXT CLASS** | **FRAMES**    **NO FRAMES**     **All Classes** |
| SUMMARY: NESTED | FIELD | CONSTR | METHOD | DETAIL: FIELD | CONSTR | METHOD |


---


## fern.network Interface AnnotationManager

**All Known Implementing Classes:**: AnnotationManagerImpl, CytoscapeAnnotationManager, FernMLAnnotationManager

---

``` public interface AnnotationManager ```

AnnotationManager objects are the places, where static (or quasi-static) properties
of a network are stored. At this time, all the annotations and the initial amounts of the species
belong to the static properties. Annotations are
only supposed to hold data which

- almost never changes
- is read very infrequently

**Author:**
:   Florian Erhard

---

| **Method Summary** | |
| --- | --- |
| `boolean` | `containsNetworkAnnotation(String typ)`             Returns true iff the network contains an annotation of the specified type. |
| `boolean` | `containsReactionAnnotation(int reaction, String typ)`             Returns true iff the reaction contains an annotation of the specified type. |
| `boolean` | `containsSpeciesAnnotation(int species, String typ)`             Returns true iff the species contains an annotation of the specified type. |
| `String` | `getNetworkAnnotation(String typ)`             Gets the network annotation of the specified field. |
| `Collection<String>` | `getNetworkAnnotationTypes()`             Gets the names of the network reaction. |
| `String` | `getReactionAnnotation(int reaction, String typ)`             Gets the reaction annotation of the specified field. |
| `Collection<String>` | `getReactionAnnotationTypes(int reaction)`             Gets the names of the species reaction. |
| `String` | `getSpeciesAnnotation(int species, String typ)`             Gets the species annotation of the specified field. |
| `Collection<String>` | `getSpeciesAnnotationTypes(int species)`             Gets the names of the species annotations. |
| `void` | `setNetworkAnnotation(String typ, String annotation)`             Sets the network annotation of the specified field. |
| `void` | `setReactionAnnotation(int reaction, String typ, String annotation)`             Sets the reaction annotation of the specified field. |
| `void` | `setSpeciesAnnotation(int species, String typ, String annotation)`             Sets the species annotation of the specified field. |

| **Method Detail** |
| --- |

### containsSpeciesAnnotation

```
boolean containsSpeciesAnnotation(int species,
                                  String typ)
```

:   Returns true iff the species contains an annotation of the specified type.

    :   **Parameters:**: `species` - index of the species: `typ` - field name of the annotation **Returns:**: true iff such an annotation is present

---


### getSpeciesAnnotationTypes

```
Collection<String> getSpeciesAnnotationTypes(int species)
```

:   Gets the names of the species annotations.

    :   **Parameters:**: `species` - index of the species **Returns:**: names of the fields

---


### getSpeciesAnnotation

```
String getSpeciesAnnotation(int species,
                            String typ)
```

:   Gets the species annotation of the specified field.

    :   **Parameters:**: `species` - index of the species: `typ` - name of the field **Returns:**: species annotation

---


### setSpeciesAnnotation

```
void setSpeciesAnnotation(int species,
                          String typ,
                          String annotation)
```

:   Sets the species annotation of the specified field.

    :   **Parameters:**: `species` - index of the species: `typ` - name of the field: `annotation` - species annotation

---


### containsReactionAnnotation

```
boolean containsReactionAnnotation(int reaction,
                                   String typ)
```

:   Returns true iff the reaction contains an annotation of the specified type.

    :   **Parameters:**: `reaction` - index of the reaction: `typ` - field name of the annotation **Returns:**: true iff such an annotation is present

---


### getReactionAnnotationTypes

```
Collection<String> getReactionAnnotationTypes(int reaction)
```

:   Gets the names of the species reaction.

    :   **Parameters:**: `reaction` - index of the reaction **Returns:**: names of the fields

---


### getReactionAnnotation

```
String getReactionAnnotation(int reaction,
                             String typ)
```

:   Gets the reaction annotation of the specified field.

    :   **Parameters:**: `reaction` - index of the reaction: `typ` - name of the field **Returns:**: reaction annotation

---


### setReactionAnnotation

```
void setReactionAnnotation(int reaction,
                           String typ,
                           String annotation)
```

:   Sets the reaction annotation of the specified field.

    :   **Parameters:**: `reaction` - index of the reaction: `typ` - name of the field: `annotation` - reaction annotation

---


### containsNetworkAnnotation

```
boolean containsNetworkAnnotation(String typ)
```

:   Returns true iff the network contains an annotation of the specified type.

    :   **Parameters:**: `typ` - field name of the annotation **Returns:**: true iff such an annotation is present

---


### getNetworkAnnotationTypes

```
Collection<String> getNetworkAnnotationTypes()
```

:   Gets the names of the network reaction.

    :   **Returns:**: names of the fields

---


### getNetworkAnnotation

```
String getNetworkAnnotation(String typ)
```

:   Gets the network annotation of the specified field.

    :   **Parameters:**: `typ` - name of the field **Returns:**: network annotation

---


### setNetworkAnnotation

```
void setNetworkAnnotation(String typ,
                          String annotation)
```

:   Sets the network annotation of the specified field.

    :   **Parameters:**: `typ` - name of the field: `annotation` - network annotation


---


|  |  |  |  |  |  |  |  |  |  |  |
| --- | --- | --- | --- | --- | --- | --- | --- | --- | --- | --- |
| |  |  |  |  |  |  |  |  | | --- | --- | --- | --- | --- | --- | --- | --- | | **Overview** | **Package** | **Class** | **Use** | **Tree** | **Deprecated** | **Index** | **Help** | | |  |
| **PREV CLASS**   **NEXT CLASS** | **FRAMES**    **NO FRAMES**     **All Classes** |
| SUMMARY: NESTED | FIELD | CONSTR | METHOD | DETAIL: FIELD | CONSTR | METHOD |


---
