# Supplementary material for: FERN – a Java framework for stochastic simulation and evaluation of reaction networks
Source: BMC Bioinformatics. 2008 Aug 29;9:356. doi: 10.1186/1471-2105-9-356 (PMC2553347; doi:10.1186/1471-2105-9-356)
Supplement: Additional file 1 — FERN distribution, Version 1.3. This archive contains the FERN source code and binaries as well as documentation and example models in FernML and SBML. [file 1471-2105-9-356-S1.zip › fern/doc/javadoc/fern/network/ArrayKineticConstantPropensityCalculator.html]

ArrayKineticConstantPropensityCalculator


---


|  |  |  |  |  |  |  |  |  |  |  |
| --- | --- | --- | --- | --- | --- | --- | --- | --- | --- | --- |
| |  |  |  |  |  |  |  |  | | --- | --- | --- | --- | --- | --- | --- | --- | | **Overview** | **Package** | **Class** | **Use** | **Tree** | **Deprecated** | **Index** | **Help** | | |  |
| **PREV CLASS**   **NEXT CLASS** | **FRAMES**    **NO FRAMES**     **All Classes** |
| SUMMARY: NESTED | FIELD | CONSTR | METHOD | DETAIL: FIELD | CONSTR | METHOD |


---


## fern.network Class ArrayKineticConstantPropensityCalculator

```
java.lang.Object
  fern.network.AbstractKineticConstantPropensityCalculator
      fern.network.ArrayKineticConstantPropensityCalculator
```

**All Implemented Interfaces:**: PropensityCalculator

---

``` public class ArrayKineticConstantPropensityCalculator extends AbstractKineticConstantPropensityCalculator ```

Implementation of an `AbstractKineticConstantPropensityCalculator` which uses
an array to store the constants for each reaction.

**Author:**
:   Florian Erhard

---

| **Constructor Summary** | |
| --- | --- |
| `ArrayKineticConstantPropensityCalculator(int[][] reactants, double[] constants)`             Create the propensity calculator with given constants and given reactant adjacency arrays |


| **Method Summary** | |
| --- | --- |
| `double` | `getConstant(int i)`             Gets the constant for a reaction |
| `void` | `setConstant(int i, double value)` |

| **Methods inherited from class fern.network.AbstractKineticConstantPropensityCalculator** |
| --- |
| `calculatePartialDerivative, calculatePropensity, getConstantFromDeterministicRateConstant` |

| **Methods inherited from class java.lang.Object** |
| --- |
| `clone, equals, finalize, getClass, hashCode, notify, notifyAll, toString, wait, wait, wait` |

| **Constructor Detail** |
| --- |

### ArrayKineticConstantPropensityCalculator

```
public ArrayKineticConstantPropensityCalculator(int[][] reactants,
                                                double[] constants)
```

:   Create the propensity calculator with given constants and given reactant
    adjacency arrays

    **Parameters:**: `reactants` - array of adjacency arrays: `constants` - array of kinetic constants


| **Method Detail** |
| --- |

### getConstant

```
public double getConstant(int i)
```

:   **Description copied from class: `AbstractKineticConstantPropensityCalculator`**
:   Gets the constant for a reaction

    :   **Specified by:**: `getConstant` in class `AbstractKineticConstantPropensityCalculator`
    :   **Parameters:**: `i` - index of the reaction **Returns:**: constant for the reaction

---


### setConstant

```
public void setConstant(int i,
                        double value)
```


---


|  |  |  |  |  |  |  |  |  |  |  |
| --- | --- | --- | --- | --- | --- | --- | --- | --- | --- | --- |
| |  |  |  |  |  |  |  |  | | --- | --- | --- | --- | --- | --- | --- | --- | | **Overview** | **Package** | **Class** | **Use** | **Tree** | **Deprecated** | **Index** | **Help** | | |  |
| **PREV CLASS**   **NEXT CLASS** | **FRAMES**    **NO FRAMES**     **All Classes** |
| SUMMARY: NESTED | FIELD | CONSTR | METHOD | DETAIL: FIELD | CONSTR | METHOD |


---
