# Supplementary material for: FERN – a Java framework for stochastic simulation and evaluation of reaction networks
Source: BMC Bioinformatics. 2008 Aug 29;9:356. doi: 10.1186/1471-2105-9-356 (PMC2553347; doi:10.1186/1471-2105-9-356)
Supplement: Additional file 1 — FERN distribution, Version 1.3. This archive contains the FERN source code and binaries as well as documentation and example models in FernML and SBML. [file 1471-2105-9-356-S1.zip › fern/doc/javadoc/fern/network/class-use/AbstractNetworkImpl.html]

Uses of Class fern.network.AbstractNetworkImpl


---


|  |  |  |  |  |  |  |  |  |  |  |
| --- | --- | --- | --- | --- | --- | --- | --- | --- | --- | --- |
| |  |  |  |  |  |  |  |  | | --- | --- | --- | --- | --- | --- | --- | --- | | **Overview** | **Package** | **Class** | **Use** | **Tree** | **Deprecated** | **Index** | **Help** | | |  |
| PREV   NEXT | **FRAMES**    **NO FRAMES**     **All Classes** |


---


## **Uses of Class fern.network.AbstractNetworkImpl**

| Packages that use AbstractNetworkImpl | |
| --- | --- |
| **fern.cellDesigner** |  |
| **fern.cytoscape** | Provides the classes for the cytoscape plugin. |
| **fern.network.creation** | Provides classes for the evolution of networks. |
| **fern.network.fernml** | Provides the classes for parsing and using FernML based networks. |
| **fern.network.sbml** | Provides the classes for parsing and using sbml based networks. |

| Uses of AbstractNetworkImpl in fern.cellDesigner | |
| --- | --- |

| Subclasses of AbstractNetworkImpl in fern.cellDesigner | |
| --- | --- |
| `class` | `CellDesignerNetworkWrapper` |

| Uses of AbstractNetworkImpl in fern.cytoscape | |
| --- | --- |

| Subclasses of AbstractNetworkImpl in fern.cytoscape | |
| --- | --- |
| `class` | `CytoscapeNetworkWrapper` |

| Uses of AbstractNetworkImpl in fern.network.creation | |
| --- | --- |

| Subclasses of AbstractNetworkImpl in fern.network.creation | |
| --- | --- |
| `class` | `AutocatalyticNetwork`             Evolve an autocatalytic network. |

| Uses of AbstractNetworkImpl in fern.network.fernml | |
| --- | --- |

| Subclasses of AbstractNetworkImpl in fern.network.fernml | |
| --- | --- |
| `class` | `FernMLNetwork`             A `FernMLNetwork` is usually loaded from a file. |

| Uses of AbstractNetworkImpl in fern.network.sbml | |
| --- | --- |

| Subclasses of AbstractNetworkImpl in fern.network.sbml | |
| --- | --- |
| `class` | `SBMLNetwork`             For specifications of the sbml format refer to http:\\www.sbml.org. |

---


|  |  |  |  |  |  |  |  |  |  |  |
| --- | --- | --- | --- | --- | --- | --- | --- | --- | --- | --- |
| |  |  |  |  |  |  |  |  | | --- | --- | --- | --- | --- | --- | --- | --- | | **Overview** | **Package** | **Class** | **Use** | **Tree** | **Deprecated** | **Index** | **Help** | | |  |
| PREV   NEXT | **FRAMES**    **NO FRAMES**     **All Classes** |


---
