# Supplementary material for: FERN – a Java framework for stochastic simulation and evaluation of reaction networks
Source: BMC Bioinformatics. 2008 Aug 29;9:356. doi: 10.1186/1471-2105-9-356 (PMC2553347; doi:10.1186/1471-2105-9-356)
Supplement: Additional file 1 — FERN distribution, Version 1.3. This archive contains the FERN source code and binaries as well as documentation and example models in FernML and SBML. [file 1471-2105-9-356-S1.zip › fern/doc/javadoc/fern/network/class-use/AmountManager.html]

Uses of Class fern.network.AmountManager


---


|  |  |  |  |  |  |  |  |  |  |  |
| --- | --- | --- | --- | --- | --- | --- | --- | --- | --- | --- |
| |  |  |  |  |  |  |  |  | | --- | --- | --- | --- | --- | --- | --- | --- | | **Overview** | **Package** | **Class** | **Use** | **Tree** | **Deprecated** | **Index** | **Help** | | |  |
| PREV   NEXT | **FRAMES**    **NO FRAMES**     **All Classes** |


---


## **Uses of Class fern.network.AmountManager**

| Packages that use AmountManager | |
| --- | --- |
| **fern.cellDesigner** |  |
| **fern.network** | Provides general classes and interfaces for storing network data. |
| **fern.network.modification** | Provides classes for modifications of networks. |
| **fern.network.sbml** | Provides the classes for parsing and using sbml based networks. |
| **fern.simulation** | Provides algorithms for simulating reaction network as well as methods for observer certain aspects of a simulation. |

| Uses of AmountManager in fern.cellDesigner | |
| --- | --- |

| Methods in fern.cellDesigner with parameters of type AmountManager | |
| --- | --- |
| `double` | `CellDesignerPropensityCalculator.calculatePropensity(int reaction, AmountManager amount, Simulator sim)` |

| Uses of AmountManager in fern.network | |
| --- | --- |

| Fields in fern.network declared as AmountManager | |
| --- | --- |
| `protected  AmountManager` | `AbstractNetworkImpl.amountManager`             Stores the `AmountManager` of the network. |

| Methods in fern.network that return AmountManager | |
| --- | --- |
| `AmountManager` | `Network.getAmountManager()`             Gets the `AmountManager` for this network. |
| `AmountManager` | `AbstractNetworkImpl.getAmountManager()` |

| Methods in fern.network with parameters of type AmountManager | |
| --- | --- |
| `double` | `AbstractKineticConstantPropensityCalculator.calculatePartialDerivative(int reaction, AmountManager amount, int reactantIndex, double volume)`             Calculates partial differentials of the propensity functions for the tau leaping methods. |
| `double` | `PropensityCalculator.calculatePropensity(int reaction, AmountManager amount, Simulator sim)`             Calculates the propensity for a reaction given the amounts of the `AmountManager`. |
| `double` | `AbstractKineticConstantPropensityCalculator.calculatePropensity(int reaction, AmountManager amount, Simulator sim)`             Calculates the propensity for `reaction` by the formula h\*c, where c is the kinetic constant for `reaction` and h is the number of distinct molecular reactant combinations for `reaction`. |

| Uses of AmountManager in fern.network.modification | |
| --- | --- |

| Methods in fern.network.modification that return AmountManager | |
| --- | --- |
| `AmountManager` | `ReversibleNetwork.getAmountManager()`             Gets the `AmountManager` for the modified network. |
| `AmountManager` | `ModifierNetwork.getAmountManager()`             Gets the `AmountManager` of the original network. |
| `AmountManager` | `ExtractSubNetwork.getAmountManager()`             Gets the `AmountManager` for the extracted subnet. |
| `AmountManager` | `CatalysedNetwork.getAmountManager()` |

| Uses of AmountManager in fern.network.sbml | |
| --- | --- |

| Methods in fern.network.sbml with parameters of type AmountManager | |
| --- | --- |
| `double` | `MathTree.calculate(AmountManager amount, Simulator sim)`             Evaluate the MathTree. |
| `double` | `SBMLPropensityCalculator.calculatePropensity(int reaction, AmountManager amount, Simulator sim)` |

| Uses of AmountManager in fern.simulation | |
| --- | --- |

| Methods in fern.simulation that return AmountManager | |
| --- | --- |
| `protected  AmountManager` | `Simulator.getAmountManager()`             Gets the `AmountManager`. |

---


|  |  |  |  |  |  |  |  |  |  |  |
| --- | --- | --- | --- | --- | --- | --- | --- | --- | --- | --- |
| |  |  |  |  |  |  |  |  | | --- | --- | --- | --- | --- | --- | --- | --- | | **Overview** | **Package** | **Class** | **Use** | **Tree** | **Deprecated** | **Index** | **Help** | | |  |
| PREV   NEXT | **FRAMES**    **NO FRAMES**     **All Classes** |


---
