# Supplementary material for: FERN – a Java framework for stochastic simulation and evaluation of reaction networks
Source: BMC Bioinformatics. 2008 Aug 29;9:356. doi: 10.1186/1471-2105-9-356 (PMC2553347; doi:10.1186/1471-2105-9-356)
Supplement: Additional file 1 — FERN distribution, Version 1.3. This archive contains the FERN source code and binaries as well as documentation and example models in FernML and SBML. [file 1471-2105-9-356-S1.zip › fern/doc/javadoc/fern/network/class-use/AnnotationManager.html]

Uses of Interface fern.network.AnnotationManager


---


|  |  |  |  |  |  |  |  |  |  |  |
| --- | --- | --- | --- | --- | --- | --- | --- | --- | --- | --- |
| |  |  |  |  |  |  |  |  | | --- | --- | --- | --- | --- | --- | --- | --- | | **Overview** | **Package** | **Class** | **Use** | **Tree** | **Deprecated** | **Index** | **Help** | | |  |
| PREV   NEXT | **FRAMES**    **NO FRAMES**     **All Classes** |


---


## **Uses of Interface fern.network.AnnotationManager**

| Packages that use AnnotationManager | |
| --- | --- |
| **fern.cytoscape** | Provides the classes for the cytoscape plugin. |
| **fern.network** | Provides general classes and interfaces for storing network data. |
| **fern.network.fernml** | Provides the classes for parsing and using FernML based networks. |
| **fern.network.modification** | Provides classes for modifications of networks. |

| Uses of AnnotationManager in fern.cytoscape | |
| --- | --- |

| Classes in fern.cytoscape that implement AnnotationManager | |
| --- | --- |
| `class` | `CytoscapeAnnotationManager` |

| Uses of AnnotationManager in fern.network | |
| --- | --- |

| Classes in fern.network that implement AnnotationManager | |
| --- | --- |
| `class` | `AnnotationManagerImpl`             Base implementation of the `AnnotationManager` interface. |

| Fields in fern.network declared as AnnotationManager | |
| --- | --- |
| `protected  AnnotationManager` | `AbstractNetworkImpl.annotationManager`             Stores the `AnnotationManager` of the network. |

| Methods in fern.network that return AnnotationManager | |
| --- | --- |
| `AnnotationManager` | `Network.getAnnotationManager()`             Gets the `AnnotationManager` for this network. |
| `AnnotationManager` | `AbstractNetworkImpl.getAnnotationManager()` |

| Uses of AnnotationManager in fern.network.fernml | |
| --- | --- |

| Classes in fern.network.fernml that implement AnnotationManager | |
| --- | --- |
| `class` | `FernMLAnnotationManager`             `AnnotationManager` for `FernMLNetwork`s. |

| Uses of AnnotationManager in fern.network.modification | |
| --- | --- |

| Methods in fern.network.modification that return AnnotationManager | |
| --- | --- |
| `AnnotationManager` | `ReversibleNetwork.getAnnotationManager()`             Gets the `AnnotationManager` for the modified network. |
| `AnnotationManager` | `ModifierNetwork.getAnnotationManager()`             Gets the `AnnotationManager` of the original network |
| `AnnotationManager` | `ExtractSubNetwork.getAnnotationManager()`             Gets an `AnnotationManager` for the redirected index space. |
| `AnnotationManager` | `CatalysedNetwork.getAnnotationManager()` |

---


|  |  |  |  |  |  |  |  |  |  |  |
| --- | --- | --- | --- | --- | --- | --- | --- | --- | --- | --- |
| |  |  |  |  |  |  |  |  | | --- | --- | --- | --- | --- | --- | --- | --- | | **Overview** | **Package** | **Class** | **Use** | **Tree** | **Deprecated** | **Index** | **Help** | | |  |
| PREV   NEXT | **FRAMES**    **NO FRAMES**     **All Classes** |


---
