# Supplementary material for: FERN – a Java framework for stochastic simulation and evaluation of reaction networks
Source: BMC Bioinformatics. 2008 Aug 29;9:356. doi: 10.1186/1471-2105-9-356 (PMC2553347; doi:10.1186/1471-2105-9-356)
Supplement: Additional file 1 — FERN distribution, Version 1.3. This archive contains the FERN source code and binaries as well as documentation and example models in FernML and SBML. [file 1471-2105-9-356-S1.zip › fern/doc/javadoc/fern/network/class-use/FeatureNotSupportedException.html]

Uses of Class fern.network.FeatureNotSupportedException


---


|  |  |  |  |  |  |  |  |  |  |  |
| --- | --- | --- | --- | --- | --- | --- | --- | --- | --- | --- |
| |  |  |  |  |  |  |  |  | | --- | --- | --- | --- | --- | --- | --- | --- | | **Overview** | **Package** | **Class** | **Use** | **Tree** | **Deprecated** | **Index** | **Help** | | |  |
| PREV   NEXT | **FRAMES**    **NO FRAMES**     **All Classes** |


---


## **Uses of Class fern.network.FeatureNotSupportedException**

| Packages that use FeatureNotSupportedException | |
| --- | --- |
| **fern.example** | Contains examples and demonstrations of the framework. |
| **fern.network** | Provides general classes and interfaces for storing network data. |
| **fern.network.sbml** | Provides the classes for parsing and using sbml based networks. |
| **fern.tools** | Provides common used tools. |

| Uses of FeatureNotSupportedException in fern.example | |
| --- | --- |

| Methods in fern.example that throw FeatureNotSupportedException | |
| --- | --- |
| `static void` | `SBMLMathTreeTest.main(String[] args)`             Dumb the MathTrees of an SBML network to Stdout |

| Uses of FeatureNotSupportedException in fern.network | |
| --- | --- |

| Methods in fern.network that throw FeatureNotSupportedException | |
| --- | --- |
| `static Network` | `NetworkLoader.readNetwork(File file)`             Tries to read the given file and returns the network in it (if there is one) |

| Uses of FeatureNotSupportedException in fern.network.sbml | |
| --- | --- |

| Constructors in fern.network.sbml that throw FeatureNotSupportedException | |
| --- | --- |
| `SBMLNetwork(File file)`             Creates a network from a sbmlfile. |
| `SBMLNetwork(File file, boolean ignoreExceptions)`             Creates a network from a sbml file. |

| Uses of FeatureNotSupportedException in fern.tools | |
| --- | --- |

| Methods in fern.tools that throw FeatureNotSupportedException | |
| --- | --- |
| `static Network` | `NetworkTools.loadNetwork(File file)`             Loads a network from file identifying the type (FernML/SBML). |

---


|  |  |  |  |  |  |  |  |  |  |  |
| --- | --- | --- | --- | --- | --- | --- | --- | --- | --- | --- |
| |  |  |  |  |  |  |  |  | | --- | --- | --- | --- | --- | --- | --- | --- | | **Overview** | **Package** | **Class** | **Use** | **Tree** | **Deprecated** | **Index** | **Help** | | |  |
| PREV   NEXT | **FRAMES**    **NO FRAMES**     **All Classes** |


---
