# Supplementary material for: FERN – a Java framework for stochastic simulation and evaluation of reaction networks
Source: BMC Bioinformatics. 2008 Aug 29;9:356. doi: 10.1186/1471-2105-9-356 (PMC2553347; doi:10.1186/1471-2105-9-356)
Supplement: Additional file 1 — FERN distribution, Version 1.3. This archive contains the FERN source code and binaries as well as documentation and example models in FernML and SBML. [file 1471-2105-9-356-S1.zip › fern/doc/javadoc/fern/network/class-use/Network.html]

Uses of Interface fern.network.Network


---


|  |  |  |  |  |  |  |  |  |  |  |
| --- | --- | --- | --- | --- | --- | --- | --- | --- | --- | --- |
| |  |  |  |  |  |  |  |  | | --- | --- | --- | --- | --- | --- | --- | --- | | **Overview** | **Package** | **Class** | **Use** | **Tree** | **Deprecated** | **Index** | **Help** | | |  |
| PREV   NEXT | **FRAMES**    **NO FRAMES**     **All Classes** |


---


## **Uses of Interface fern.network.Network**

| Packages that use Network | |
| --- | --- |
| **fern.analysis** | Provides classes and algorithms for analysing networks like ShortestPath, AutocatalyticDetection. |
| **fern.benchmark** | Provides classes for benchmarking simulations (time benchmarks, histogram distance calculation). |
| **fern.cellDesigner** |  |
| **fern.cellDesigner.ui** |  |
| **fern.cytoscape** | Provides the classes for the cytoscape plugin. |
| **fern.cytoscape.ui** |  |
| **fern.network** | Provides general classes and interfaces for storing network data. |
| **fern.network.creation** | Provides classes for the evolution of networks. |
| **fern.network.fernml** | Provides the classes for parsing and using FernML based networks. |
| **fern.network.modification** | Provides classes for modifications of networks. |
| **fern.network.sbml** | Provides the classes for parsing and using sbml based networks. |
| **fern.simulation** | Provides algorithms for simulating reaction network as well as methods for observer certain aspects of a simulation. |
| **fern.simulation.algorithm** | Provides algorithms for stochastic simultion of reaction networks. |
| **fern.tools** | Provides common used tools. |

| Uses of Network in fern.analysis | |
| --- | --- |

| Fields in fern.analysis declared as Network | |
| --- | --- |
| `protected  Network` | `AnalysisBase.network`             Contains the network. |
| `protected  Network` | `AnalysisBase.originalNetwork`             Contains the original network if `network` is a `ModifierNetwork`. |

| Methods in fern.analysis with parameters of type Network | |
| --- | --- |
| `boolean` | `NodeCheckerByAnnotation.checkReactionNode(Network network, int reaction)` |
| `boolean` | `NodeChecker.checkReactionNode(Network network, int reaction)` |
| `boolean` | `NodeCheckerByAnnotation.checkSpeciesNode(Network network, int species)` |
| `boolean` | `NodeChecker.checkSpeciesNode(Network network, int species)` |
| `void` | `NetworkSearchAction.initialize(Network net)`             Gets called before anything is inserted into the search structure. |

| Constructors in fern.analysis with parameters of type Network | |
| --- | --- |
| `AnalysisBase(Network network)`             Creates an analysis instance. |
| `AutocatalyticNetworkDetection(Network network)`             Creates the AutocatalyticDetection by using the in the network built in `CatalystIterator`. |
| `AutocatalyticNetworkDetection(Network network, CatalystIterator cataIt)`             Creates the AutocatalyticDetection by using the second argument as `CatalystIterator`. |
| `ShortestPath(Network network)`             Creates the class with the specified network. |

| Uses of Network in fern.benchmark | |
| --- | --- |

| Constructors in fern.benchmark with parameters of type Network | |
| --- | --- |
| `SimulatorCorrectness(Network net, double moment, String... speciesNames)`             Creates the benchmark instance with given network, moment in time and species. |
| `SimulatorFireTypes(Network net, double time)`             Create the benchmark and defines the time each simulator has to run in one iteration. |
| `SimulatorPerformance(Network net)`             Registers the six built-in simulators for the performance benchmarks. |
| `SimulatorRandomNumbers(Network net, double time)`             Create the benchmark and defines the time each simulator has to run in one iteration. |
| `SimulatorTime(Network net, double time)`             Create the benchmark and defines the time each simulator has to run in one iteration. |

| Uses of Network in fern.cellDesigner | |
| --- | --- |

| Classes in fern.cellDesigner that implement Network | |
| --- | --- |
| `class` | `CellDesignerNetworkWrapper` |

| Uses of Network in fern.cellDesigner.ui | |
| --- | --- |

| Methods in fern.cellDesigner.ui with parameters of type Network | |
| --- | --- |
| `void` | `MainFrame.loadNetwork(Network net)` |
| `void` | `OverviewPane.setErrorMessage(Network net, String message)` |
| `void` | `ExtendedPane.setSpecies(Network net)` |

| Uses of Network in fern.cytoscape | |
| --- | --- |

| Classes in fern.cytoscape that implement Network | |
| --- | --- |
| `class` | `CytoscapeNetworkWrapper` |

| Uses of Network in fern.cytoscape.ui | |
| --- | --- |

| Methods in fern.cytoscape.ui with parameters of type Network | |
| --- | --- |
| `void` | `MainFrame.loadNetwork(Network net)` |
| `void` | `OverviewPane.setErrorMessage(Network net, String message)` |
| `void` | `ExtendedPane.setSpecies(Network net)` |

| Uses of Network in fern.network | |
| --- | --- |

| Classes in fern.network that implement Network | |
| --- | --- |
| `class` | `AbstractNetworkImpl`             Base implementation for the `Network` interface. |

| Methods in fern.network that return Network | |
| --- | --- |
| `static Network` | `NetworkLoader.readNetwork(File file)`             Tries to read the given file and returns the network in it (if there is one) |

| Constructors in fern.network with parameters of type Network | |
| --- | --- |
| `AmountManager(Network net)`             Creates an `AmountManager` for a given network |

| Uses of Network in fern.network.creation | |
| --- | --- |

| Classes in fern.network.creation that implement Network | |
| --- | --- |
| `class` | `AutocatalyticNetwork`             Evolve an autocatalytic network. |

| Uses of Network in fern.network.fernml | |
| --- | --- |

| Classes in fern.network.fernml that implement Network | |
| --- | --- |
| `class` | `FernMLNetwork`             A `FernMLNetwork` is usually loaded from a file. |

| Constructors in fern.network.fernml with parameters of type Network | |
| --- | --- |
| `FernMLNetwork(Network net)`             Create a `FernMLNetwork` from an existing `Network`. |
| `FernMLNetwork(Network net, double[] kineticConstants)`             Creates a FernMLNetwork out of an existing network (e.g. to save it to a fernml file) using explicitly given kineticConstants (when net doesn't use `KineticConstantPropensityCalculator` If `kineticConstants` is `null` or to short, a default value of 1 is taken. |

| Uses of Network in fern.network.modification | |
| --- | --- |

| Classes in fern.network.modification that implement Network | |
| --- | --- |
| `class` | `CatalysedNetwork`             Modifies the network by adding reactions X+C -> Y+C (where C is each catalyst of the original reaction). |
| `class` | `ExtractSubNetwork`             Extracts some reactions / species from a given net to form a new network. |
| `class` | `ModifierNetwork`             Base class for modified networks, which implements the full `Network` interface. |
| `class` | `ReversibleNetwork`             Doubles each reaction in a way that each original unidirectional reaction becomes reversible.As proposed by `ModifierNetwork`, the reactions are not copied but the indices are redirected. |

| Methods in fern.network.modification that return Network | |
| --- | --- |
| `Network` | `ModifierNetwork.getOriginalNetwork()`             Gets the original network. |
| `Network` | `ModifierNetwork.getParentNetwork()`             Gets the parent network. |

| Constructors in fern.network.modification with parameters of type Network | |
| --- | --- |
| `CatalysedNetwork(Network originalNet)`             Create a catalyzed network from an original network. |
| `ExtractSubNetwork(Network originalNet, BitVector reactions, BitVector species)`             Extracts a given subnet from a network. |
| `ModifierNetwork(Network originalNet)`             Creates a `ModifierNetwork` from an original network. |
| `ReversibleNetwork(Network originalNet, PropensityCalculator reversiblePropensityCalculator)`             Creates a new network from an original network and virtually creates for each reaction a new inverse reaction. |

| Uses of Network in fern.network.sbml | |
| --- | --- |

| Classes in fern.network.sbml that implement Network | |
| --- | --- |
| `class` | `SBMLNetwork`             For specifications of the sbml format refer to http:\\www.sbml.org. |

| Constructors in fern.network.sbml with parameters of type Network | |
| --- | --- |
| `MathTree(Network net, org.sbml.libsbml.ASTNode ast, Map<String,Double> globals, Map<String,Double> locals, Map<String,Integer> bindings)`             Creates a MathTree from an libsbml `ASTNode`. |
| `SBMLNetwork(Network net)`             Create a `SBMLNetwork` from an existing `Network`. |

| Uses of Network in fern.simulation | |
| --- | --- |

| Methods in fern.simulation that return Network | |
| --- | --- |
| `Network` | `Simulator.getNet()`             Gets the simulation network. |

| Constructors in fern.simulation with parameters of type Network | |
| --- | --- |
| `Simulator(Network net)`             Creates a new simulator for the given network. |

| Uses of Network in fern.simulation.algorithm | |
| --- | --- |

| Constructors in fern.simulation.algorithm with parameters of type Network | |
| --- | --- |
| `AbstractBaseTauLeaping(Network net)`             Create the simulator for a given network. |
| `AbstractTauLeapingPropensityBoundSimulator(Network net)` |
| `DependencyGraph(Network net)`             Creates the dependency graph for a given network |
| `GibsonBruckSimulator(Network net)` |
| `GillespieEnhanced(Network net)` |
| `GillespieSimple(Network net)` |
| `HybridMaximalTimeStep(Network net)` |
| `TauLeapingAbsoluteBoundSimulator(Network net)` |
| `TauLeapingRelativeBoundSimulator(Network net)` |
| `TauLeapingSpeciesPopulationBoundSimulator(Network net)` |

| Uses of Network in fern.tools | |
| --- | --- |

| Methods in fern.tools that return Network | |
| --- | --- |
| `static Network` | `NetworkTools.loadNetwork(File file)`             Loads a network from file identifying the type (FernML/SBML). |

| Methods in fern.tools with parameters of type Network | |
| --- | --- |
| `static boolean` | `NetworkTools.areEqual(Network a, Network b)`             Gets whether or not the two given networks contain the same species, the same reactions and yield the same propensities when the amounts of each reactant are respectively 1, 5, 10, 100. |
| `static void` | `NetworkTools.dumpNetwork(Network net)`             Dumps the network to stdout. |
| `static void` | `NetworkTools.dumpNetwork(Network net, Writer writer)`             Dumps the network to the given `Writer`. |
| `static double` | `NetworkTools.getConstantBySettingReactantsToStoich(Network net, int r)`             Gets the reaction rate constant by setting the reactant amounts to the stoichiometric coefficient. |
| `static String[]` | `NetworkTools.getReactionNames(Network net, int[] reaction)`             Transforms the reaction indices in the given array to the corresponding names. |
| `static String` | `NetworkTools.getReactionNameWithAmounts(Network net, int... reactions)`             Gets a string representation of the given reactions and the amounts of each participating species in parentheses. |
| `static int[]` | `NetworkTools.getReactionsOf(Network net, String[] speciesName)`             Gets the reaction whose products / reactants are in speciesName |
| `static int[]` | `NetworkTools.getSpeciesIndices(Network net, String[] speciesName)`             Transforms the species names in the given array to the corresponding indices. |
| `static String[]` | `NetworkTools.getSpeciesNames(Network net, int[] species)`             Transforms the species indices in the given array to the corresponding names. |
| `static String` | `NetworkTools.getSpeciesNameWithAmount(Network net, int... species)`             Gets the species names together with its actual amount in parentheses |
| `static void` | `NetworkTools.useActualAmountAsInitialAmount(Network net)`             Copies the actual amount of the species in the network to its initial amount. |

---


|  |  |  |  |  |  |  |  |  |  |  |
| --- | --- | --- | --- | --- | --- | --- | --- | --- | --- | --- |
| |  |  |  |  |  |  |  |  | | --- | --- | --- | --- | --- | --- | --- | --- | | **Overview** | **Package** | **Class** | **Use** | **Tree** | **Deprecated** | **Index** | **Help** | | |  |
| PREV   NEXT | **FRAMES**    **NO FRAMES**     **All Classes** |


---
