# Supplementary material for: FERN – a Java framework for stochastic simulation and evaluation of reaction networks
Source: BMC Bioinformatics. 2008 Aug 29;9:356. doi: 10.1186/1471-2105-9-356 (PMC2553347; doi:10.1186/1471-2105-9-356)
Supplement: Additional file 1 — FERN distribution, Version 1.3. This archive contains the FERN source code and binaries as well as documentation and example models in FernML and SBML. [file 1471-2105-9-356-S1.zip › fern/doc/javadoc/fern/network/class-use/PropensityCalculator.html]

Uses of Interface fern.network.PropensityCalculator


---


|  |  |  |  |  |  |  |  |  |  |  |
| --- | --- | --- | --- | --- | --- | --- | --- | --- | --- | --- |
| |  |  |  |  |  |  |  |  | | --- | --- | --- | --- | --- | --- | --- | --- | | **Overview** | **Package** | **Class** | **Use** | **Tree** | **Deprecated** | **Index** | **Help** | | |  |
| PREV   NEXT | **FRAMES**    **NO FRAMES**     **All Classes** |


---


## **Uses of Interface fern.network.PropensityCalculator**

| Packages that use PropensityCalculator | |
| --- | --- |
| **fern.cellDesigner** |  |
| **fern.network** | Provides general classes and interfaces for storing network data. |
| **fern.network.creation** | Provides classes for the evolution of networks. |
| **fern.network.modification** | Provides classes for modifications of networks. |
| **fern.network.sbml** | Provides the classes for parsing and using sbml based networks. |
| **fern.simulation** | Provides algorithms for simulating reaction network as well as methods for observer certain aspects of a simulation. |

| Uses of PropensityCalculator in fern.cellDesigner | |
| --- | --- |

| Classes in fern.cellDesigner that implement PropensityCalculator | |
| --- | --- |
| `class` | `CellDesignerPropensityCalculator`             Propensity calculator which is used for `SBMLNetwork`s. |

| Uses of PropensityCalculator in fern.network | |
| --- | --- |

| Subinterfaces of PropensityCalculator in fern.network | |
| --- | --- |
| `interface` | `ComplexDependenciesPropensityCalculator`             In a SBML network, the propensities of reaction are calculated by using the kineticLaw tag, which contains a MathML expression. |

| Classes in fern.network that implement PropensityCalculator | |
| --- | --- |
| `class` | `AbstractKineticConstantPropensityCalculator`             Base implementation of a `PropensityCalculator`. |
| `class` | `ArrayKineticConstantPropensityCalculator`             Implementation of an `AbstractKineticConstantPropensityCalculator` which uses an array to store the constants for each reaction. |

| Fields in fern.network declared as PropensityCalculator | |
| --- | --- |
| `protected  PropensityCalculator` | `AbstractNetworkImpl.propensitiyCalculator`             Stores the `PropensityCalculator` of the network. |

| Methods in fern.network that return PropensityCalculator | |
| --- | --- |
| `PropensityCalculator` | `Network.getPropensityCalculator()`             Gets the `PropensityCalculator` for this network. |
| `PropensityCalculator` | `AbstractNetworkImpl.getPropensityCalculator()` |

| Uses of PropensityCalculator in fern.network.creation | |
| --- | --- |

| Methods in fern.network.creation that return PropensityCalculator | |
| --- | --- |
| `PropensityCalculator` | `AutocatalyticNetwork.getReversePropensityCalculator()`             Gets the `PropensityCalculator` which has to be used for instantiation of the `ReversibleNetwork`. |

| Uses of PropensityCalculator in fern.network.modification | |
| --- | --- |

| Methods in fern.network.modification that return PropensityCalculator | |
| --- | --- |
| `PropensityCalculator` | `ReversibleNetwork.getPropensityCalculator()`             Gets the `PropensityCalculator` for the modified network. |
| `PropensityCalculator` | `ModifierNetwork.getPropensityCalculator()`             Gets the `PropensityCalculator` of the original network. |
| `PropensityCalculator` | `ExtractSubNetwork.getPropensityCalculator()`             Gets the `PropensityCalculator` for the extracted subnet. |
| `PropensityCalculator` | `CatalysedNetwork.getPropensityCalculator()` |

| Constructors in fern.network.modification with parameters of type PropensityCalculator | |
| --- | --- |
| `ReversibleNetwork(Network originalNet, PropensityCalculator reversiblePropensityCalculator)`             Creates a new network from an original network and virtually creates for each reaction a new inverse reaction. |

| Uses of PropensityCalculator in fern.network.sbml | |
| --- | --- |

| Classes in fern.network.sbml that implement PropensityCalculator | |
| --- | --- |
| `class` | `SBMLPropensityCalculator`             Propensity calculator which is used for `SBMLNetwork`s. |

| Uses of PropensityCalculator in fern.simulation | |
| --- | --- |

| Methods in fern.simulation that return PropensityCalculator | |
| --- | --- |
| `PropensityCalculator` | `Simulator.getPropensityCalculator()`             Gets the `PropensityCalculator`. |

---


|  |  |  |  |  |  |  |  |  |  |  |
| --- | --- | --- | --- | --- | --- | --- | --- | --- | --- | --- |
| |  |  |  |  |  |  |  |  | | --- | --- | --- | --- | --- | --- | --- | --- | | **Overview** | **Package** | **Class** | **Use** | **Tree** | **Deprecated** | **Index** | **Help** | | |  |
| PREV   NEXT | **FRAMES**    **NO FRAMES**     **All Classes** |


---
