# Supplementary material for: FERN – a Java framework for stochastic simulation and evaluation of reaction networks
Source: BMC Bioinformatics. 2008 Aug 29;9:356. doi: 10.1186/1471-2105-9-356 (PMC2553347; doi:10.1186/1471-2105-9-356)
Supplement: Additional file 1 — FERN distribution, Version 1.3. This archive contains the FERN source code and binaries as well as documentation and example models in FernML and SBML. [file 1471-2105-9-356-S1.zip › fern/doc/javadoc/fern/network/ComplexDependenciesPropensityCalculator.html]

ComplexDependenciesPropensityCalculator


---


|  |  |  |  |  |  |  |  |  |  |  |
| --- | --- | --- | --- | --- | --- | --- | --- | --- | --- | --- |
| |  |  |  |  |  |  |  |  | | --- | --- | --- | --- | --- | --- | --- | --- | | **Overview** | **Package** | **Class** | **Use** | **Tree** | **Deprecated** | **Index** | **Help** | | |  |
| **PREV CLASS**   **NEXT CLASS** | **FRAMES**    **NO FRAMES**     **All Classes** |
| SUMMARY: NESTED | FIELD | CONSTR | METHOD | DETAIL: FIELD | CONSTR | METHOD |


---


## fern.network Interface ComplexDependenciesPropensityCalculator

**All Superinterfaces:**: PropensityCalculator

**All Known Implementing Classes:**: CellDesignerPropensityCalculator, SBMLPropensityCalculator

---

``` public interface ComplexDependenciesPropensityCalculator extends PropensityCalculator ```

In a SBML network, the propensities of reaction are calculated by using the
kineticLaw tag, which contains a MathML expression. Since therein arbitrary species can
be included, there must be a way to fetch dependencies between the reactions in order to
create the `DependencyGraph`.

**Author:**
:   Florian Erhard

---

| **Method Summary** | |
| --- | --- |
| `List<Integer>` | `getKineticLawSpecies(int reaction)`             Gets the indices of the species that are included in the calculation of the given reaction. |

| **Methods inherited from interface fern.network.PropensityCalculator** |
| --- |
| `calculatePropensity` |

| **Method Detail** |
| --- |

### getKineticLawSpecies

```
List<Integer> getKineticLawSpecies(int reaction)
```

:   Gets the indices of the species that are included in the calculation of
    the given reaction.

    :   **Parameters:**: `reaction` - index of the reaction **Returns:**: indices of the species included in the reaction's kinetic law


---


|  |  |  |  |  |  |  |  |  |  |  |
| --- | --- | --- | --- | --- | --- | --- | --- | --- | --- | --- |
| |  |  |  |  |  |  |  |  | | --- | --- | --- | --- | --- | --- | --- | --- | | **Overview** | **Package** | **Class** | **Use** | **Tree** | **Deprecated** | **Index** | **Help** | | |  |
| **PREV CLASS**   **NEXT CLASS** | **FRAMES**    **NO FRAMES**     **All Classes** |
| SUMMARY: NESTED | FIELD | CONSTR | METHOD | DETAIL: FIELD | CONSTR | METHOD |


---
