# Supplementary material for: FERN – a Java framework for stochastic simulation and evaluation of reaction networks
Source: BMC Bioinformatics. 2008 Aug 29;9:356. doi: 10.1186/1471-2105-9-356 (PMC2553347; doi:10.1186/1471-2105-9-356)
Supplement: Additional file 1 — FERN distribution, Version 1.3. This archive contains the FERN source code and binaries as well as documentation and example models in FernML and SBML. [file 1471-2105-9-356-S1.zip › fern/doc/javadoc/fern/network/creation/AutocatalyticNetwork.html]

AutocatalyticNetwork


---


|  |  |  |  |  |  |  |  |  |  |  |
| --- | --- | --- | --- | --- | --- | --- | --- | --- | --- | --- |
| |  |  |  |  |  |  |  |  | | --- | --- | --- | --- | --- | --- | --- | --- | | **Overview** | **Package** | **Class** | **Use** | **Tree** | **Deprecated** | **Index** | **Help** | | |  |
| PREV CLASS   **NEXT CLASS** | **FRAMES**    **NO FRAMES**     **All Classes** |
| SUMMARY: NESTED | FIELD | CONSTR | METHOD | DETAIL: FIELD | CONSTR | METHOD |


---


## fern.network.creation Class AutocatalyticNetwork

```
java.lang.Object
  fern.network.AbstractNetworkImpl
      fern.network.creation.AutocatalyticNetwork
```

**All Implemented Interfaces:**: CatalystIterator, Network

---

``` public class AutocatalyticNetwork extends AbstractNetworkImpl implements CatalystIterator ```

Evolve an autocatalytic network. The evolution starts at some monomers, let them aggregate
(e.g. A+B -> AB) by a given probability and up to a given length. Then, each reaction
is catalyzed by a given probability by some molecule species (the catalysts are stored
as fields in the net's annotation). Since the reactions are only unidirectional you have
to create a `ReversibleNetwork` out of it. Because of this, also catalysts for
the reverse reactions are stored in the corresponding fields.

The advantage of the unidirectional reactions is space efficiency since the `ReversibleNetwork`
does not copy the reactions but redirects the indices.

This network can of course be used for stochastic simulations. If it is just converted
into a `ReversibleNetwork`, there are just different kinetic constants used for
catalyzed and not catalyzed reactions (`getCatalyzedKineticConstant()`
and `getUncatalyzedKineticConstant()`).

**Author:**
:   Florian Erhard

---

| **Field Summary** | |
| --- | --- |
| `static String` | `CATALYSTS_FIELD`             Name of the field where catalysts are stored. |
| `static String` | `CATALYSTS_FIELD_REVERSIBLE`             Name of the field where catalysts for the reverse reactions are stored. |

| **Fields inherited from class fern.network.AbstractNetworkImpl** |
| --- |
| `adjListPro, adjListRea, amountManager, annotationManager, indexToSpeciesId, name, propensitiyCalculator, speciesIdToIndex` |


| **Constructor Summary** | |
| --- | --- |
| `AutocatalyticNetwork(char[] monomers, Probability createProb, Probability catProb, int maxLength)`             Creates the autocatalytic network from given monomers, reaction probability, catalysis probability up to a given polymer length. |
| `AutocatalyticNetwork(char[] monomers, Probability createProb, Probability catProb, int maxLength, boolean useFastMethod)`             Creates the autocatalytic network from given monomers, reaction probability, catalysis probability up to a given polymer length. useDefault should usually set to true unless you want to evolve really huge networks. |


| **Method Summary** | |
| --- | --- |
| `protected  void` | `createAdjacencyLists()`             Creates the adjacency lists for this network. |
| `protected  void` | `createAmountManager()`             Creates the `AmountManager` for this network. |
| `protected  void` | `createAnnotationManager()`             Creates the `AnnotationManager` for this network. |
| `protected  void` | `createPropensityCalulator()`             Creates the `PropensityCalculator` for this network. |
| `protected  void` | `createSpeciesMapping()`             Reminds extending class to fill `AbstractNetworkImpl.speciesIdToIndex` and `AbstractNetworkImpl.indexToSpeciesId`. |
| `Iterable<Integer>` | `getCatalysts(int reaction)`             Implementation for the `CatalystIterator`. |
| `double` | `getCatalyzedKineticConstant()`             Gets the kinetic constant for catalyzed reactions. |
| `long` | `getInitialAmount(int species)`             Gets the initial amount of the specified molecule species. |
| `long` | `getMonomerAmount()`             Gets the initial amount of the monomers for a simulation algorithm. |
| `int` | `getNumMonomers()`             Gets if the number of monomers |
| `long` | `getOtherAmount()`             Gets the initial amount of the not-monomers for a simulation algorithm. |
| `PropensityCalculator` | `getReversePropensityCalculator()`             Gets the `PropensityCalculator` which has to be used for instantiation of the `ReversibleNetwork`. |
| `double` | `getUncatalyzedKineticConstant()`             Gets the kinetic constant for not catalyzed reactions. |
| `void` | `setCatalyzedKineticConstant(double catalyzedKineticConstant)`             Sets the kinetic constant for catalyzed reactions. |
| `void` | `setInitialAmount(int species, long value)`             Sets the initial amount of the specified molecule species. |
| `void` | `setMonomerAmount(long monomerAmount)`             Sets the initial amount of the monomers for a simulation algorithm. |
| `void` | `setOtherAmount(long otherAmount)`             Sets the initial amount of the not-monomers for a simulation algorithm. |
| `void` | `setUncatalyzedKineticConstant(double uncatalyzedKineticConstant)`             Sets the kinetic constant for not catalyzed reactions. |

| **Methods inherited from class fern.network.AbstractNetworkImpl** |
| --- |
| `getAmountManager, getAnnotationManager, getName, getNumReactions, getNumSpecies, getProducts, getPropensityCalculator, getReactants, getReactionName, getSpeciesByName, getSpeciesMapping, getSpeciesName` |

| **Methods inherited from class java.lang.Object** |
| --- |
| `clone, equals, finalize, getClass, hashCode, notify, notifyAll, toString, wait, wait, wait` |

| **Field Detail** |
| --- |

### CATALYSTS\_FIELD

```
public static final String CATALYSTS_FIELD
```

:   Name of the field where catalysts are stored.

    **See Also:**: Constant Field Values

---


### CATALYSTS\_FIELD\_REVERSIBLE

```
public static final String CATALYSTS_FIELD_REVERSIBLE
```

:   Name of the field where catalysts for the reverse reactions are stored.

    **See Also:**: Constant Field Values


| **Constructor Detail** |
| --- |

### AutocatalyticNetwork

```
public AutocatalyticNetwork(char[] monomers,
                            Probability createProb,
                            Probability catProb,
                            int maxLength)
```

:   Creates the autocatalytic network from given monomers, reaction probability, catalysis probability up to a
    given polymer length. By default, the fast (but memory consuming) method of creating / catalyzing is beeing used.

    **Parameters:**: `monomers` - the monomers to start the network evolution with: `createProb` - the reaction probability: `catProb` - the catalyzation probability: `maxLength` - the maximal polymer length **See Also:**: `Probability`

---


### AutocatalyticNetwork

```
public AutocatalyticNetwork(char[] monomers,
                            Probability createProb,
                            Probability catProb,
                            int maxLength,
                            boolean useFastMethod)
```

:   Creates the autocatalytic network from given monomers, reaction probability, catalysis probability up to a
    given polymer length. useDefault should usually set to true unless you want to evolve really huge networks. The
    slower method only needs O(log(V)) extra space where the faster method needs O(V).

    **Parameters:**: `monomers` - the monomers to start the network evolution with: `createProb` - the reaction probability: `catProb` - the catalysis probability: `maxLength` - the maximal polymer length: `useFastMethod` - what method is going to be used for creating / catalyzing **See Also:**: `Probability`


| **Method Detail** |
| --- |

### createAdjacencyLists

```
protected void createAdjacencyLists()
```

:   Creates the adjacency lists for this network.

    :   **Specified by:**: `createAdjacencyLists` in class `AbstractNetworkImpl`

---


### createAmountManager

```
protected void createAmountManager()
```

:   Creates the `AmountManager` for this network.

    :   **Specified by:**: `createAmountManager` in class `AbstractNetworkImpl`

---


### createAnnotationManager

```
protected void createAnnotationManager()
```

:   Creates the `AnnotationManager` for this network.

    :   **Specified by:**: `createAnnotationManager` in class `AbstractNetworkImpl`

---


### createPropensityCalulator

```
protected void createPropensityCalulator()
```

:   Creates the `PropensityCalculator` for this network.

    :   **Specified by:**: `createPropensityCalulator` in class `AbstractNetworkImpl`

---


### getReversePropensityCalculator

```
public PropensityCalculator getReversePropensityCalculator()
```

:   Gets the `PropensityCalculator` which has to be used for instantiation
    of the `ReversibleNetwork`.

    :   **Returns:**: the `PropensityCalculator` for the `ReversibleNetwork`

---


### createSpeciesMapping

```
protected void createSpeciesMapping()
```

:   **Description copied from class: `AbstractNetworkImpl`**
:   Reminds extending class to fill `AbstractNetworkImpl.speciesIdToIndex` and `AbstractNetworkImpl.indexToSpeciesId`.

    :   **Specified by:**: `createSpeciesMapping` in class `AbstractNetworkImpl`

---


### getCatalysts

```
public Iterable<Integer> getCatalysts(int reaction)
```

:   Implementation for the `CatalystIterator`. Returns the indices of
    catalysts for the given reaction. By using `getAnnotationManager`
    it returns the correct catalysts even if a `ReversibleNetwork` is used.

    :   **Specified by:**: `getCatalysts` in interface `CatalystIterator`
    :   **Parameters:**: `reaction` - index of the reaction for which the catalysts have to be returned **Returns:**: the catalysts of the reaction

---


### getInitialAmount

```
public long getInitialAmount(int species)
```

:   **Description copied from interface: `Network`**
:   Gets the initial amount of the specified molecule species.

    :   **Specified by:**: `getInitialAmount` in interface `Network`
    :   **Parameters:**: `species` - index of the species **Returns:**: initial amount of the species

---


### setInitialAmount

```
public void setInitialAmount(int species,
                             long value)
```

:   **Description copied from interface: `Network`**
:   Sets the initial amount of the specified molecule species.

    :   **Specified by:**: `setInitialAmount` in interface `Network`
    :   **Parameters:**: `species` - index of the species: `value` - initial amount of the species

---


### getNumMonomers

```
public int getNumMonomers()
```

:   Gets if the number of monomers

    :   **Returns:**: number of monomers

---


### getMonomerAmount

```
public long getMonomerAmount()
```

:   Gets the initial amount of the monomers for a simulation algorithm.
    The default is 1000.

    :   **Returns:**: initial amount of the monomers

---


### setMonomerAmount

```
public void setMonomerAmount(long monomerAmount)
```

:   Sets the initial amount of the monomers for a simulation algorithm.
    The default is 1000.

    :   **Parameters:**: `monomerAmount` - initial amount of the monomers

---


### getOtherAmount

```
public long getOtherAmount()
```

:   Gets the initial amount of the not-monomers for a simulation algorithm.
    The default is 1.

    :   **Returns:**: initial amount of the not-monomers

---


### setOtherAmount

```
public void setOtherAmount(long otherAmount)
```

:   Sets the initial amount of the not-monomers for a simulation algorithm.
    The default is 1.

    :   **Parameters:**: `otherAmount` - initial amount of the not-monomers

---


### getCatalyzedKineticConstant

```
public double getCatalyzedKineticConstant()
```

:   Gets the kinetic constant for catalyzed reactions.
    The default is 1.

    :   **Returns:**: the catalyzedKineticConstant

---


### setCatalyzedKineticConstant

```
public void setCatalyzedKineticConstant(double catalyzedKineticConstant)
```

:   Sets the kinetic constant for catalyzed reactions.
    The default is 1.

    :   **Parameters:**: `catalyzedKineticConstant` - the catalyzedKineticConstant to set

---


### getUncatalyzedKineticConstant

```
public double getUncatalyzedKineticConstant()
```

:   Gets the kinetic constant for not catalyzed reactions.
    The default is 0.001.

    :   **Returns:**: the uncatalyzedKineticConstant

---


### setUncatalyzedKineticConstant

```
public void setUncatalyzedKineticConstant(double uncatalyzedKineticConstant)
```

:   Sets the kinetic constant for not catalyzed reactions.
    The default is 0.001.

    :   **Parameters:**: `uncatalyzedKineticConstant` - the uncatalyzedKineticConstant to set


---


|  |  |  |  |  |  |  |  |  |  |  |
| --- | --- | --- | --- | --- | --- | --- | --- | --- | --- | --- |
| |  |  |  |  |  |  |  |  | | --- | --- | --- | --- | --- | --- | --- | --- | | **Overview** | **Package** | **Class** | **Use** | **Tree** | **Deprecated** | **Index** | **Help** | | |  |
| PREV CLASS   **NEXT CLASS** | **FRAMES**    **NO FRAMES**     **All Classes** |
| SUMMARY: NESTED | FIELD | CONSTR | METHOD | DETAIL: FIELD | CONSTR | METHOD |


---
