# Supplementary material for: FERN – a Java framework for stochastic simulation and evaluation of reaction networks
Source: BMC Bioinformatics. 2008 Aug 29;9:356. doi: 10.1186/1471-2105-9-356 (PMC2553347; doi:10.1186/1471-2105-9-356)
Supplement: Additional file 1 — FERN distribution, Version 1.3. This archive contains the FERN source code and binaries as well as documentation and example models in FernML and SBML. [file 1471-2105-9-356-S1.zip › fern/doc/javadoc/fern/network/creation/CatalystIterator.html]

CatalystIterator


---


|  |  |  |  |  |  |  |  |  |  |  |
| --- | --- | --- | --- | --- | --- | --- | --- | --- | --- | --- |
| |  |  |  |  |  |  |  |  | | --- | --- | --- | --- | --- | --- | --- | --- | | **Overview** | **Package** | **Class** | **Use** | **Tree** | **Deprecated** | **Index** | **Help** | | |  |
| **PREV CLASS**   NEXT CLASS | **FRAMES**    **NO FRAMES**     **All Classes** |
| SUMMARY: NESTED | FIELD | CONSTR | METHOD | DETAIL: FIELD | CONSTR | METHOD |


---


## fern.network.creation Interface CatalystIterator

**All Known Implementing Classes:**: AutocatalyticNetwork

---

``` public interface CatalystIterator ```

A `CatalystIterator` is used to enumerate the catalysts of a reaction in
an `AutocatalyticNetwork`. For instance the `AutocatalyticNetworkDetection`
uses it to be able to walk from reactions to their catalysts in its breadth first searches.

**Author:**
:   Florian Erhard

---

| **Method Summary** | |
| --- | --- |
| `Iterable<Integer>` | `getCatalysts(int reaction)`             Gets the indices of the catalysts of a reaction. |

| **Method Detail** |
| --- |

### getCatalysts

```
Iterable<Integer> getCatalysts(int reaction)
```

:   Gets the indices of the catalysts of a reaction.

    :   **Parameters:**: `reaction` - the index of the reaction **Returns:**: catalysts of the reaction


---


|  |  |  |  |  |  |  |  |  |  |  |
| --- | --- | --- | --- | --- | --- | --- | --- | --- | --- | --- |
| |  |  |  |  |  |  |  |  | | --- | --- | --- | --- | --- | --- | --- | --- | | **Overview** | **Package** | **Class** | **Use** | **Tree** | **Deprecated** | **Index** | **Help** | | |  |
| **PREV CLASS**   NEXT CLASS | **FRAMES**    **NO FRAMES**     **All Classes** |
| SUMMARY: NESTED | FIELD | CONSTR | METHOD | DETAIL: FIELD | CONSTR | METHOD |


---
