# Supplementary material for: FERN – a Java framework for stochastic simulation and evaluation of reaction networks
Source: BMC Bioinformatics. 2008 Aug 29;9:356. doi: 10.1186/1471-2105-9-356 (PMC2553347; doi:10.1186/1471-2105-9-356)
Supplement: Additional file 1 — FERN distribution, Version 1.3. This archive contains the FERN source code and binaries as well as documentation and example models in FernML and SBML. [file 1471-2105-9-356-S1.zip › fern/doc/javadoc/fern/network/creation/package-use.html]

Uses of Package fern.network.creation


---


|  |  |  |  |  |  |  |  |  |  |  |
| --- | --- | --- | --- | --- | --- | --- | --- | --- | --- | --- |
| |  |  |  |  |  |  |  |  | | --- | --- | --- | --- | --- | --- | --- | --- | | **Overview** | **Package** | Class | **Use** | **Tree** | **Deprecated** | **Index** | **Help** | | |  |
| PREV   NEXT | **FRAMES**    **NO FRAMES**     **All Classes** |


---


## **Uses of Package fern.network.creation**

| Packages that use fern.network.creation | |
| --- | --- |
| **fern.analysis** | Provides classes and algorithms for analysing networks like ShortestPath, AutocatalyticDetection. |
| **fern.network.creation** | Provides classes for the evolution of networks. |

| Classes in fern.network.creation used by fern.analysis | |
| --- | --- |
| ****CatalystIterator****             A `CatalystIterator` is used to enumerate the catalysts of a reaction in an `AutocatalyticNetwork`. |

| Classes in fern.network.creation used by fern.network.creation | |
| --- | --- |
| ****CatalystIterator****             A `CatalystIterator` is used to enumerate the catalysts of a reaction in an `AutocatalyticNetwork`. |

---


|  |  |  |  |  |  |  |  |  |  |  |
| --- | --- | --- | --- | --- | --- | --- | --- | --- | --- | --- |
| |  |  |  |  |  |  |  |  | | --- | --- | --- | --- | --- | --- | --- | --- | | **Overview** | **Package** | Class | **Use** | **Tree** | **Deprecated** | **Index** | **Help** | | |  |
| PREV   NEXT | **FRAMES**    **NO FRAMES**     **All Classes** |


---
