# Supplementary material for: FERN – a Java framework for stochastic simulation and evaluation of reaction networks
Source: BMC Bioinformatics. 2008 Aug 29;9:356. doi: 10.1186/1471-2105-9-356 (PMC2553347; doi:10.1186/1471-2105-9-356)
Supplement: Additional file 1 — FERN distribution, Version 1.3. This archive contains the FERN source code and binaries as well as documentation and example models in FernML and SBML. [file 1471-2105-9-356-S1.zip › fern/doc/javadoc/fern/network/fernml/FernMLNetwork.html]

FernMLNetwork


---


|  |  |  |  |  |  |  |  |  |  |  |
| --- | --- | --- | --- | --- | --- | --- | --- | --- | --- | --- |
| |  |  |  |  |  |  |  |  | | --- | --- | --- | --- | --- | --- | --- | --- | | **Overview** | **Package** | **Class** | **Use** | **Tree** | **Deprecated** | **Index** | **Help** | | |  |
| **PREV CLASS**   NEXT CLASS | **FRAMES**    **NO FRAMES**     **All Classes** |
| SUMMARY: NESTED | FIELD | CONSTR | METHOD | DETAIL: FIELD | CONSTR | METHOD |


---


## fern.network.fernml Class FernMLNetwork

```
java.lang.Object
  fern.network.AbstractNetworkImpl
      fern.network.fernml.FernMLNetwork
```

**All Implemented Interfaces:**: Network

---

``` public class FernMLNetwork extends AbstractNetworkImpl ```

A `FernMLNetwork` is usually loaded from a file. For specifications see
the included FernMLSchema.xsd or the examples. Additionally, a `FernMLNetwork`
can be created out of an arbitrary `Network`. By using the `saveToFile`
method, every `Network` can be saved as a fernml-File.

**Author:**
:   Florian Erhard

---

| **Field Summary** | |
| --- | --- |

| **Fields inherited from class fern.network.AbstractNetworkImpl** |
| --- |
| `adjListPro, adjListRea, amountManager, annotationManager, indexToSpeciesId, name, propensitiyCalculator, speciesIdToIndex` |


| **Constructor Summary** | |
| --- | --- |
| `FernMLNetwork(File file)`             Creates a `FernMLNetwork` from a file. |
| `FernMLNetwork(Network net)`             Create a `FernMLNetwork` from an existing `Network`. |
| `FernMLNetwork(Network net, double[] kineticConstants)`             Creates a FernMLNetwork out of an existing network (e.g. to save it to a fernml file) using explicitly given kineticConstants (when net doesn't use `KineticConstantPropensityCalculator` If `kineticConstants` is `null` or to short, a default value of 1 is taken. |


| **Method Summary** | |
| --- | --- |
| `protected  void` | `createAdjacencyLists()`             Creates the adjacency lists by parsing the jdom tree. |
| `protected  void` | `createAmountManager()`             Reminds extending class to fill `AbstractNetworkImpl.amountManager`. |
| `protected  void` | `createAnnotationManager()`             Creates the `AnnotationManager` as a `FernMLAnnotationManager`. |
| `protected  void` | `createPropensityCalulator()`             Does nothing, the `PropensityCalculator` is created in `createAdjacencyLists` because the reactions constants are already parsed there. |
| `protected  void` | `createSpeciesMapping()`             Creates the species mapping by parsing the jdom tree. |
| `long` | `getInitialAmount(int species)`             Gets the initial amount of the specified molecule species. |
| `int` | `getNumReactions()`             Gets the number of reaction within the network. |
| `int` | `getNumSpecies()`             Gets the number of species within the network. |
| `void` | `saveToFile(File file)`             Saves the actual `FernMLNetwork` to a file. |
| `void` | `setInitialAmount(int species, long value)`             Sets the initial amount of the specified molecule species. |

| **Methods inherited from class fern.network.AbstractNetworkImpl** |
| --- |
| `getAmountManager, getAnnotationManager, getName, getProducts, getPropensityCalculator, getReactants, getReactionName, getSpeciesByName, getSpeciesMapping, getSpeciesName` |

| **Methods inherited from class java.lang.Object** |
| --- |
| `clone, equals, finalize, getClass, hashCode, notify, notifyAll, toString, wait, wait, wait` |

| **Constructor Detail** |
| --- |

### FernMLNetwork

```
public FernMLNetwork(File file)
              throws IOException,
                     JDOMException
```

:   Creates a `FernMLNetwork` from a file.

    **Parameters:**: `file` - file containing the network **Throws:**: `IOException` - if the file cannot be read: `JDOMException` - if the file is malformed

---


### FernMLNetwork

```
public FernMLNetwork(Network net)
```

:   Create a `FernMLNetwork` from an existing `Network`. If the
    network's `PropensityCalculator` is not an `AbstractKineticConstantPropensityCalculator`,
    the constant for the rate reaction is obtained by the propensity calculator by setting
    each reactant species' amount to 1. If the stoichiometry of some reactant is greater than 1 the value
    is set accordingly.

    **Parameters:**: `net` - the network to create a `FernMLNetwork` from

---


### FernMLNetwork

```
public FernMLNetwork(Network net,
                     double[] kineticConstants)
```

:   Creates a FernMLNetwork out of an existing network (e.g. to save it to a fernml file)
    using explicitly given kineticConstants (when net doesn't use `KineticConstantPropensityCalculator`
    If `kineticConstants` is `null` or to short, a default value of 1 is taken.

    **Parameters:**: `net` - An existing network: `kineticConstants` - kinetic constants for each reaction in `net`


| **Method Detail** |
| --- |

### getNumReactions

```
public int getNumReactions()
```

:   **Description copied from interface: `Network`**
:   Gets the number of reaction within the network.

    :   **Specified by:**: `getNumReactions` in interface `Network` **Overrides:**: `getNumReactions` in class `AbstractNetworkImpl`
    :   **Returns:**: number of reactions

---


### getNumSpecies

```
public int getNumSpecies()
```

:   **Description copied from interface: `Network`**
:   Gets the number of species within the network.

    :   **Specified by:**: `getNumSpecies` in interface `Network` **Overrides:**: `getNumSpecies` in class `AbstractNetworkImpl`
    :   **Returns:**: number of species

---


### setInitialAmount

```
public void setInitialAmount(int species,
                             long value)
```

:   **Description copied from interface: `Network`**
:   Sets the initial amount of the specified molecule species.

    :   **Parameters:**: `species` - index of the species: `value` - initial amount of the species

---


### getInitialAmount

```
public long getInitialAmount(int species)
```

:   **Description copied from interface: `Network`**
:   Gets the initial amount of the specified molecule species.

    :   **Parameters:**: `species` - index of the species **Returns:**: initial amount of the species

---


### createAmountManager

```
protected void createAmountManager()
```

:   **Description copied from class: `AbstractNetworkImpl`**
:   Reminds extending class to fill `AbstractNetworkImpl.amountManager`.

    :   **Specified by:**: `createAmountManager` in class `AbstractNetworkImpl`

---


### createAdjacencyLists

```
protected void createAdjacencyLists()
```

:   Creates the adjacency lists by parsing the jdom tree.

    :   **Specified by:**: `createAdjacencyLists` in class `AbstractNetworkImpl`

---


### createPropensityCalulator

```
protected void createPropensityCalulator()
```

:   Does nothing, the `PropensityCalculator` is created in `createAdjacencyLists`
    because the reactions constants are already parsed there.

    :   **Specified by:**: `createPropensityCalulator` in class `AbstractNetworkImpl`

---


### createAnnotationManager

```
protected void createAnnotationManager()
```

:   Creates the `AnnotationManager` as a `FernMLAnnotationManager`.

    :   **Specified by:**: `createAnnotationManager` in class `AbstractNetworkImpl`

---


### createSpeciesMapping

```
protected void createSpeciesMapping()
```

:   Creates the species mapping by parsing the jdom tree.

    :   **Specified by:**: `createSpeciesMapping` in class `AbstractNetworkImpl`

---


### saveToFile

```
public void saveToFile(File file)
                throws IOException
```

:   Saves the actual `FernMLNetwork` to a file.

    :   **Parameters:**: `file` - the file to save the network in **Throws:**: `IOException` - if the file cannot be written


---


|  |  |  |  |  |  |  |  |  |  |  |
| --- | --- | --- | --- | --- | --- | --- | --- | --- | --- | --- |
| |  |  |  |  |  |  |  |  | | --- | --- | --- | --- | --- | --- | --- | --- | | **Overview** | **Package** | **Class** | **Use** | **Tree** | **Deprecated** | **Index** | **Help** | | |  |
| **PREV CLASS**   NEXT CLASS | **FRAMES**    **NO FRAMES**     **All Classes** |
| SUMMARY: NESTED | FIELD | CONSTR | METHOD | DETAIL: FIELD | CONSTR | METHOD |


---
