# Supplementary material for: FERN – a Java framework for stochastic simulation and evaluation of reaction networks
Source: BMC Bioinformatics. 2008 Aug 29;9:356. doi: 10.1186/1471-2105-9-356 (PMC2553347; doi:10.1186/1471-2105-9-356)
Supplement: Additional file 1 — FERN distribution, Version 1.3. This archive contains the FERN source code and binaries as well as documentation and example models in FernML and SBML. [file 1471-2105-9-356-S1.zip › fern/doc/javadoc/fern/network/modification/CatalysedNetwork.html]

CatalysedNetwork


---


|  |  |  |  |  |  |  |  |  |  |  |
| --- | --- | --- | --- | --- | --- | --- | --- | --- | --- | --- |
| |  |  |  |  |  |  |  |  | | --- | --- | --- | --- | --- | --- | --- | --- | | **Overview** | **Package** | **Class** | **Use** | **Tree** | **Deprecated** | **Index** | **Help** | | |  |
| PREV CLASS   **NEXT CLASS** | **FRAMES**    **NO FRAMES**     **All Classes** |
| SUMMARY: NESTED | FIELD | CONSTR | METHOD | DETAIL: FIELD | CONSTR | METHOD |


---


## fern.network.modification Class CatalysedNetwork

```
java.lang.Object
  fern.network.modification.ModifierNetwork
      fern.network.modification.CatalysedNetwork
```

**All Implemented Interfaces:**: Network

---

``` public class CatalysedNetwork extends ModifierNetwork ```

Modifies the network by adding reactions X+C -> Y+C (where C is each catalyst of the
original reaction). If a reaction has n catalysts, there will be n+1 reactions
generated. It is only possible to create a `CatalysedNetwork` out of a
`AutocatalyticNetwork` (or at least of a `ModifierNetwork` whose original network
is a `AutocatalyticNetwork`).

The `AmountManager` automatically monitors the food molecules amounts and whenever
it changes, it is reset to the initial value (given by `AutocatalyticNetwork.getMonomerAmount()`.

The `PropensityCalculator`'s constant is `AutocatalyticNetwork.getCatalyzedKineticConstant()`
for each reaction with a catalyst and `AutocatalyticNetwork.getUncatalyzedKineticConstant()` for
the other ones.

The `AnnotationManager` uses the underlying one but removes the `AutocatalyticNetwork.CATALYSTS_FIELD`
and the field `Autocatalytic` from not catalyzed reactions.

**Author:**
:   Florian Erhard

---

| **Constructor Summary** | |
| --- | --- |
| `CatalysedNetwork(Network originalNet)`             Create a catalyzed network from an original network. |


| **Method Summary** | |
| --- | --- |
| `AmountManager` | `getAmountManager()`             Gets the `AmountManager` of the original network. |
| `AnnotationManager` | `getAnnotationManager()`             Gets the `AnnotationManager` of the original network |
| `int` | `getNumReactions()`             Gets the number of reaction in the original network. |
| `int[]` | `getProducts(int reaction)`             Gets the products of a reaction in the original network. |
| `PropensityCalculator` | `getPropensityCalculator()`             Gets the `PropensityCalculator` of the original network. |
| `int[]` | `getReactants(int reaction)`             Gets the reactants of a reaction in the original network. |
| `String` | `getReactionName(int index)`             Gets a string representation of the reactio in the original network. |

| **Methods inherited from class fern.network.modification.ModifierNetwork** |
| --- |
| `getInitialAmount, getName, getNumSpecies, getOriginalNetwork, getParentNetwork, getSpeciesByName, getSpeciesName, setInitialAmount` |

| **Methods inherited from class java.lang.Object** |
| --- |
| `clone, equals, finalize, getClass, hashCode, notify, notifyAll, toString, wait, wait, wait` |

| **Constructor Detail** |
| --- |

### CatalysedNetwork

```
public CatalysedNetwork(Network originalNet)
```

:   Create a catalyzed network from an original network.

    **Parameters:**: `originalNet` - the original network


| **Method Detail** |
| --- |

### getNumReactions

```
public int getNumReactions()
```

:   **Description copied from class: `ModifierNetwork`**
:   Gets the number of reaction in the original network.

    :   **Specified by:**: `getNumReactions` in interface `Network` **Overrides:**: `getNumReactions` in class `ModifierNetwork`
    :   **Returns:**: number of reactions in the original network

---


### getReactants

```
public int[] getReactants(int reaction)
```

:   **Description copied from class: `ModifierNetwork`**
:   Gets the reactants of a reaction in the original network.

    :   **Specified by:**: `getReactants` in interface `Network` **Overrides:**: `getReactants` in class `ModifierNetwork`
    :   **Parameters:**: `reaction` - index of the reaction in the original network **Returns:**: indices of the reactants in the original network

---


### getProducts

```
public int[] getProducts(int reaction)
```

:   **Description copied from class: `ModifierNetwork`**
:   Gets the products of a reaction in the original network.

    :   **Specified by:**: `getProducts` in interface `Network` **Overrides:**: `getProducts` in class `ModifierNetwork`
    :   **Parameters:**: `reaction` - index of the reaction in the original network **Returns:**: indices of the products in the original network

---


### getPropensityCalculator

```
public PropensityCalculator getPropensityCalculator()
```

:   **Description copied from class: `ModifierNetwork`**
:   Gets the `PropensityCalculator` of the original network.

    :   **Specified by:**: `getPropensityCalculator` in interface `Network` **Overrides:**: `getPropensityCalculator` in class `ModifierNetwork`
    :   **Returns:**: `PropensityCalculator` of the the original network

---


### getAmountManager

```
public AmountManager getAmountManager()
```

:   **Description copied from class: `ModifierNetwork`**
:   Gets the `AmountManager` of the original network.

    :   **Specified by:**: `getAmountManager` in interface `Network` **Overrides:**: `getAmountManager` in class `ModifierNetwork`
    :   **Returns:**: `AmountManager` of the the original network

---


### getAnnotationManager

```
public AnnotationManager getAnnotationManager()
```

:   **Description copied from class: `ModifierNetwork`**
:   Gets the `AnnotationManager` of the original network

    :   **Specified by:**: `getAnnotationManager` in interface `Network` **Overrides:**: `getAnnotationManager` in class `ModifierNetwork`
    :   **Returns:**: `AnnotationManager` of the original network

---


### getReactionName

```
public String getReactionName(int index)
```

:   **Description copied from class: `ModifierNetwork`**
:   Gets a string representation of the reactio in the original network.

    :   **Specified by:**: `getReactionName` in interface `Network` **Overrides:**: `getReactionName` in class `ModifierNetwork`
    :   **Parameters:**: `index` - index of the reaction in the original network **Returns:**: string represenation of the reaction


---


|  |  |  |  |  |  |  |  |  |  |  |
| --- | --- | --- | --- | --- | --- | --- | --- | --- | --- | --- |
| |  |  |  |  |  |  |  |  | | --- | --- | --- | --- | --- | --- | --- | --- | | **Overview** | **Package** | **Class** | **Use** | **Tree** | **Deprecated** | **Index** | **Help** | | |  |
| PREV CLASS   **NEXT CLASS** | **FRAMES**    **NO FRAMES**     **All Classes** |
| SUMMARY: NESTED | FIELD | CONSTR | METHOD | DETAIL: FIELD | CONSTR | METHOD |


---
