# Supplementary material for: FERN – a Java framework for stochastic simulation and evaluation of reaction networks
Source: BMC Bioinformatics. 2008 Aug 29;9:356. doi: 10.1186/1471-2105-9-356 (PMC2553347; doi:10.1186/1471-2105-9-356)
Supplement: Additional file 1 — FERN distribution, Version 1.3. This archive contains the FERN source code and binaries as well as documentation and example models in FernML and SBML. [file 1471-2105-9-356-S1.zip › fern/doc/javadoc/fern/network/modification/class-use/ModifierNetwork.html]

Uses of Class fern.network.modification.ModifierNetwork


---


|  |  |  |  |  |  |  |  |  |  |  |
| --- | --- | --- | --- | --- | --- | --- | --- | --- | --- | --- |
| |  |  |  |  |  |  |  |  | | --- | --- | --- | --- | --- | --- | --- | --- | | **Overview** | **Package** | **Class** | **Use** | **Tree** | **Deprecated** | **Index** | **Help** | | |  |
| PREV   NEXT | **FRAMES**    **NO FRAMES**     **All Classes** |


---


## **Uses of Class fern.network.modification.ModifierNetwork**

| Packages that use ModifierNetwork | |
| --- | --- |
| **fern.network.modification** | Provides classes for modifications of networks. |

| Uses of ModifierNetwork in fern.network.modification | |
| --- | --- |

| Subclasses of ModifierNetwork in fern.network.modification | |
| --- | --- |
| `class` | `CatalysedNetwork`             Modifies the network by adding reactions X+C -> Y+C (where C is each catalyst of the original reaction). |
| `class` | `ExtractSubNetwork`             Extracts some reactions / species from a given net to form a new network. |
| `class` | `ReversibleNetwork`             Doubles each reaction in a way that each original unidirectional reaction becomes reversible.As proposed by `ModifierNetwork`, the reactions are not copied but the indices are redirected. |

---


|  |  |  |  |  |  |  |  |  |  |  |
| --- | --- | --- | --- | --- | --- | --- | --- | --- | --- | --- |
| |  |  |  |  |  |  |  |  | | --- | --- | --- | --- | --- | --- | --- | --- | | **Overview** | **Package** | **Class** | **Use** | **Tree** | **Deprecated** | **Index** | **Help** | | |  |
| PREV   NEXT | **FRAMES**    **NO FRAMES**     **All Classes** |


---
