# Supplementary material for: FERN – a Java framework for stochastic simulation and evaluation of reaction networks
Source: BMC Bioinformatics. 2008 Aug 29;9:356. doi: 10.1186/1471-2105-9-356 (PMC2553347; doi:10.1186/1471-2105-9-356)
Supplement: Additional file 1 — FERN distribution, Version 1.3. This archive contains the FERN source code and binaries as well as documentation and example models in FernML and SBML. [file 1471-2105-9-356-S1.zip › fern/doc/javadoc/fern/network/modification/ExtractSubNetwork.html]

ExtractSubNetwork


---


|  |  |  |  |  |  |  |  |  |  |  |
| --- | --- | --- | --- | --- | --- | --- | --- | --- | --- | --- |
| |  |  |  |  |  |  |  |  | | --- | --- | --- | --- | --- | --- | --- | --- | | **Overview** | **Package** | **Class** | **Use** | **Tree** | **Deprecated** | **Index** | **Help** | | |  |
| **PREV CLASS**   **NEXT CLASS** | **FRAMES**    **NO FRAMES**     **All Classes** |
| SUMMARY: NESTED | FIELD | CONSTR | METHOD | DETAIL: FIELD | CONSTR | METHOD |


---


## fern.network.modification Class ExtractSubNetwork

```
java.lang.Object
  fern.network.modification.ModifierNetwork
      fern.network.modification.ExtractSubNetwork
```

**All Implemented Interfaces:**: Network

---

``` public class ExtractSubNetwork extends ModifierNetwork ```

Extracts some reactions / species from a given net to form a new network. As proposed by
`ModifierNetwork`, the network is not copied but the indices are redirected.

The subnet to be extracted has to be given by `BitVector`s containing a 1 for an index
to be in the extracted subnet.

This class can be used e.g. to extract only the autocatalytic set of an evolved network.

**Author:**
:   Florian Erhard

---

| **Constructor Summary** | |
| --- | --- |
| `ExtractSubNetwork(Network originalNet, BitVector reactions, BitVector species)`             Extracts a given subnet from a network. |


| **Method Summary** | |
| --- | --- |
| `AmountManager` | `getAmountManager()`             Gets the `AmountManager` for the extracted subnet. |
| `AnnotationManager` | `getAnnotationManager()`             Gets an `AnnotationManager` for the redirected index space. |
| `long` | `getInitialAmount(int species)`             Gets the initial amount of the specified molecule species. |
| `int` | `getNumReactions()`             Gets the number of reactions in the extracted subnet. |
| `int` | `getNumSpecies()`             Gets the number of species in the extracted subnet. |
| `protected  int` | `getOriginalReaction(int reaction)`             Redirects a reaction index from the subnet index space to the original index space |
| `protected  int` | `getOriginalSpecies(int species)`             Redirects a species index from the subnet index space to the original index space |
| `int[]` | `getProducts(int reaction)`             Gets the products of a reaction. |
| `PropensityCalculator` | `getPropensityCalculator()`             Gets the `PropensityCalculator` for the extracted subnet. |
| `int[]` | `getReactants(int reaction)`             Gets the reactants of a reaction. |
| `String` | `getReactionName(int index)`             Gets a string representation of the reaction |
| `int` | `getSpeciesByName(String name)`             Gets the index of the species by its name. |
| `String` | `getSpeciesName(int index)`             Gets the name of the species with given index. |
| `void` | `setInitialAmount(int species, long value)`             Sets the initial amount of the specified molecule species. |

| **Methods inherited from class fern.network.modification.ModifierNetwork** |
| --- |
| `getName, getOriginalNetwork, getParentNetwork` |

| **Methods inherited from class java.lang.Object** |
| --- |
| `clone, equals, finalize, getClass, hashCode, notify, notifyAll, toString, wait, wait, wait` |

| **Constructor Detail** |
| --- |

### ExtractSubNetwork

```
public ExtractSubNetwork(Network originalNet,
                         BitVector reactions,
                         BitVector species)
```

:   Extracts a given subnet from a network. The subnet has to be given by `BitVector`s containing a 1
    for each index which has to be in the extracted subnet.

    **Parameters:**: `originalNet` - network containing the subnet: `reactions` - reactions of the subnet: `species` - species of the subnet


| **Method Detail** |
| --- |

### getOriginalReaction

```
protected int getOriginalReaction(int reaction)
```

:   Redirects a reaction index from the subnet index space to the original index space

    :   **Parameters:**: `reaction` - index in subnet index space **Returns:**: index in original index space

---


### getOriginalSpecies

```
protected int getOriginalSpecies(int species)
```

:   Redirects a species index from the subnet index space to the original index space

    :   **Parameters:**: `species` - index in subnet index space **Returns:**: index in original index space

---


### getAnnotationManager

```
public AnnotationManager getAnnotationManager()
```

:   Gets an `AnnotationManager` for the redirected index space.

    :   **Specified by:**: `getAnnotationManager` in interface `Network` **Overrides:**: `getAnnotationManager` in class `ModifierNetwork`
    :   **Returns:**: the `AnnotationManager` object

---


### getNumReactions

```
public int getNumReactions()
```

:   Gets the number of reactions in the extracted subnet.

    :   **Specified by:**: `getNumReactions` in interface `Network` **Overrides:**: `getNumReactions` in class `ModifierNetwork`
    :   **Returns:**: number of reactions

---


### getNumSpecies

```
public int getNumSpecies()
```

:   Gets the number of species in the extracted subnet.

    :   **Specified by:**: `getNumSpecies` in interface `Network` **Overrides:**: `getNumSpecies` in class `ModifierNetwork`
    :   **Returns:**: number of species

---


### getProducts

```
public int[] getProducts(int reaction)
```

:   Gets the products of a reaction.

    :   **Specified by:**: `getProducts` in interface `Network` **Overrides:**: `getProducts` in class `ModifierNetwork`
    :   **Parameters:**: `reaction` - the index of the reaction **Returns:**: indices of the products

---


### getReactants

```
public int[] getReactants(int reaction)
```

:   Gets the reactants of a reaction.

    :   **Specified by:**: `getReactants` in interface `Network` **Overrides:**: `getReactants` in class `ModifierNetwork`
    :   **Parameters:**: `reaction` - the index of the reaction **Returns:**: indices of the reactants

---


### getInitialAmount

```
public long getInitialAmount(int species)
```

:   **Description copied from interface: `Network`**
:   Gets the initial amount of the specified molecule species.

    :   **Specified by:**: `getInitialAmount` in interface `Network` **Overrides:**: `getInitialAmount` in class `ModifierNetwork`
    :   **Parameters:**: `species` - index of the species **Returns:**: initial amount of the species

---


### setInitialAmount

```
public void setInitialAmount(int species,
                             long value)
```

:   **Description copied from interface: `Network`**
:   Sets the initial amount of the specified molecule species.

    :   **Specified by:**: `setInitialAmount` in interface `Network` **Overrides:**: `setInitialAmount` in class `ModifierNetwork`
    :   **Parameters:**: `species` - index of the species: `value` - initial amount of the species

---


### getSpeciesName

```
public String getSpeciesName(int index)
```

:   Gets the name of the species with given index.

    :   **Specified by:**: `getSpeciesName` in interface `Network` **Overrides:**: `getSpeciesName` in class `ModifierNetwork`
    :   **Parameters:**: `index` - index of the species **Returns:**: name of the species

---


### getSpeciesByName

```
public int getSpeciesByName(String name)
```

:   Gets the index of the species by its name.

    :   **Specified by:**: `getSpeciesByName` in interface `Network` **Overrides:**: `getSpeciesByName` in class `ModifierNetwork`
    :   **Parameters:**: `name` - name of the species **Returns:**: index of the species

---


### getAmountManager

```
public AmountManager getAmountManager()
```

:   Gets the `AmountManager` for the extracted subnet.

    :   **Specified by:**: `getAmountManager` in interface `Network` **Overrides:**: `getAmountManager` in class `ModifierNetwork`
    :   **Returns:**: the amount manager

---


### getPropensityCalculator

```
public PropensityCalculator getPropensityCalculator()
```

:   Gets the `PropensityCalculator` for the extracted subnet.

    :   **Specified by:**: `getPropensityCalculator` in interface `Network` **Overrides:**: `getPropensityCalculator` in class `ModifierNetwork`
    :   **Returns:**: the propensity calculator

---


### getReactionName

```
public String getReactionName(int index)
```

:   Gets a string representation of the reaction

    :   **Specified by:**: `getReactionName` in interface `Network` **Overrides:**: `getReactionName` in class `ModifierNetwork`
    :   **Parameters:**: `index` - index of the reaction **Returns:**: string representation of the reaction


---


|  |  |  |  |  |  |  |  |  |  |  |
| --- | --- | --- | --- | --- | --- | --- | --- | --- | --- | --- |
| |  |  |  |  |  |  |  |  | | --- | --- | --- | --- | --- | --- | --- | --- | | **Overview** | **Package** | **Class** | **Use** | **Tree** | **Deprecated** | **Index** | **Help** | | |  |
| **PREV CLASS**   **NEXT CLASS** | **FRAMES**    **NO FRAMES**     **All Classes** |
| SUMMARY: NESTED | FIELD | CONSTR | METHOD | DETAIL: FIELD | CONSTR | METHOD |


---
