# Supplementary material for: FERN – a Java framework for stochastic simulation and evaluation of reaction networks
Source: BMC Bioinformatics. 2008 Aug 29;9:356. doi: 10.1186/1471-2105-9-356 (PMC2553347; doi:10.1186/1471-2105-9-356)
Supplement: Additional file 3 — Supplementary Figures. This file contains Supplementary Figures on FernML, the simulation cycle, the Cytoscape plugin and runtime comparisons between FERN and gillespie2. [file 1471-2105-9-356-S3.pdf]

Supplementary Figures

Supplementary Figure 1 - Example FernML

The FernML code is shown for a small example. Here, two substrates, S_1 and S_2 react via a single interaction to a product P. The initial amounts of S_1, S_2 and P are set to 30, 20 and 0, respectively. FernML supports only the reaction rate equations used by Gillespie [1]. Thus, in the given example the reaction rate equation is $\frac{dP}{dt} = k[S_1][S_2]$ with a kinetic constant $k = 0.01$.

```xml
<fernml version="1.0">
  <listOfSpecies>
    <species initialAmount="30" name="S1">
      <listOfAnnotations>
        <annotation name="Description">Substrate 1</annotation>
      </listOfAnnotations>
    </species>
    <species initialAmount="20" name="S2"/>
    <species initialAmount="0" name="P"/>
  </listOfSpecies>
  <listOfReactions>
    <reaction kineticConstant="0.01">
      <listOfReactants>
        <speciesReference name="S1"/>
        <speciesReference name="S2"/>
      </listOfReactants>
      <listOfProducts>
        <speciesReference name="P"/>
      </listOfProducts>
    </reaction>
  </listOfReactions>
</fernml>
```

Supplementary Figure 2 - Simulation cycle

This figure illustrates the simulation cycle and at what points in the simulation observer methods are invoked. Observers are notified at the beginning of each step (call to *step()* method of observer), as soon as a registered point of time is crossed (*thetaEvent()*), when an reaction is fired(*fireReaction()*) and at the beginning and end of each simulation (*start()*, *finished()* and *print()*). The simulation is performed step by step (one reaction for exact methods, several reactions for tau-leaping) until the simulation controller stops the simulation. The individual simulation algorithms implement only the *performStep()* method.

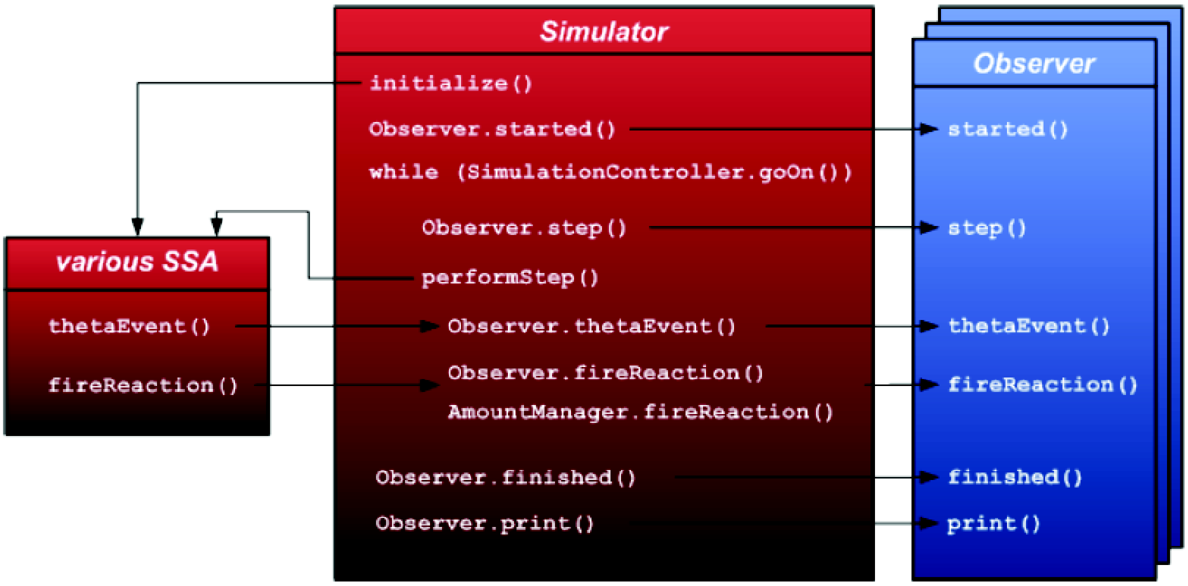

Supplementary Figure 3 - Cytoscape plugin

This figure shows the user interface of the Cytoscape [2] plugin of FERN. Networks can be either loaded directly into Cytoscape or via the plugin. In the first case, the attributes identifying the species and reactions, molecular numbers for species and reaction constants have to be specified. Exact, approximative and hybrid simulation algorithms can be chosen and the simulation progress can be visualized both on the Cytoscape network and with gnuplot.

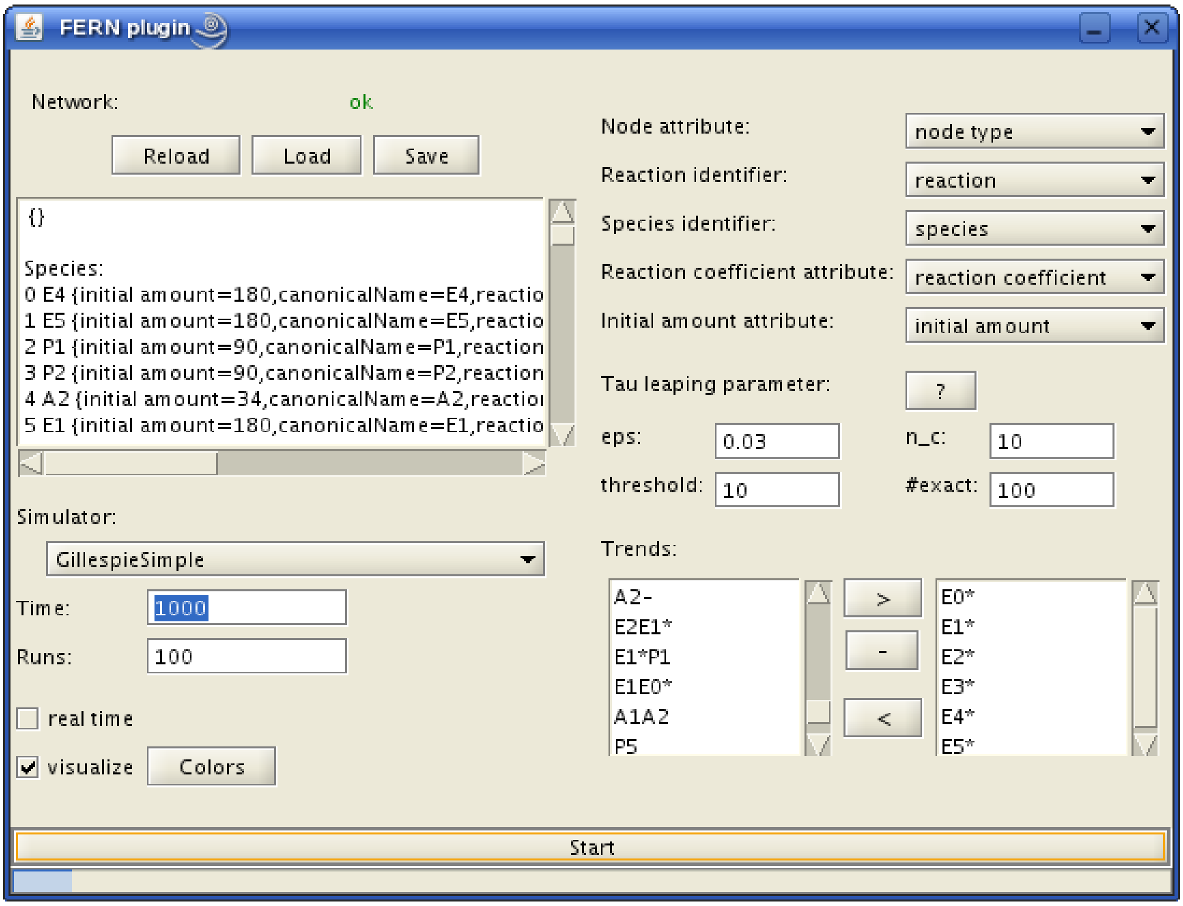

Supplementary Figure 4 - Runtime Comparison on DSMTS models

We compared the runtime of the implementations of the original Gillespie algorithm of FERN and the C implementations of the gillespie2 program [3] on the Discrete Stochastic Models Test Suite (DSMTS) [4] (left hand side) as well as the EGF signaling model described by Lee et al. [5] (right hand side). DSMTS models were simulated 10,000 times for a simulated time of 50 seconds and the EGF model 1,000 times for a simulated time of 800 seconds. All models were simulated as SBML networks in FERN. For the DSMTS model the ratio of gillespie2 runtime to FERN runtime is shown (blue). The green horizontal line indicates equal runtime, above the line FERN is faster and below the line gillespie2. As can be seen, FERN is significantly faster than gillespie2 for all DSMTS models except Model 1.14. For this model, gillespie2 is only faster because it does not parse the rate law correctly and, thus, simulates a wrong model in which molecule concentrations decrease very rapidly. On average, FERN is almost twice as fast as gillespie2.

For the EGF model, average runtime in milliseconds is given. Again, FERN is significantly faster than gillespie2 by a factor of 3.6.

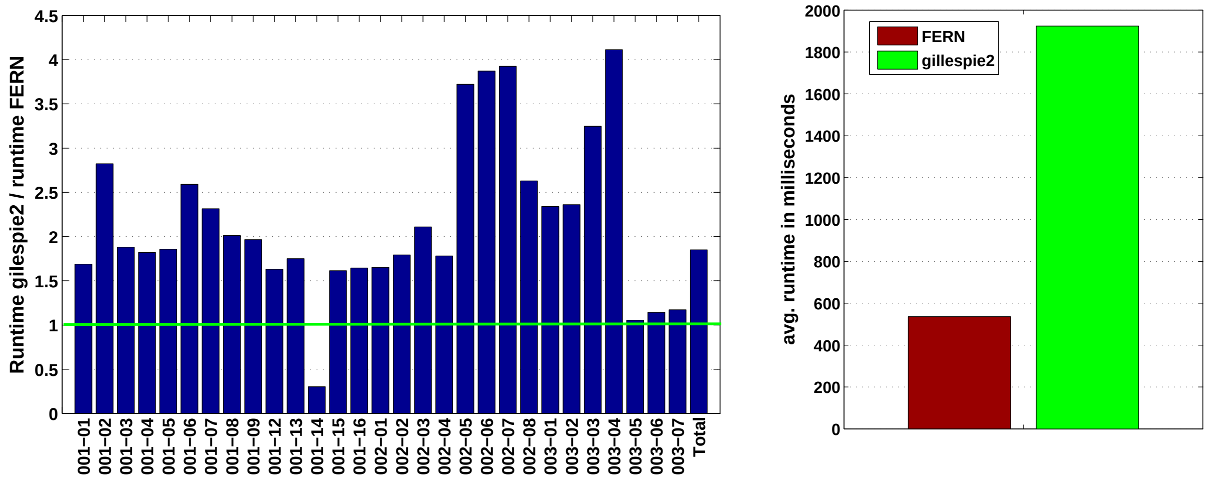

References

[1] Gillespie DT: **A general method for numerically simulating the stochastic time evolution of coupled chemical reactions**. *Journal of Computational Physics* 1976, **22**(4):403–434.

[2] Shannon P, Markiel A, Ozier O, Baliga NS, Wang JT, Ramage D, Amin N, Schwikowski B, Ideker T: **Cytoscape: a software environment for integrated models of biomolecular interaction networks.** *Genome Res* 2003, **13**(11):2498–2504.

[3] Gillespie CS, Wilkinson DJ, Proctor CJ, Shanley DP, Boys RJ, Kirkwood TBL: **Tools for the SBML Community.** *Bioinformatics* 2006, **22**(5):628–629.

[4] Evans TW, Gillespie CS, Wilkinson DJ: **The SBML Discrete Stochastic Models Test Suite.** *Bioinformatics* 2007.

[5] Lee DY, Zimmer R, Lee SY, Park S: **Colored Petri net modeling and simulation of signal transduction pathways.** *Metab Eng* 2006, **8**(2):112–122.
